# Supplementary material for: Stereodivergent synthesis of vicinal quaternary-quaternary stereocenters and bioactive hyperolactones
Source: Nat Commun. 2018 May 17;9:1968. doi: 10.1038/s41467-018-04123-w (PMC5958103; doi:10.1038/s41467-018-04123-w)
Supplement: Supplementary file 1 — Supplementary Information [file 41467_2018_4123_MOESM1_ESM.pdf]

Stereodivergent Synthesis of Vicinal Quaternary-Quaternary Stereocenters and Bioactive Hyperolactones

Haifeng Zheng,[1] Yan Wang,[1] Chaoran Xu,[1] Xi Xu,[1] Lili Lin,[1] Xiaohua Liu,[1,*] and Xiaoming Feng[1,2,*]

[1]Key Laboratory of Green Chemistry & Technology, Ministry of Education, College of Chemistry, Sichuan University, Chengdu 610064, China.
[2]Collaborative Innovation Center of Chemical Science and Engineering (Tianjin), China.

Supplementary Figures

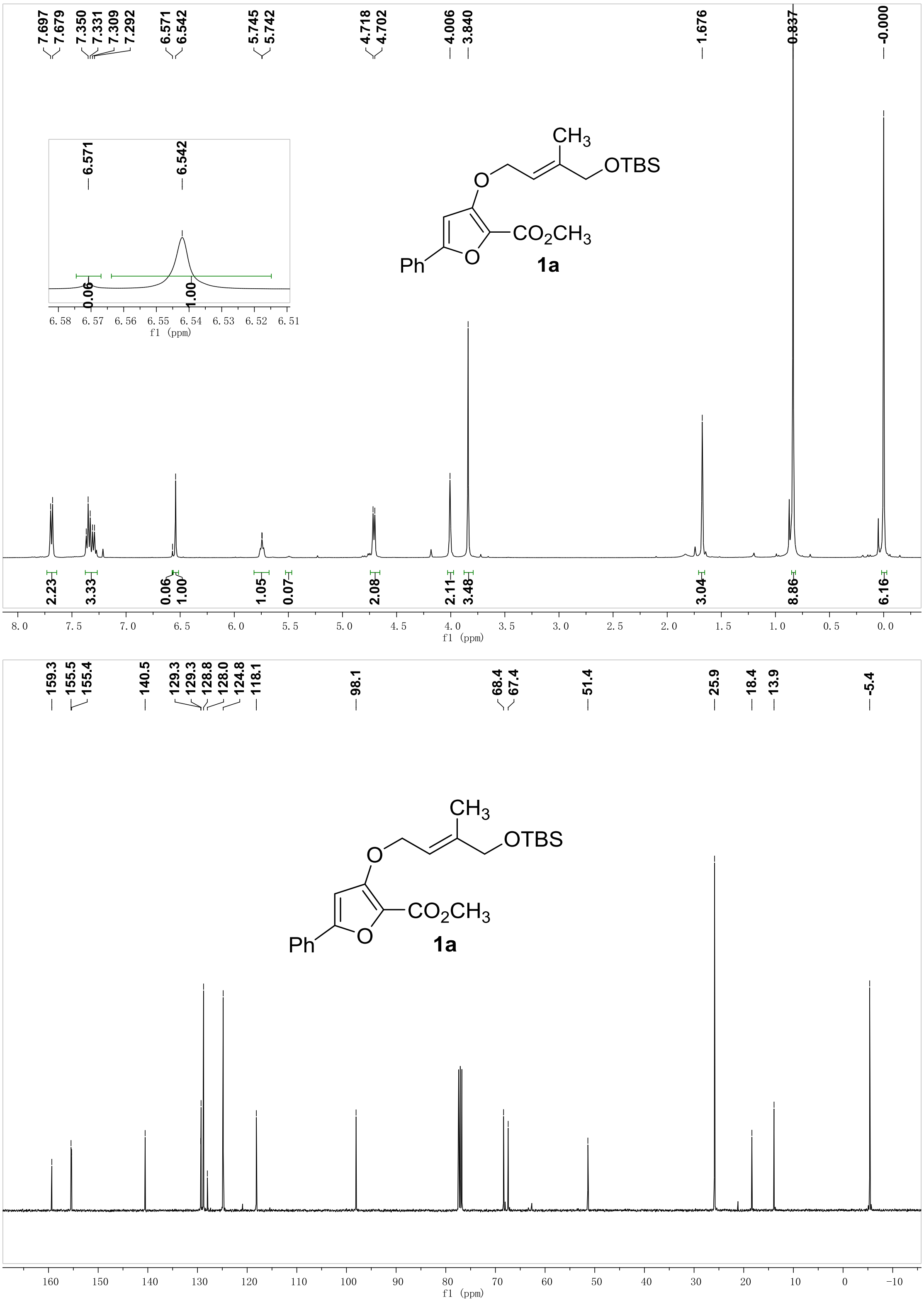

Supplementary Figure 1. ^{1}H and ^{13}C spectra for substrate **1a**

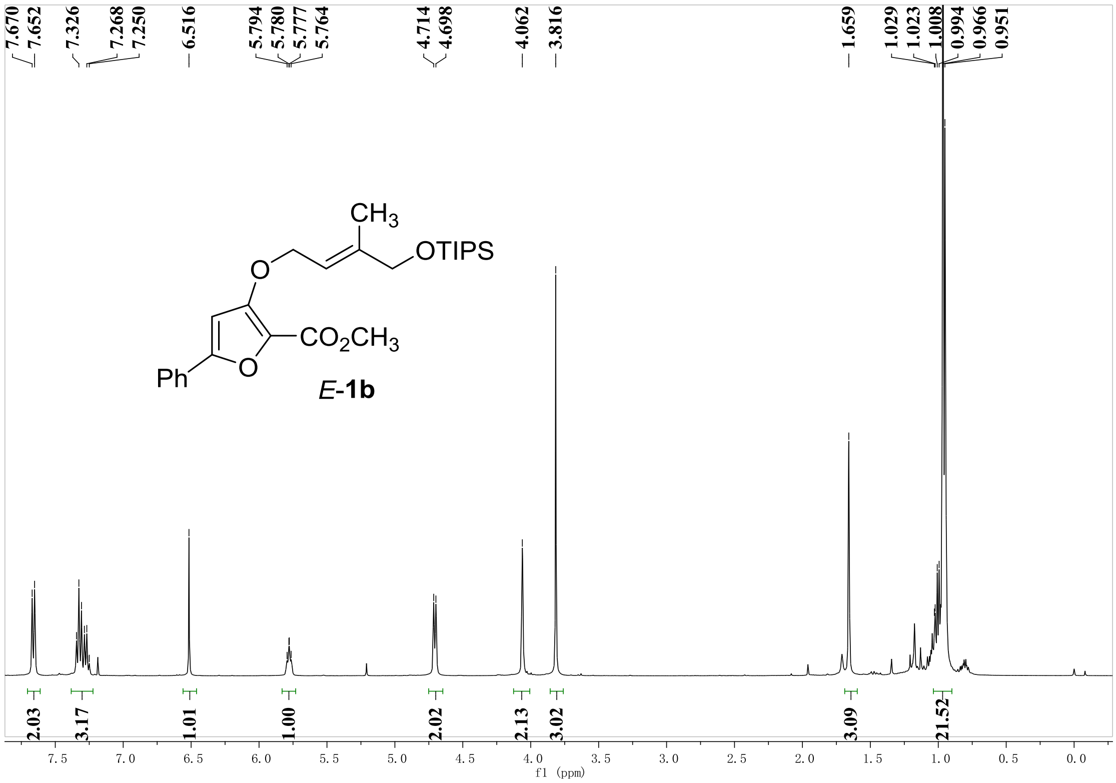

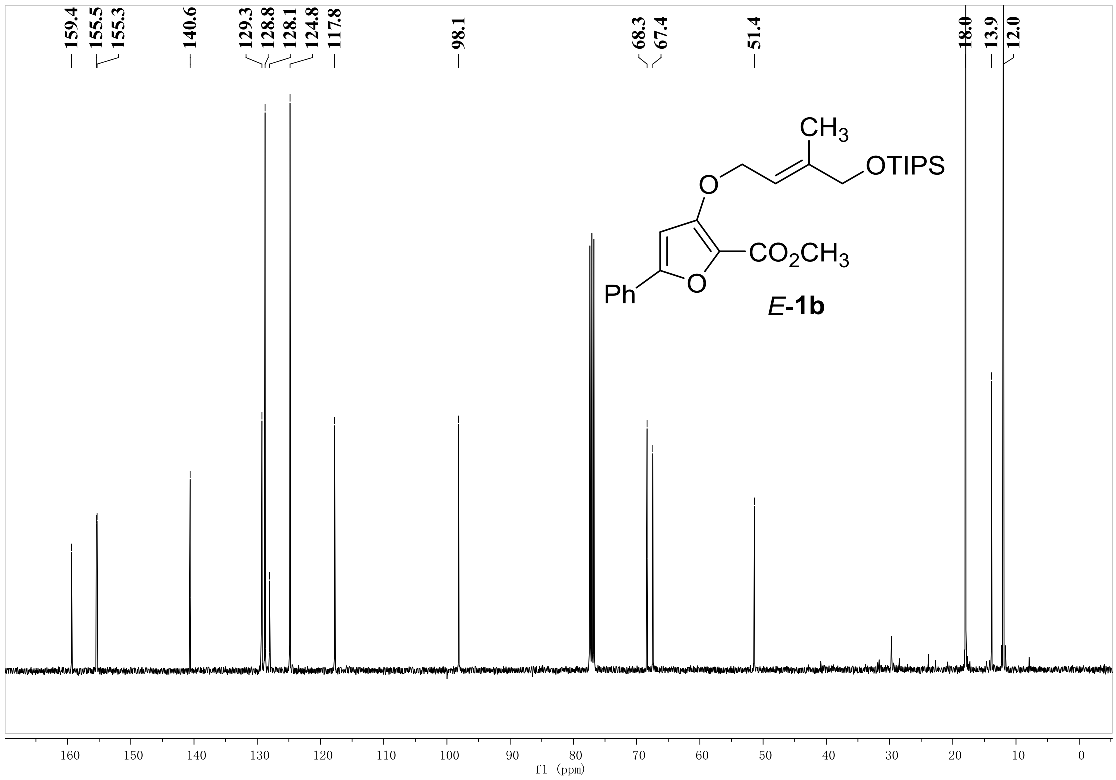

Supplementary Figure 2. ^{1}H and ^{13}C spectra for substrate *E*-**1b**

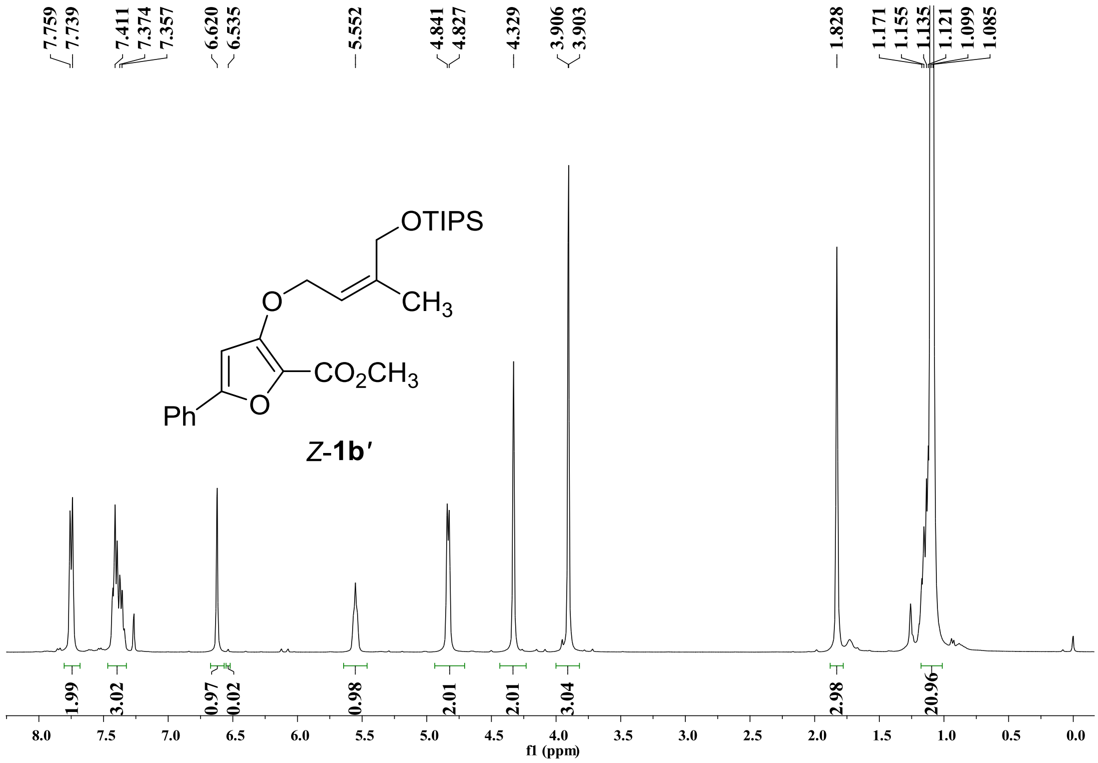

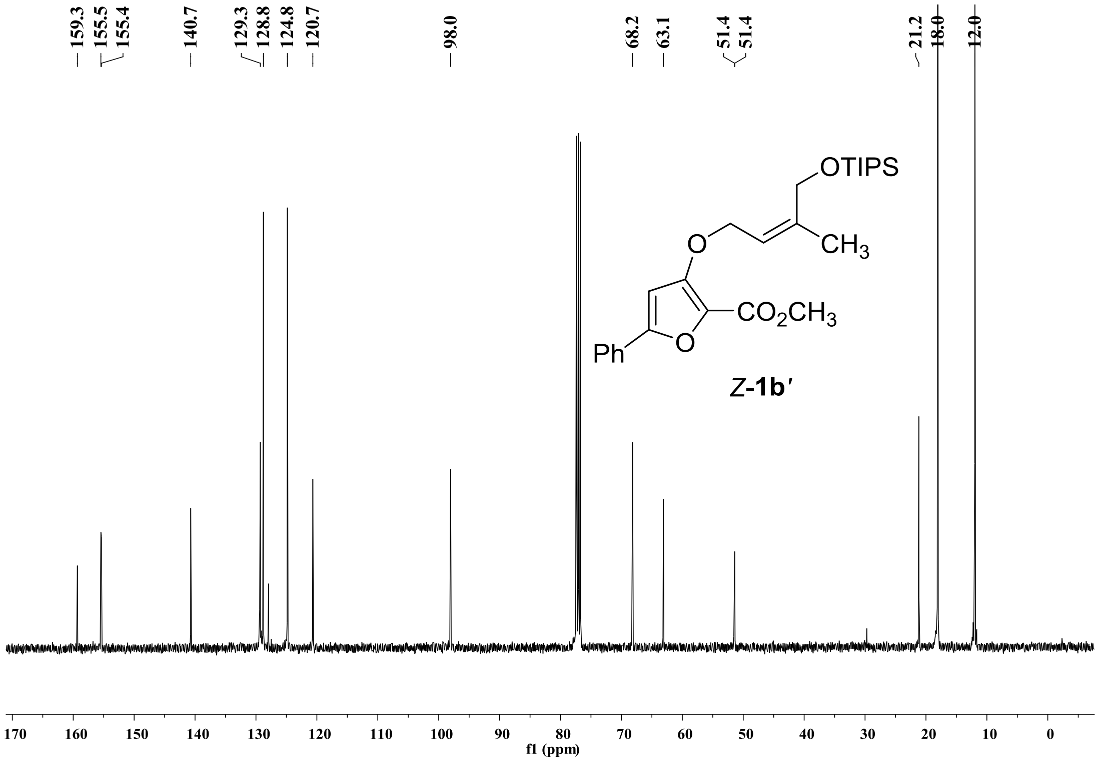

Supplementary Figure 3. ^{1}H and ^{13}C spectra for substrate *Z*-**1b'**

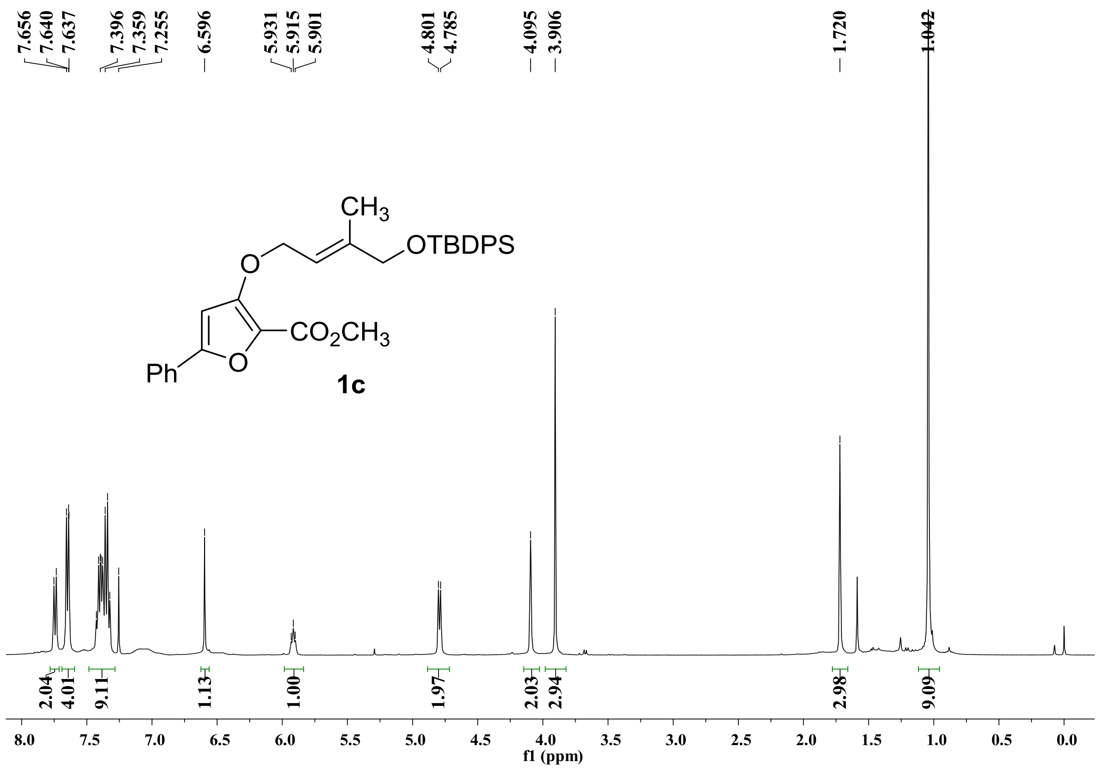

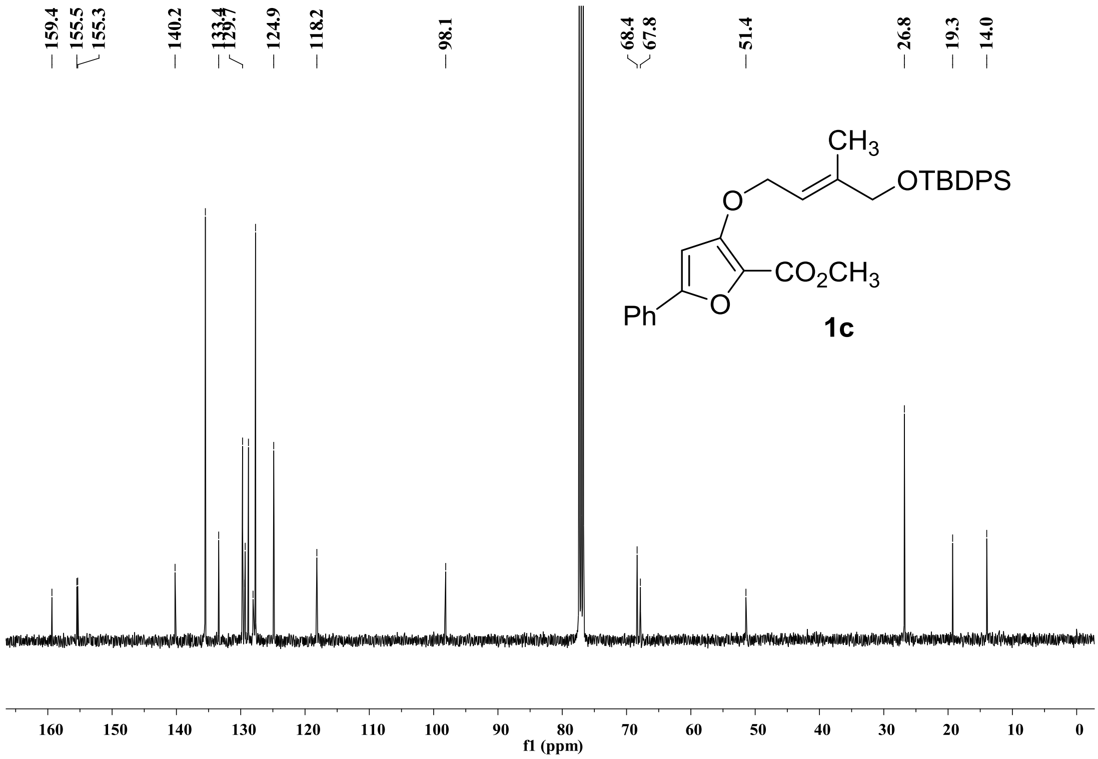

Supplementary Figure 4. ^{1}H and ^{13}C spectra for substrate **1c**

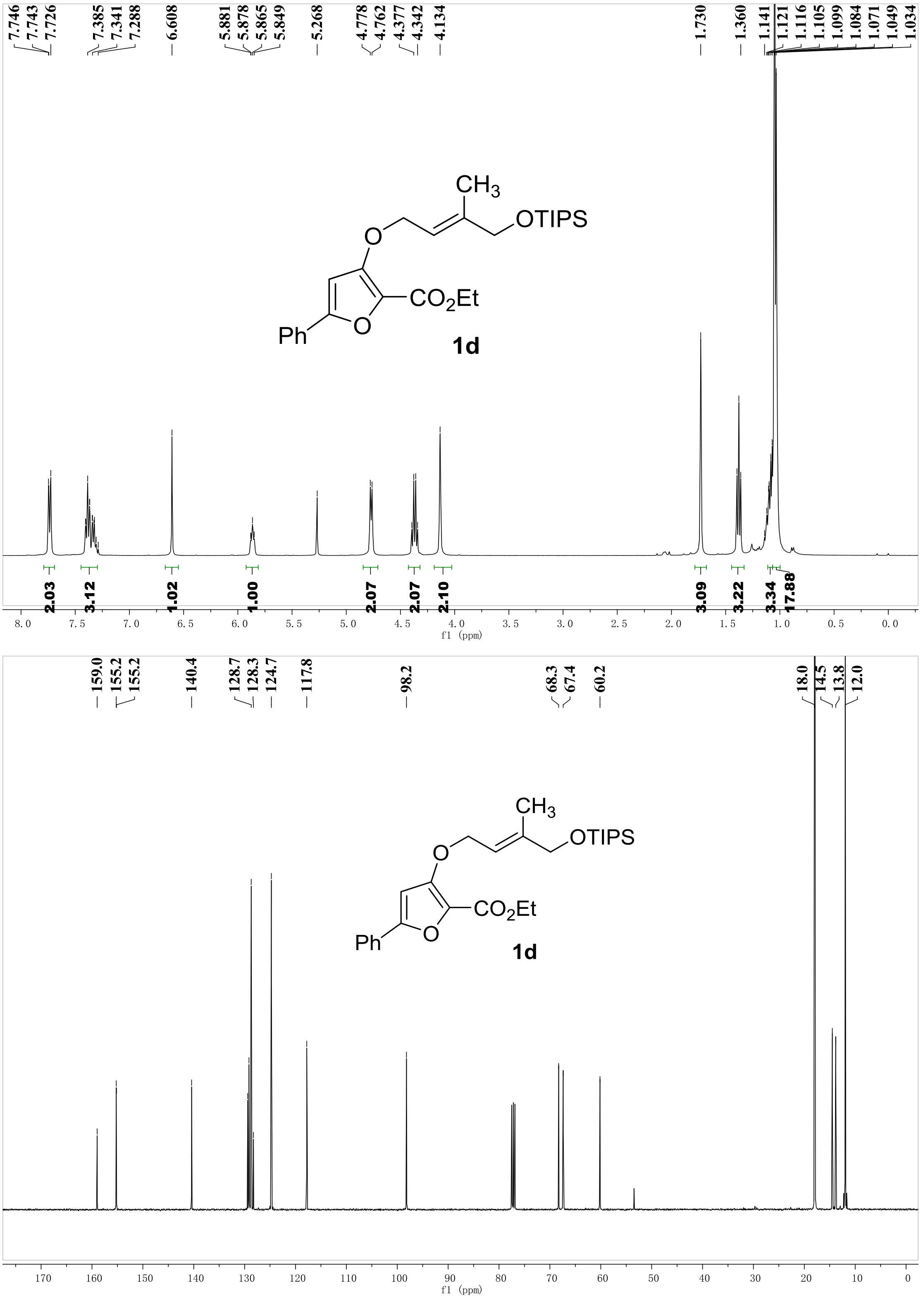

Supplementary Figure 5. ^{1}H and ^{13}C spectra for substrate **1d**

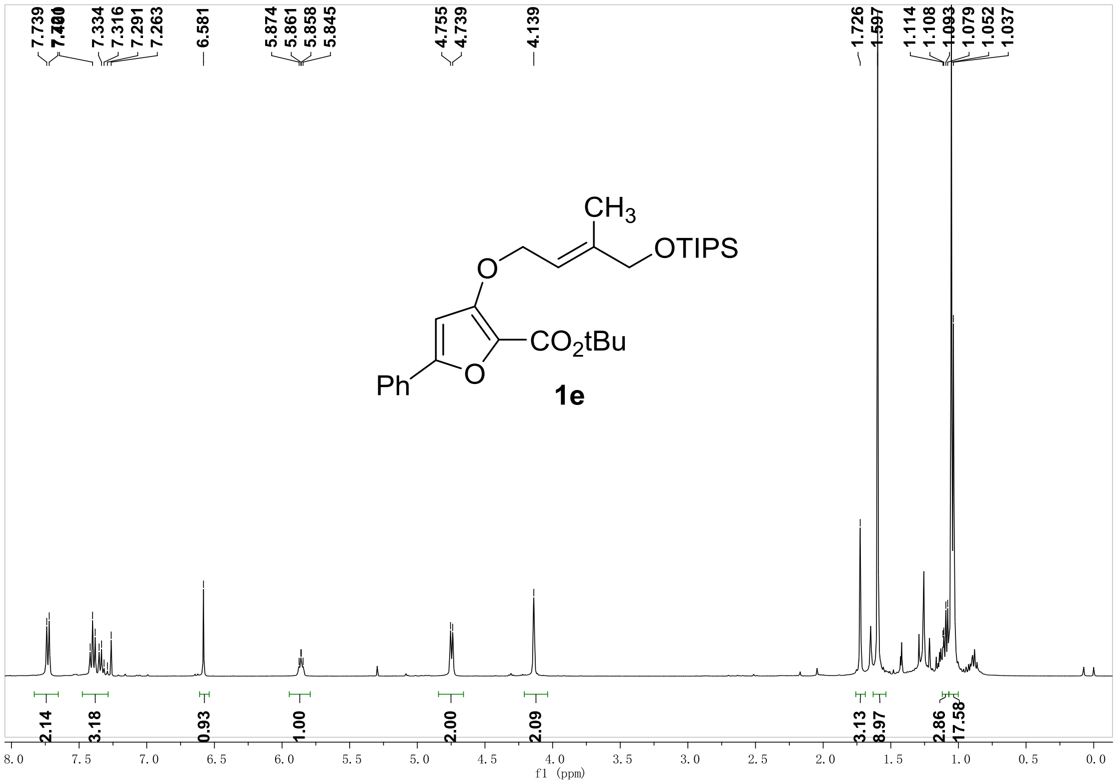

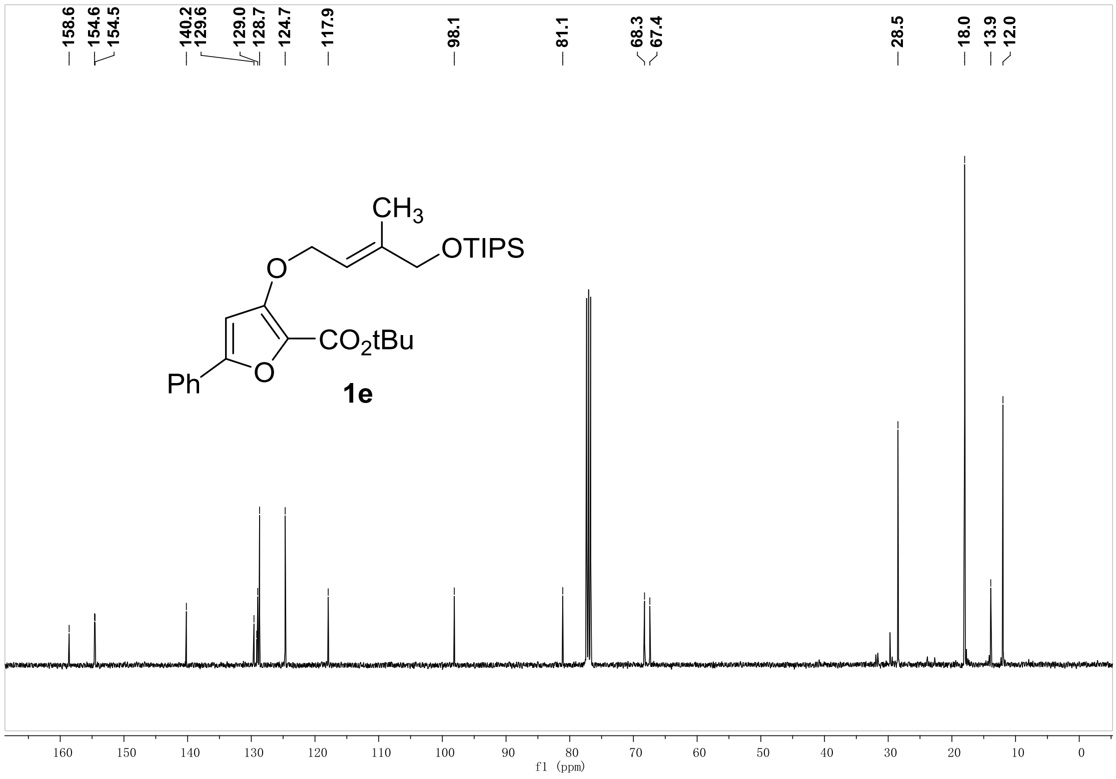

Supplementary Figure 6. ^{1}H and ^{13}C spectra for substrate **1e**

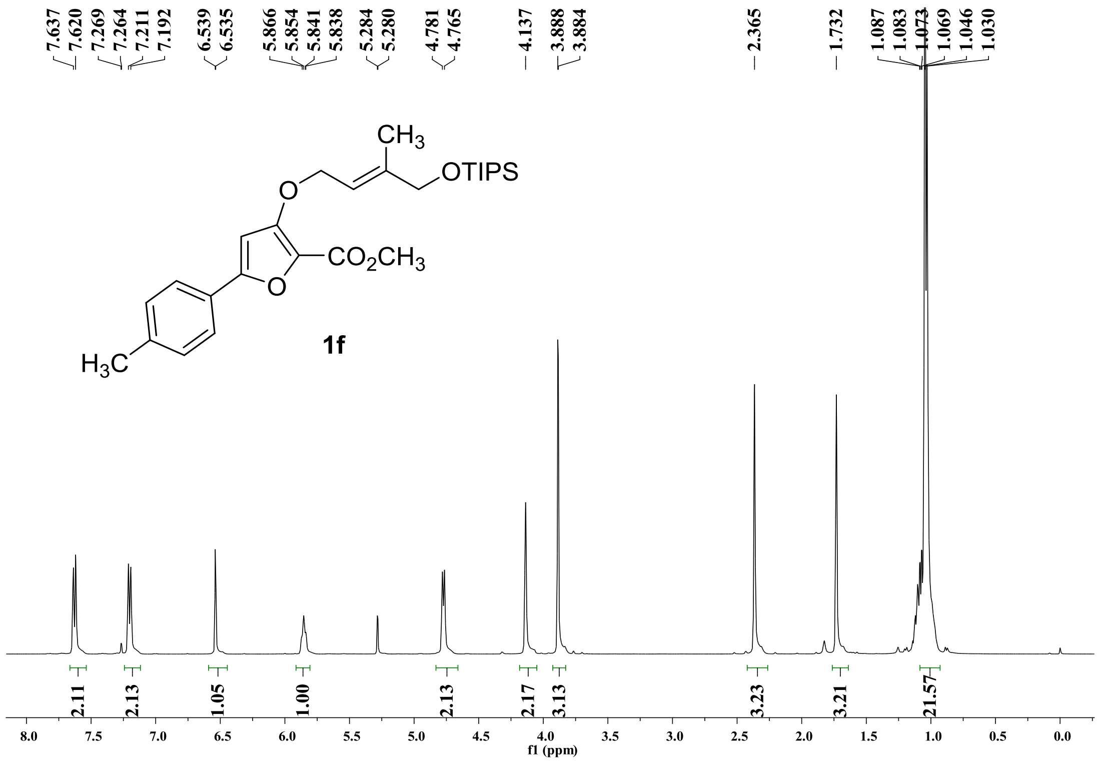

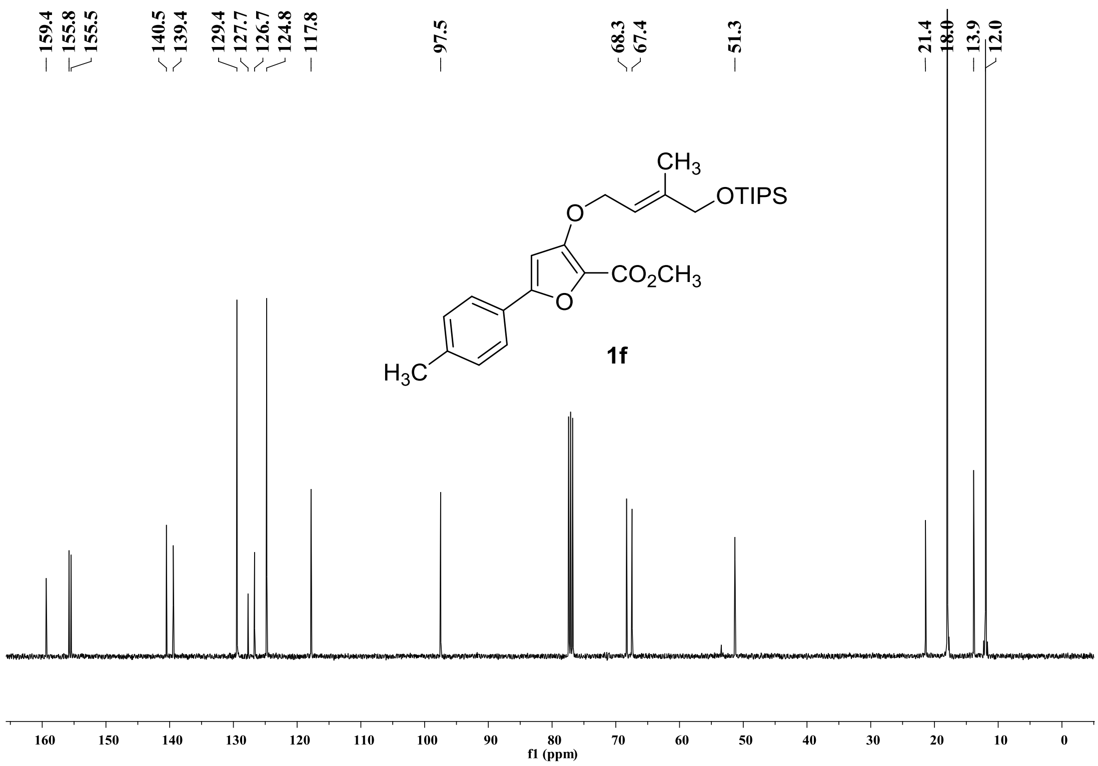

Supplementary Figure 7. ^{1}H and ^{13}C spectra for substrate **1f**

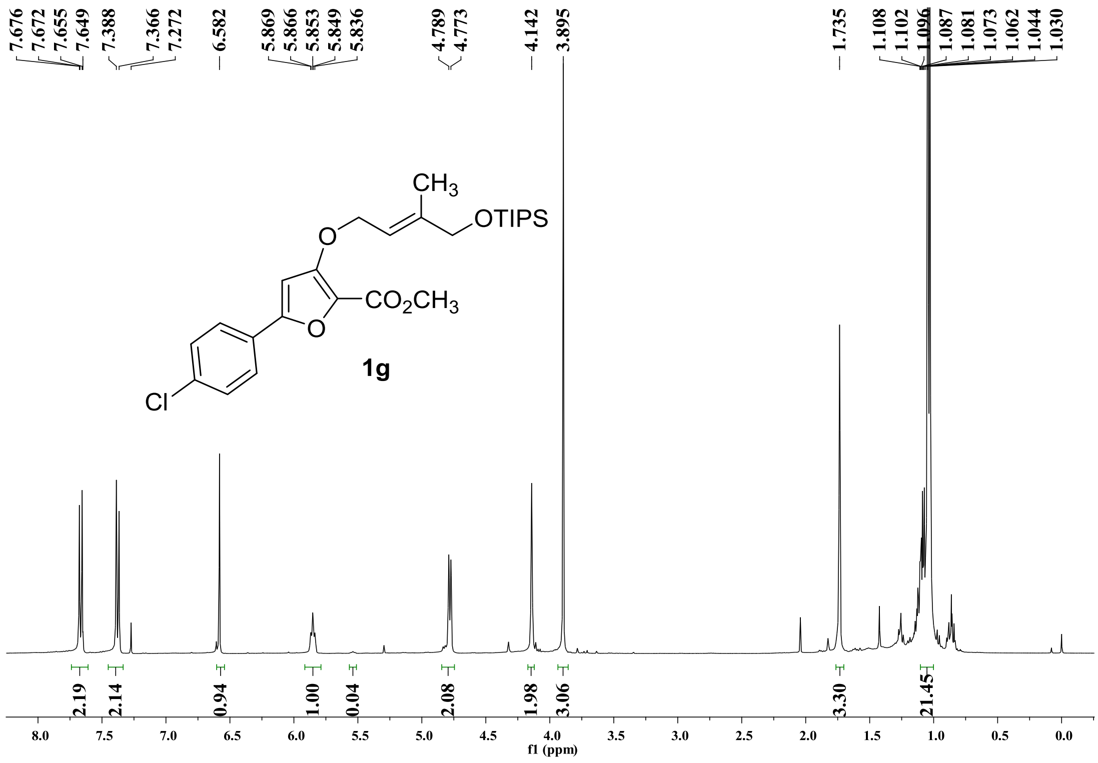

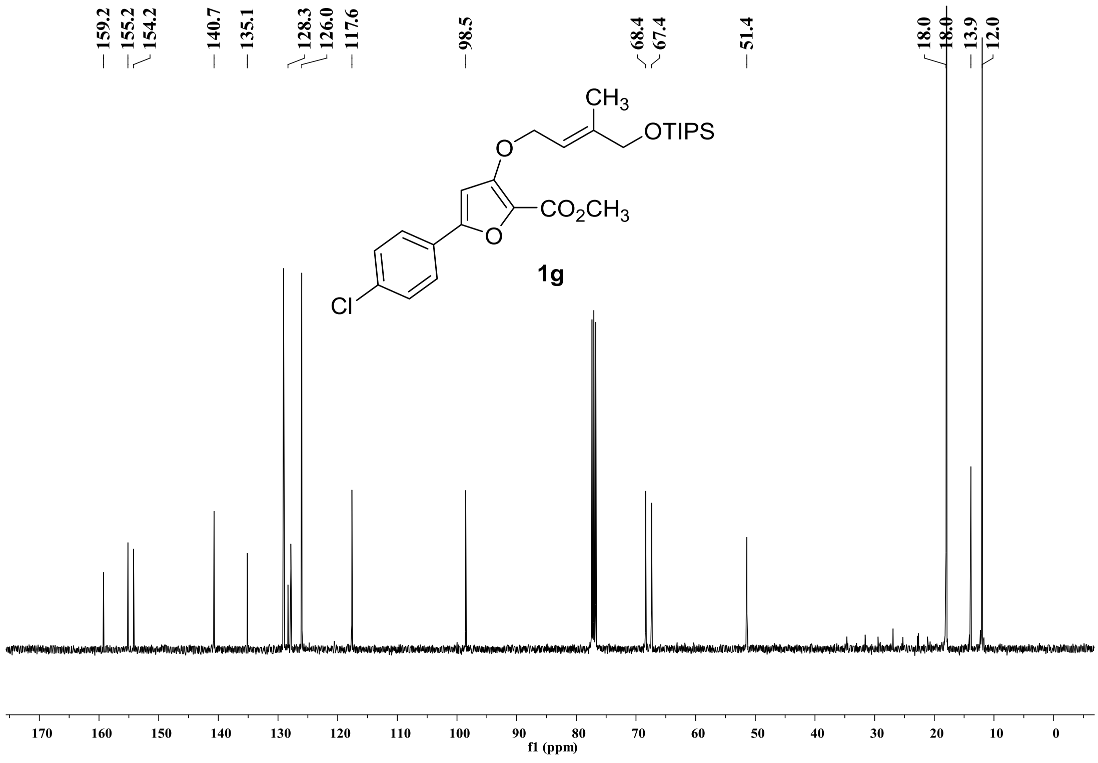

Supplementary Figure 8. ^{1}H and ^{13}C spectra for substrate **1g**

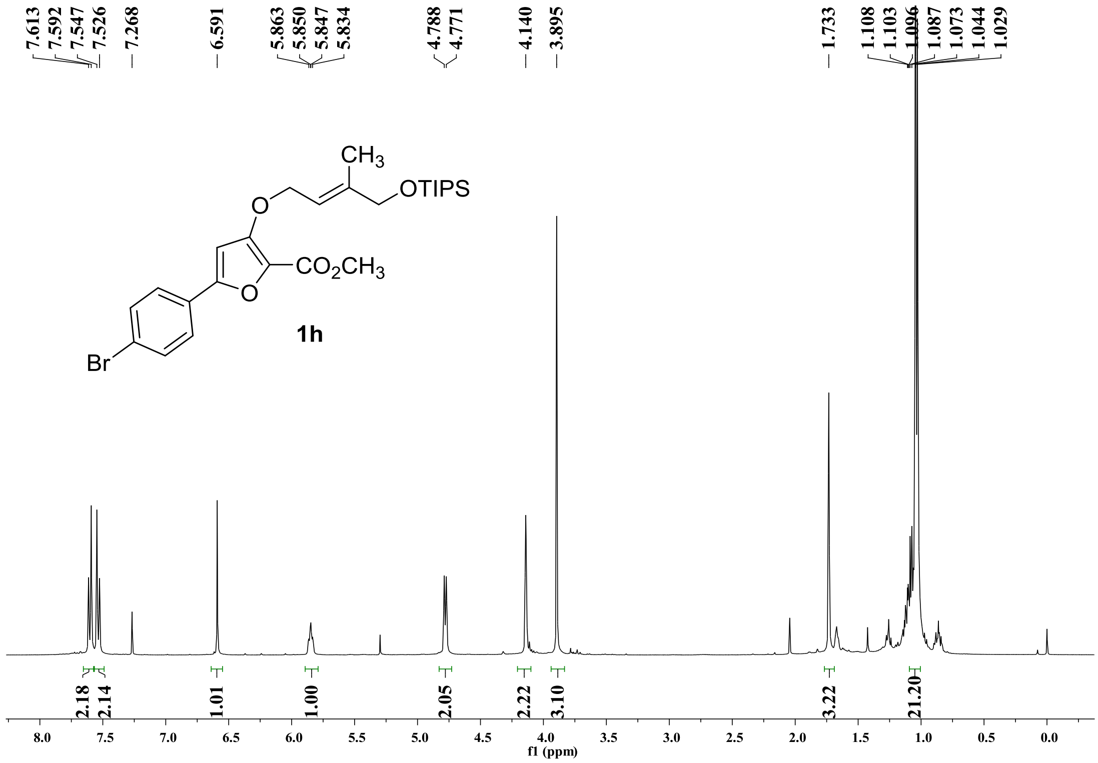

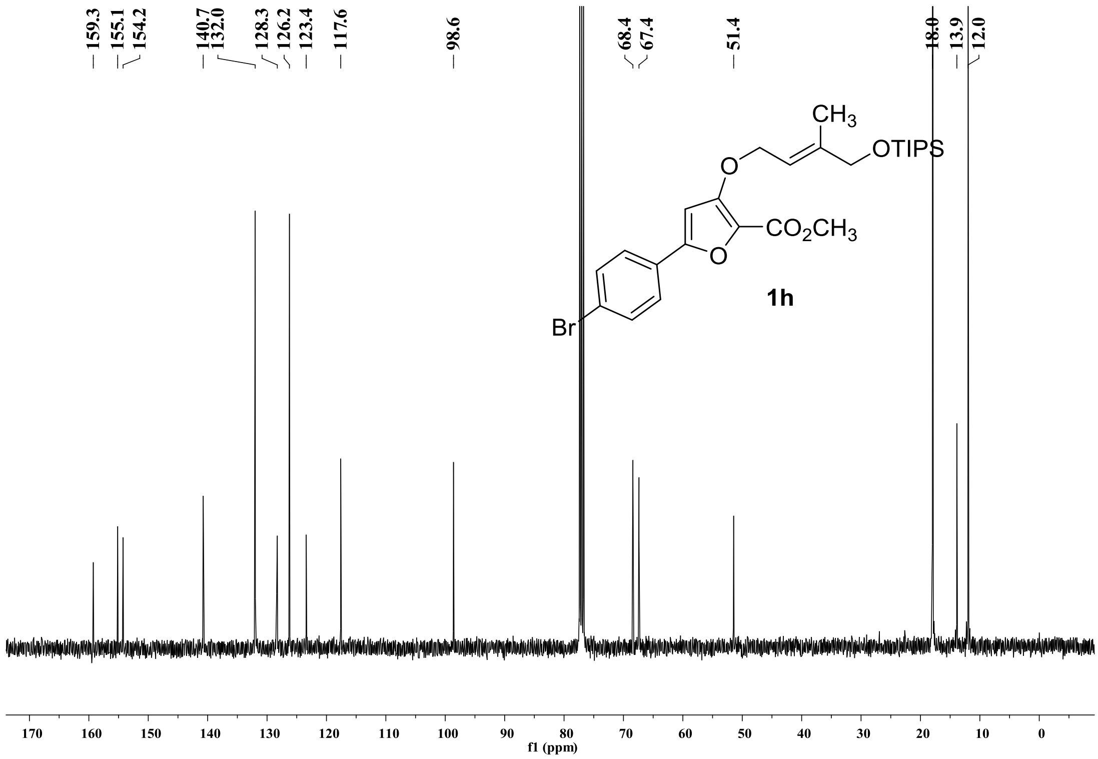

Supplementary Figure 9. ^{1}H and ^{13}C spectra for substrate **1h**

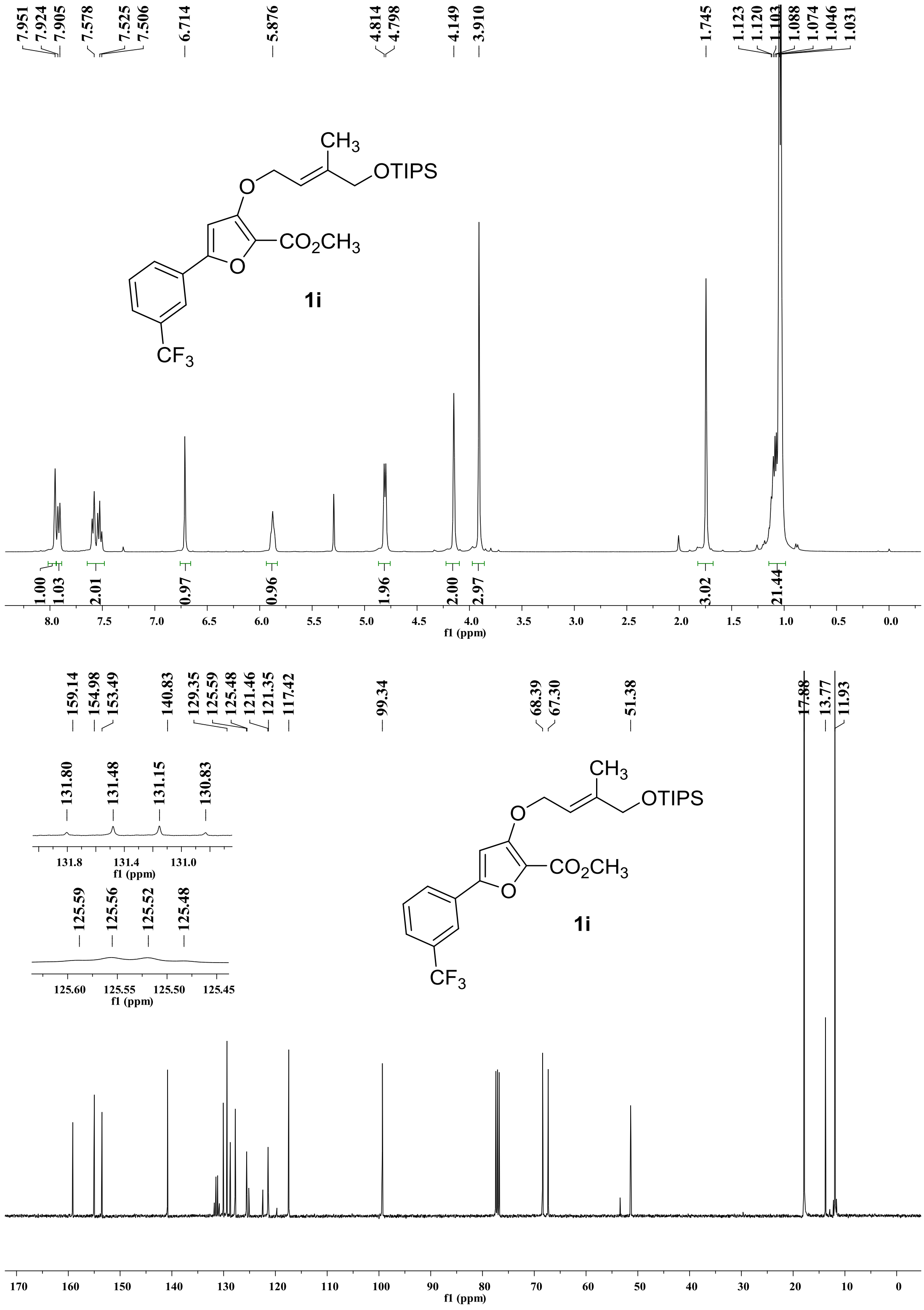

Supplementary Figure 10. ^{1}H and ^{13}C spectra for substrate **1i**

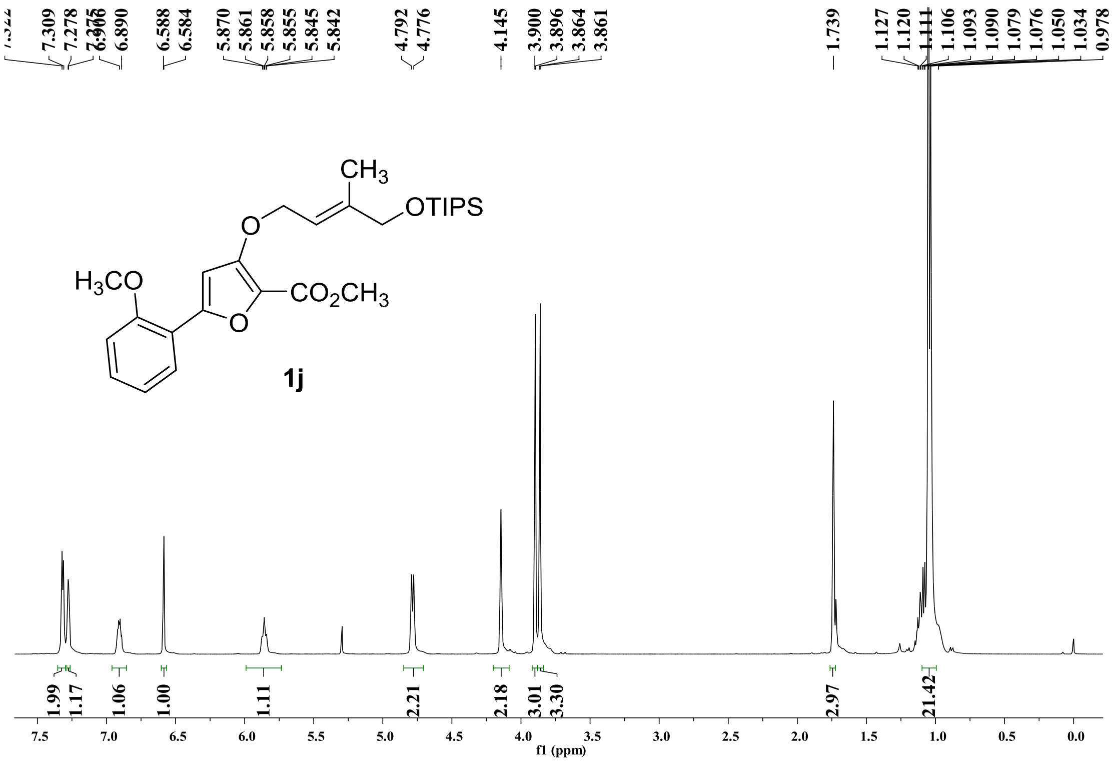

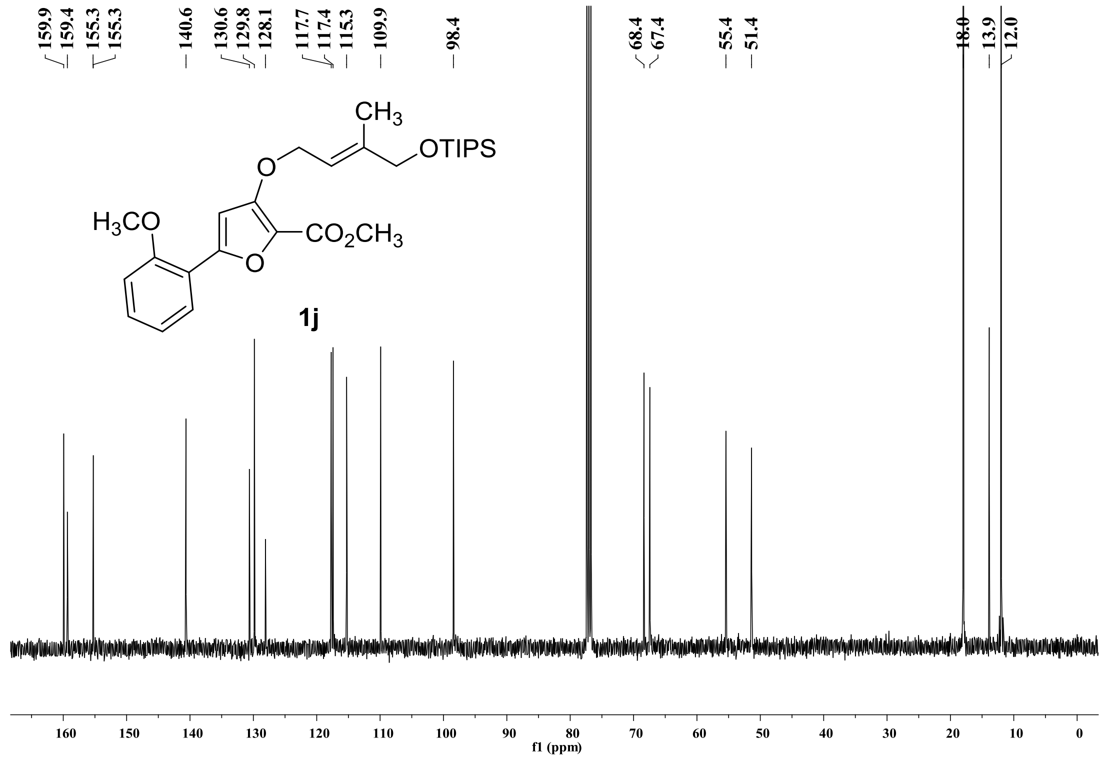

Supplementary Figure 11. ^{1}H and ^{13}C spectra for substrate **1j**

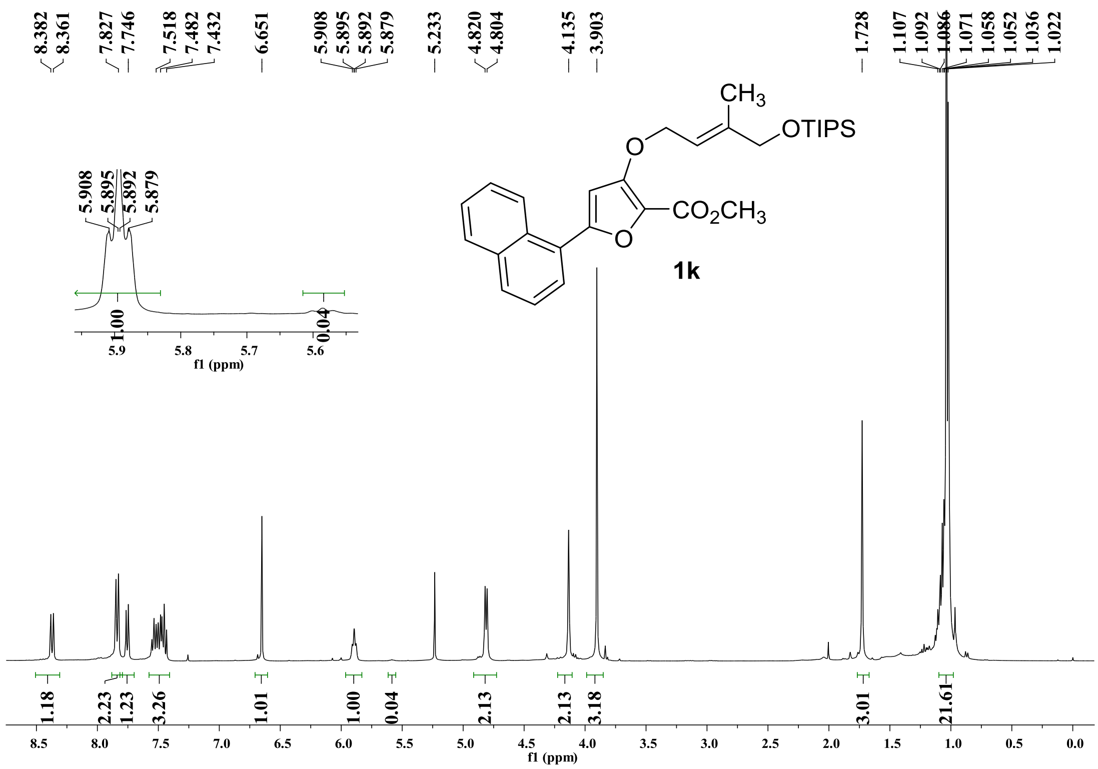

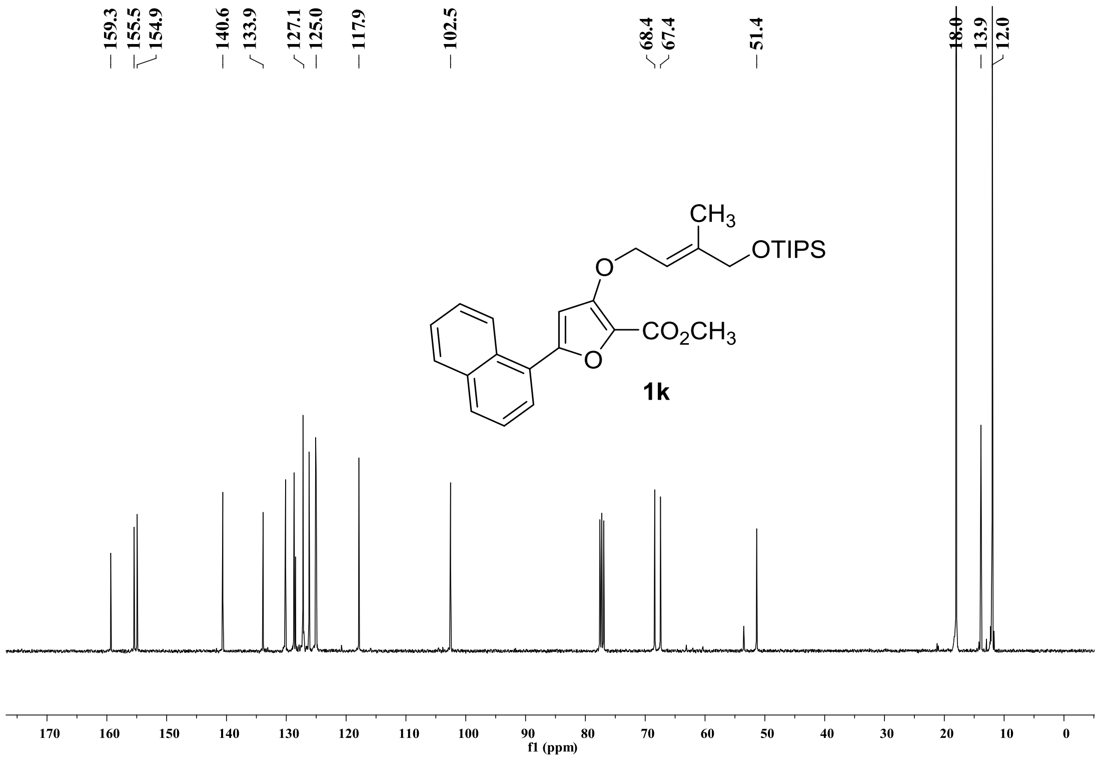

Supplementary Figure 12. ^{1}H and ^{13}C spectra for substrate **1k**

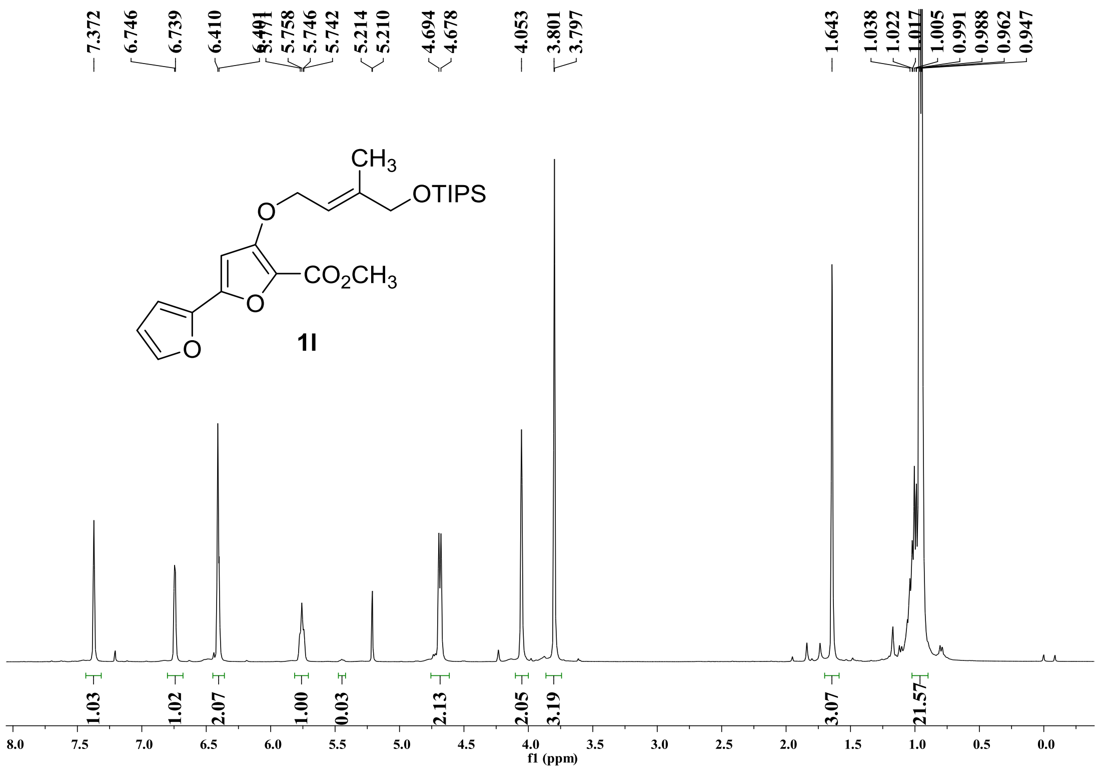

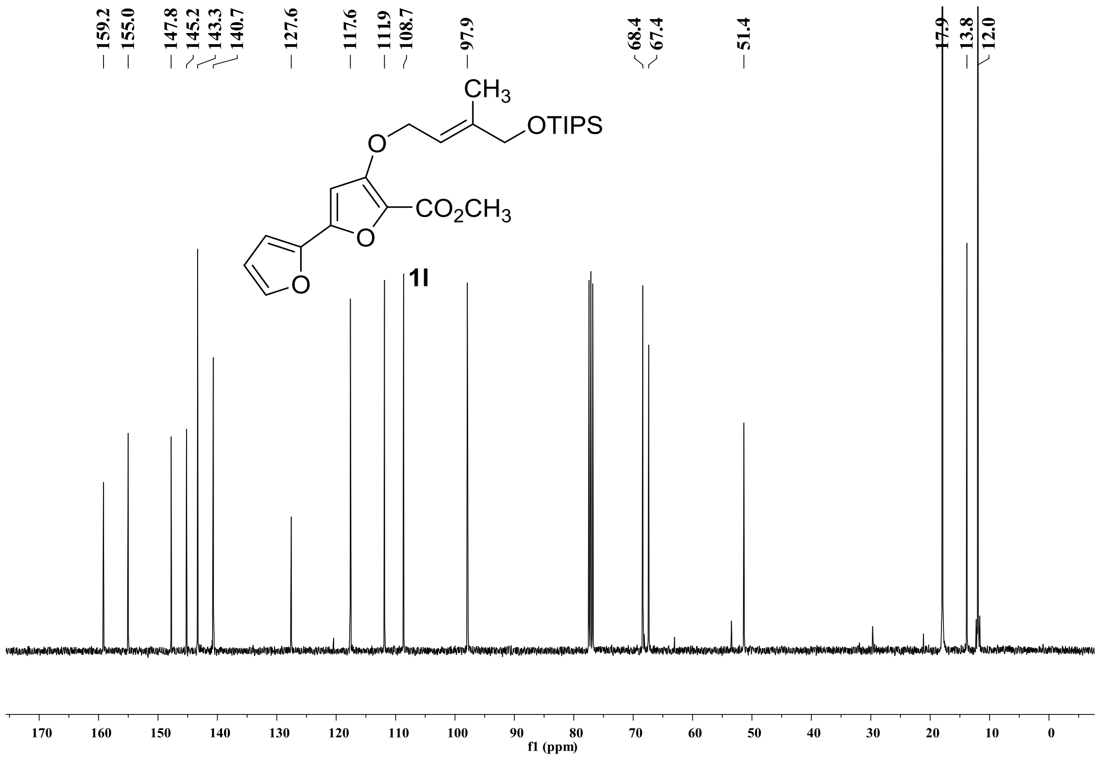

Supplementary Figure 13. ^{1}H and ^{13}C spectra for substrate **1l**

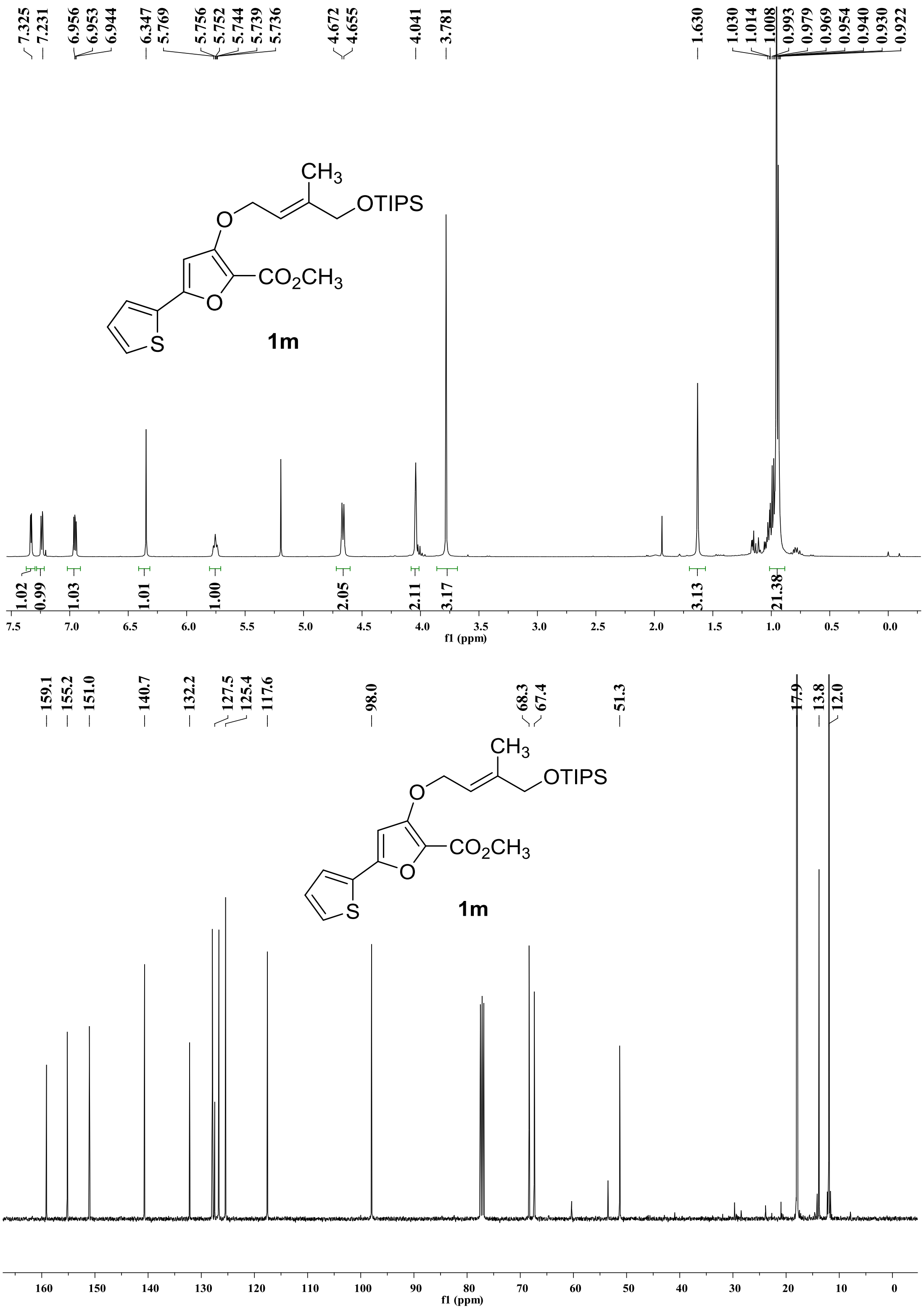

Supplementary Figure 14. ^{1}H and ^{13}C spectra for substrate **1l**

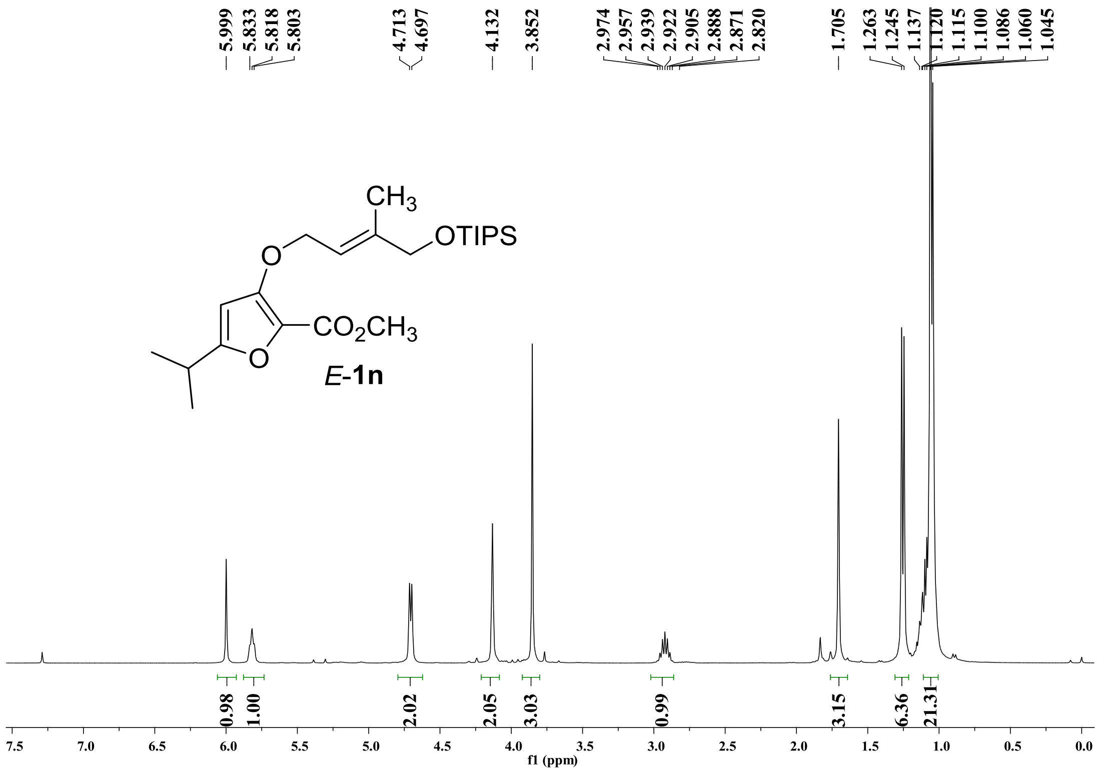

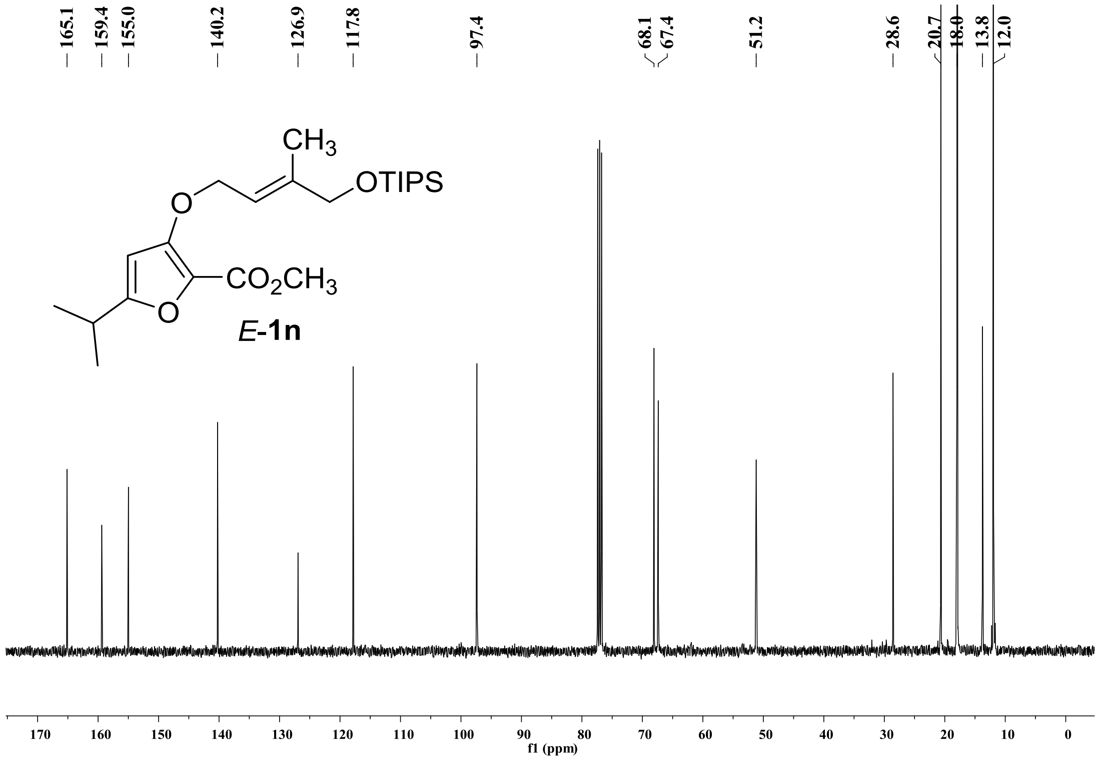

Supplementary Figure 15. ^{1}H and ^{13}C spectra for substrate *E*-**1n**

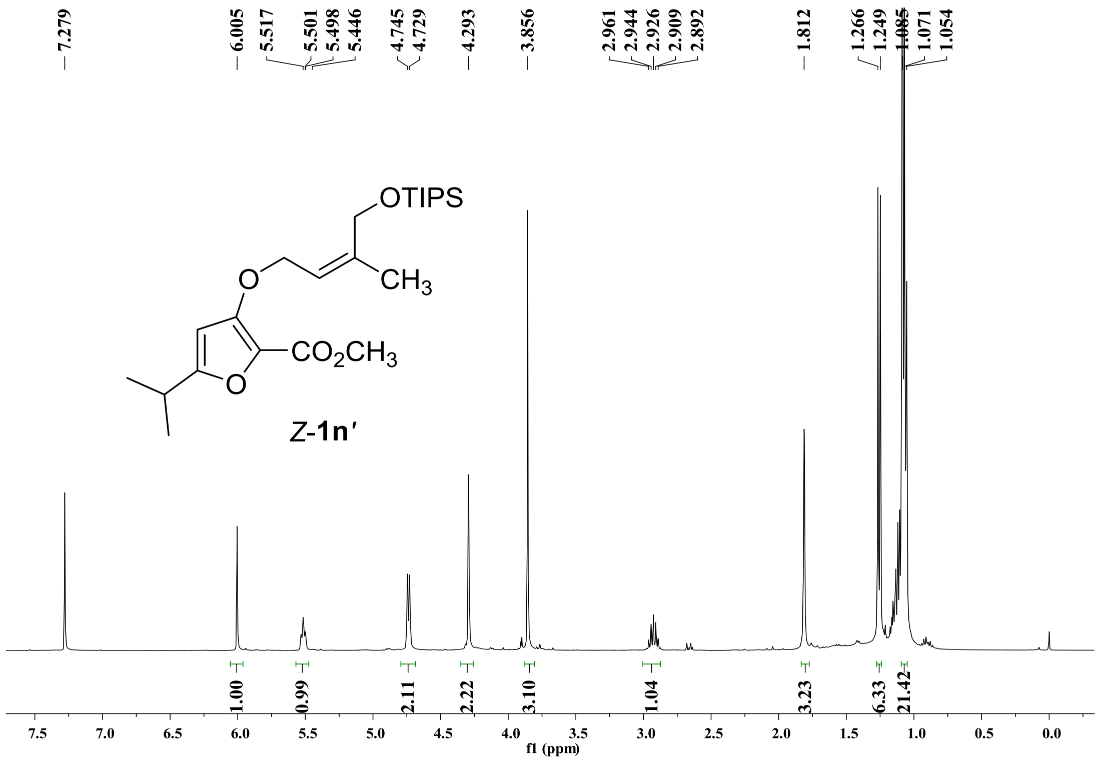

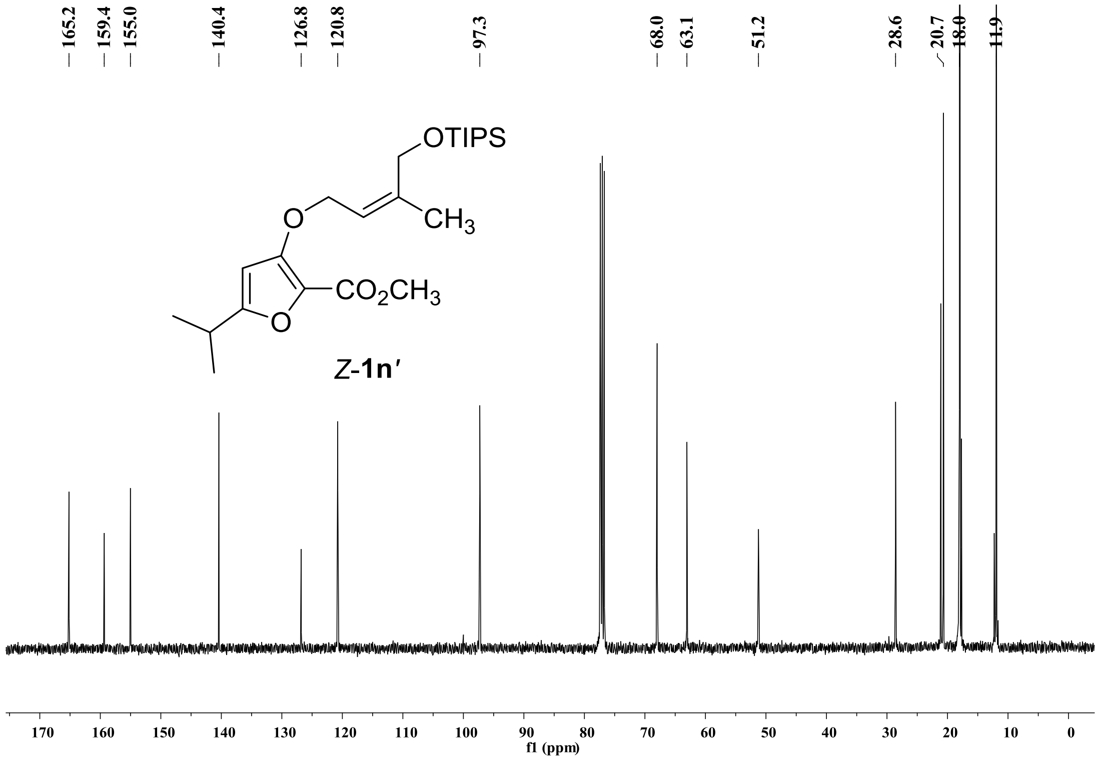

Supplementary Figure 16. ^{1}H and ^{13}C spectra for substrate *Z*-**1n'**

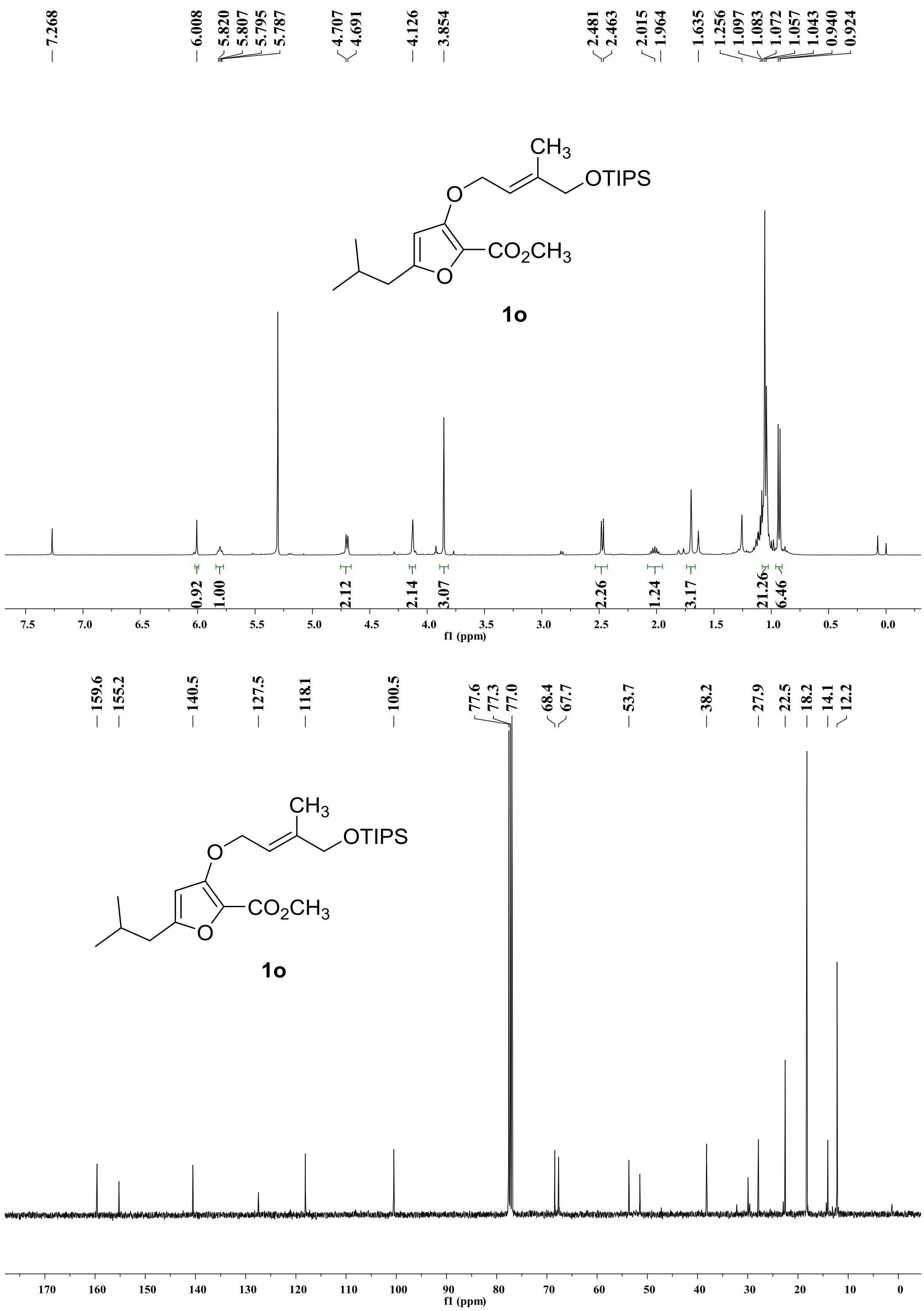

Supplementary Figure 17. ^{1}H and ^{13}C spectra for substrate **1o**

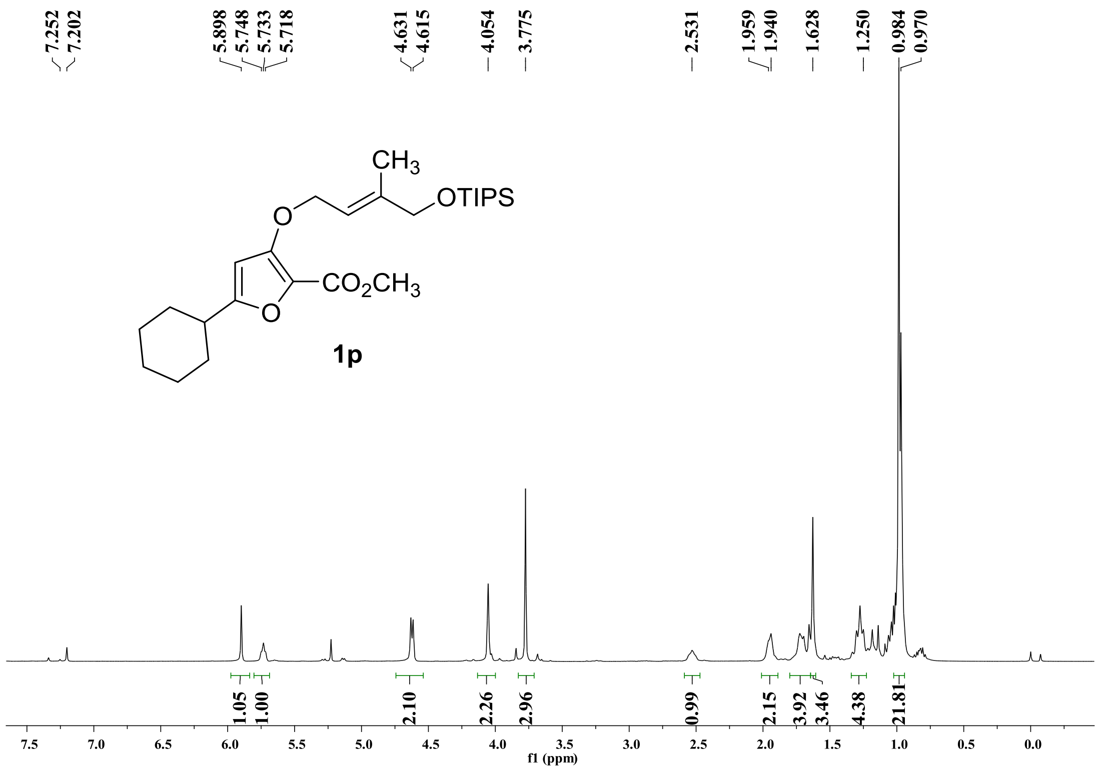

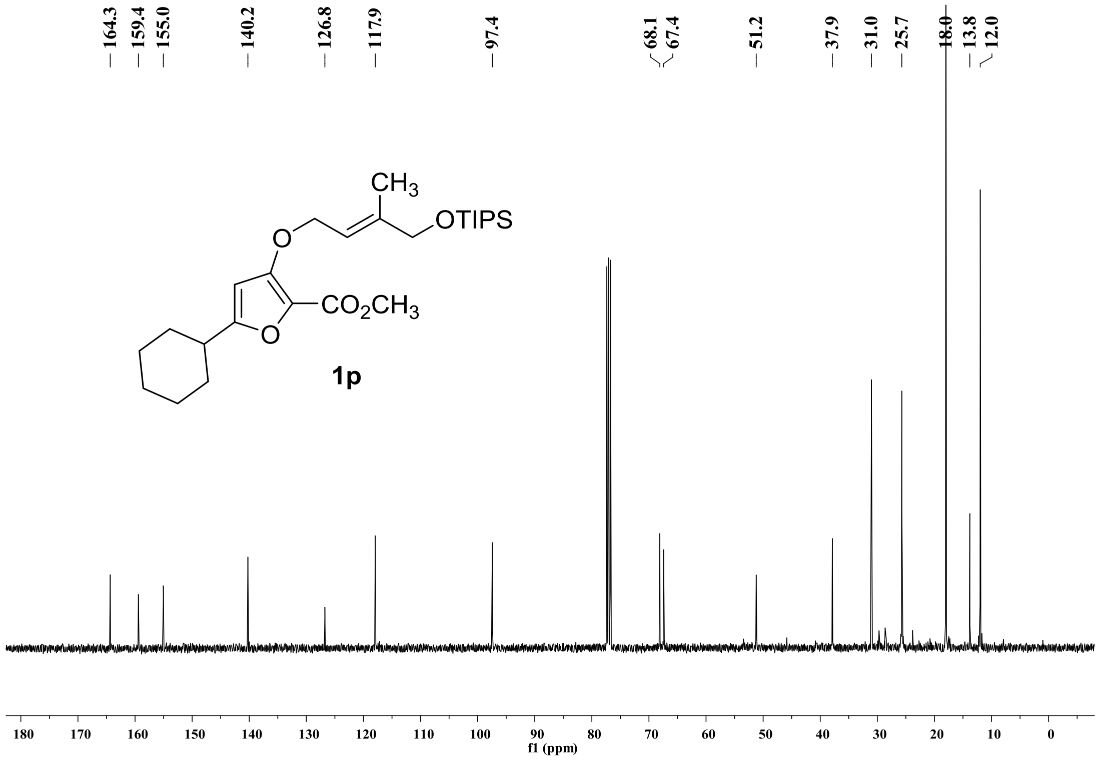

Supplementary Figure 18. ^{1}H and ^{13}C spectra for substrate **1p**

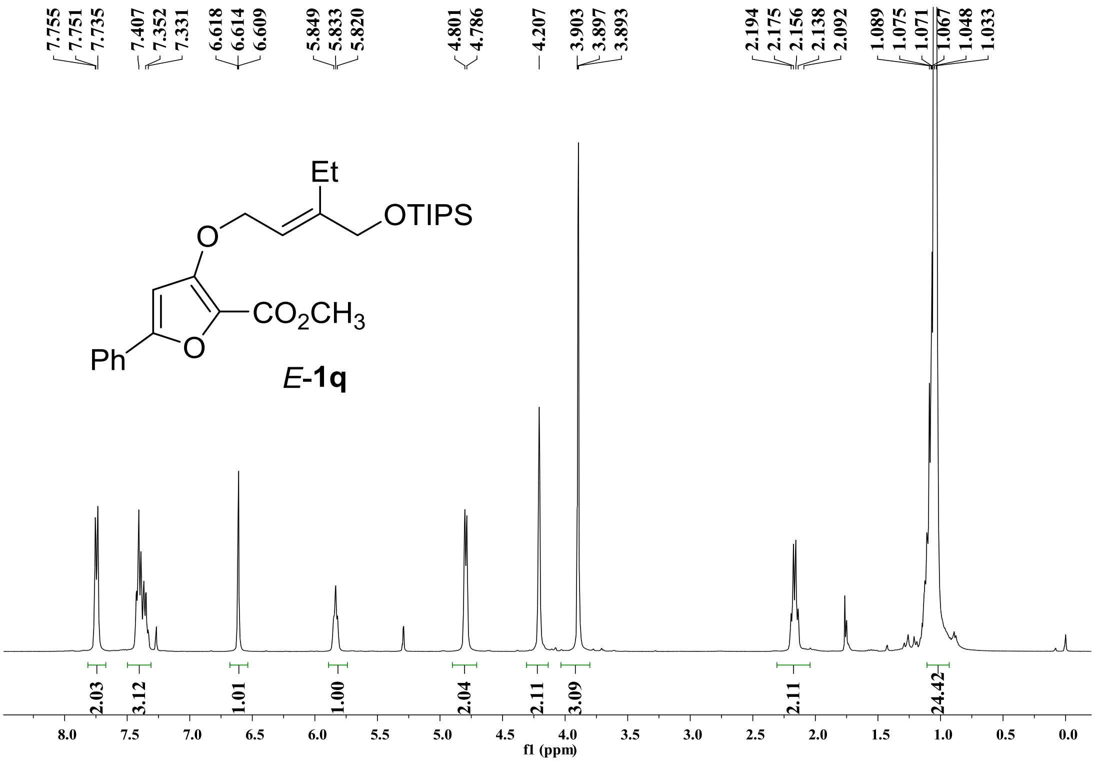

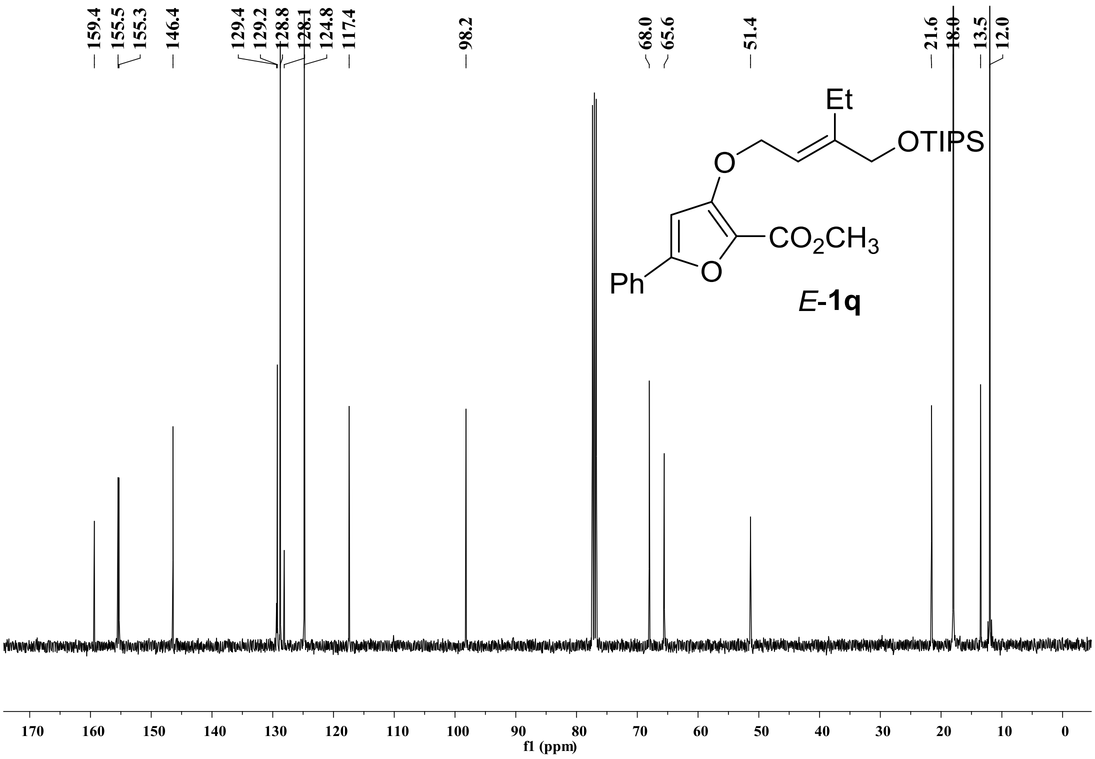

Supplementary Figure 19. ^{1}H and ^{13}C spectra for substrate *E*-**1q**

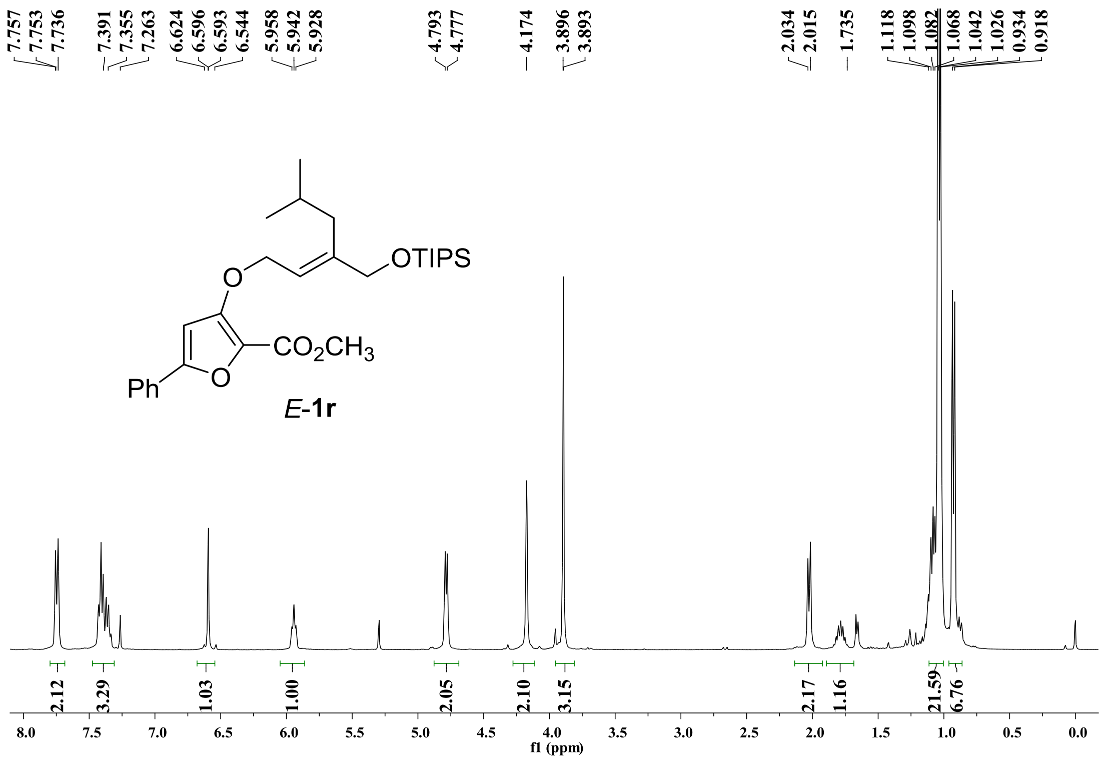

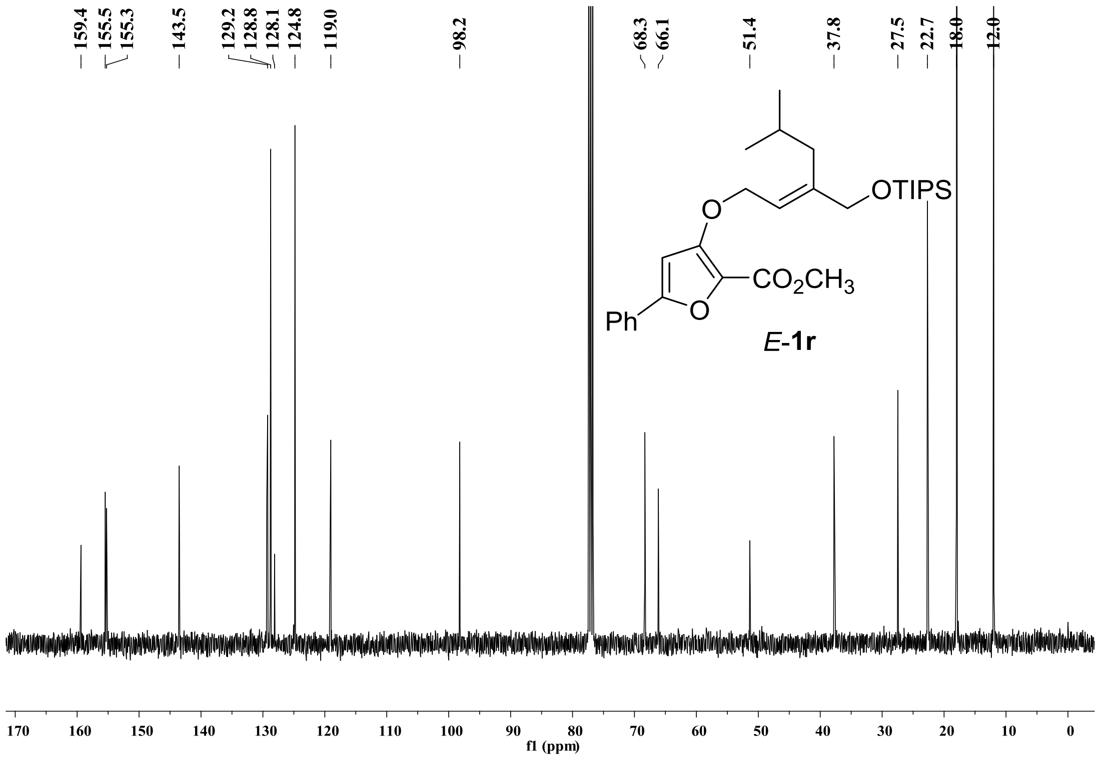

Supplementary Figure 20. ^{1}H and ^{13}C spectra for substrate *E*-**1r**

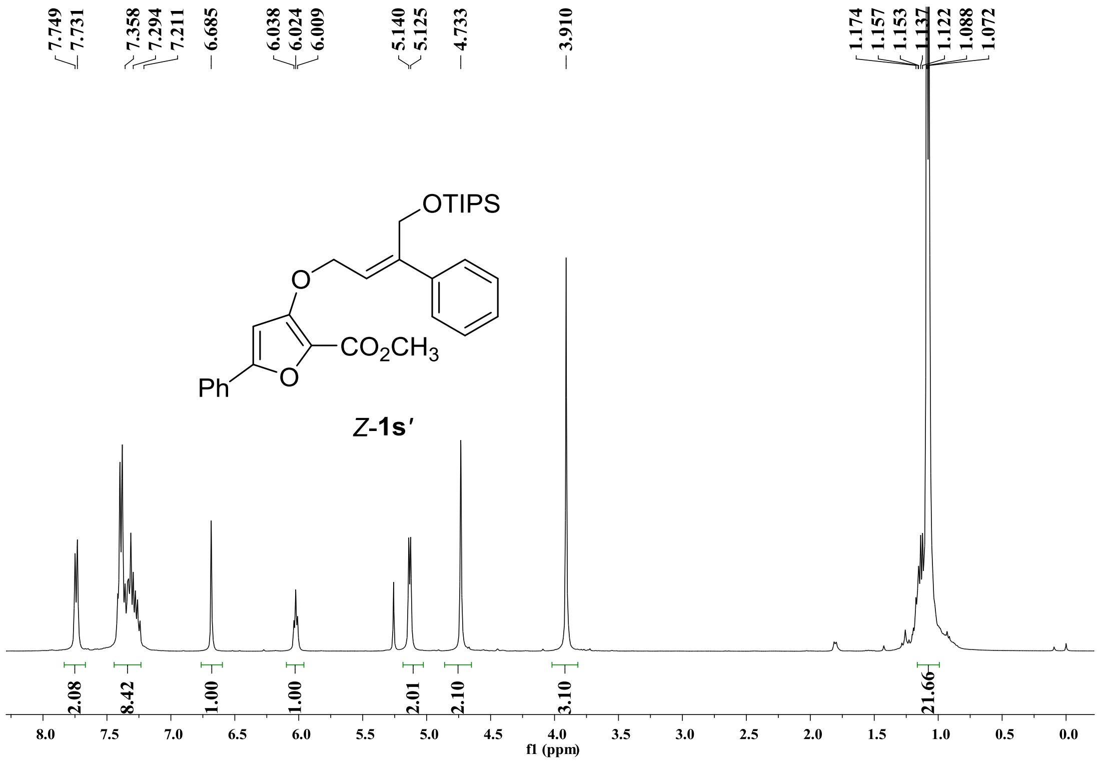

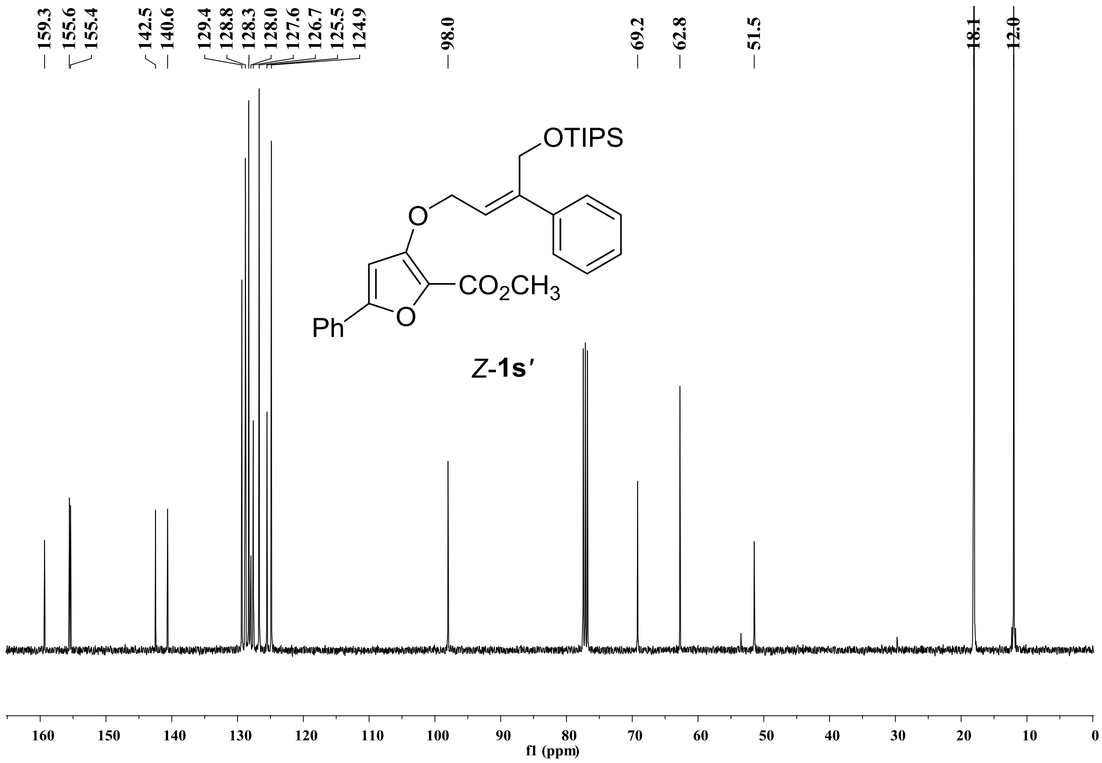

Supplementary Figure 21. ^{1}H and ^{13}C spectra for substrate *Z*-**1s'**

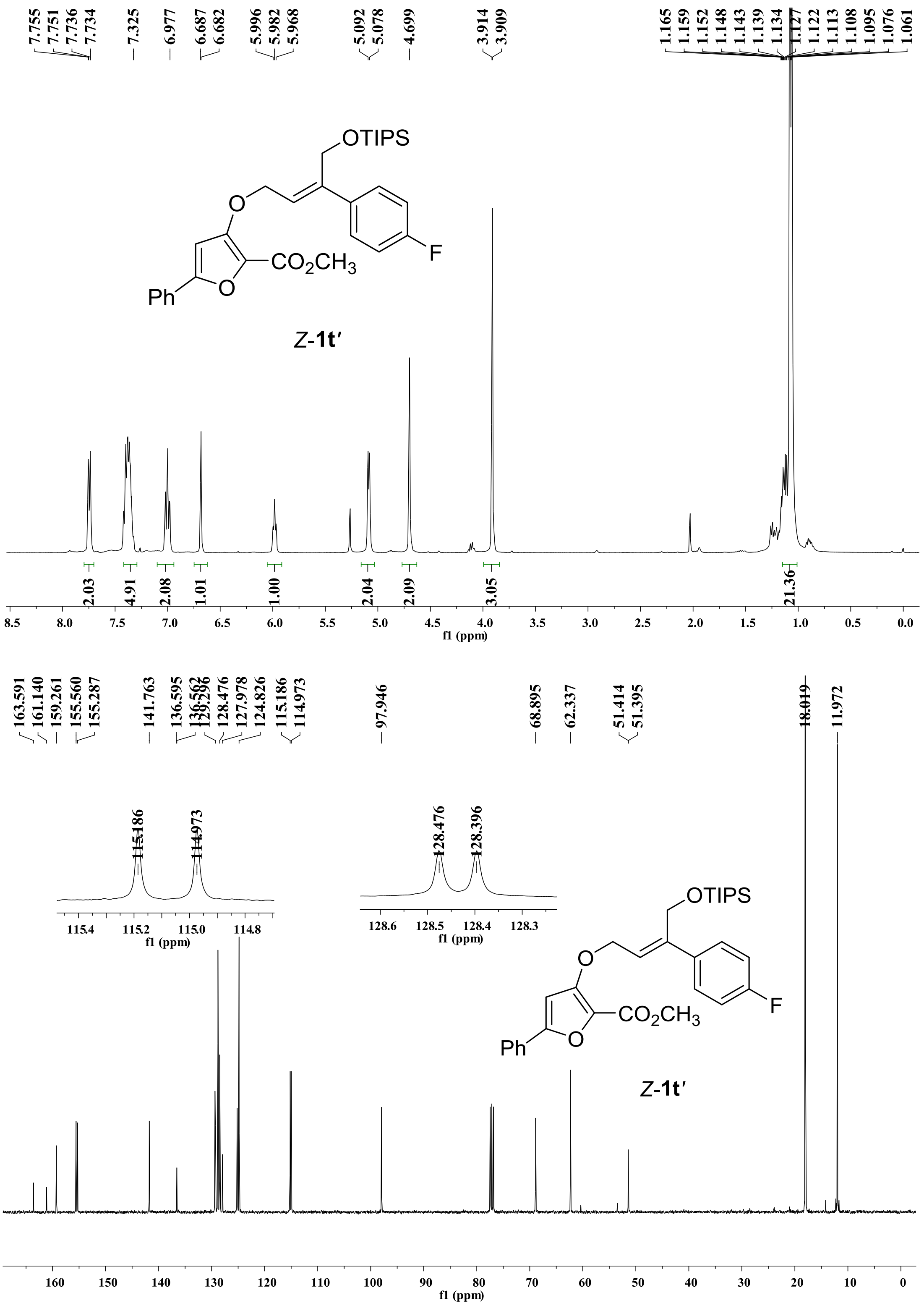

Supplementary Figure 22. ^{1}H and ^{13}C spectra for substrate *Z*-**1t'**

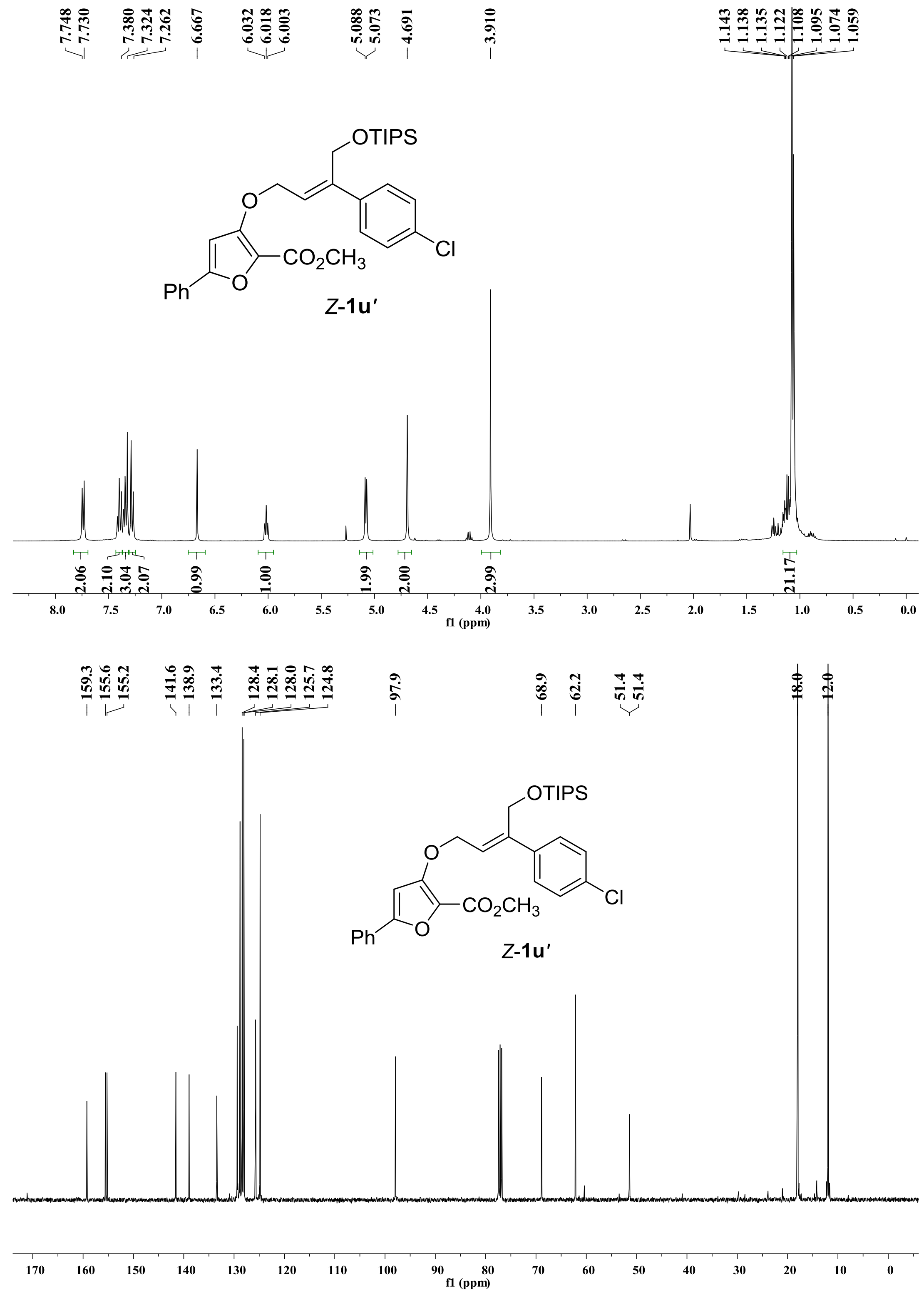

Supplementary Figure 23. ^{1}H and ^{13}C spectra for substrate *Z*-**1u'**

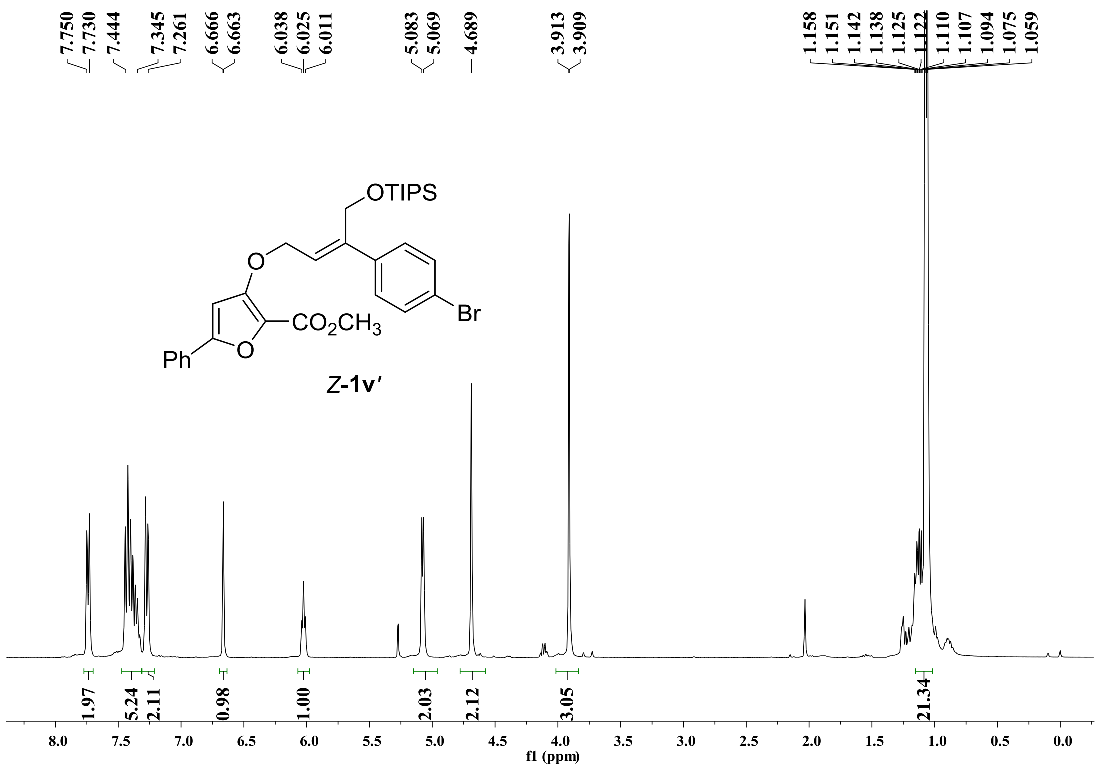

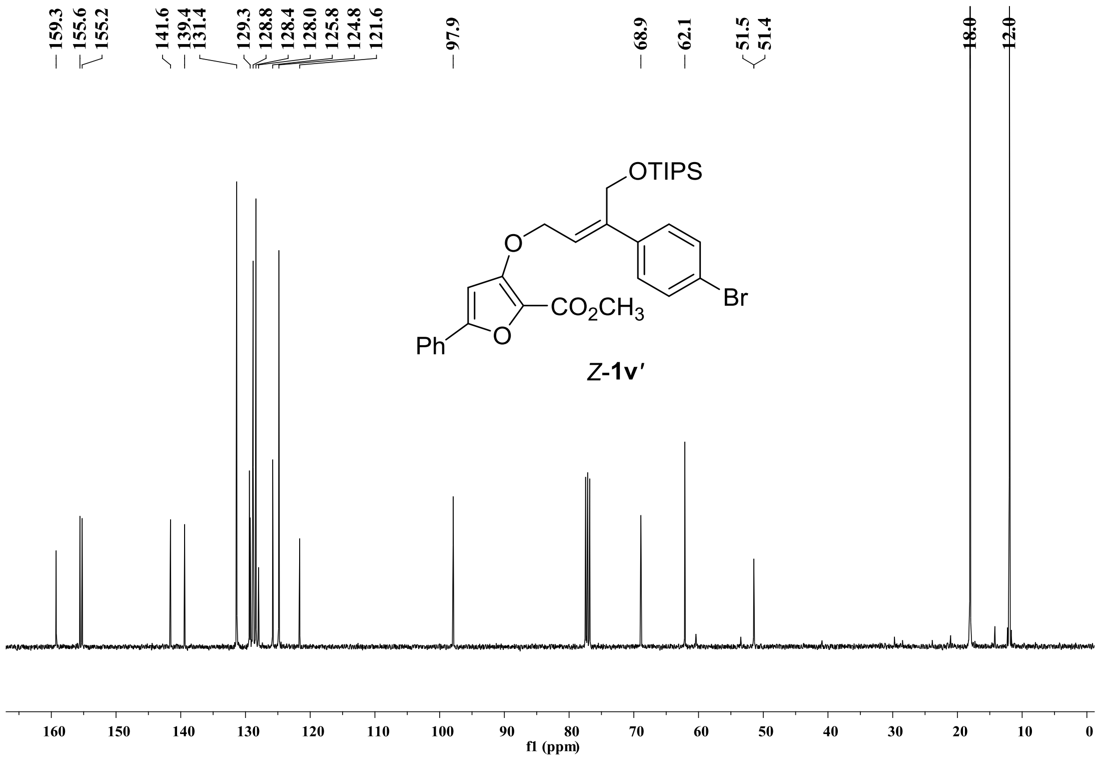

Supplementary Figure 24. ^{1}H and ^{13}C spectra for substrate *Z*-**1v'**

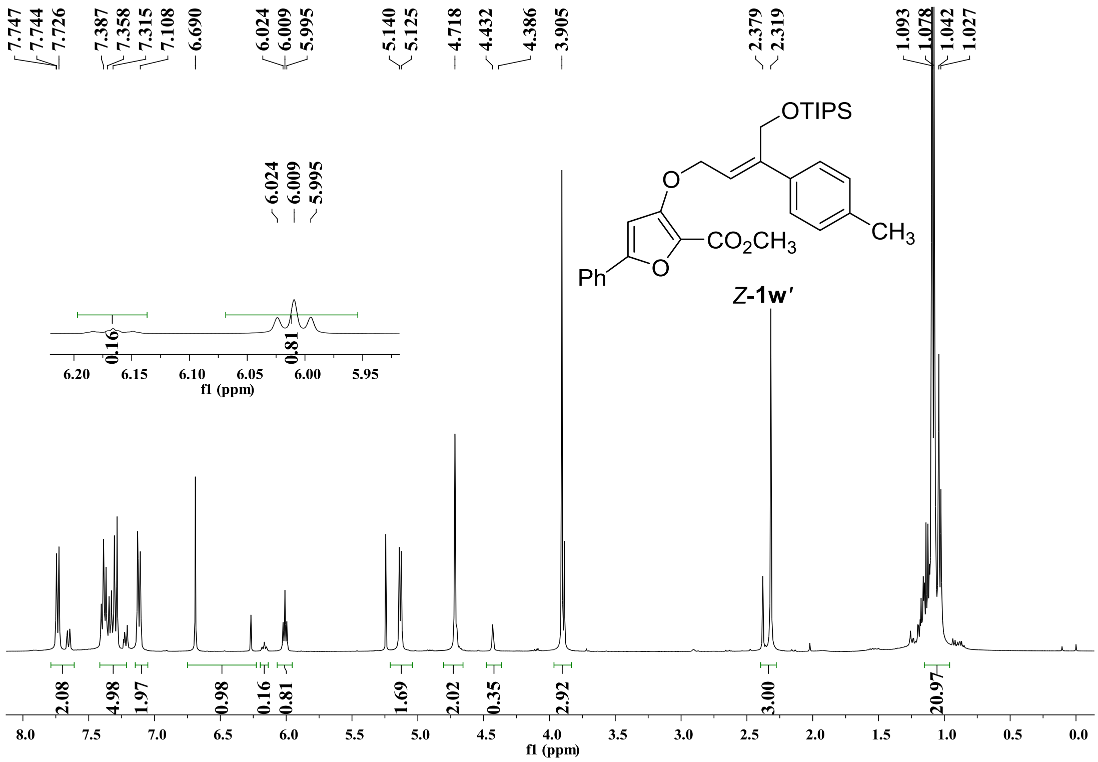

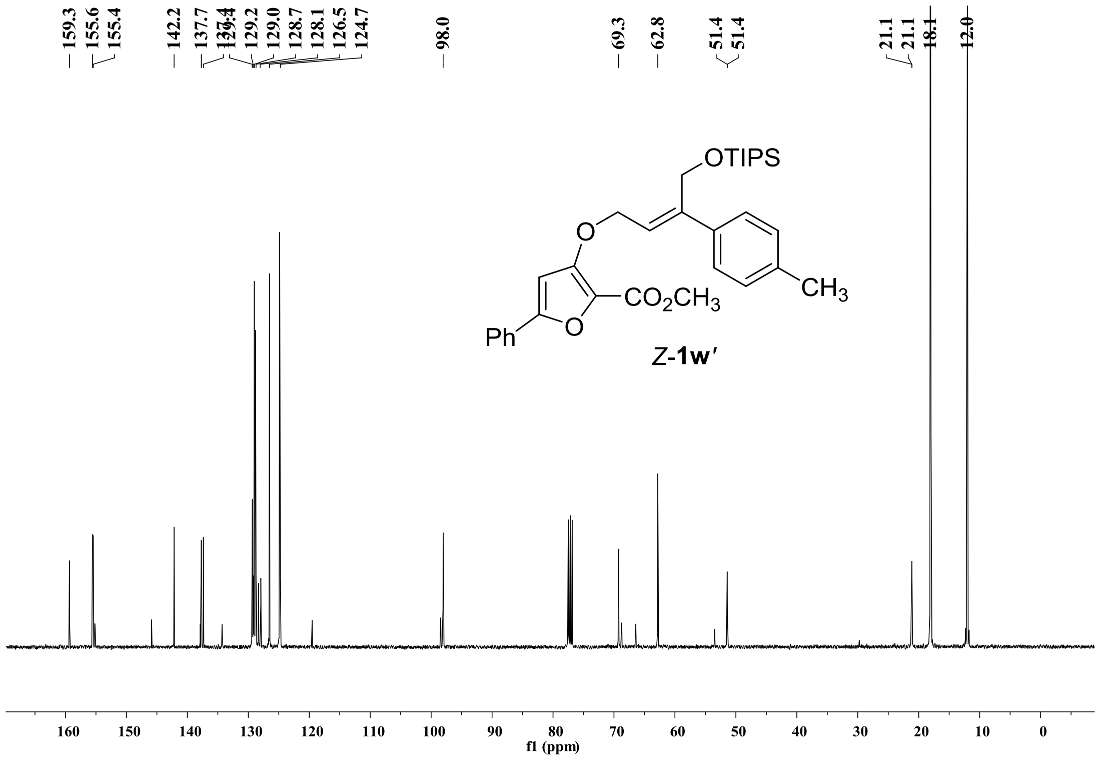

Supplementary Figure 25. ^{1}H and ^{13}C spectra for substrate *Z*-**1w'**

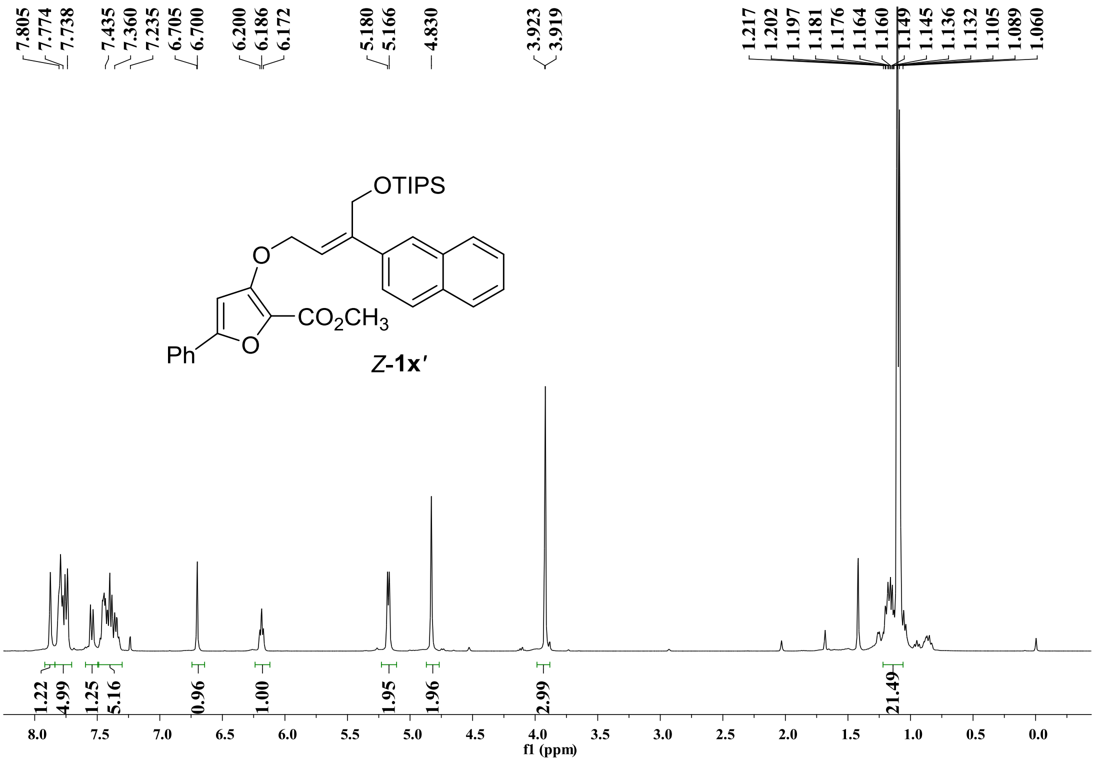

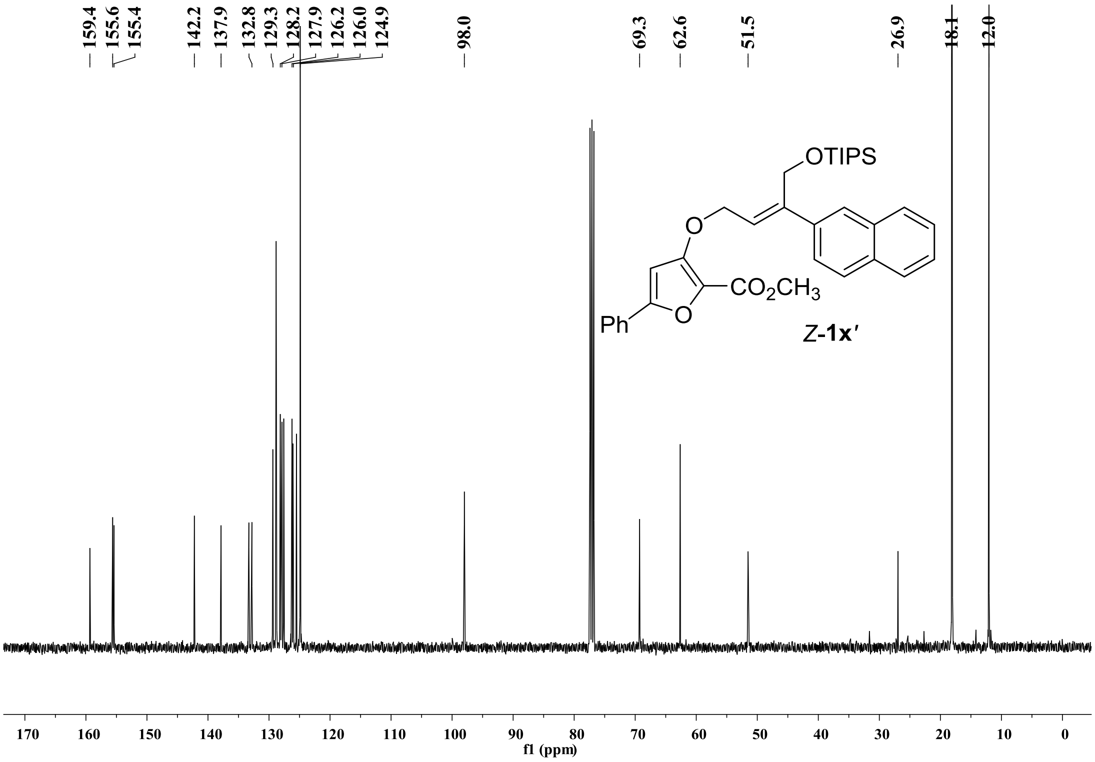

Supplementary Figure 26. ^{1}H and ^{13}C spectra for substrate *Z*-**1x'**

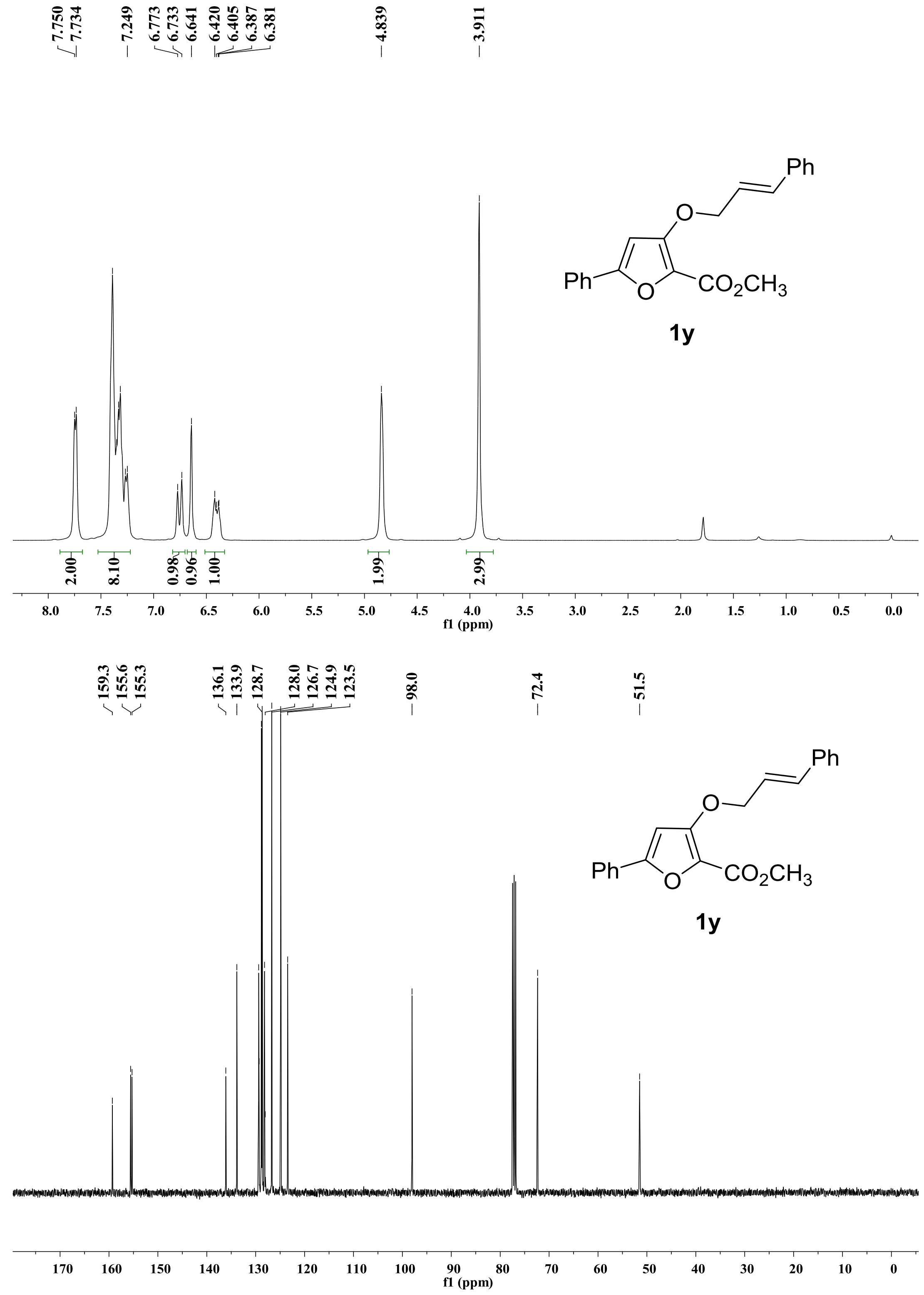

Supplementary Figure 27. ^{1}H and ^{13}C spectra for substrate **1y**

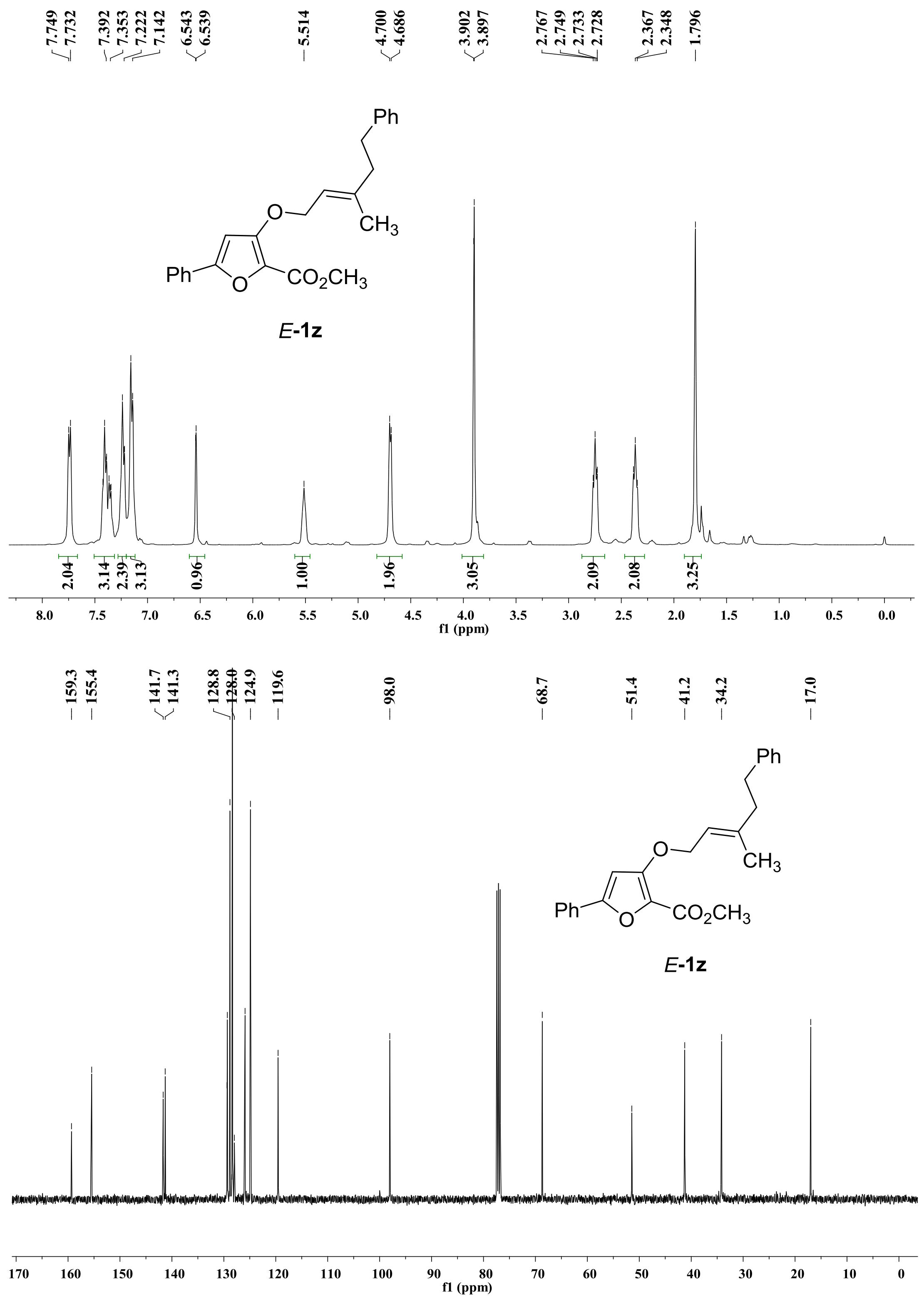

Supplementary Figure 28. ^{1}H and ^{13}C spectra for substrate *E*-**1z**

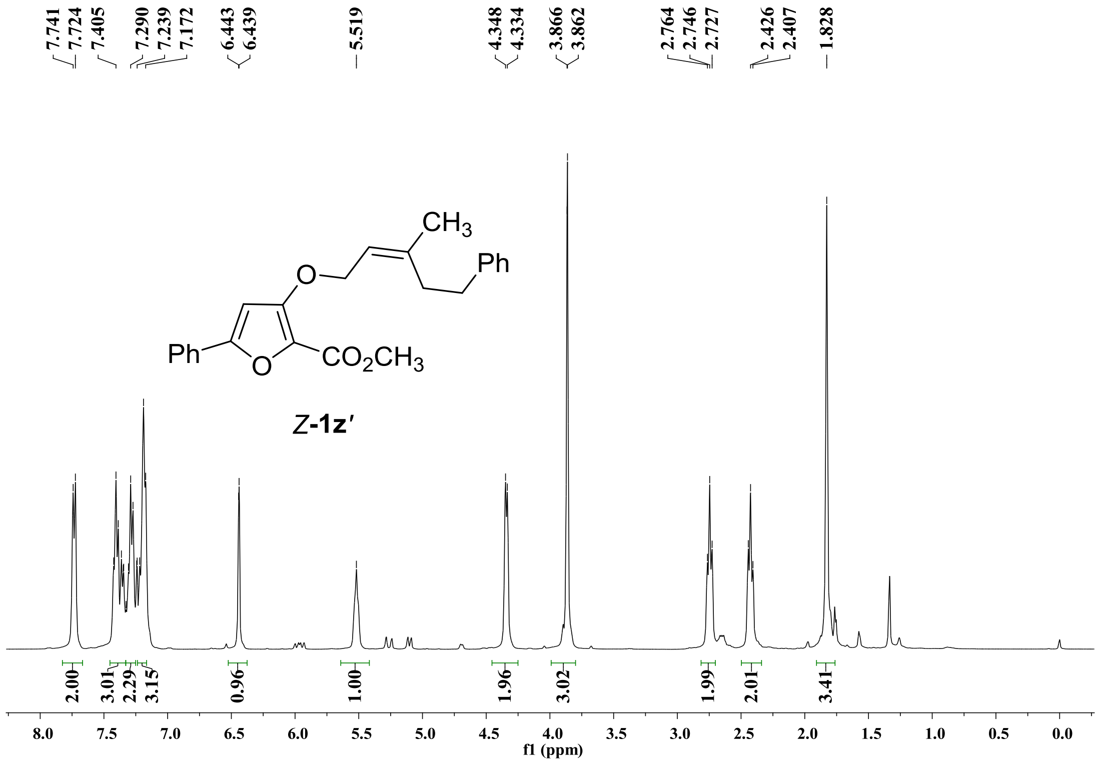

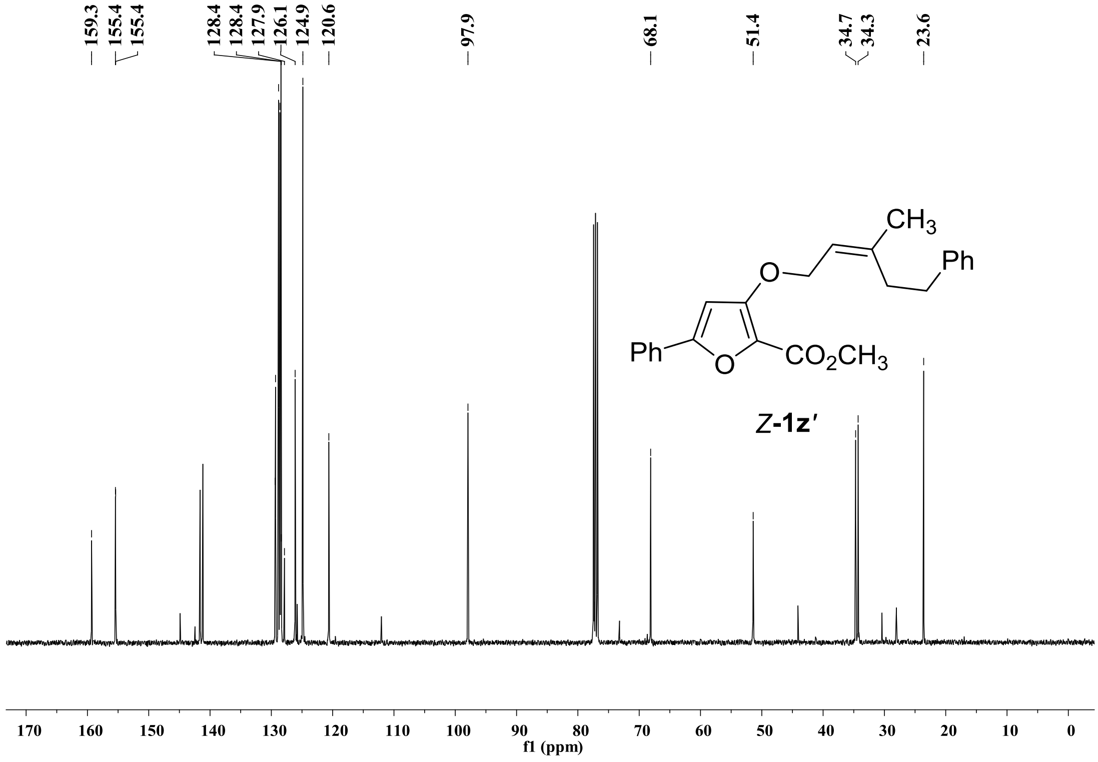

Supplementary Figure 29. ^{1}H and ^{13}C spectra for substrate *Z*-**1z'**

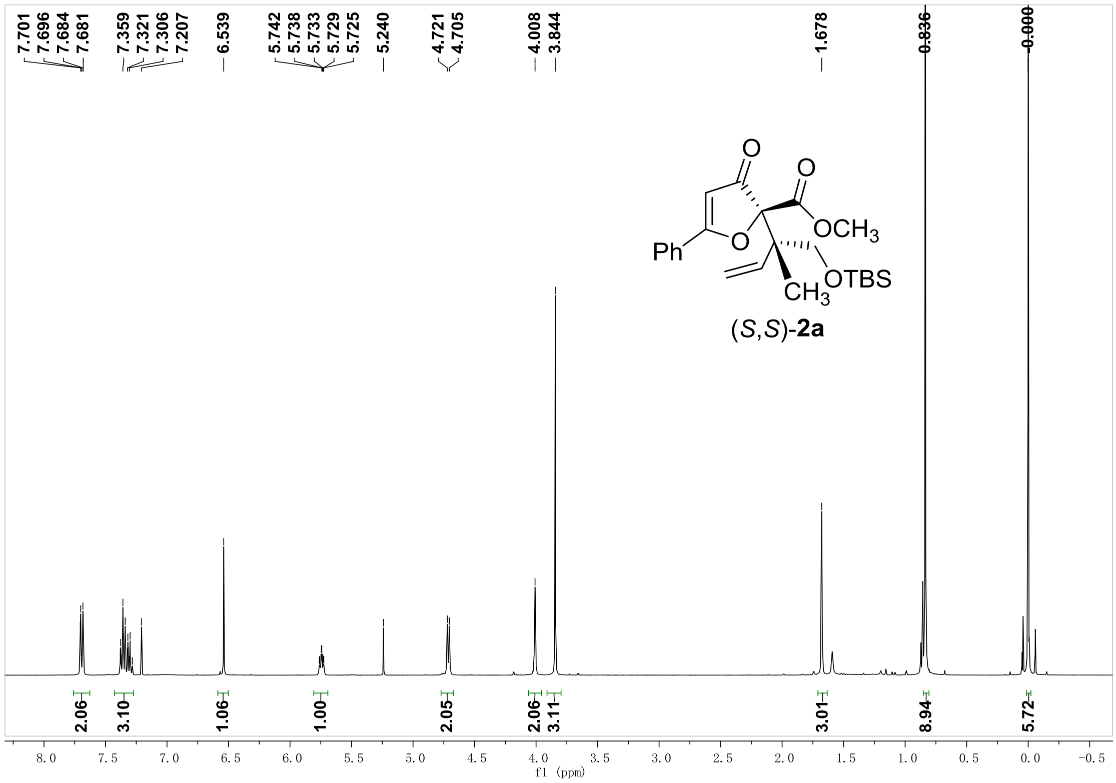

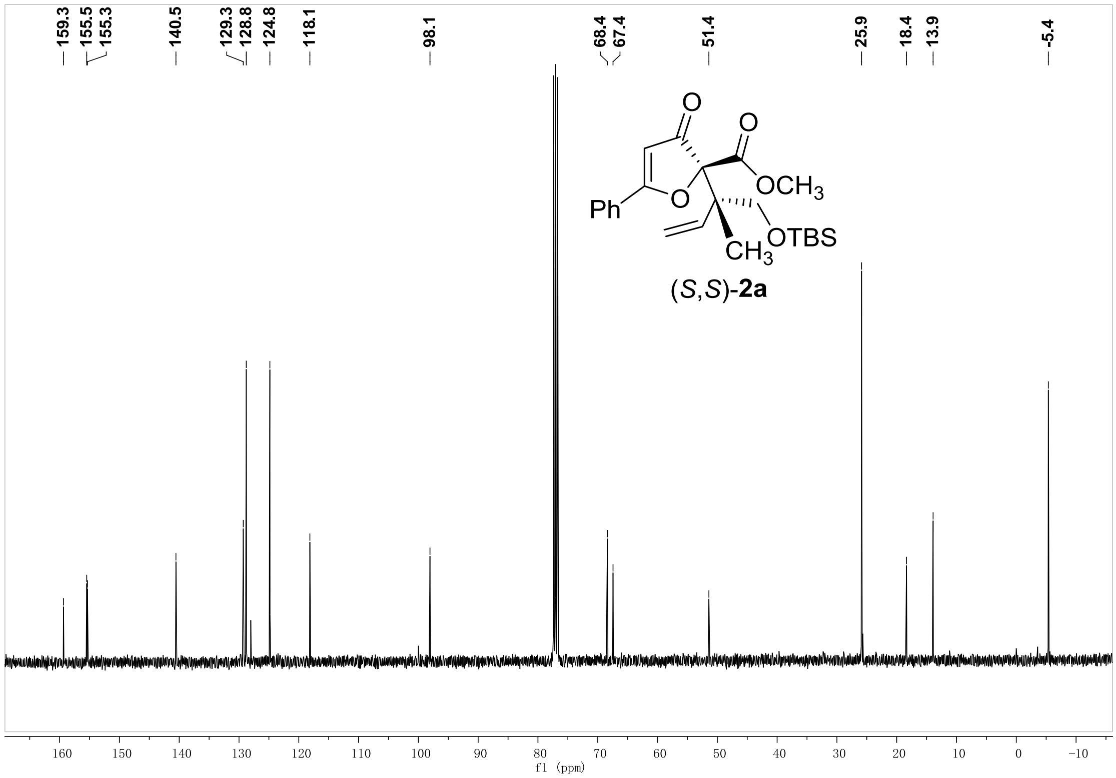

Supplementary Figure 30. ^{1}H and ^{13}C spectra for product (*S*,*S*)-**2a**

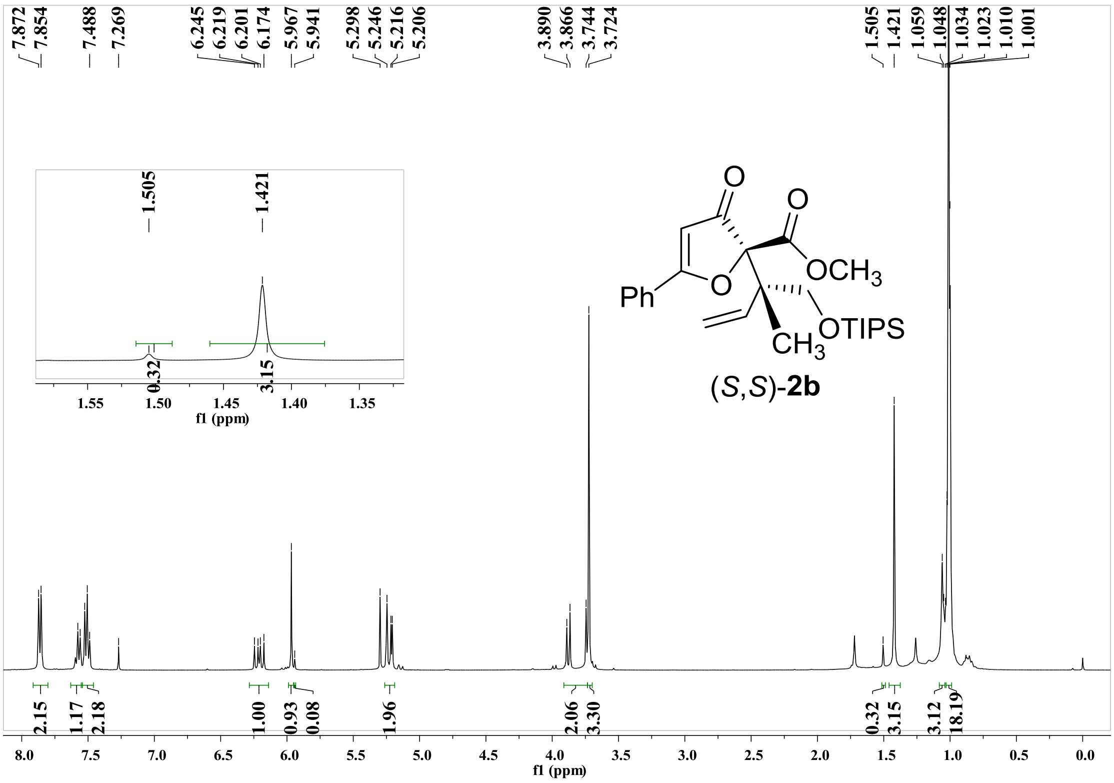

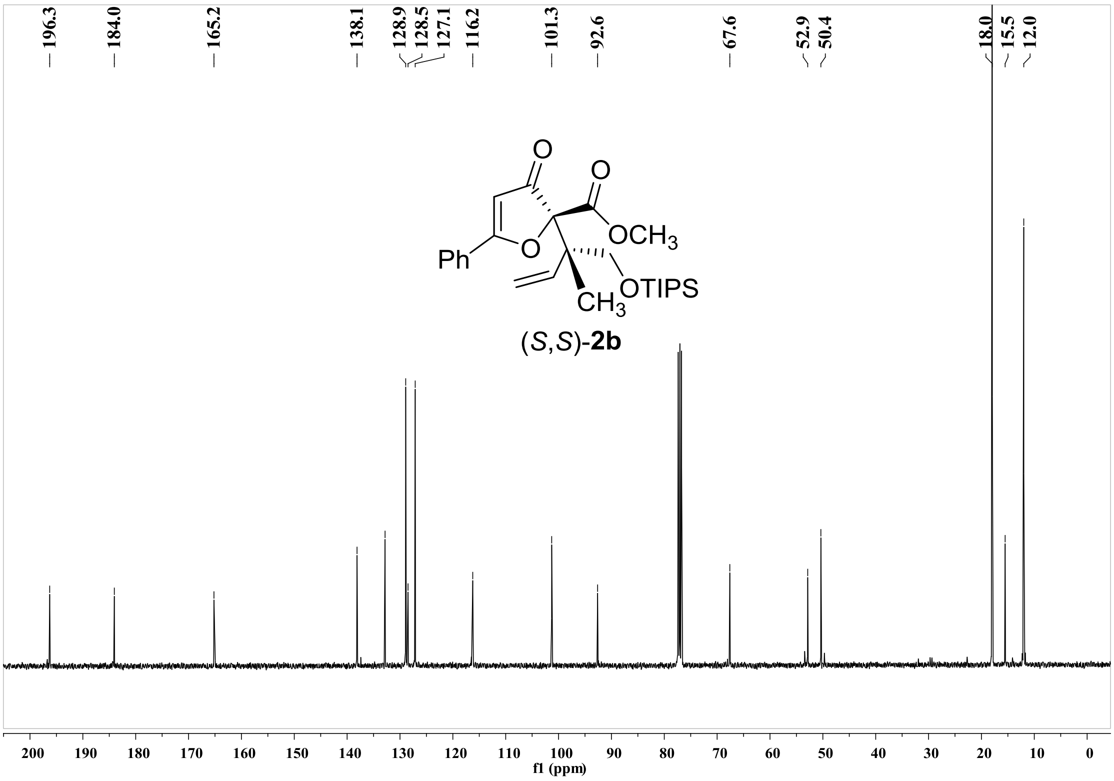

Supplementary Figure 31. ^{1}H and ^{13}C spectra for product (*S*,*S*)-**2b**

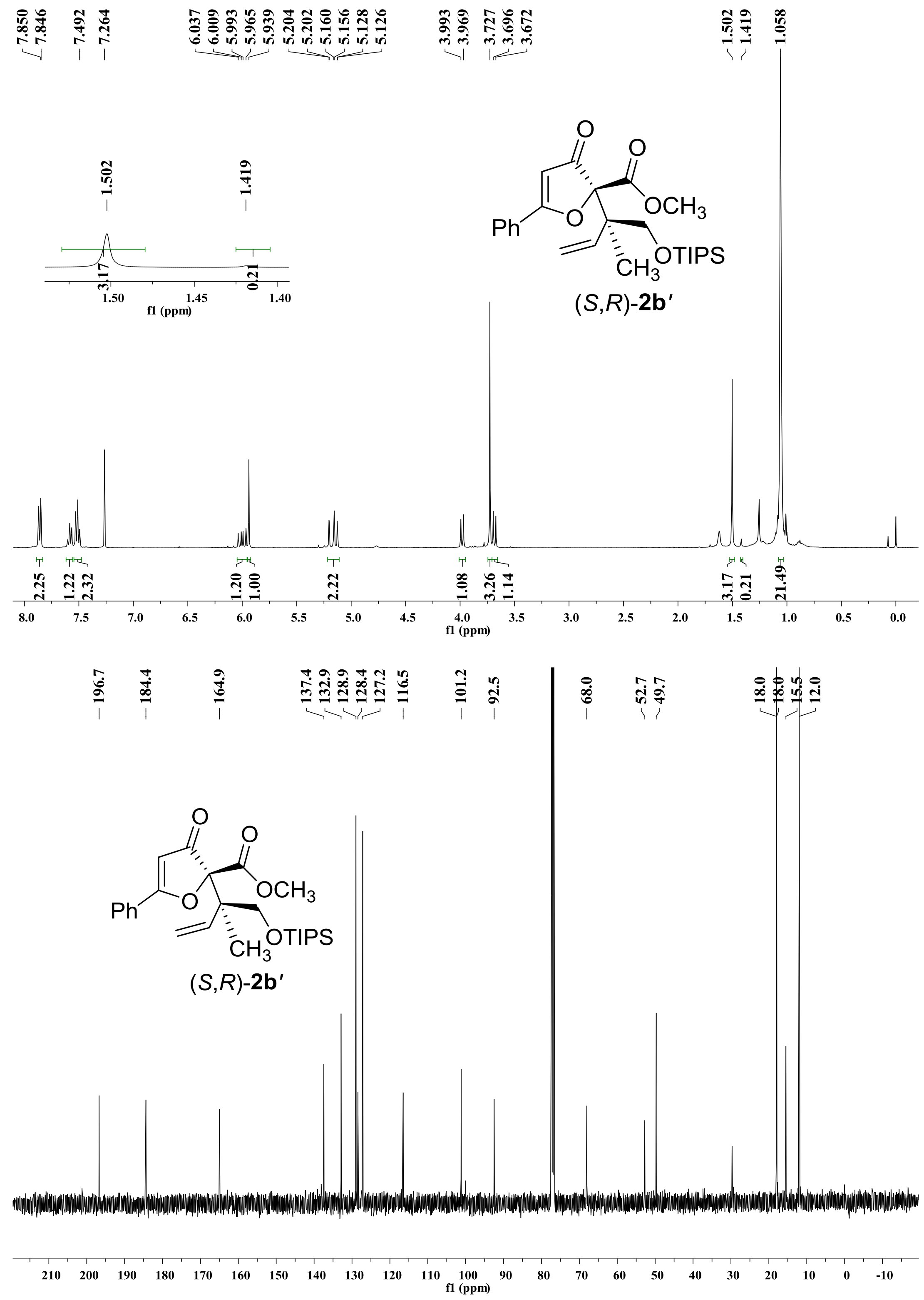

Supplementary Figure 32. ^{1}H and ^{13}C spectra for product (*S*,*R*)-**2b'**

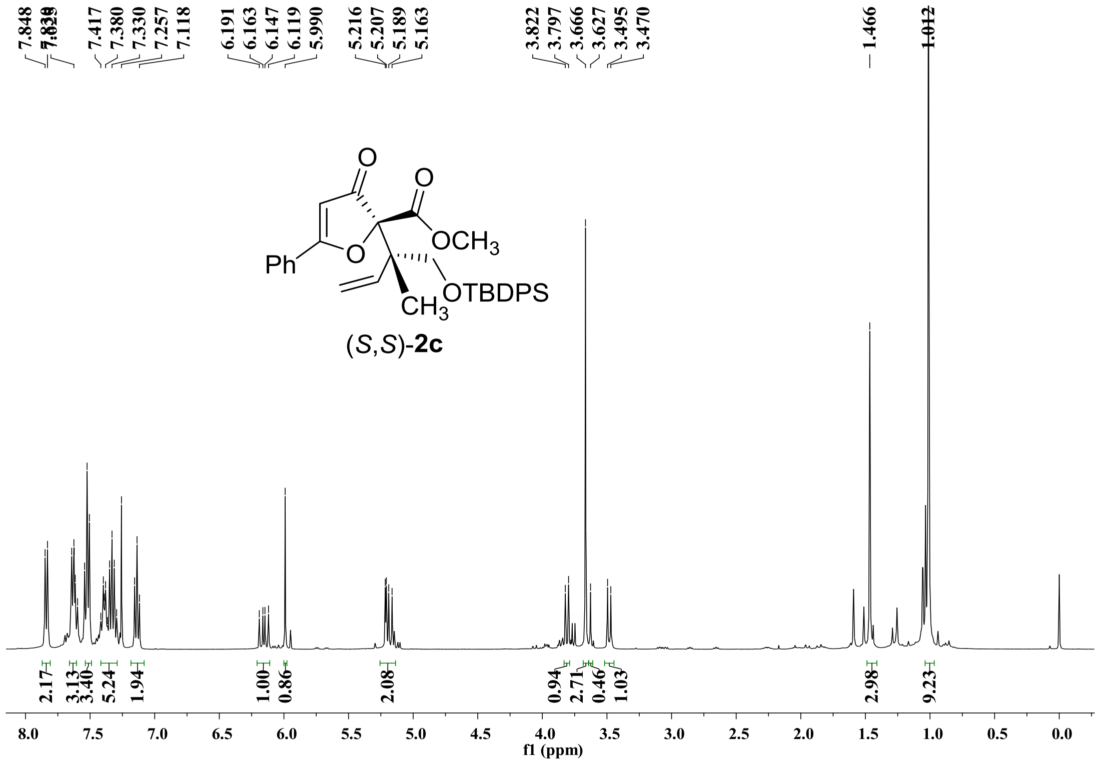

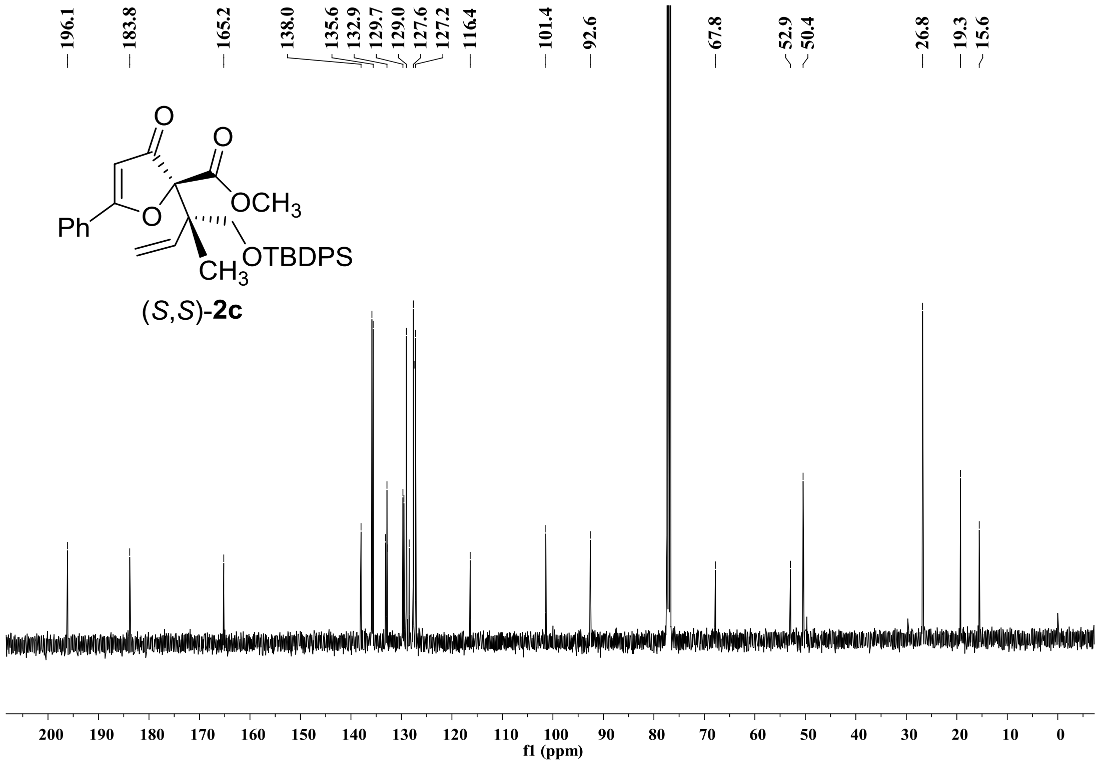

Supplementary Figure 33. ^{1}H and ^{13}C spectra for product (*S*,*S*)-**2c**

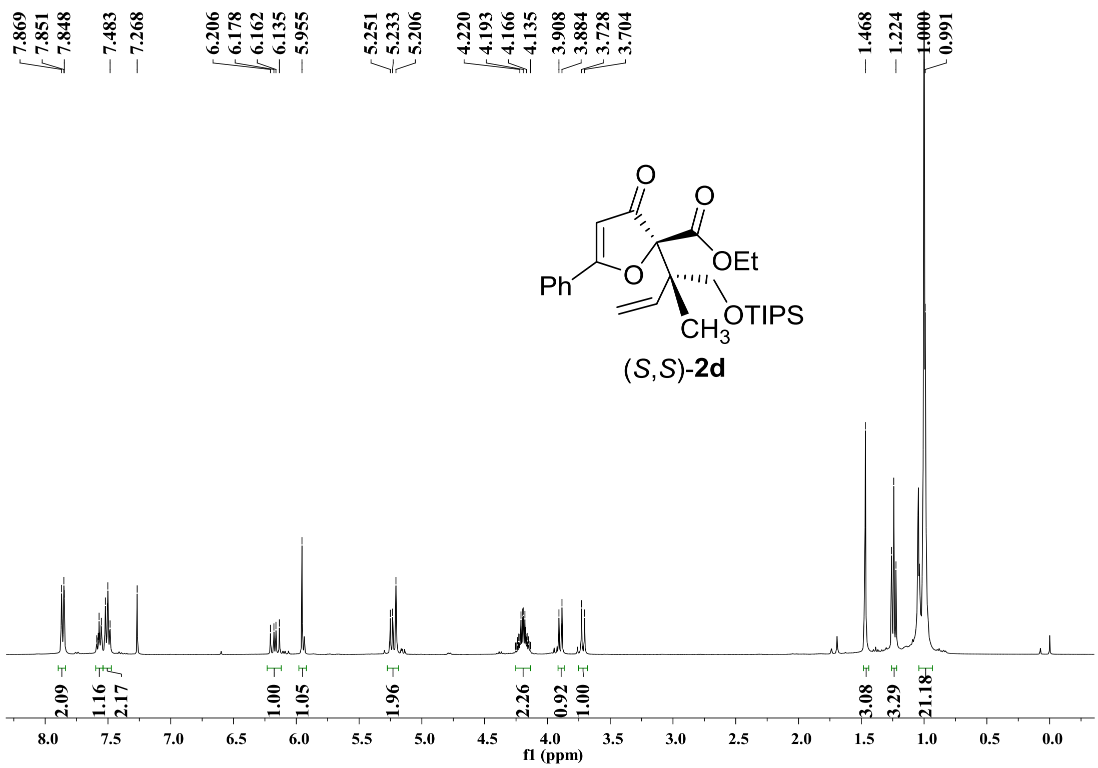

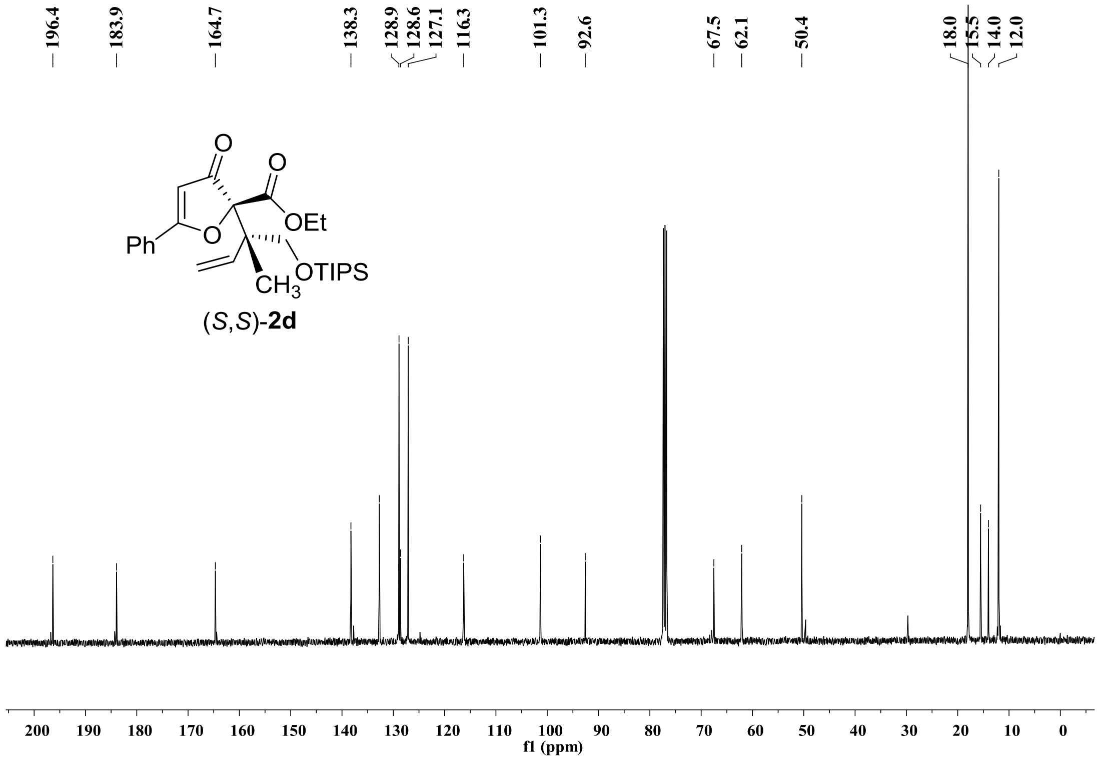

Supplementary Figure 34. ^{1}H and ^{13}C spectra for product (*S*,*S*)-**2d**

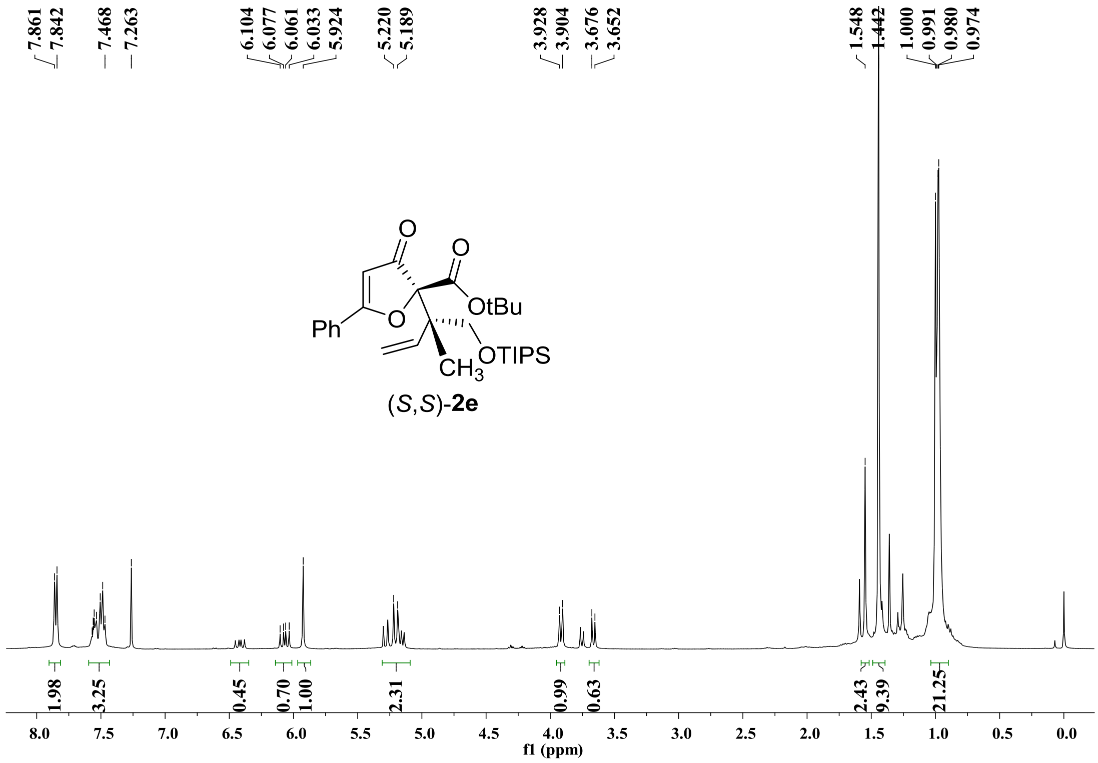

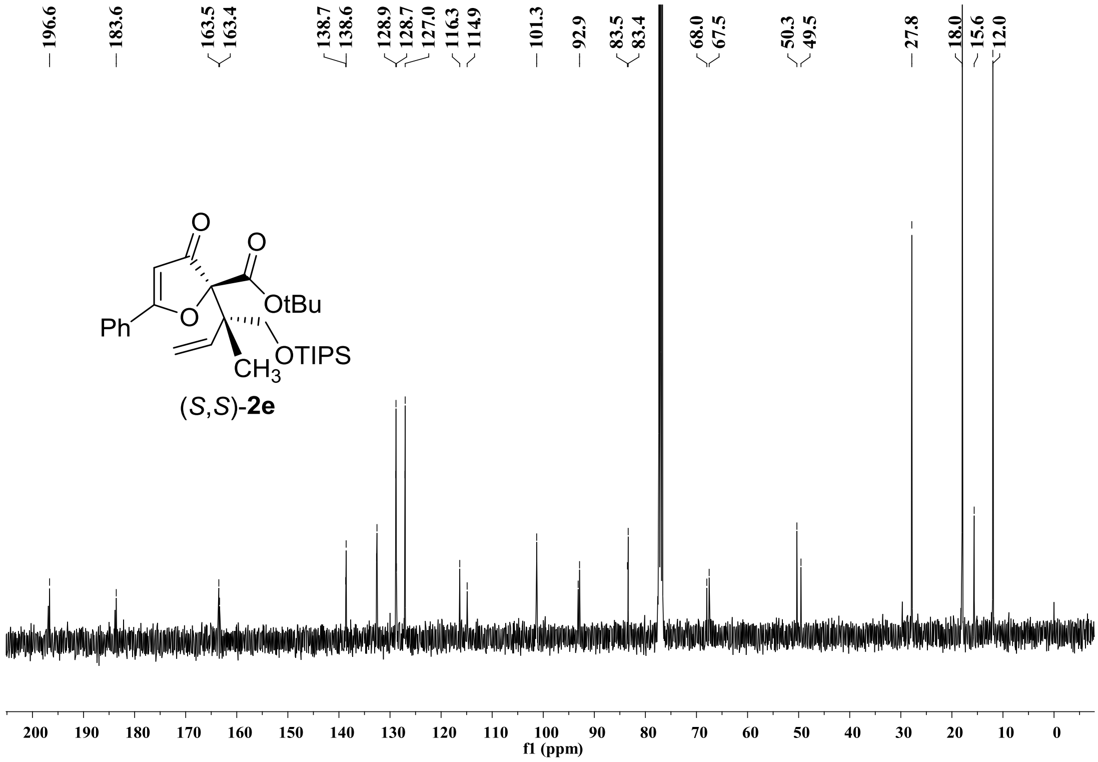

Supplementary Figure 35. ^{1}H and ^{13}C spectra for product (*S*,*S*)-**2e**

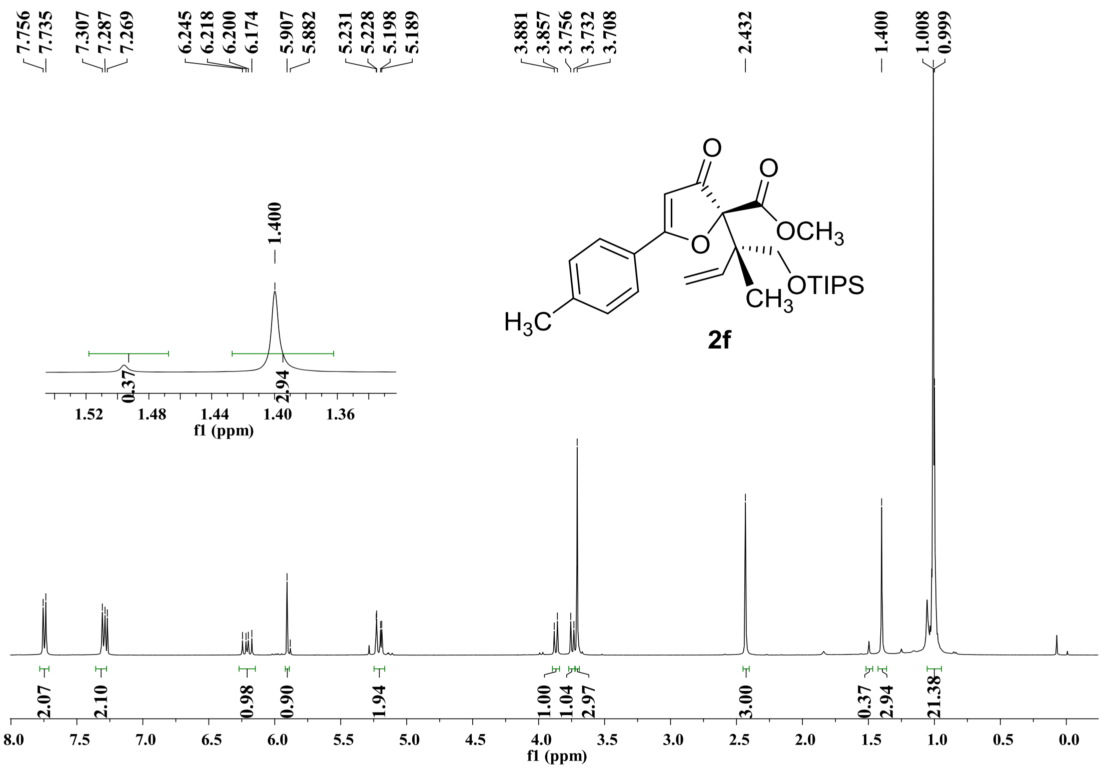

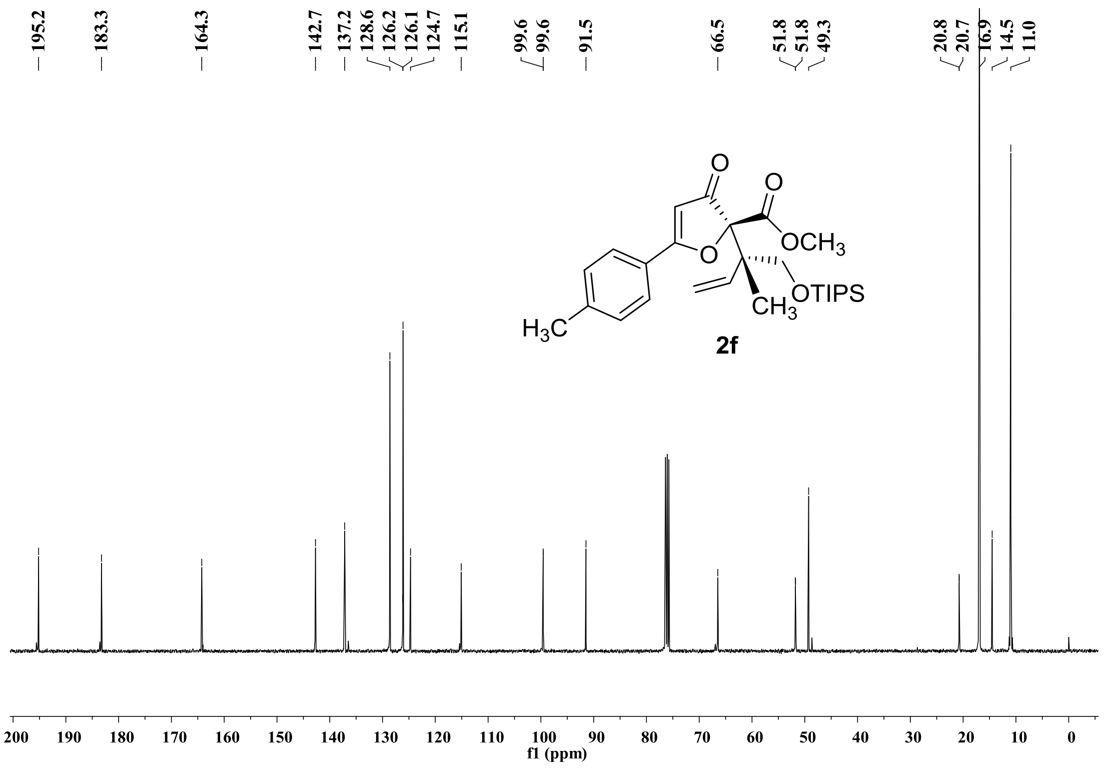

Supplementary Figure 36. ^{1}H and ^{13}C spectra for product **2f**

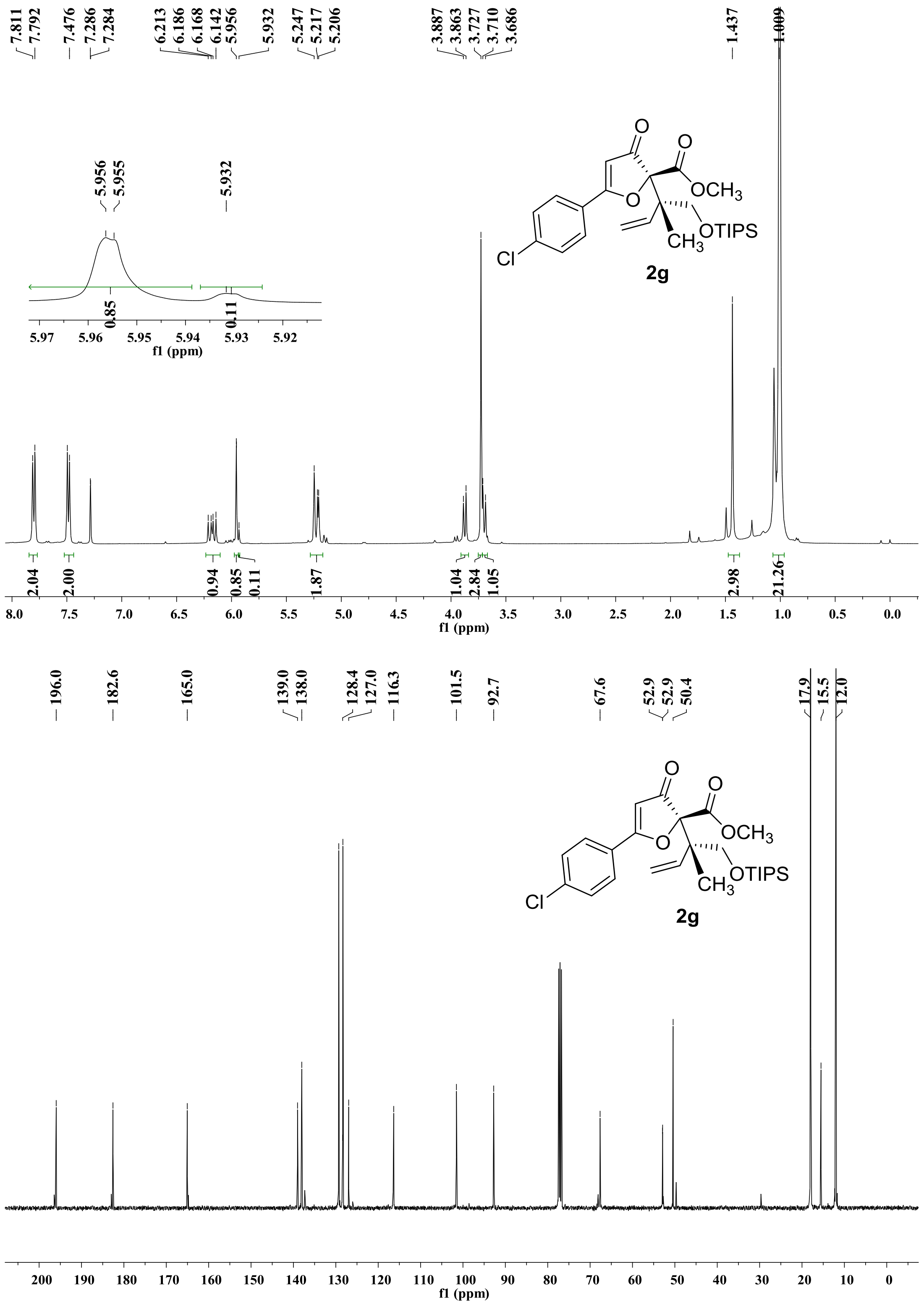

Supplementary Figure 37. ^{1}H and ^{13}C spectra for product **2g**

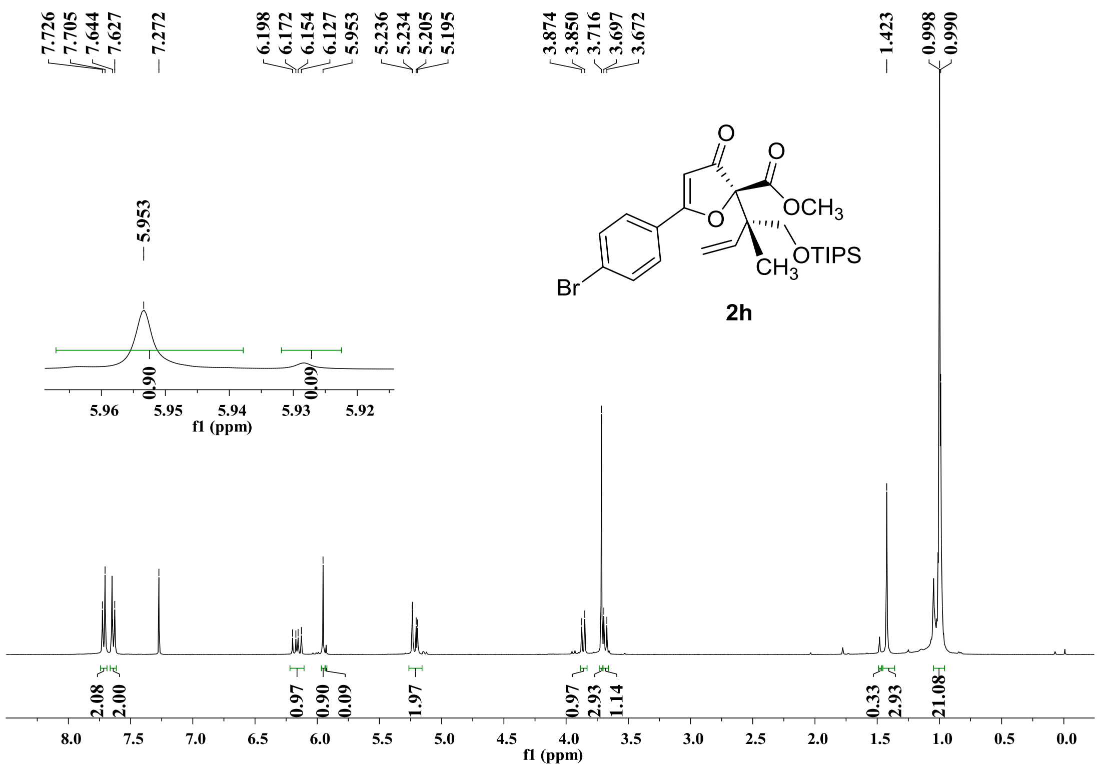

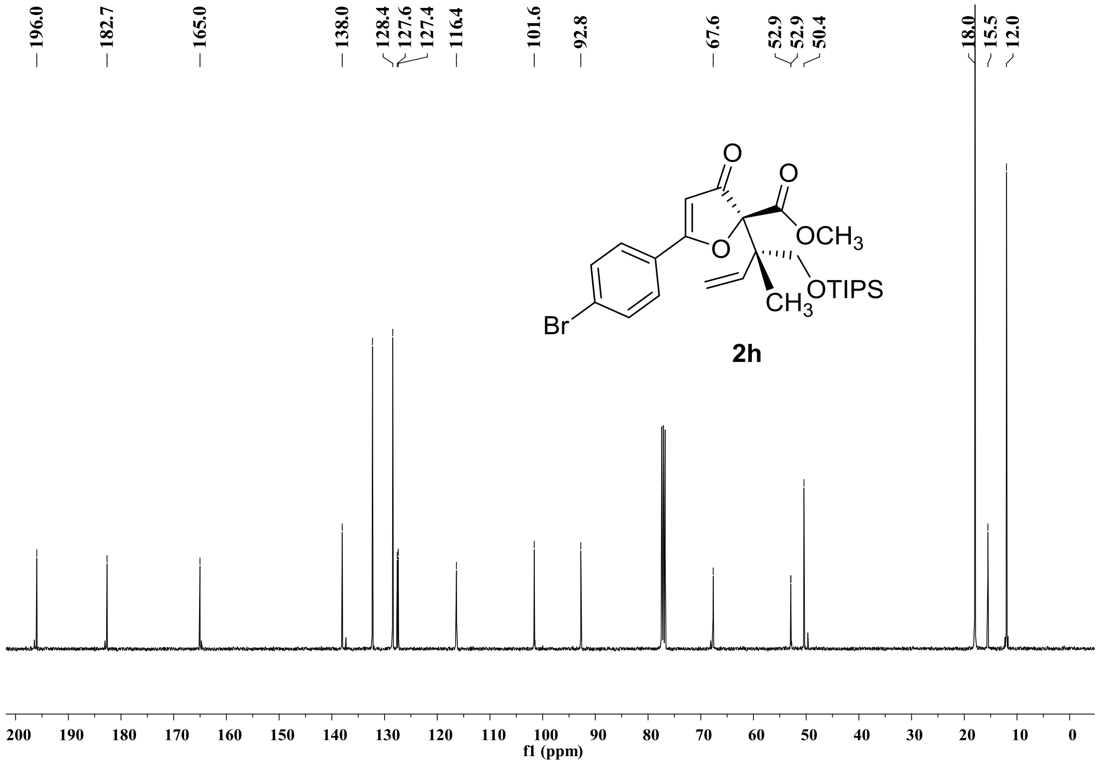

Supplementary Figure 38. ^{1}H and ^{13}C spectra for product **2h**

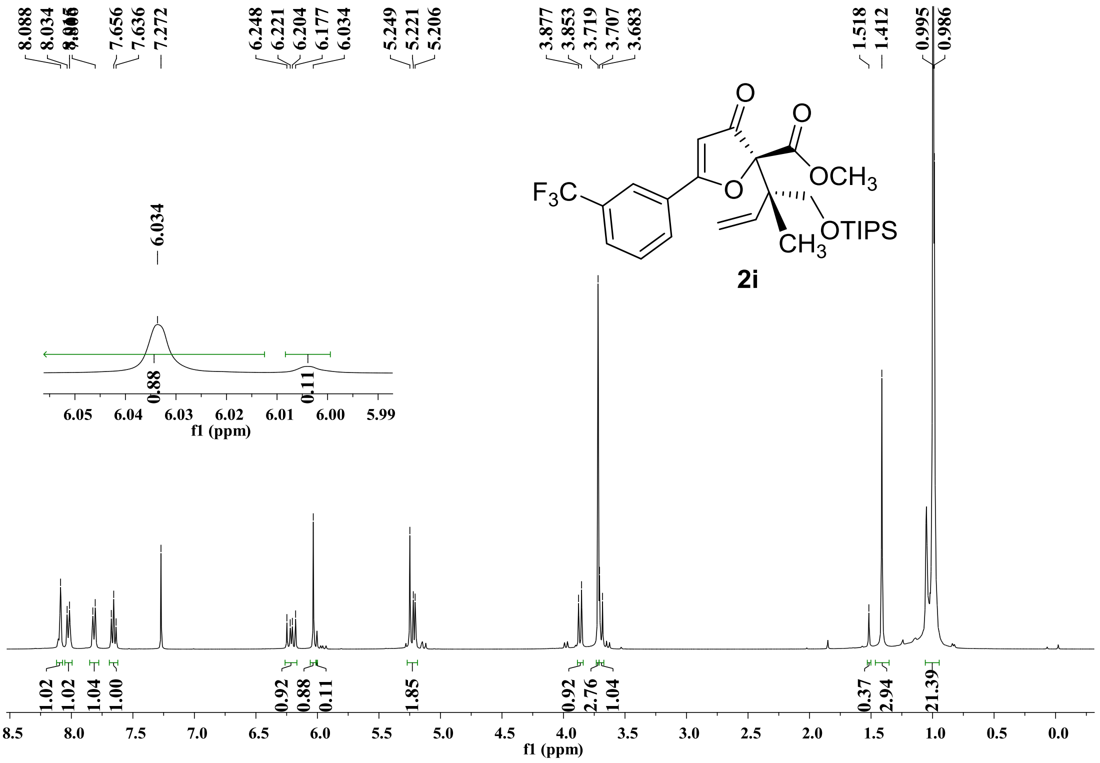

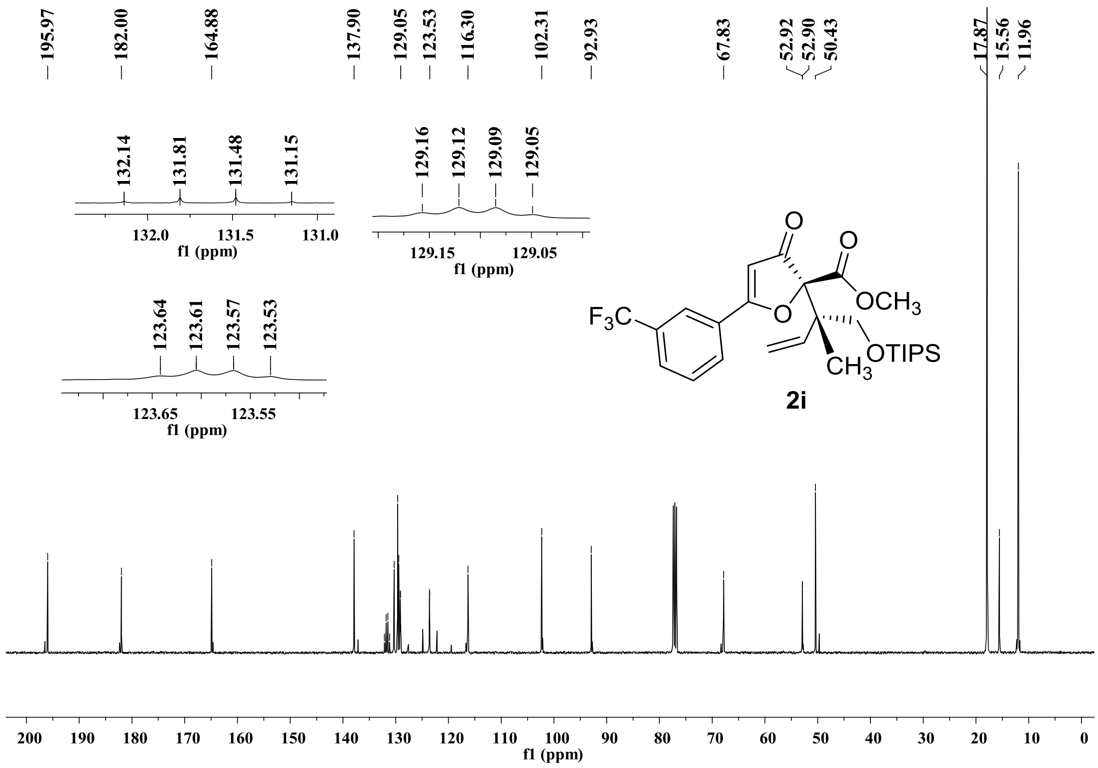

Supplementary Figure 39. ^{1}H and ^{13}C spectra for product **2i**

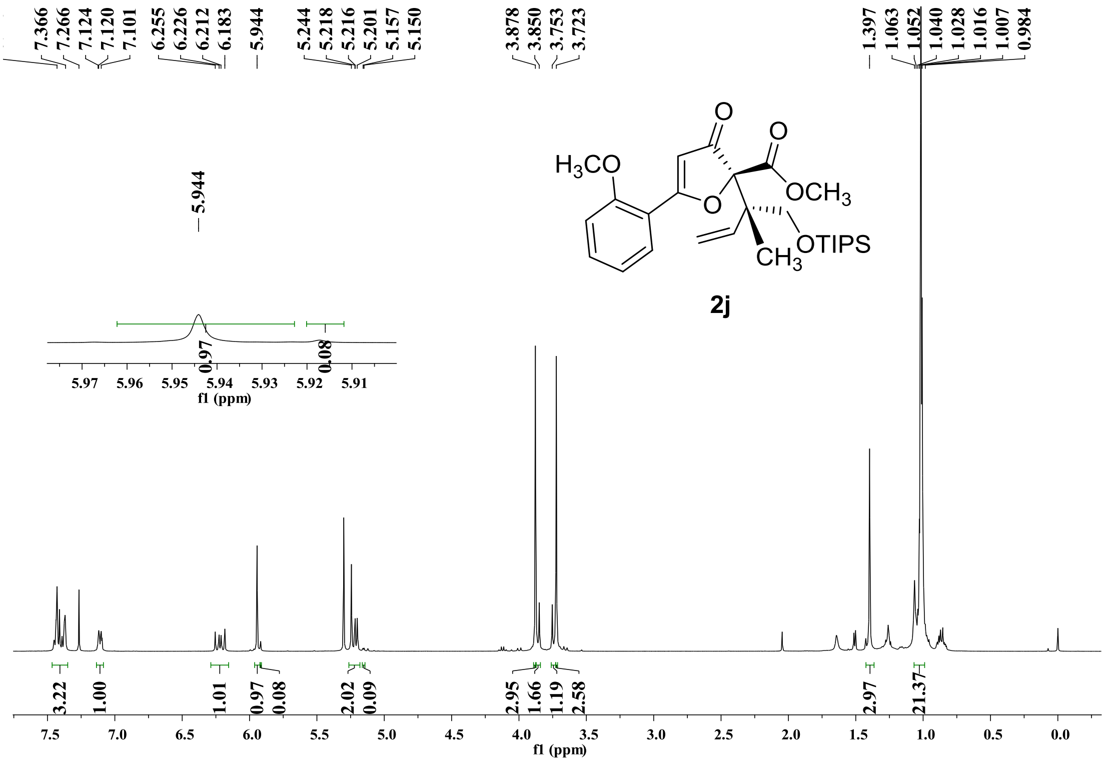

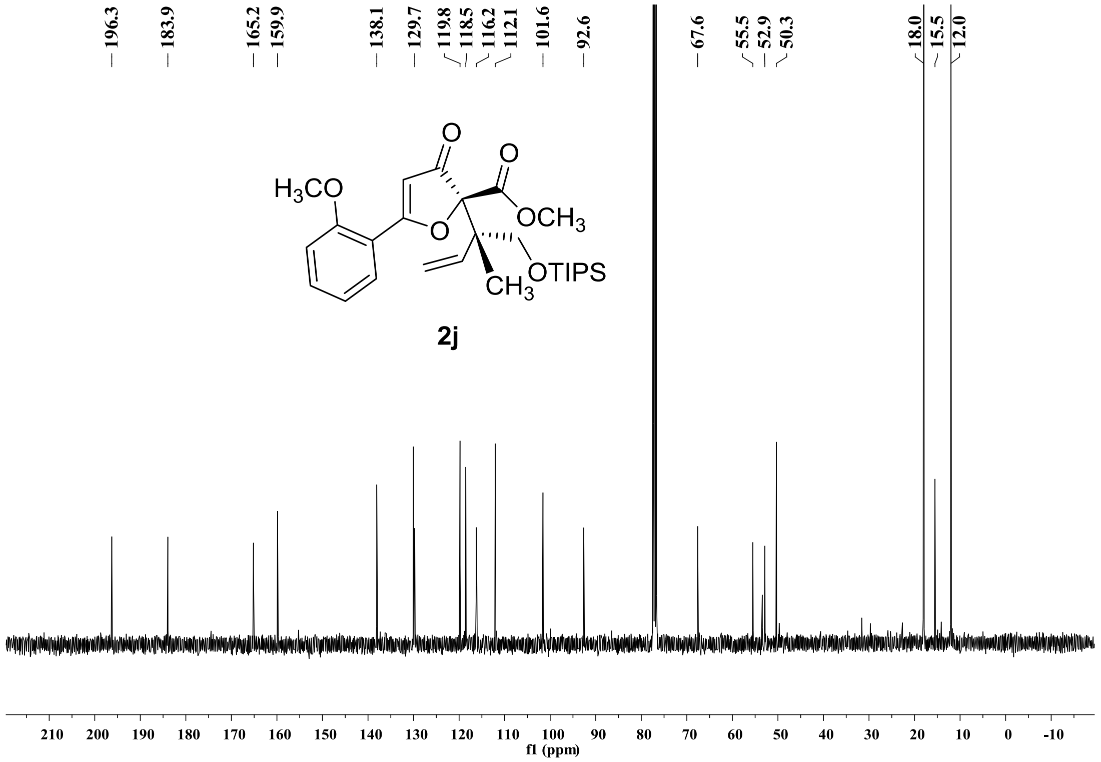

Supplementary Figure 40. ^{1}H and ^{13}C spectra for product **2j**

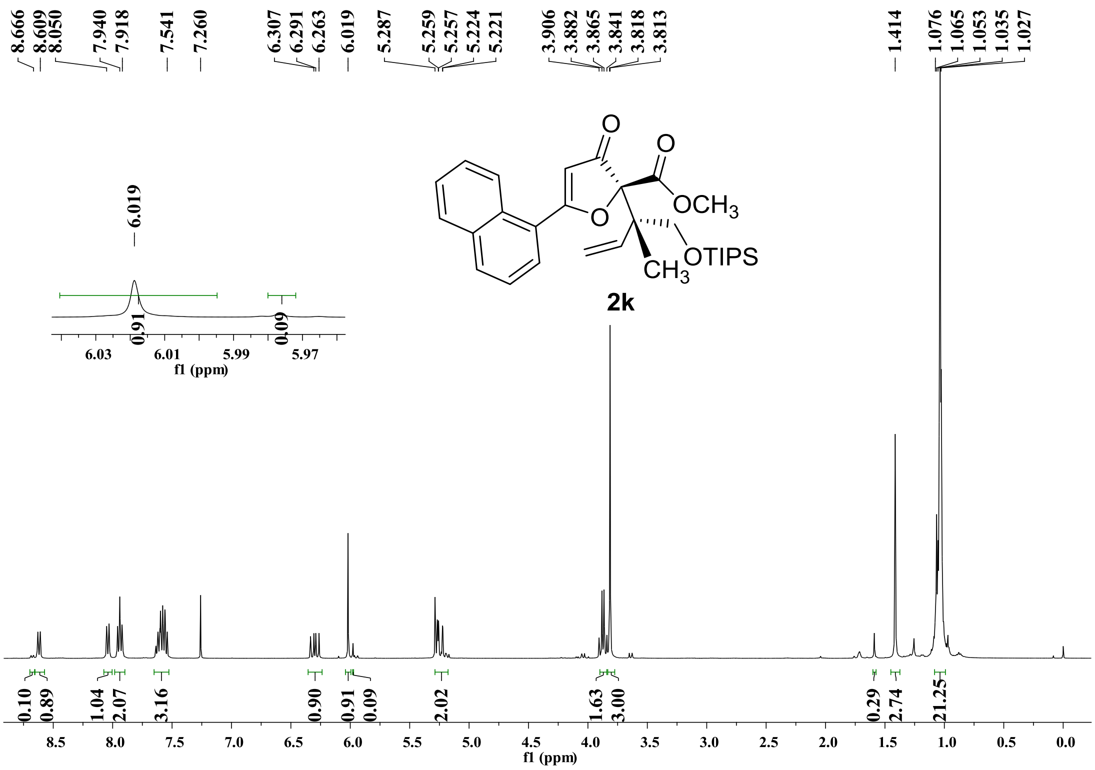

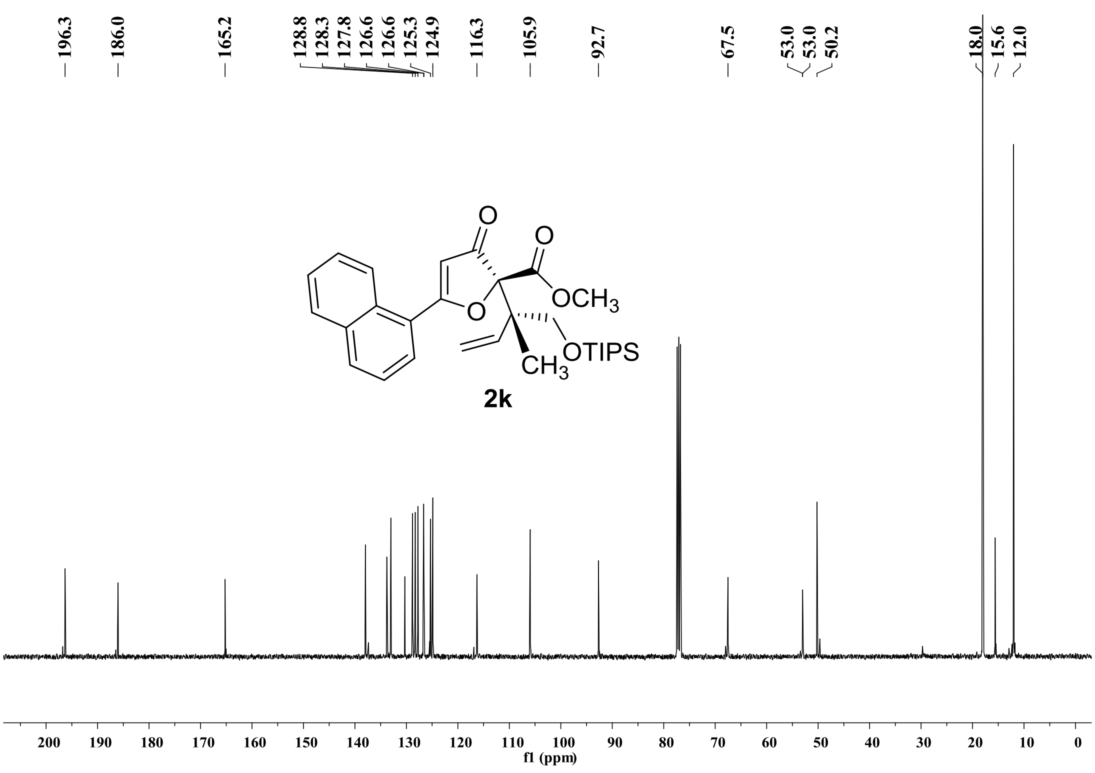

Supplementary Figure 41. ^{1}H and ^{13}C spectra for product **2k**

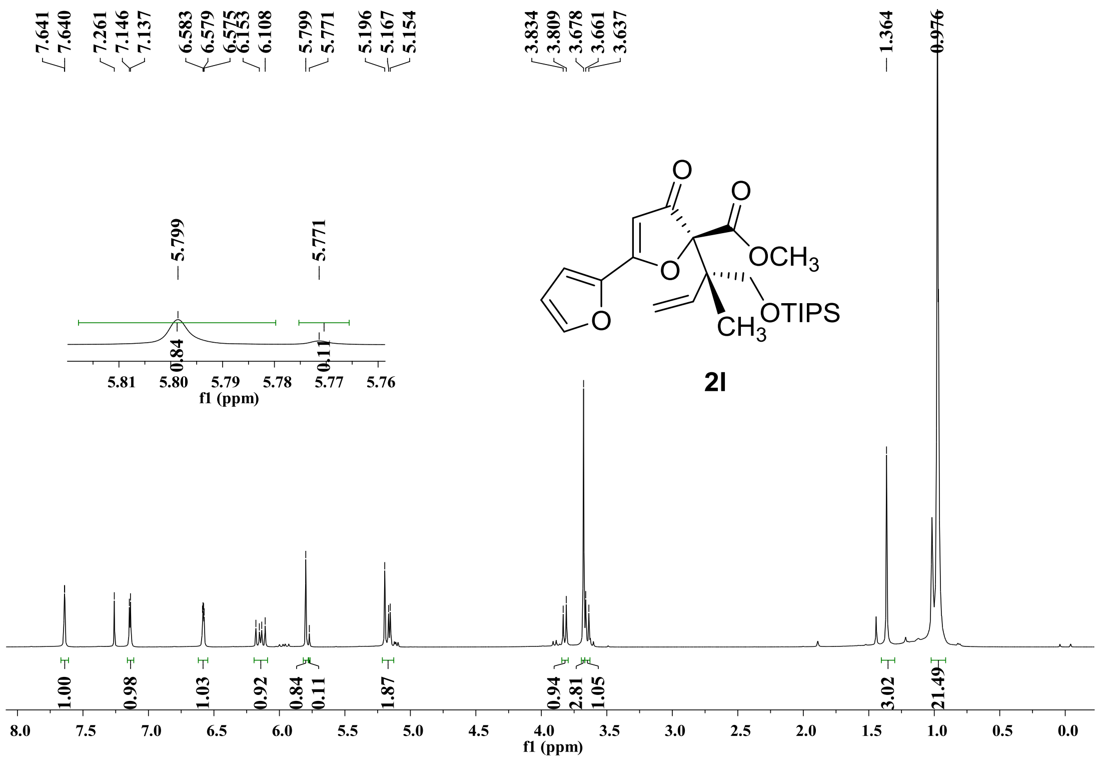

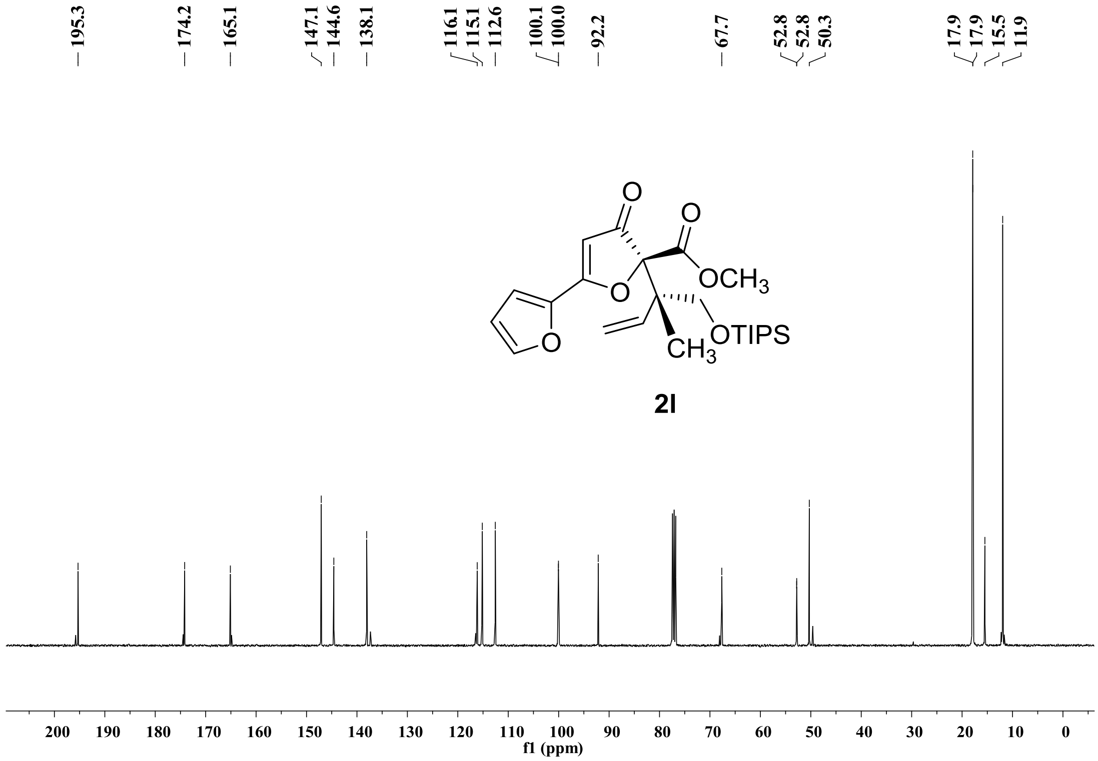

Supplementary Figure 42. ^{1}H and ^{13}C spectra for product **2l**

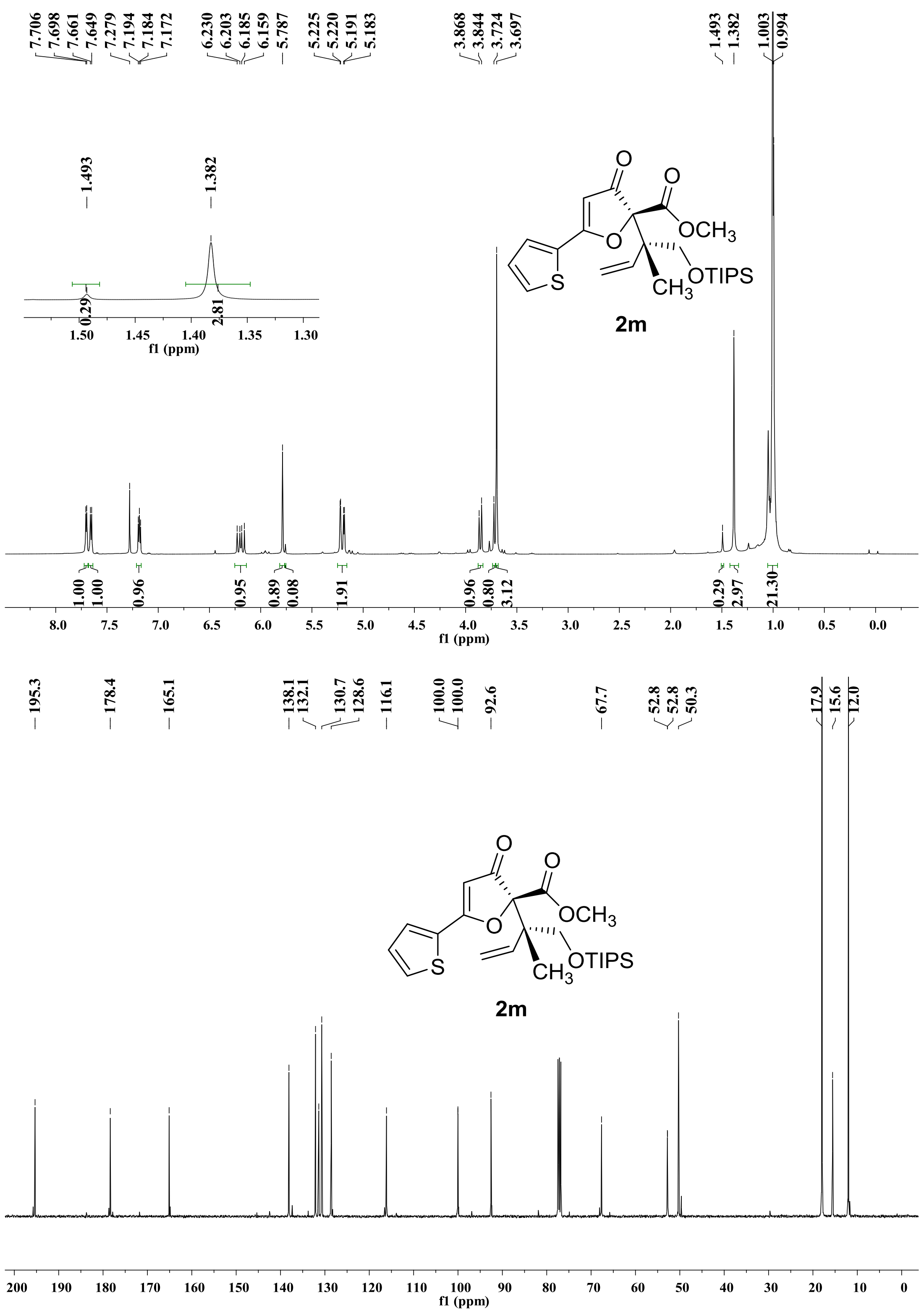

Supplementary Figure 43. ^{1}H and ^{13}C spectra for product **2m**

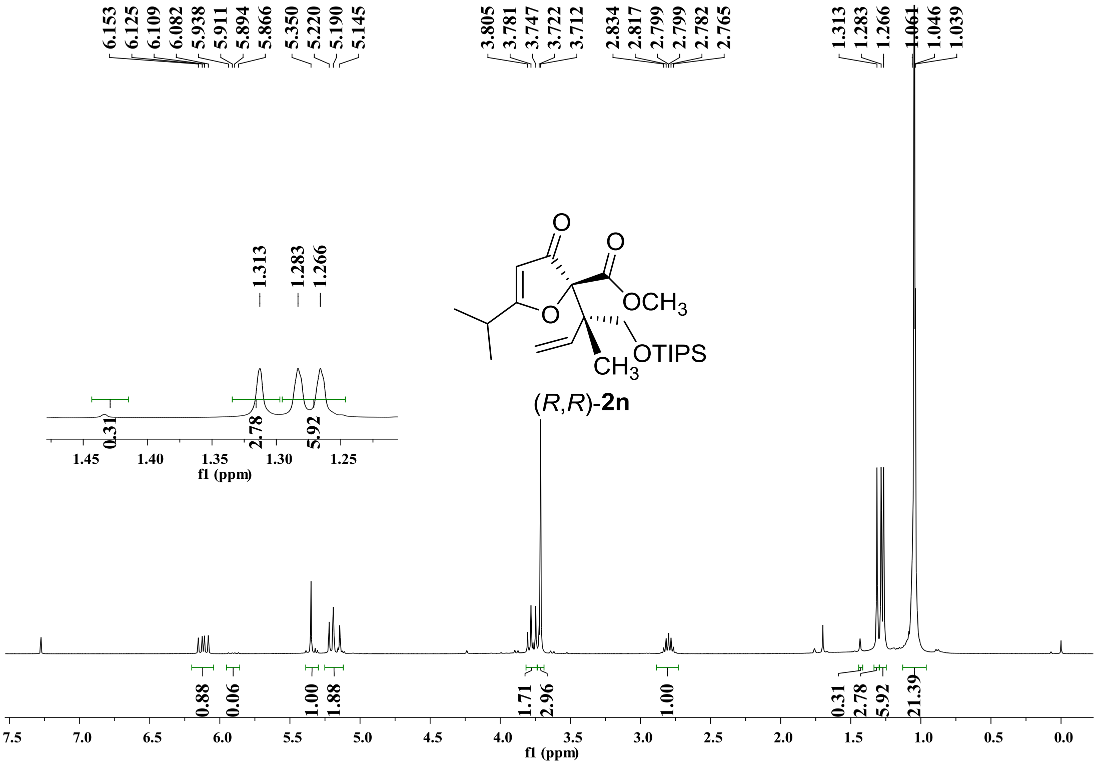

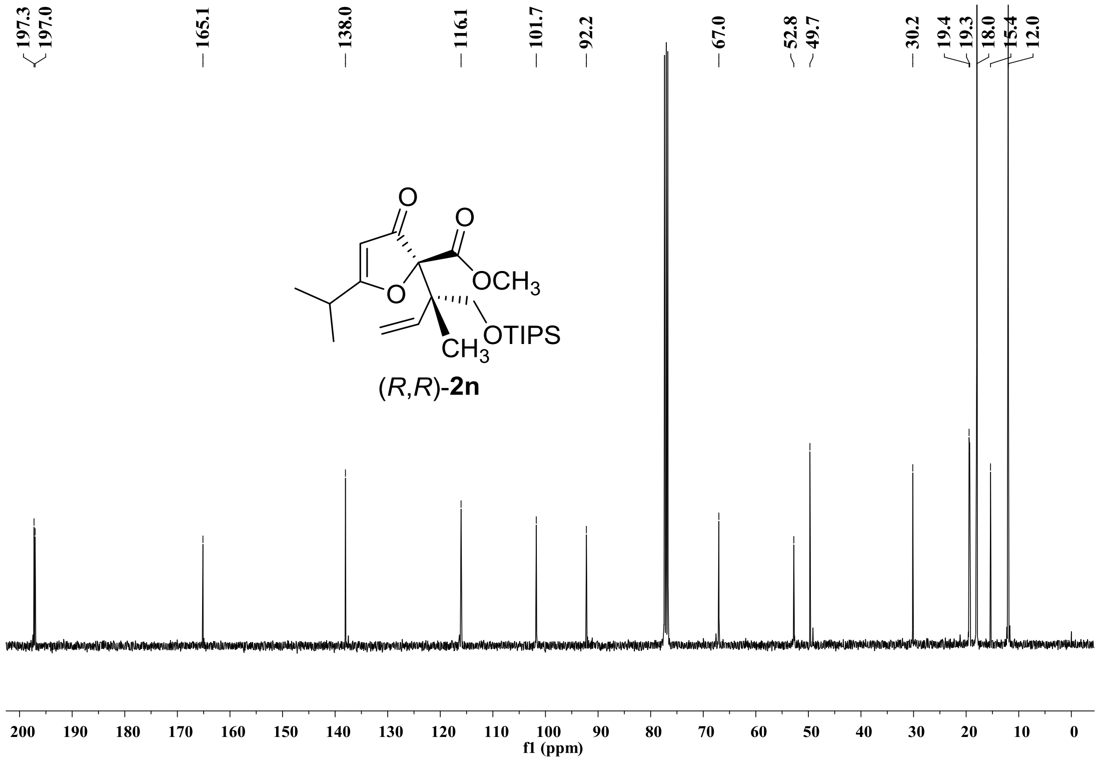

Supplementary Figure 44. ^{1}H and ^{13}C spectra for product (*R*,*R*)-**2n**

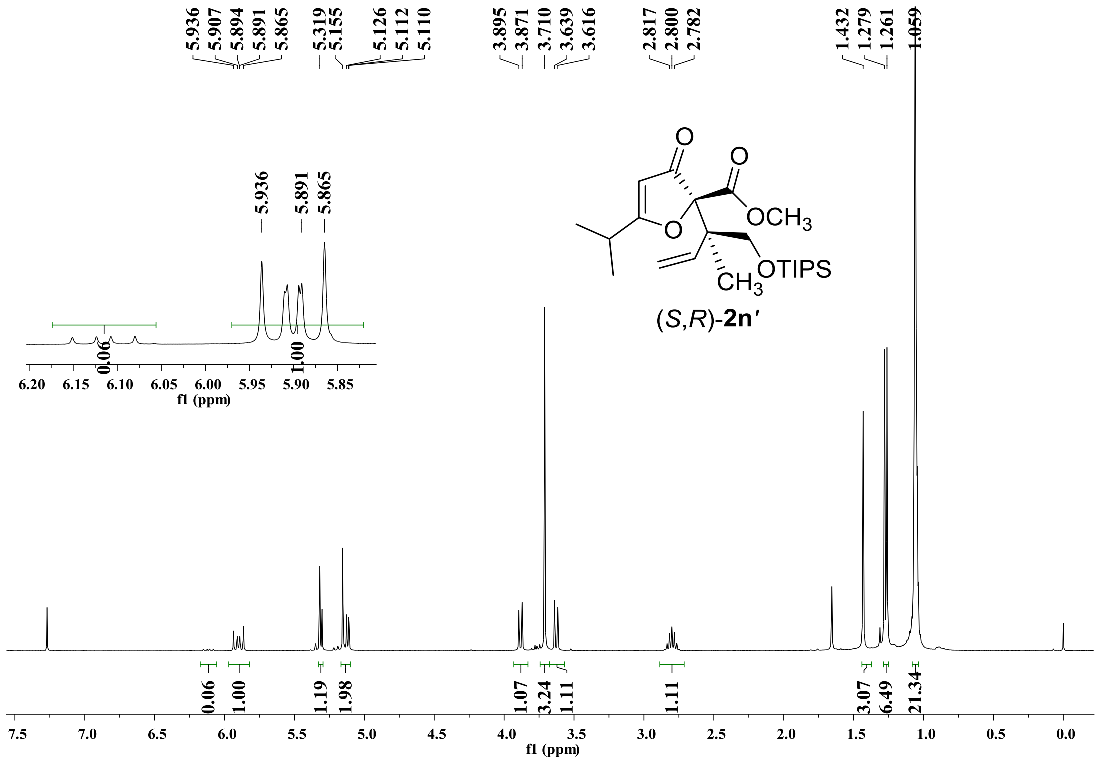

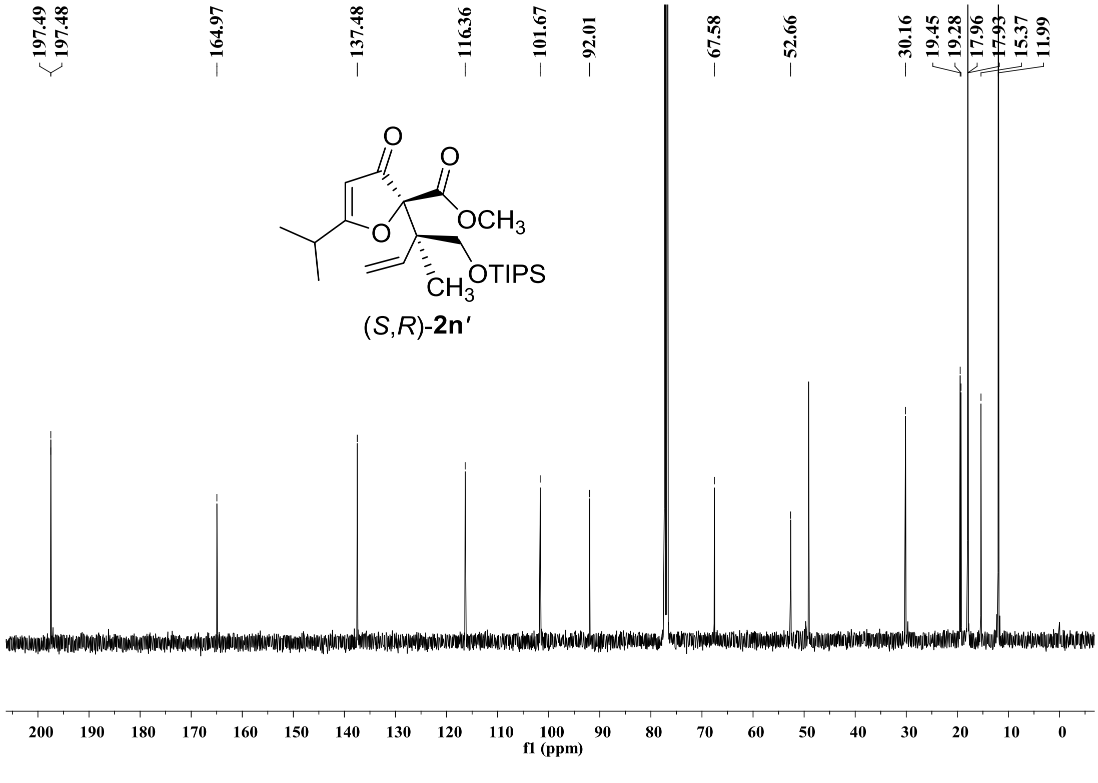

Supplementary Figure 45. ^{1}H and ^{13}C spectra for product (*S*,*R*)-**2n'**

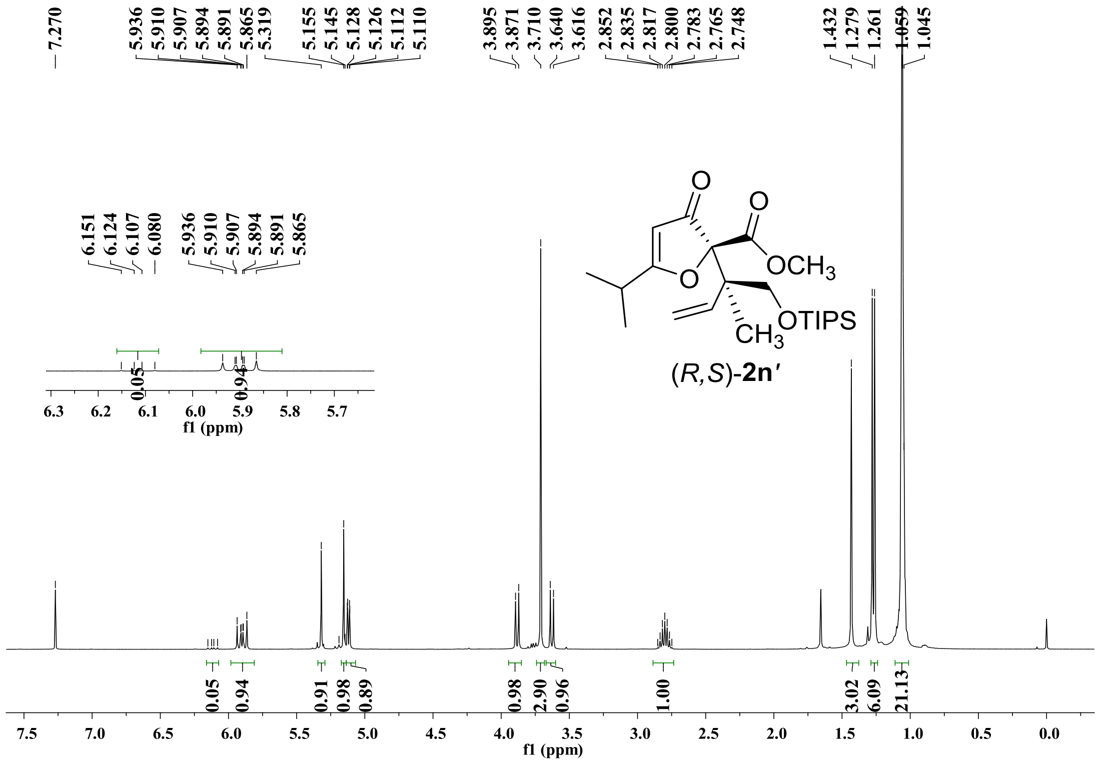

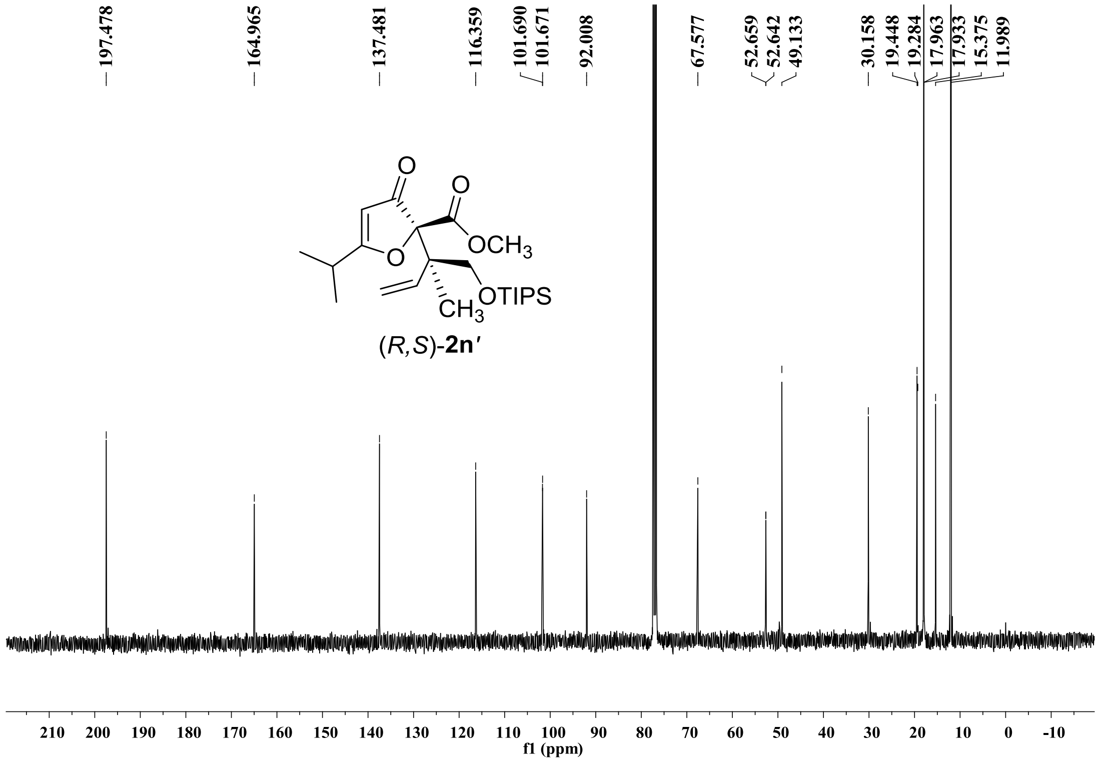

Supplementary Figure 46. ^{1}H and ^{13}C spectra for product (*R*,*S*)-**2n'**

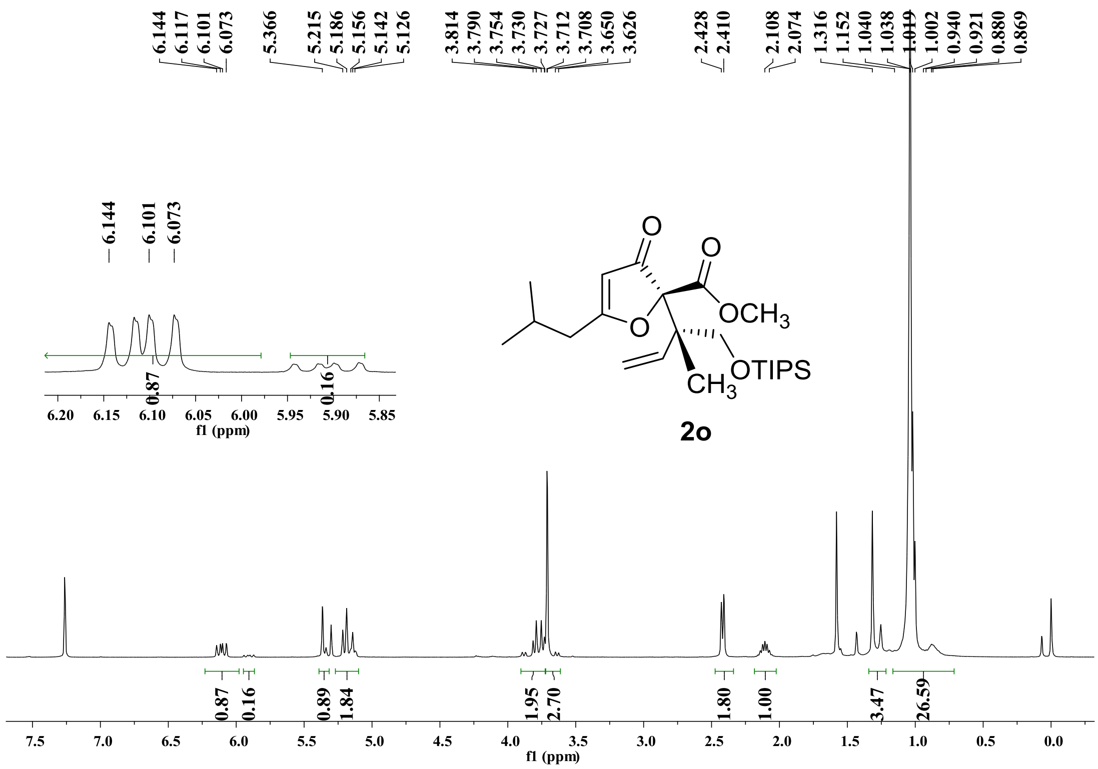

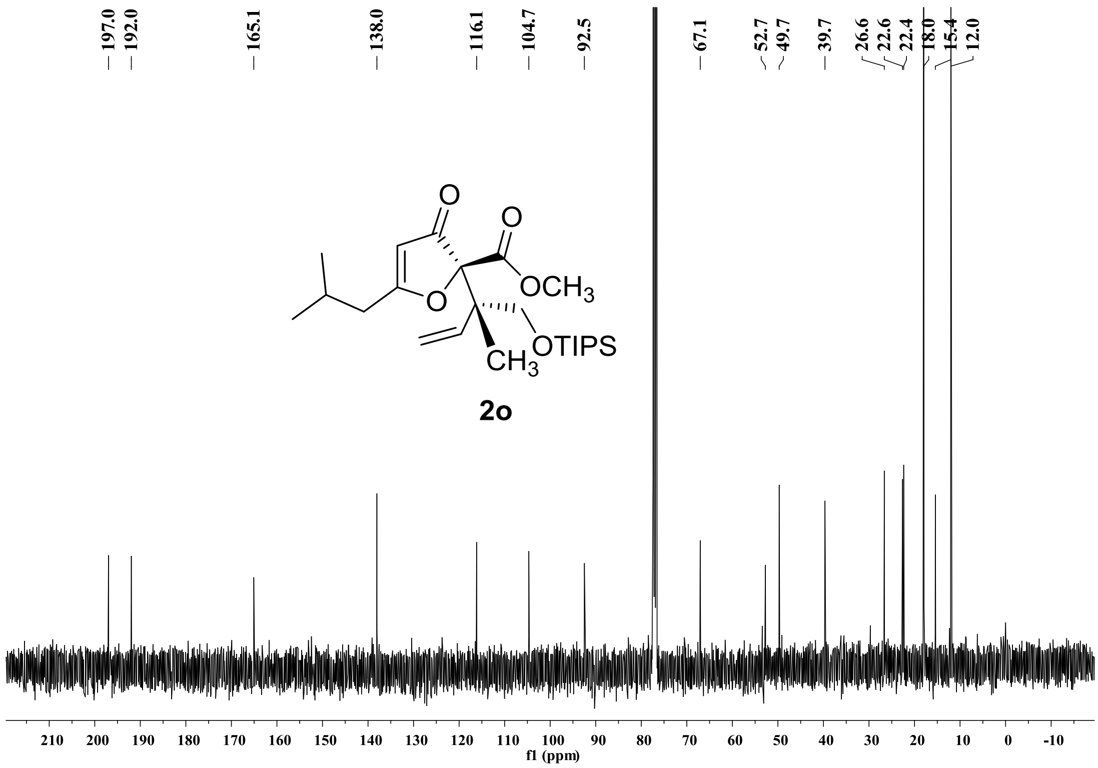

Supplementary Figure 47. ^{1}H and ^{13}C spectra for product **2o**

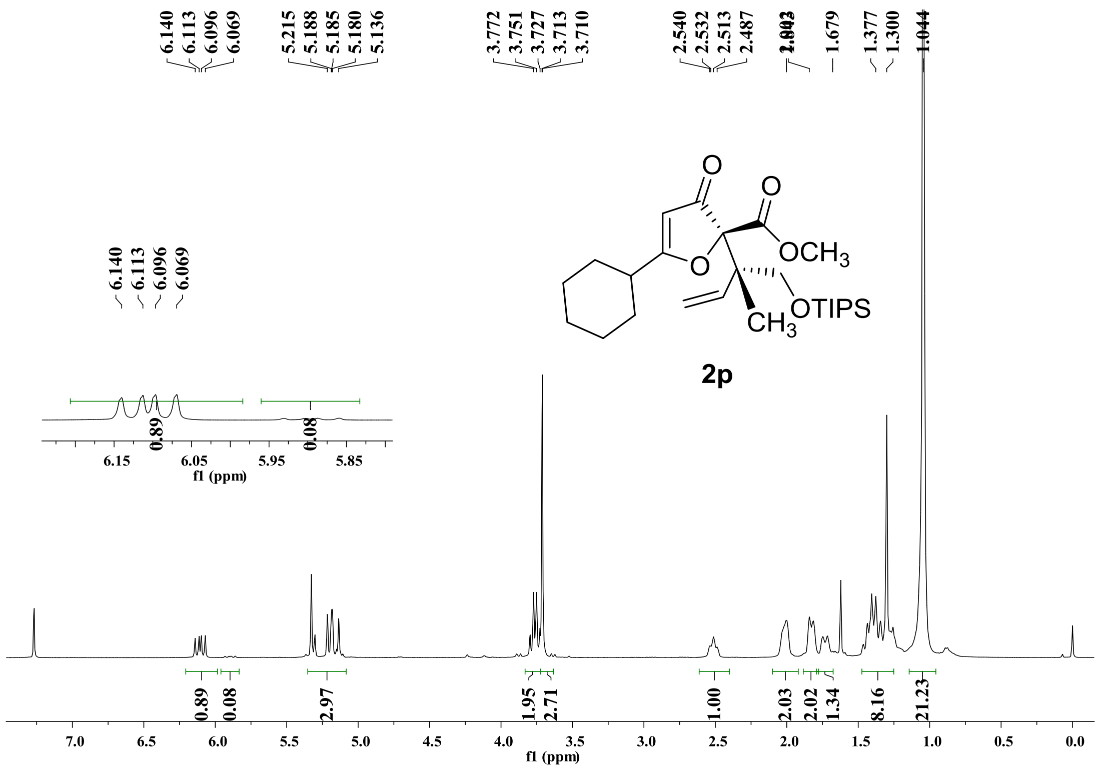

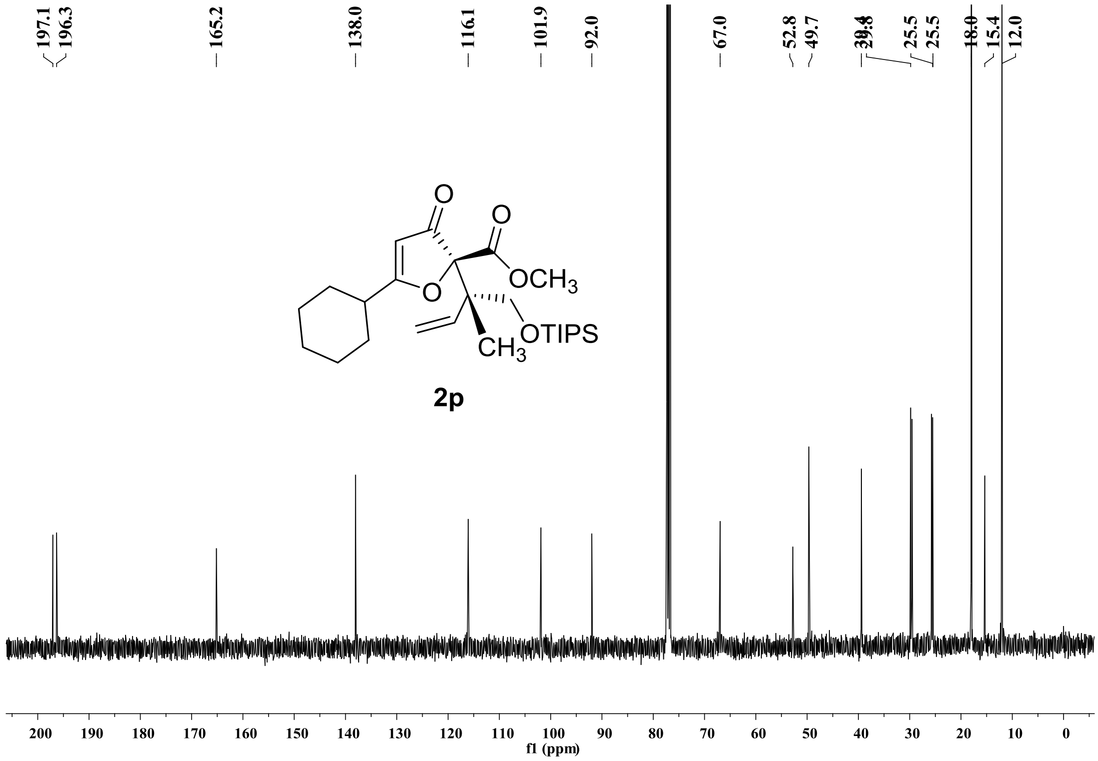

Supplementary Figure 48. ^{1}H and ^{13}C spectra for product **2p**

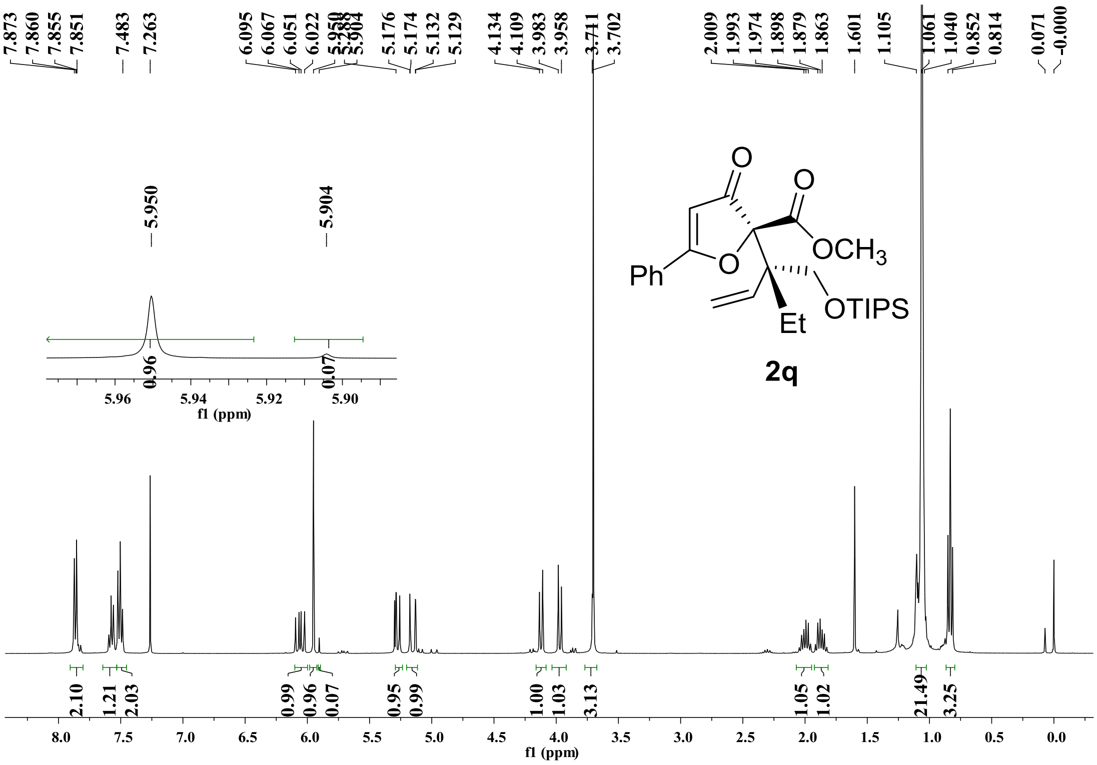

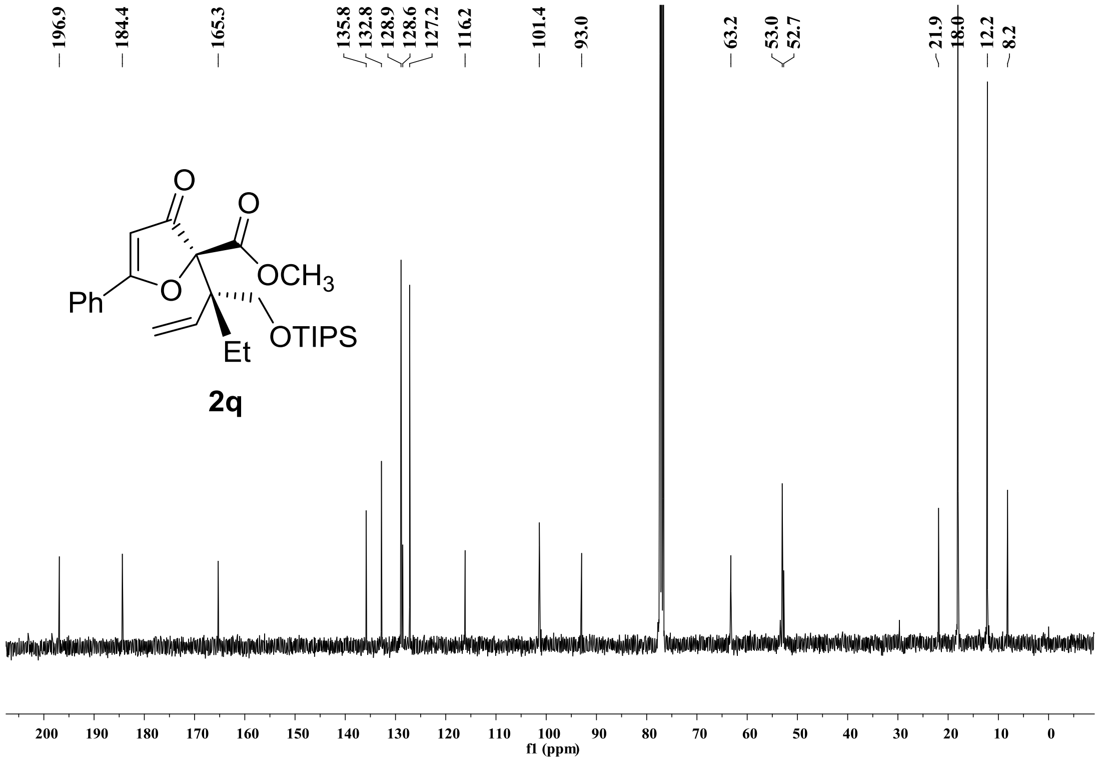

Supplementary Figure 49. ^{1}H and ^{13}C spectra for product **2q**

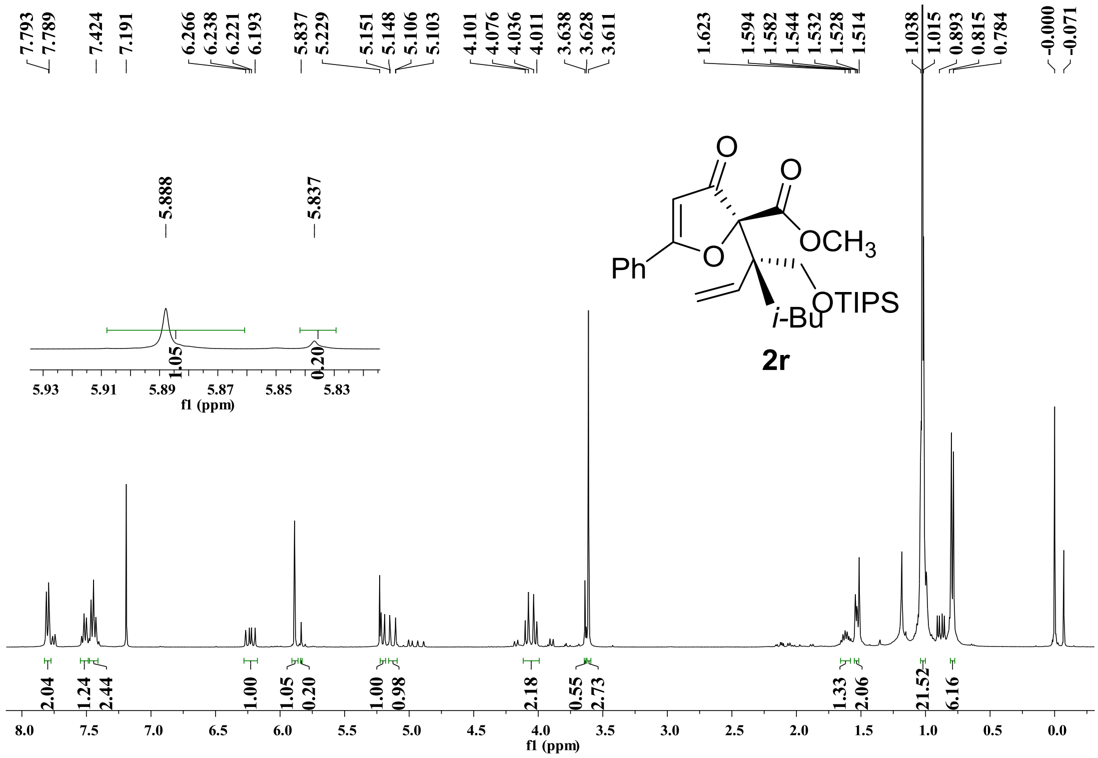

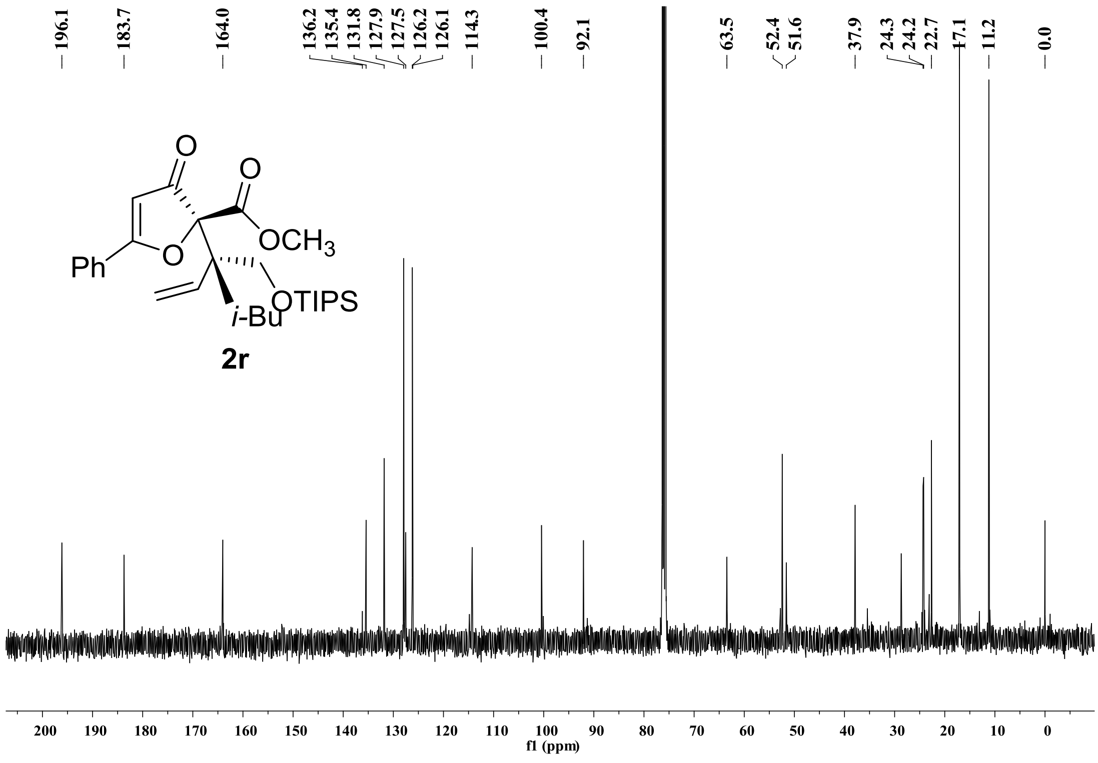

Supplementary Figure 50. ^{1}H and ^{13}C spectra for product **2r**

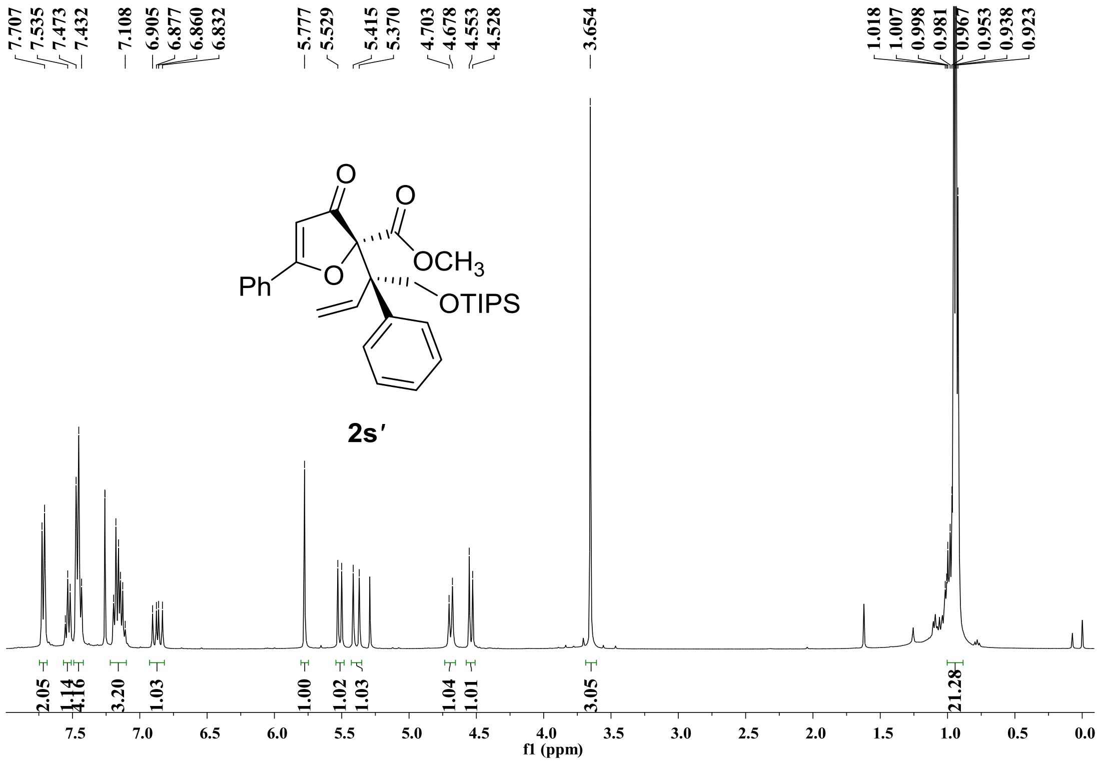

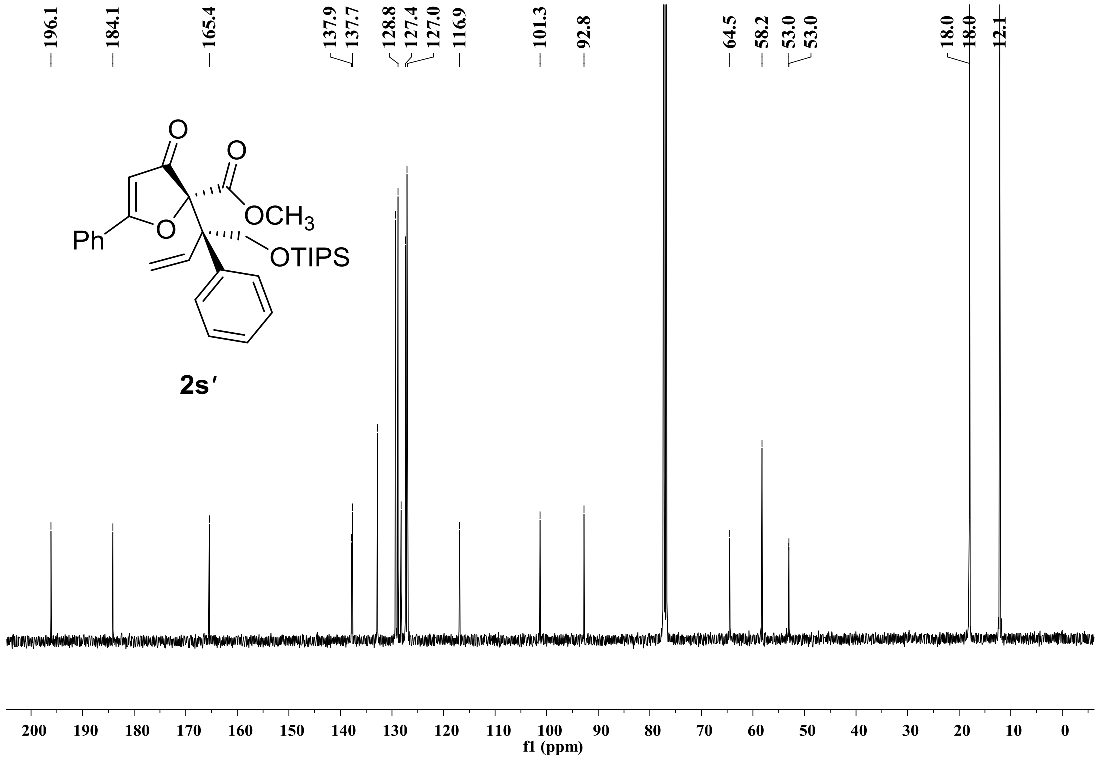

Supplementary Figure 51. ^{1}H and ^{13}C spectra for product **2s'**

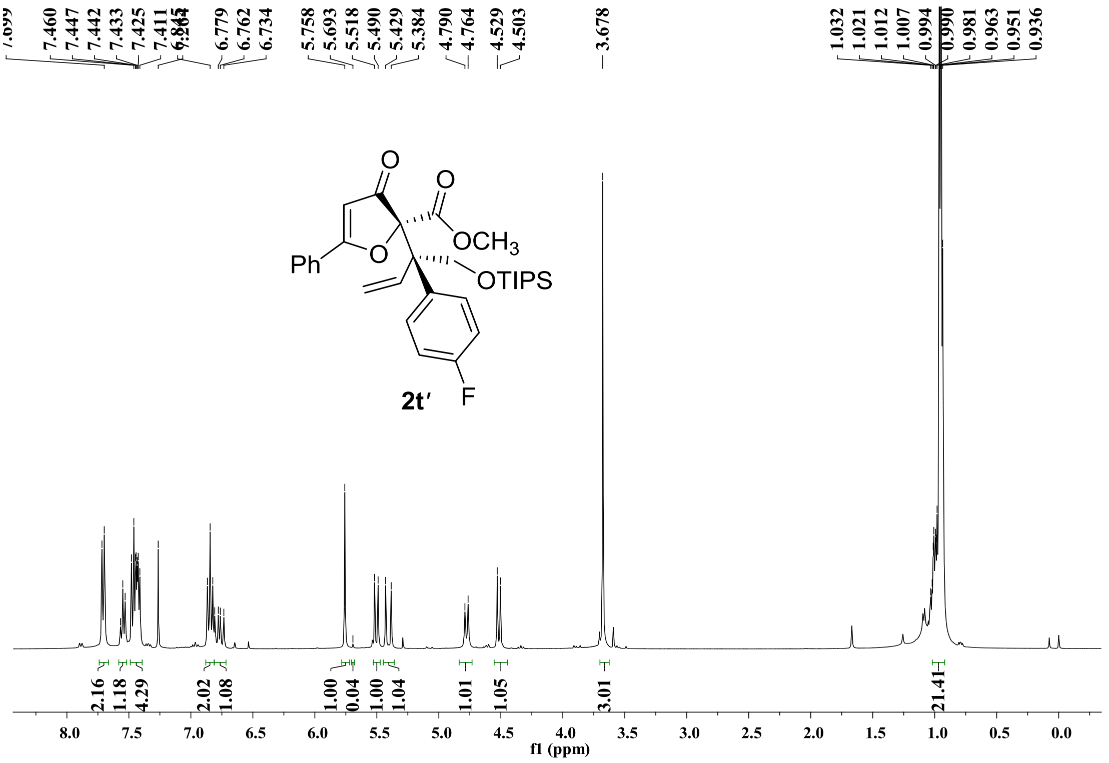

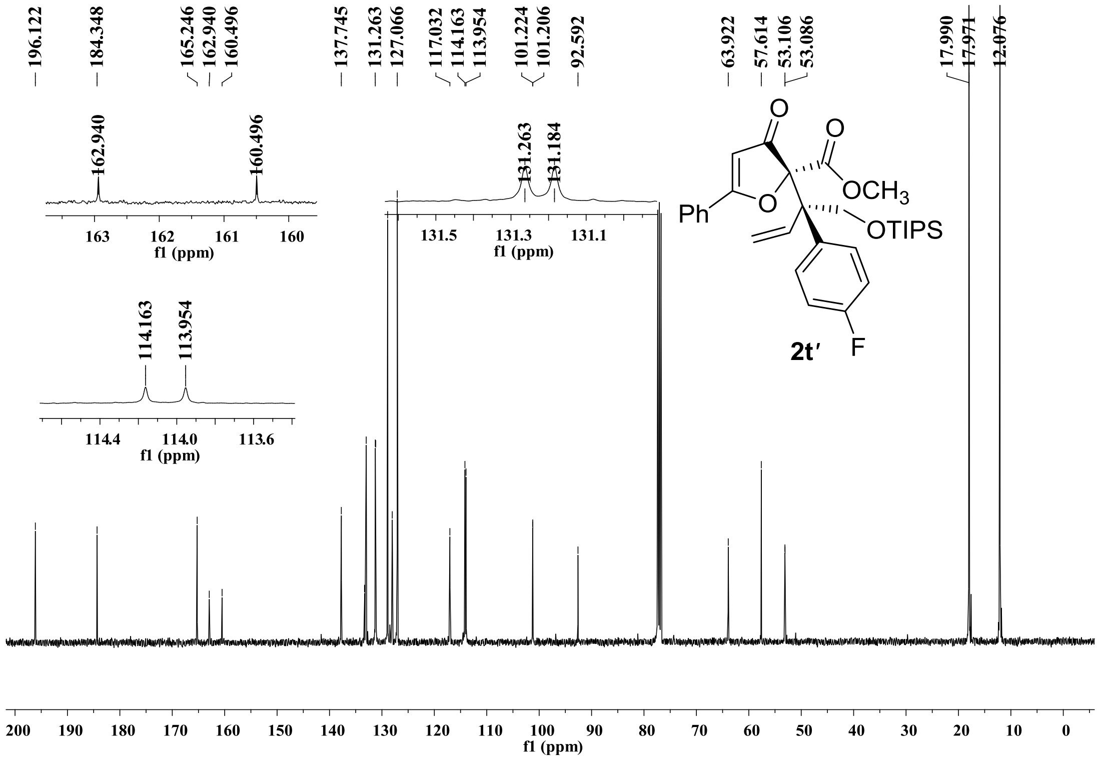

Supplementary Figure 52. ^{1}H and ^{13}C spectra for product **2t'**

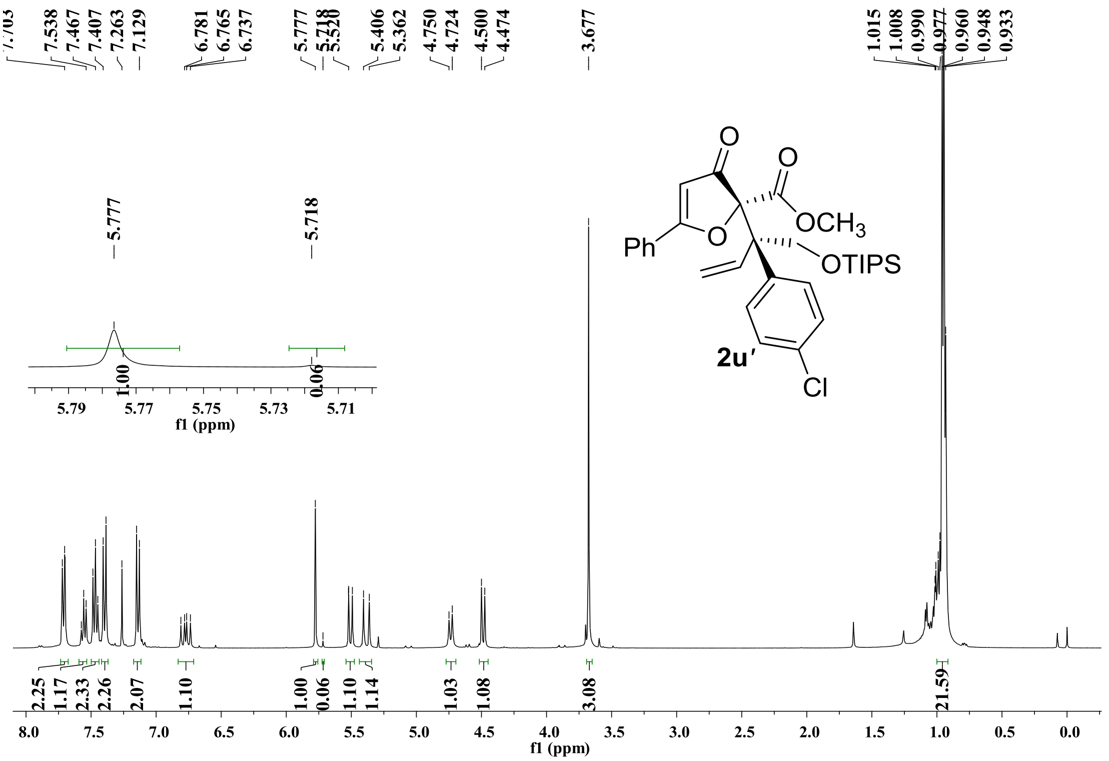

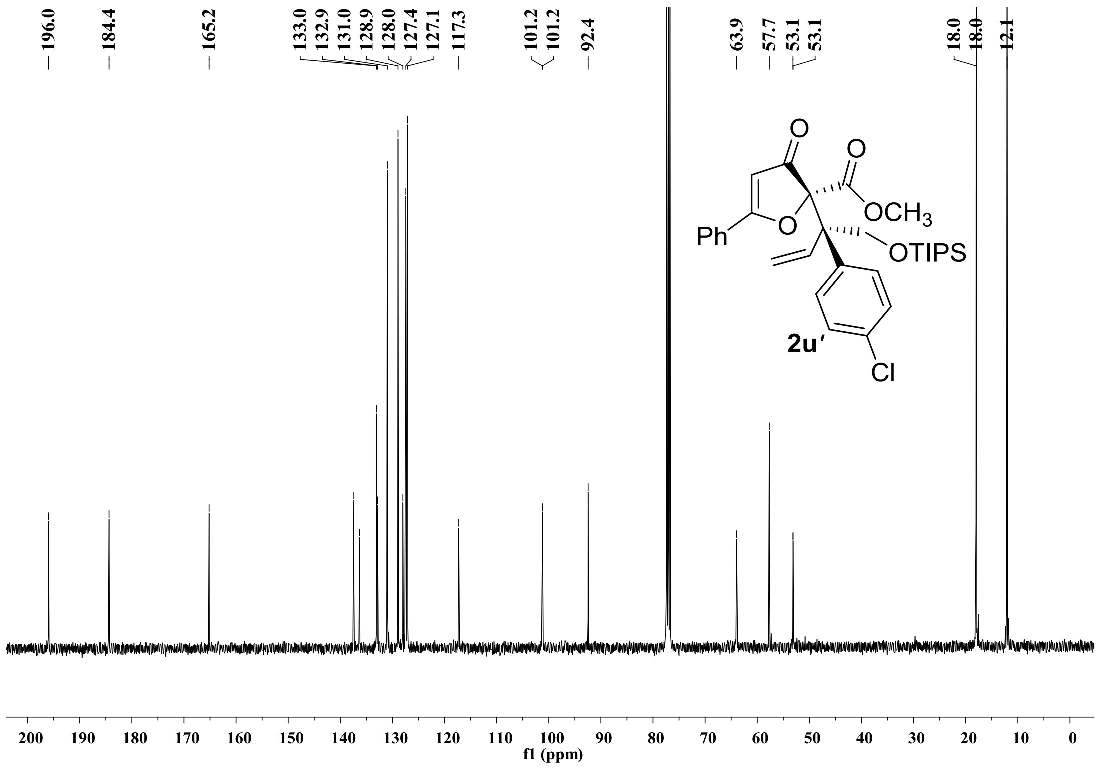

Supplementary Figure 53. ^{1}H and ^{13}C spectra for product **2u'**

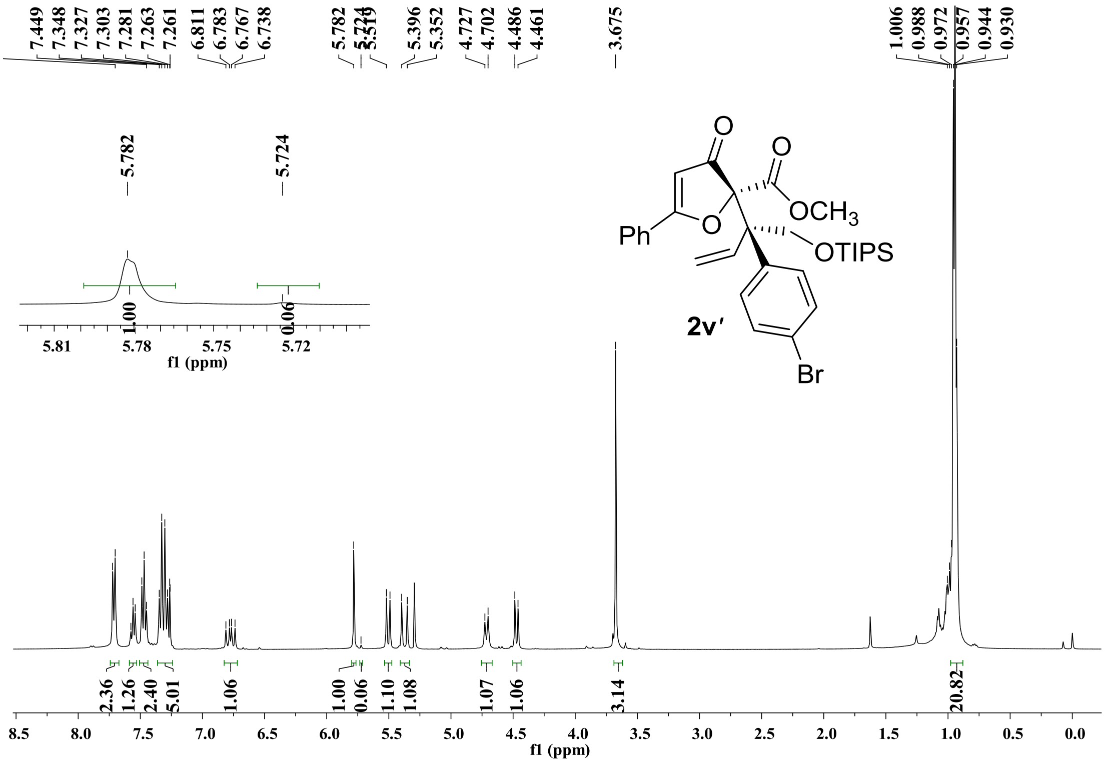

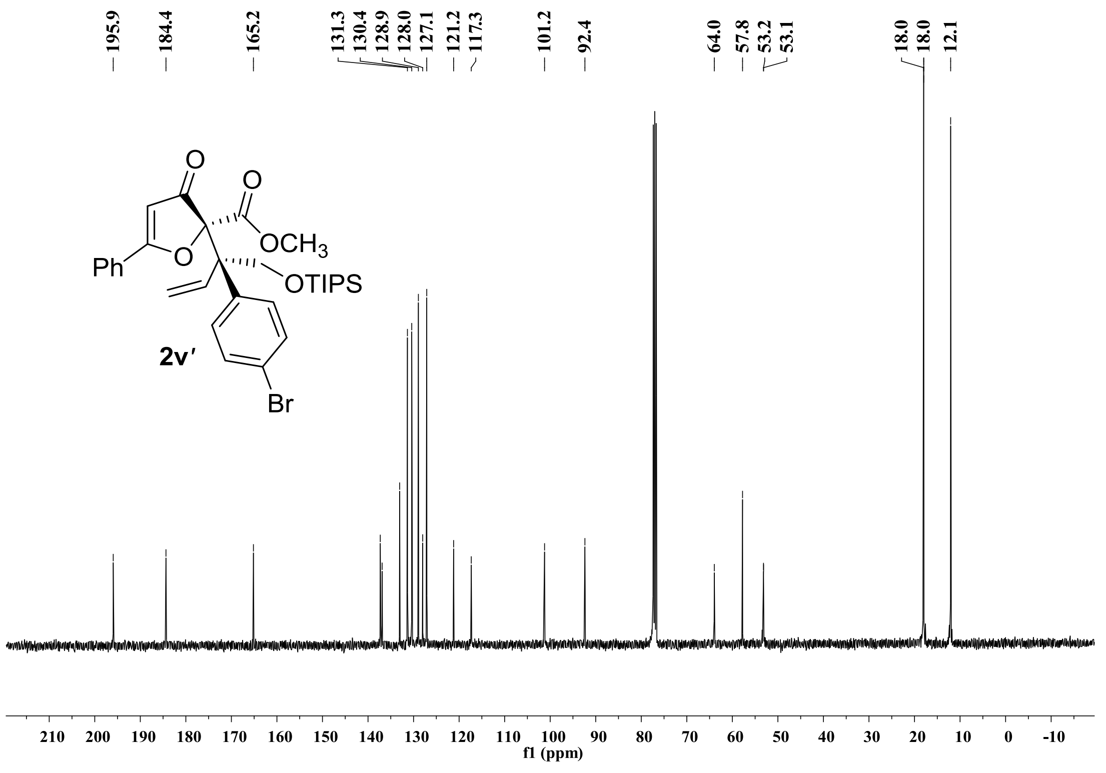

Supplementary Figure 54. ^{1}H and ^{13}C spectra for product **2v'**

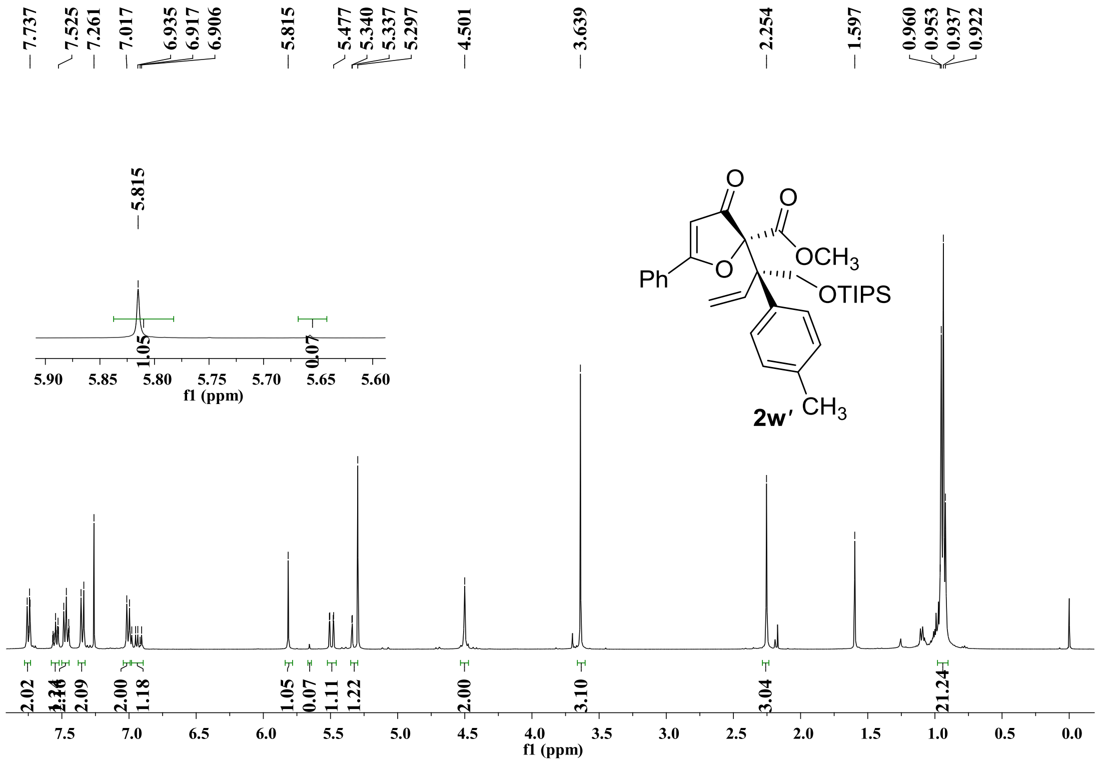

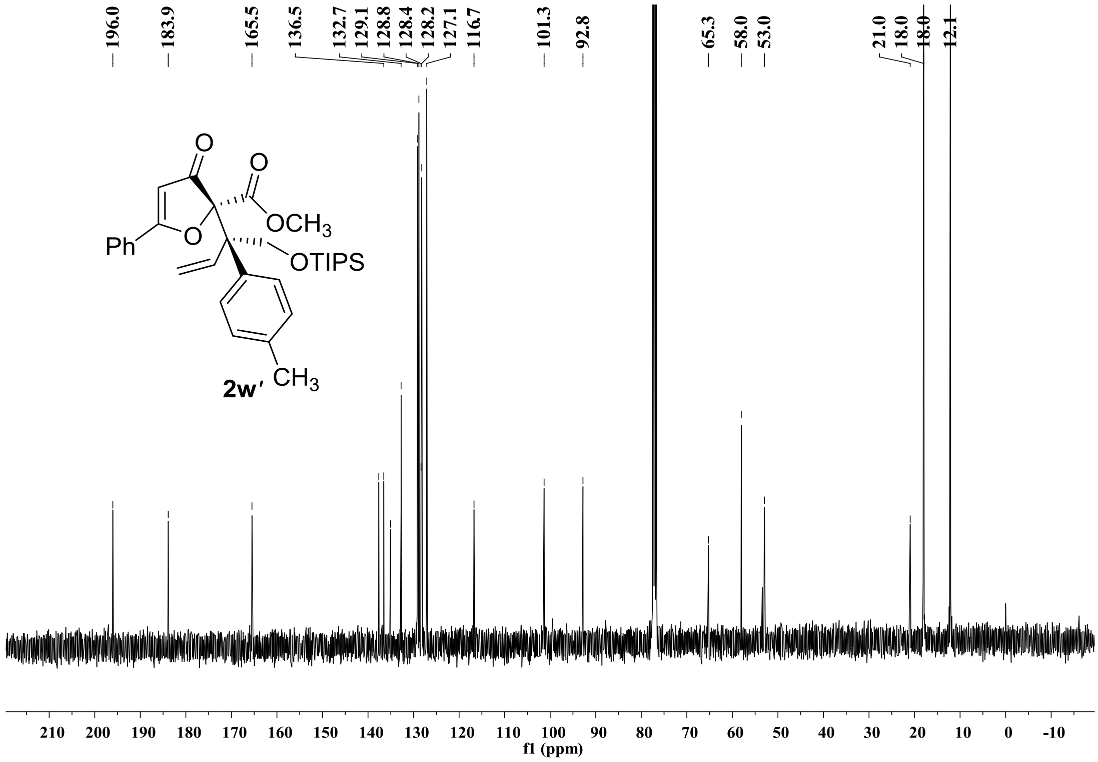

Supplementary Figure 55. ^{1}H and ^{13}C spectra for product **2w'**

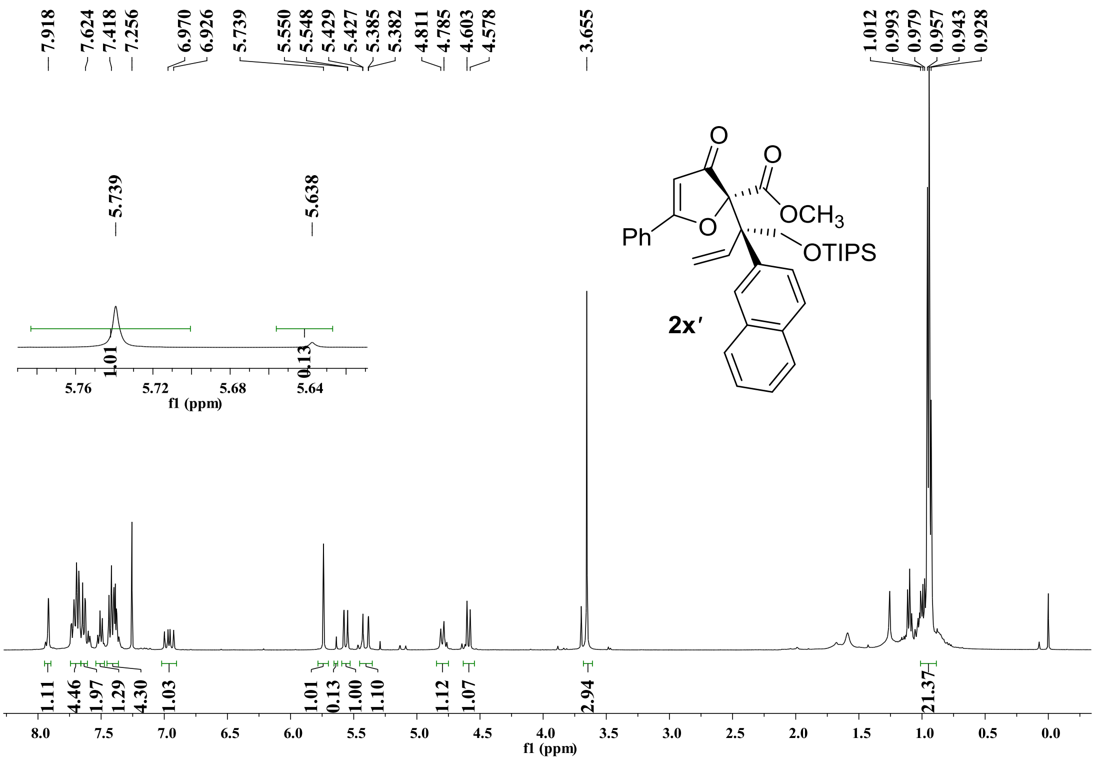

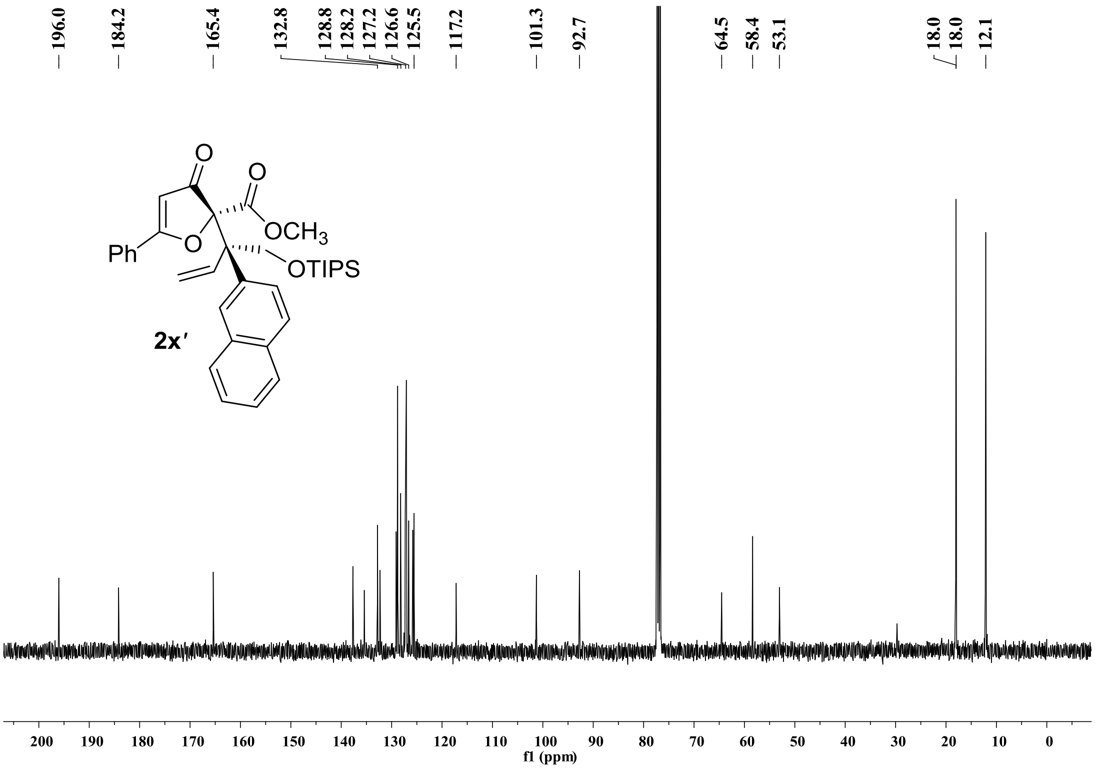

Supplementary Figure 56. ^{1}H and ^{13}C spectra for product **2x'**

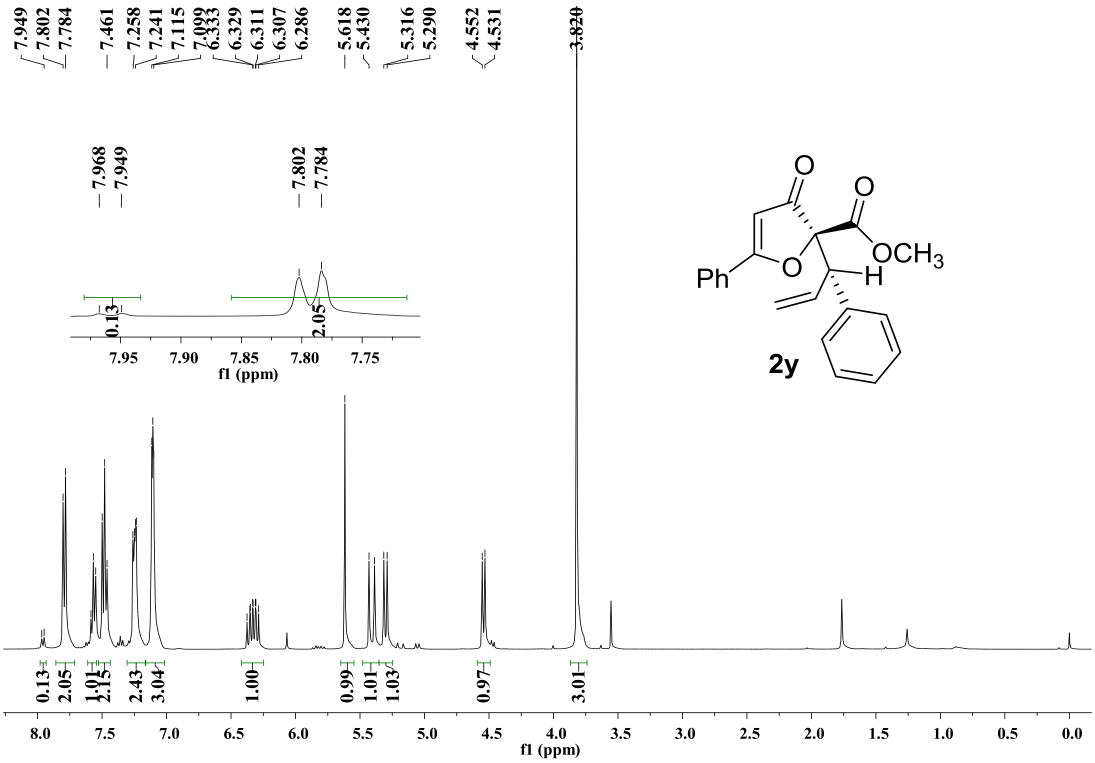

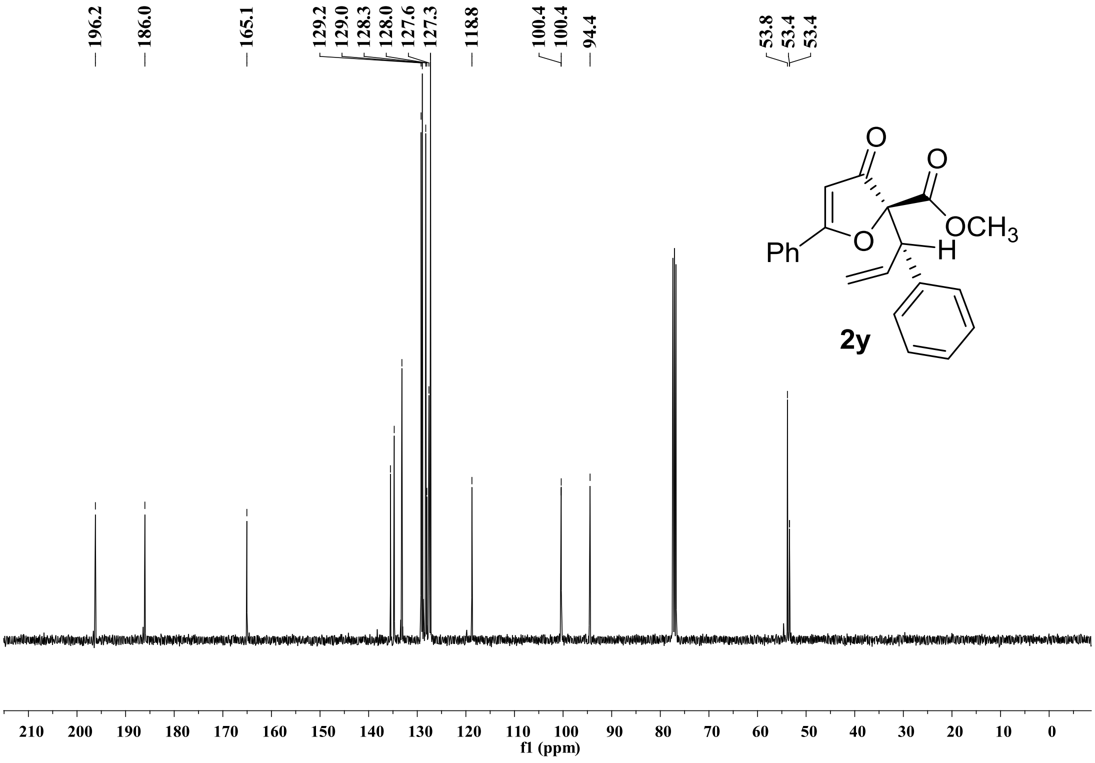

Supplementary Figure 57. ^{1}H and ^{13}C spectra for product **2y**

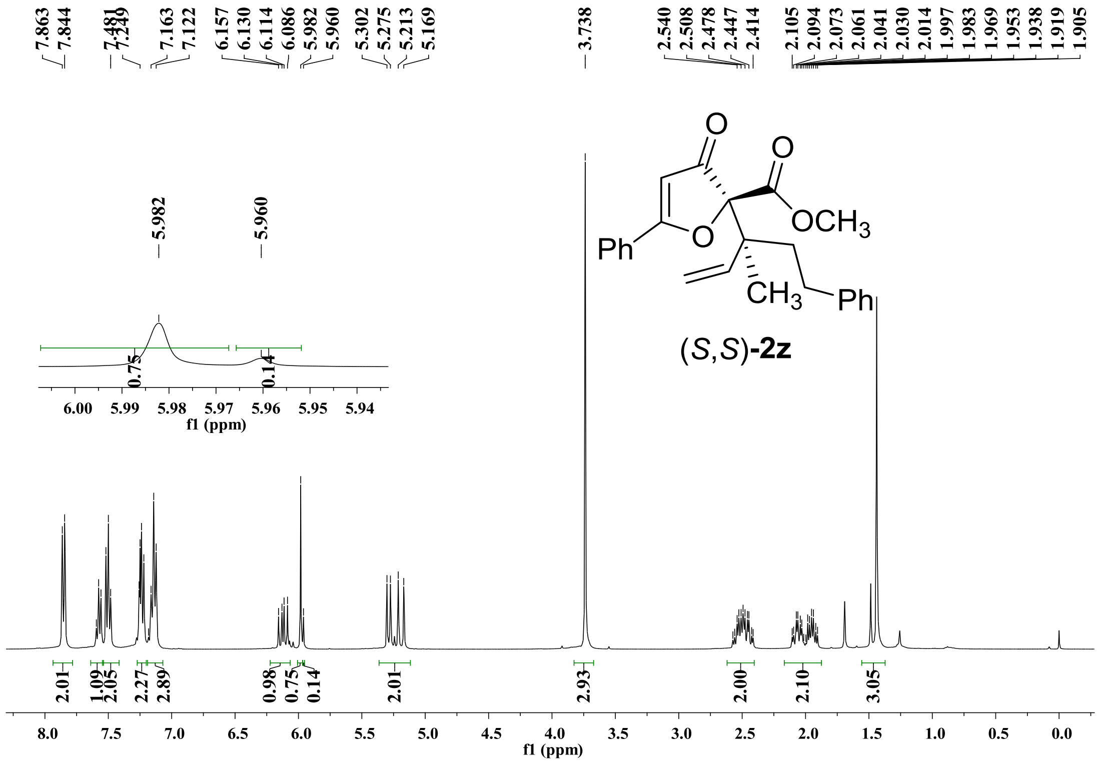

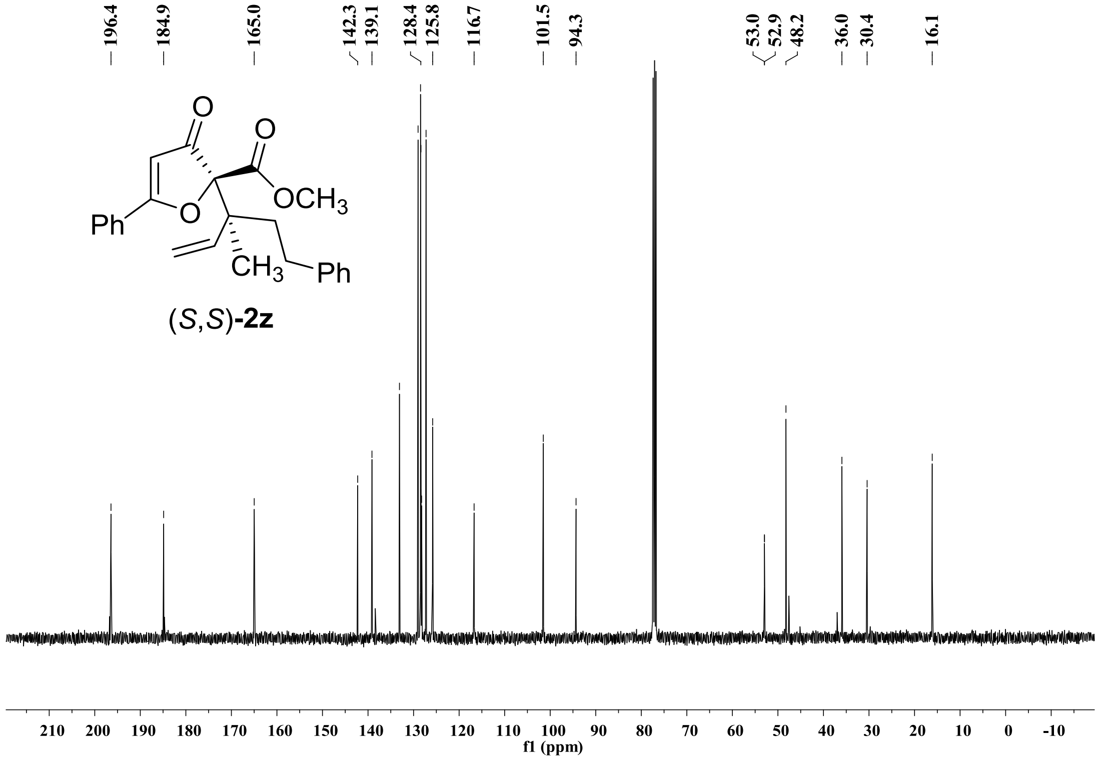

Supplementary Figure 58. ^{1}H and ^{13}C spectra for product (*S*,*S*)-**2z**

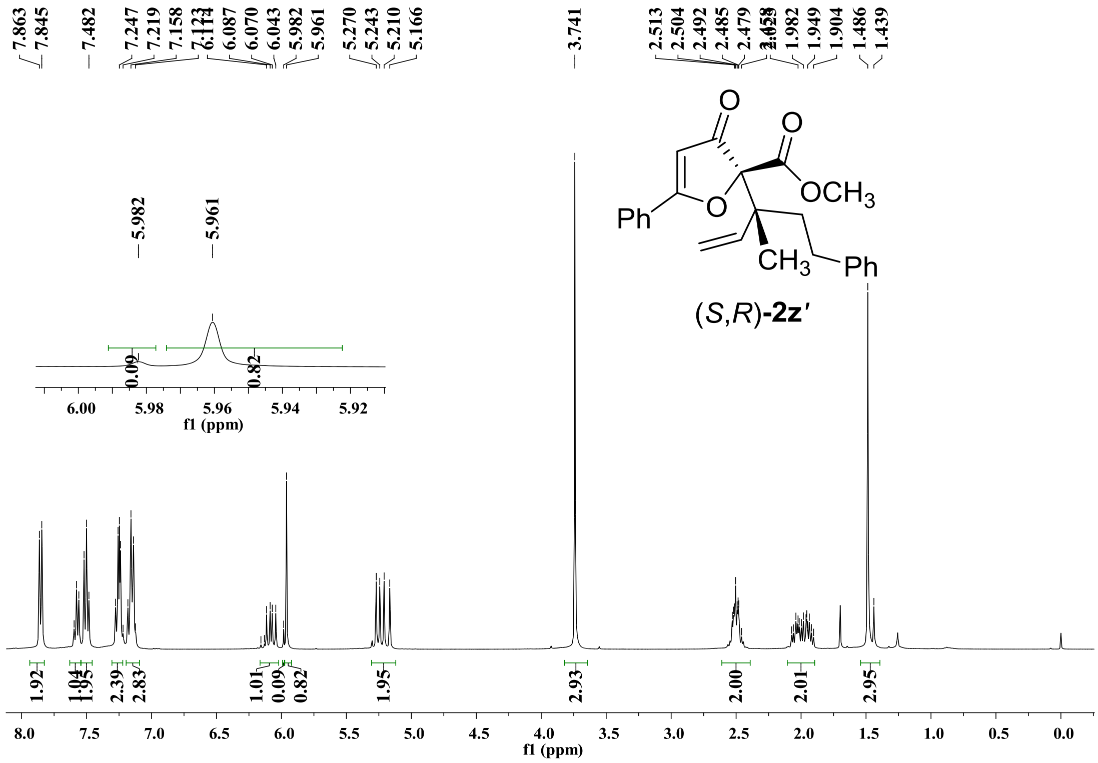

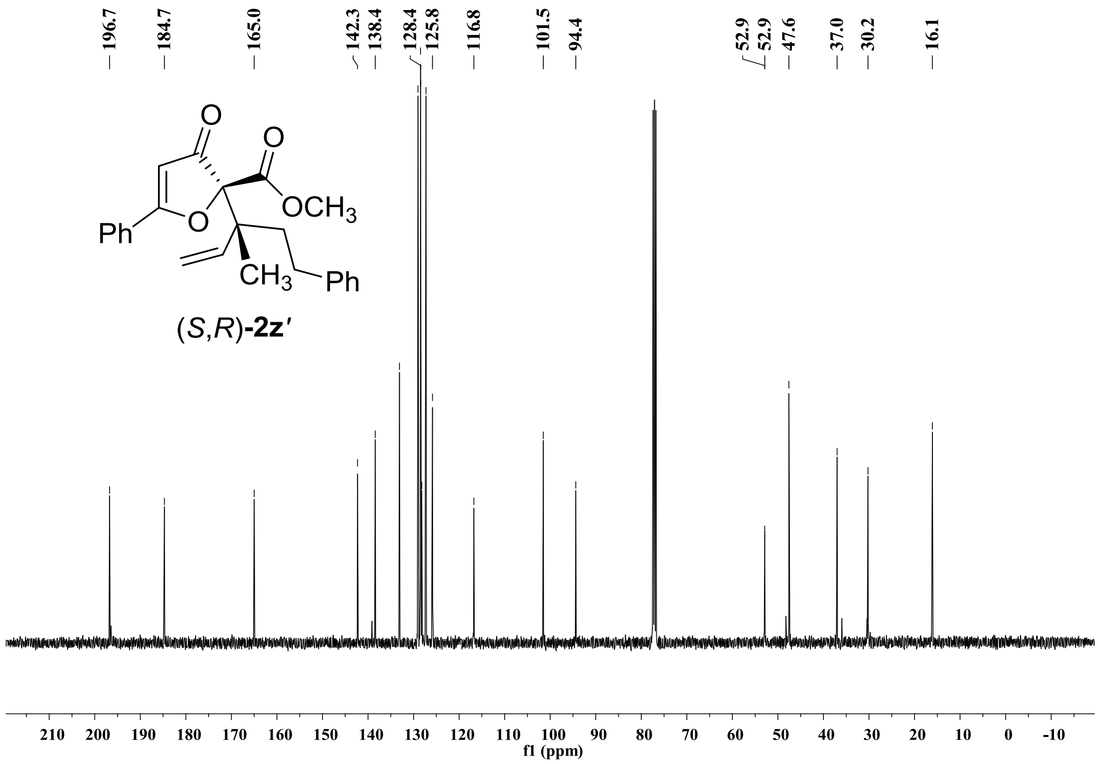

Supplementary Figure 59. ^{1}H and ^{13}C spectra for product (*S*,*R*)-**2z'**

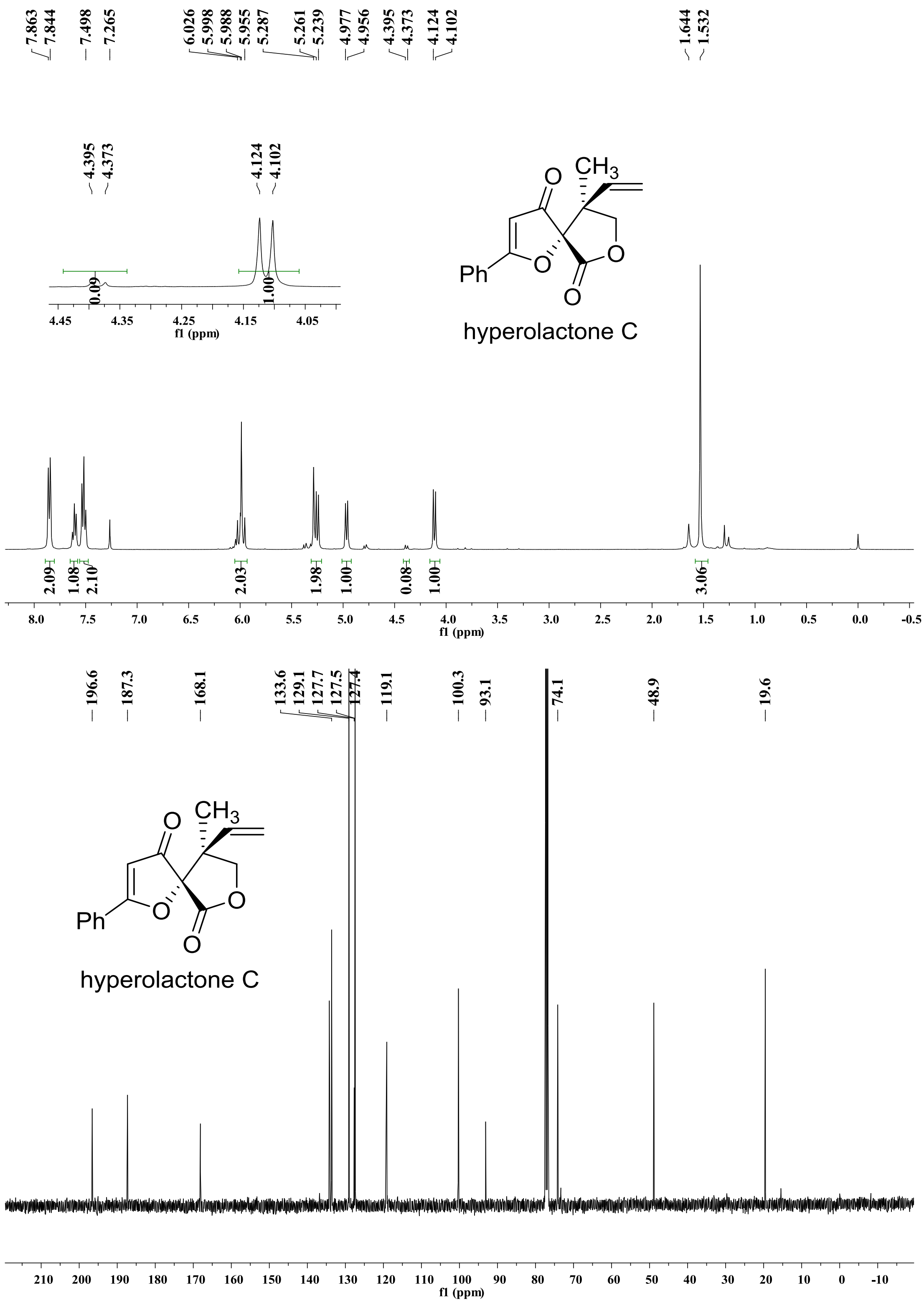

Supplementary Figure 60. ^{1}H and ^{13}C spectra for hyperolactone C

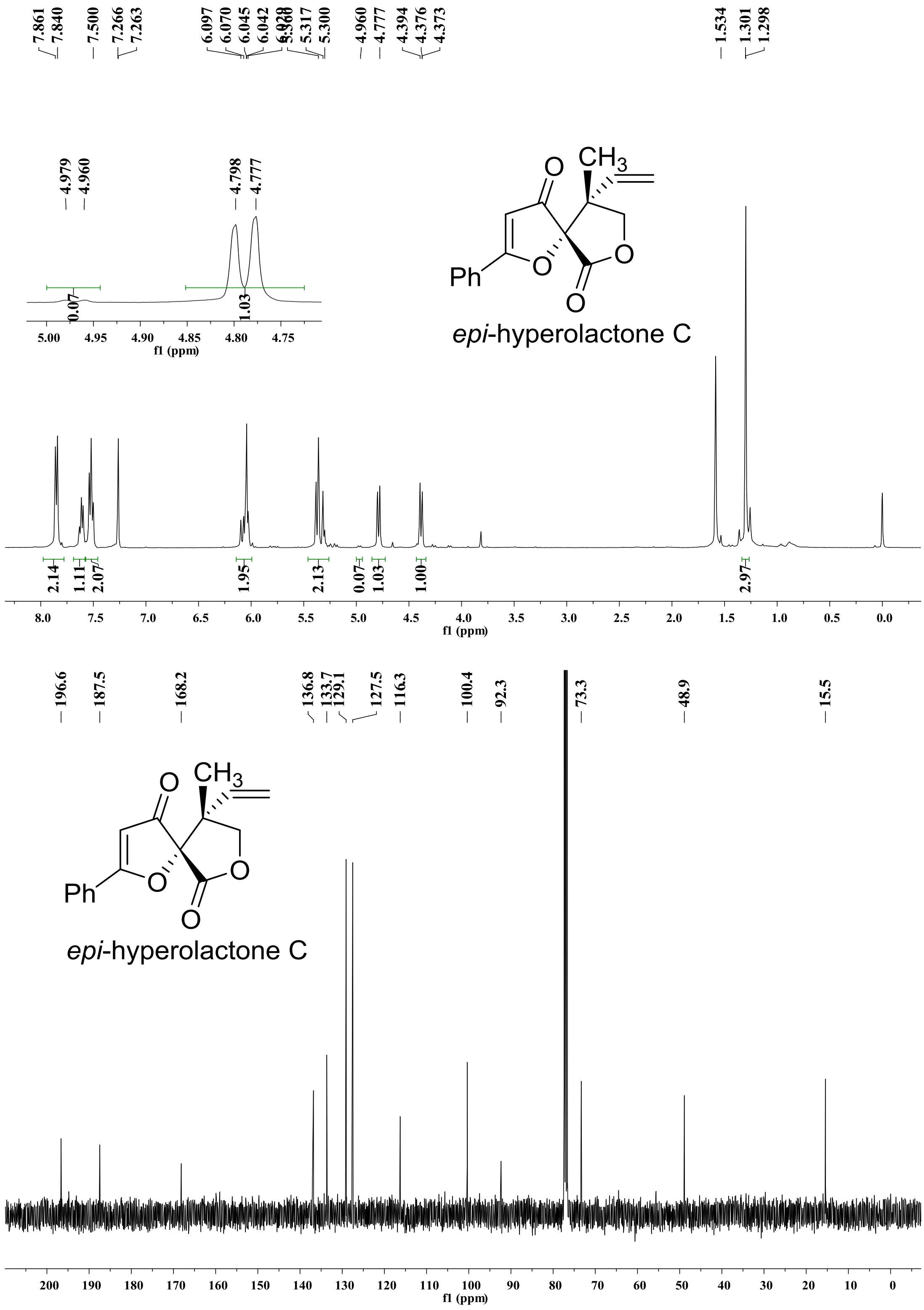

Supplementary Figure 61. ^{1}H and ^{13}C spectra for *epi*-hyperolactone C

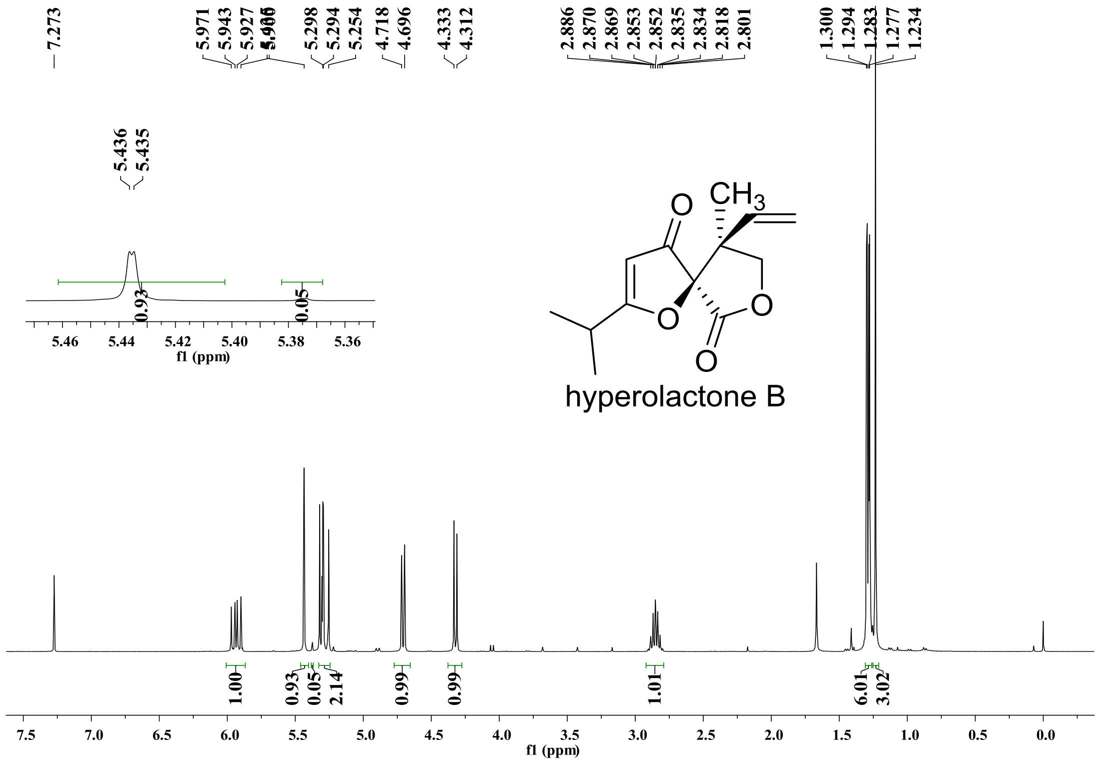

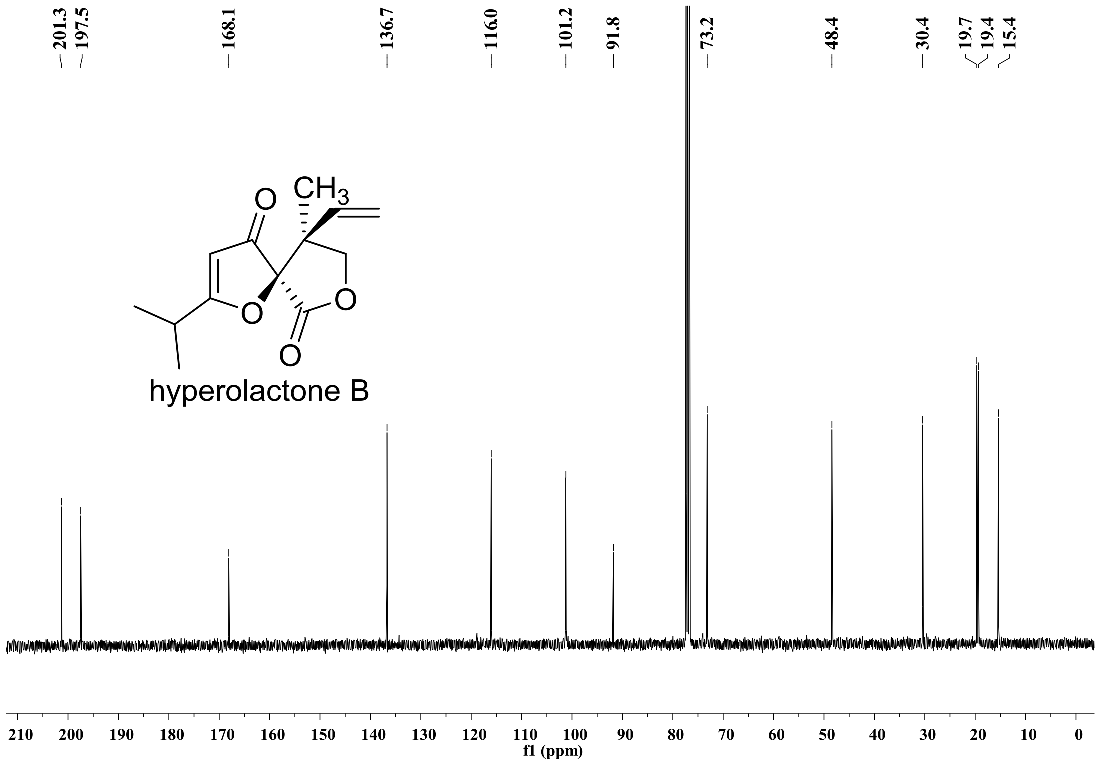

Supplementary Figure 62. ^{1}H and ^{13}C spectra for hyperolactone B

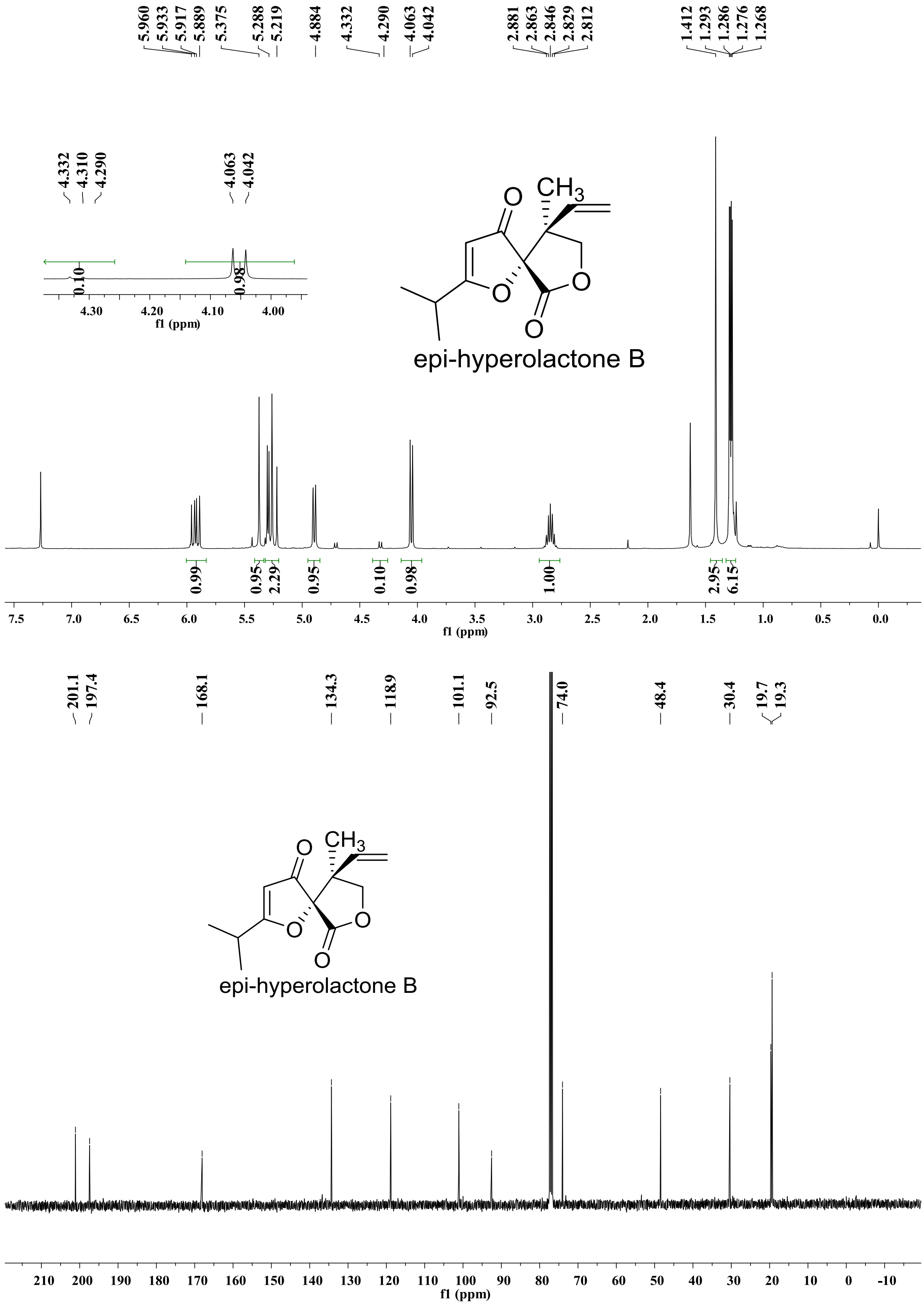

Supplementary Figure 63. ^{1}H and ^{13}C spectra for *epi*-hyperolactone B

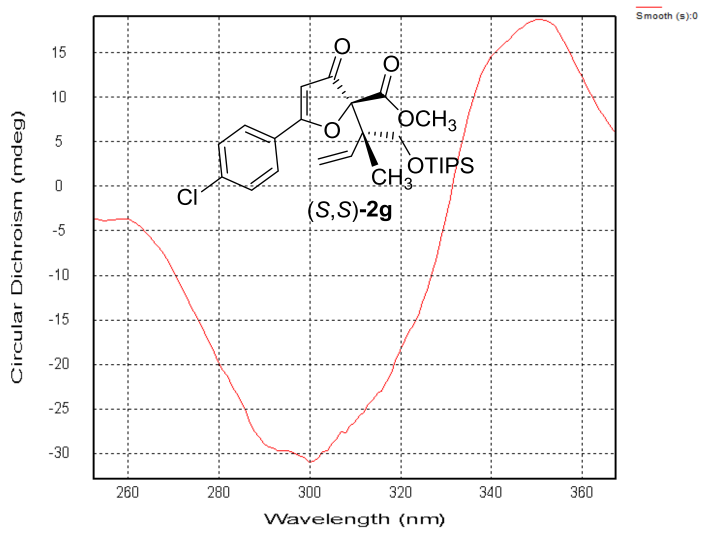

Supplementary Figure 64. CD spectra for the product (*S*,*S*)-**2g**

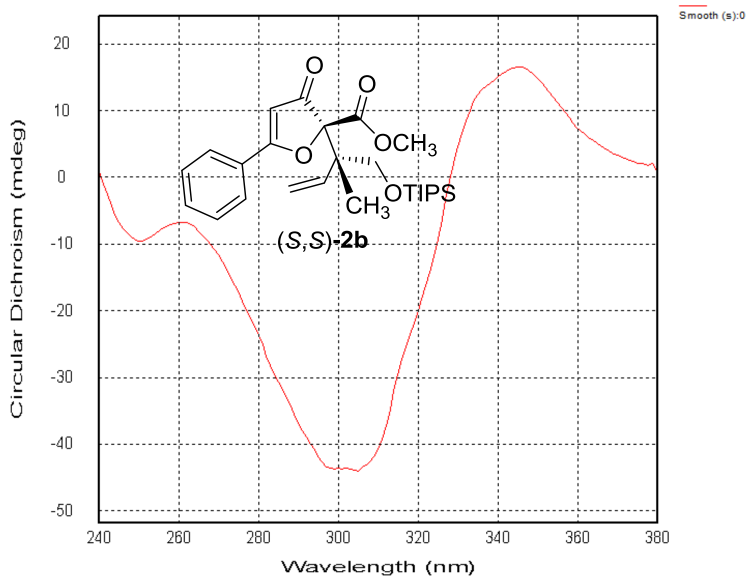

Supplementary Figure 65. CD spectra for the product (*S*,*S*)-**2b**

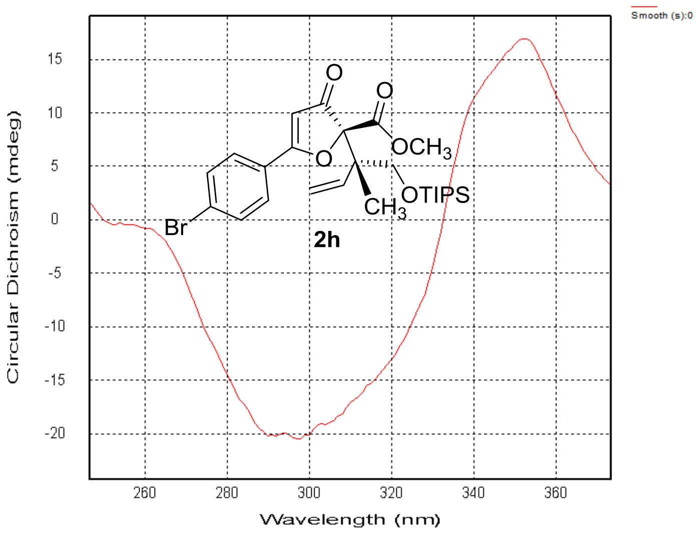

Supplementary Figure 66. CD spectra for the product **2h**

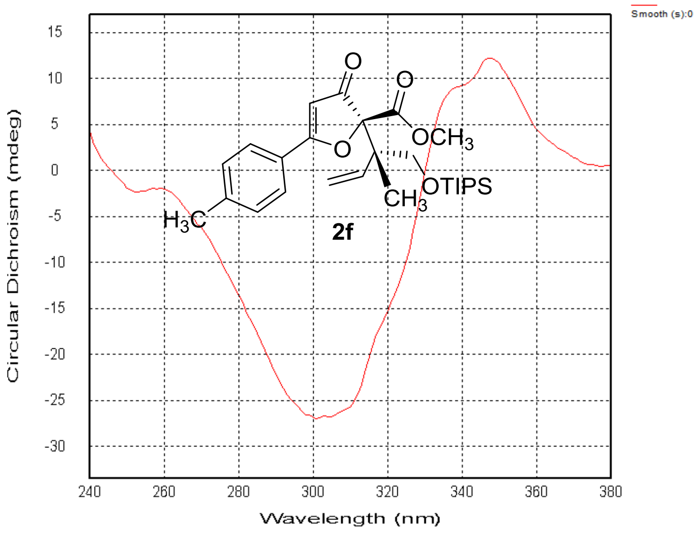

Supplementary Figure 67. CD spectra for the product **2h**

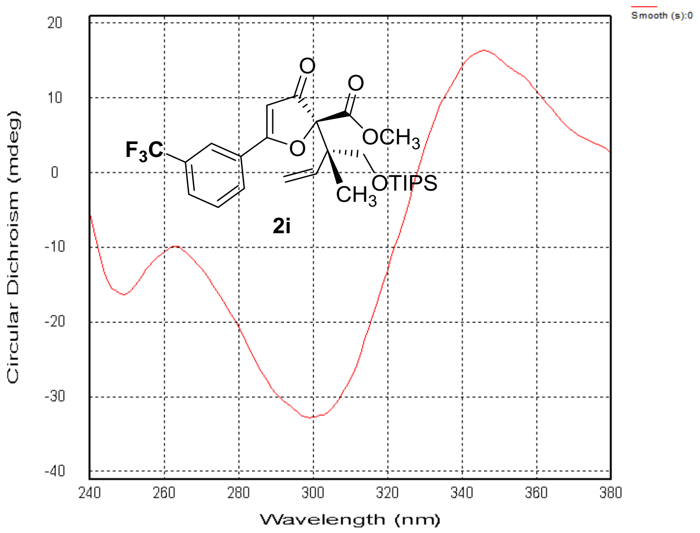

Supplementary Figure 68. CD spectra for the product **2i**

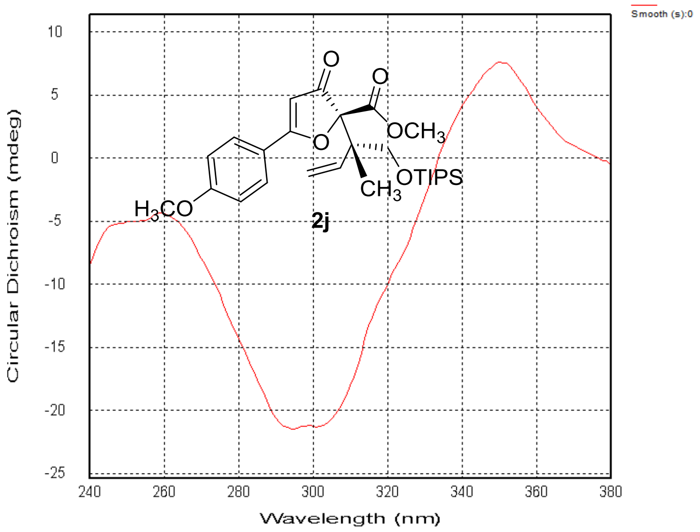

Supplementary Figure 69. CD spectra for the product **2j**

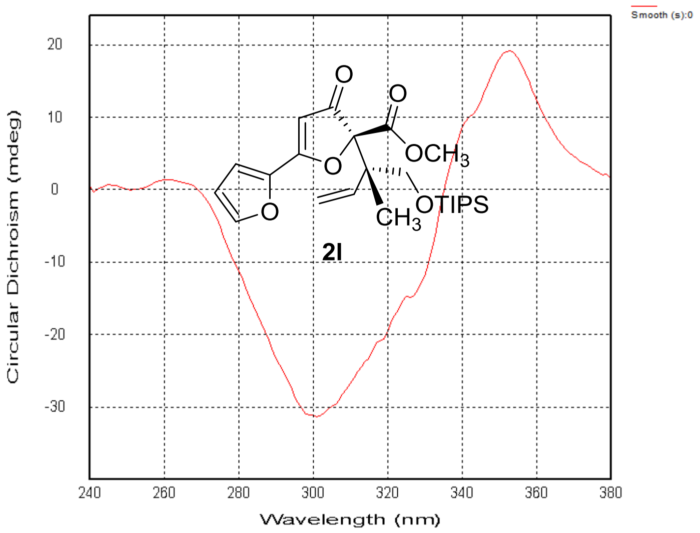

Supplementary Figure 70. CD spectra for the product **2l**

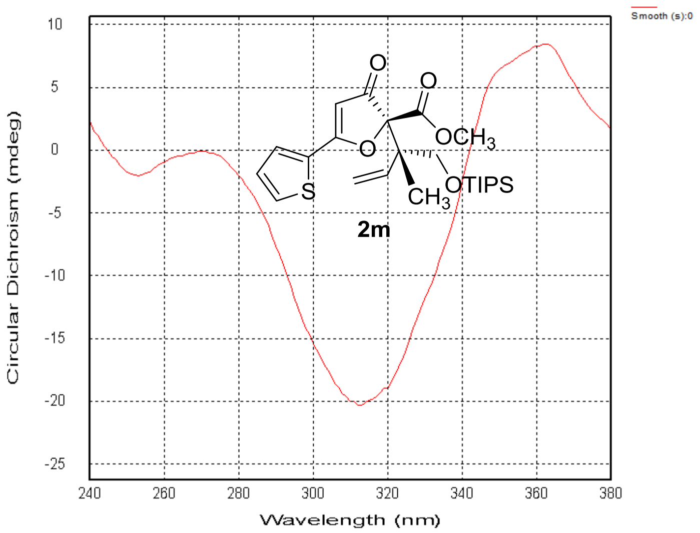

Supplementary Figure 71. CD spectra for the product **2m**

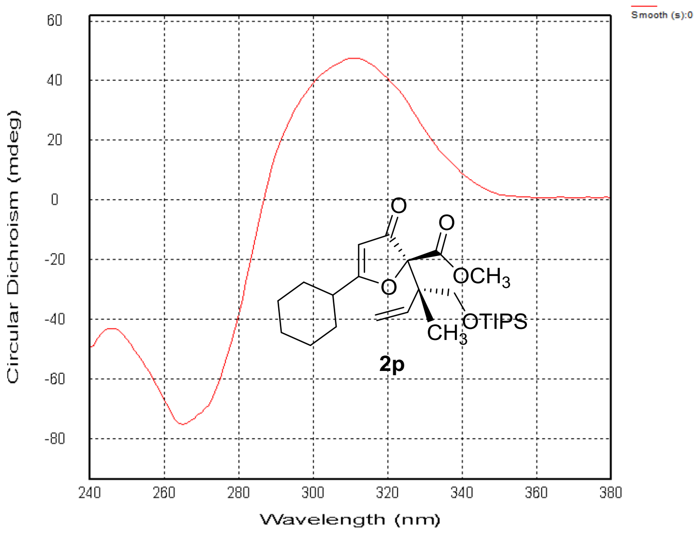

Supplementary Figure 72. CD spectra for the product **2p**

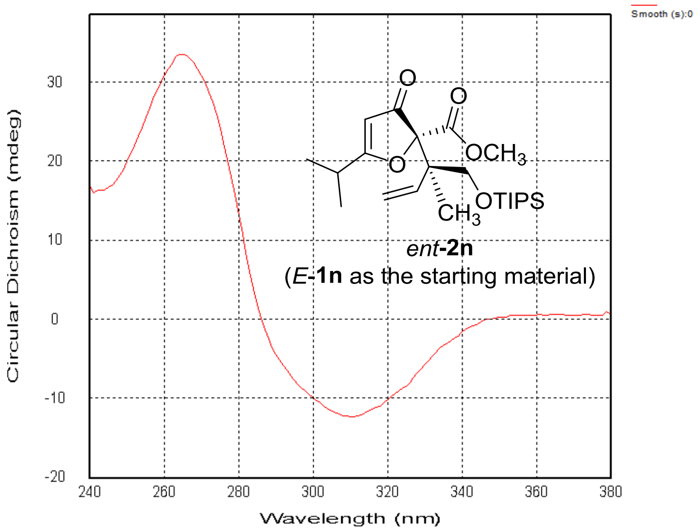

Supplementary Figure 73. CD spectra for the product ***ent*-2n**

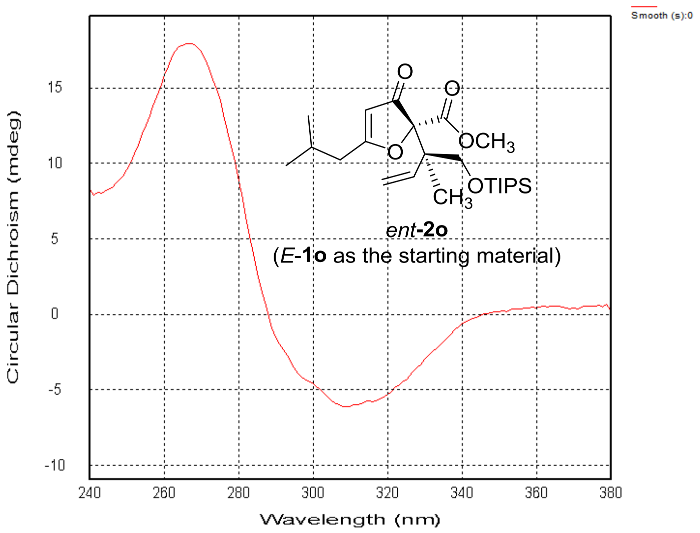

Supplementary Figure 74. CD spectra for the product *ent*-**2o**

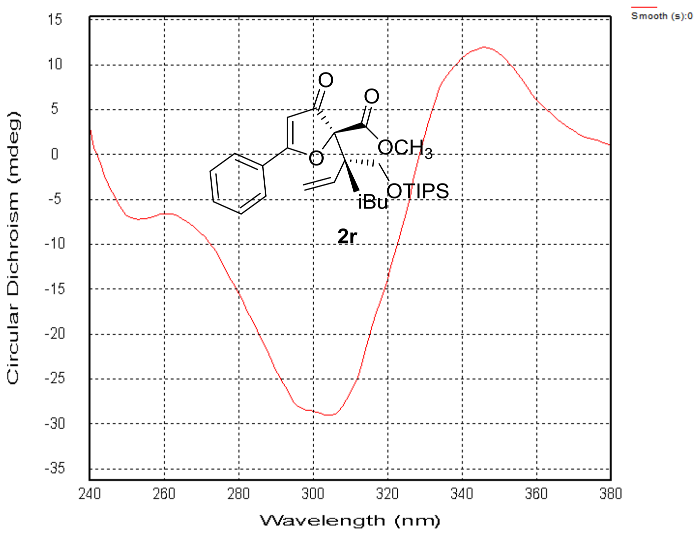

Supplementary Figure 75. CD spectra for the product **2r**

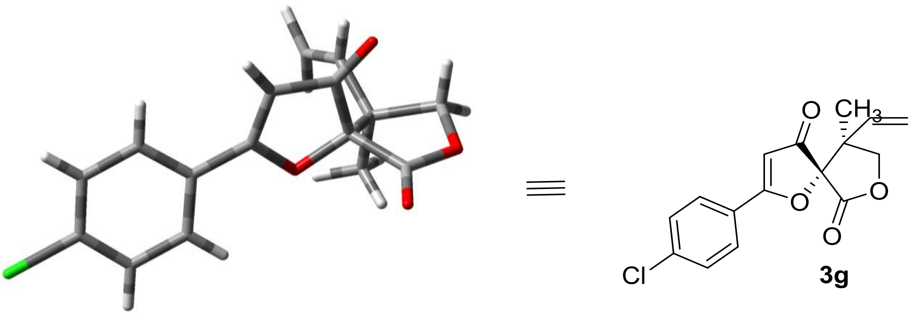

Supplementary Figure 76. X-ray crystal structure of the product **3g**

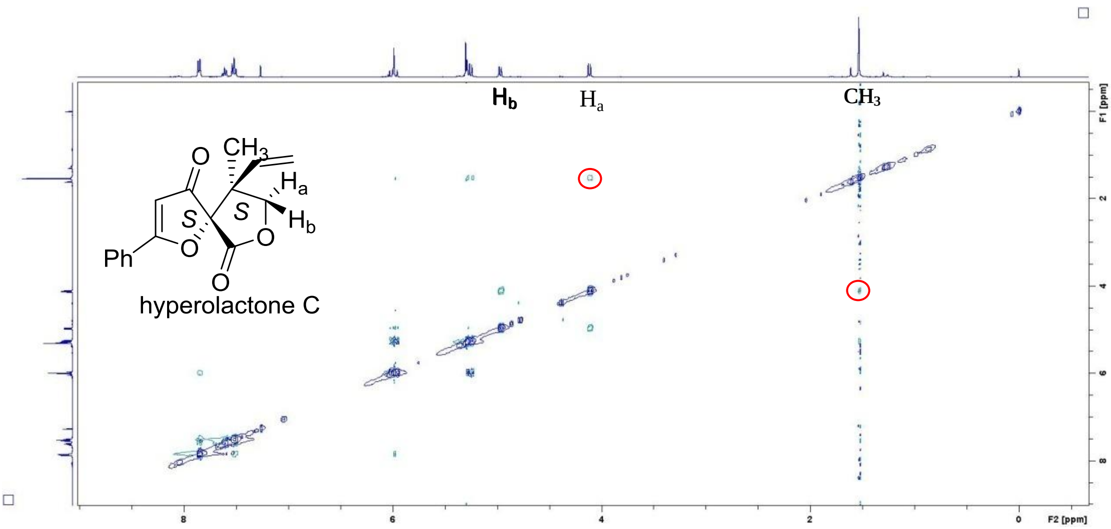

Supplementary Figure 77. NOE spectra of hyperolactone C[1]

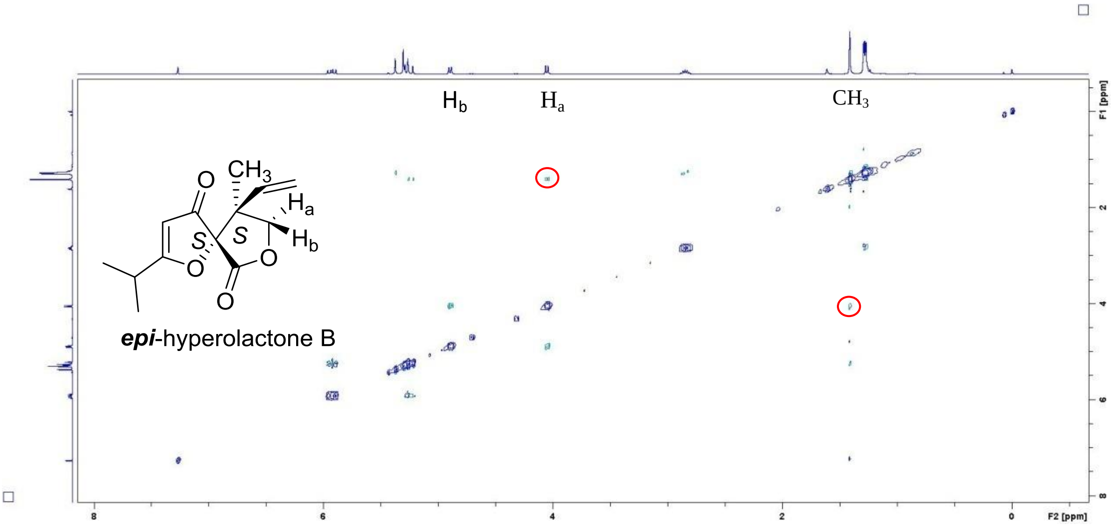

Supplementary Figure 78. NOE spectra of *epi*-hyperolactone C[1]

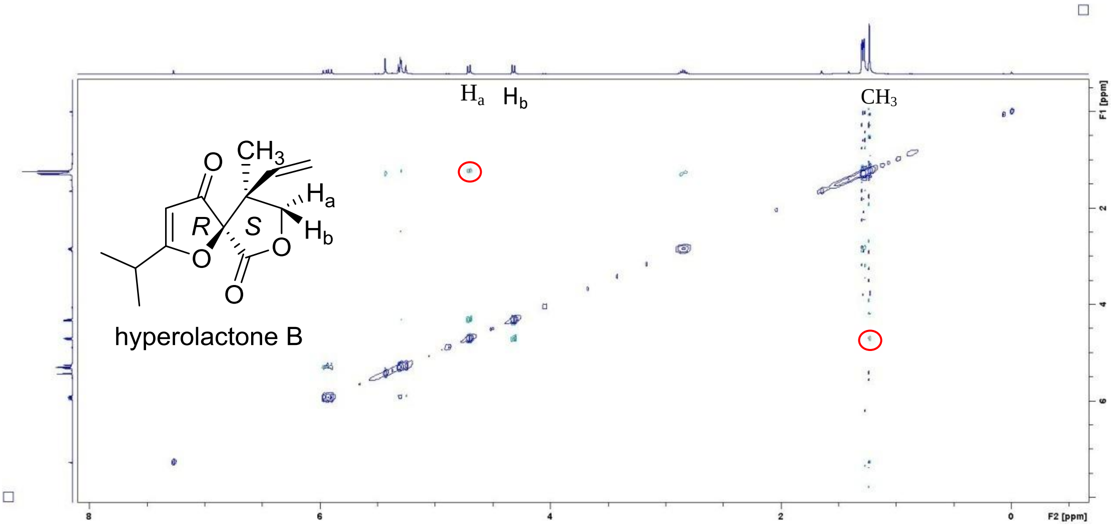

Supplementary Figure 79. NOE spectra of hyperolactone B[1]

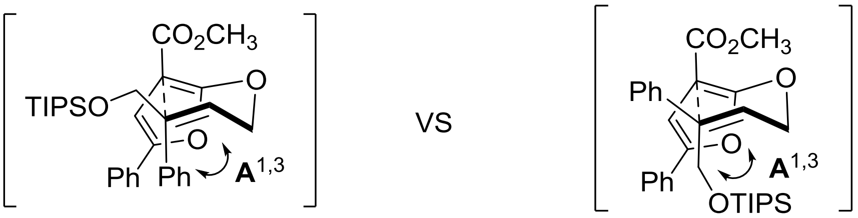

TS-1 (chair) **TS-2** (chair)

E-**2s** *Z*-**2s'**

Supplementary Figure 80. 1,3-Repulsion interaction in the reaction model for *E*-**2s** and *Z*-**2s'**

Supplementary Tables

Supplementary Table 1. Screening of the metal salts

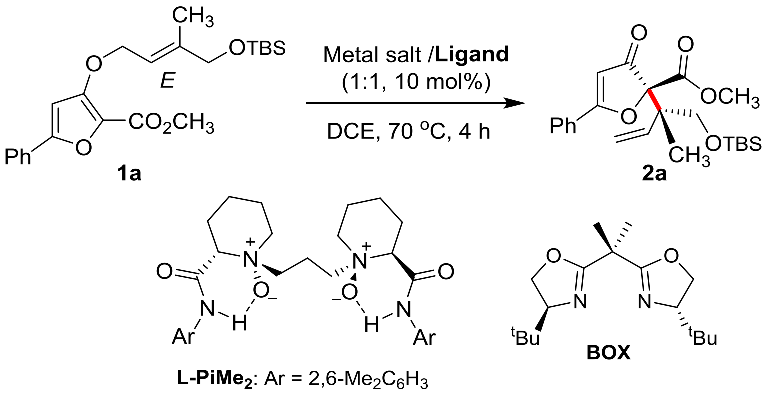

Entry[a]	Metal salt/**Ligand**	Yield [%][b]	dr[c]	Ee[%][d]
1	$Mg(OTf)_2$/**L-PiMe$_2$**	85	3:1	94
2	$Cu(OTf)_2$/**L-PiMe$_2$**	trace	-	-
3	$Yb(OTf)_3$/**L-PiMe$_2$**	47	3:1	33
4	$Ni(OTf)_2$/**L-PiMe$_2$**	85	5:1	98
5	$Cu(OTf)_2$/**BOX**	trace	-	-

[a] Unless otherwise noted, the reactions were performed with **1a** (0.1 mmol), metal/**L-PiMe$_2$** (1:1, 10 mol%), in DCE (1.0 mL) at 70 ^{o}C for 4 hours. [b] Isolated yield. [c] The dr value was determined by ^{1}H NMR and HPLC analysis. [d] Determined by HPLC analysis on chiral stationary phases.

Supplementary Table 2. Screening of the ligands

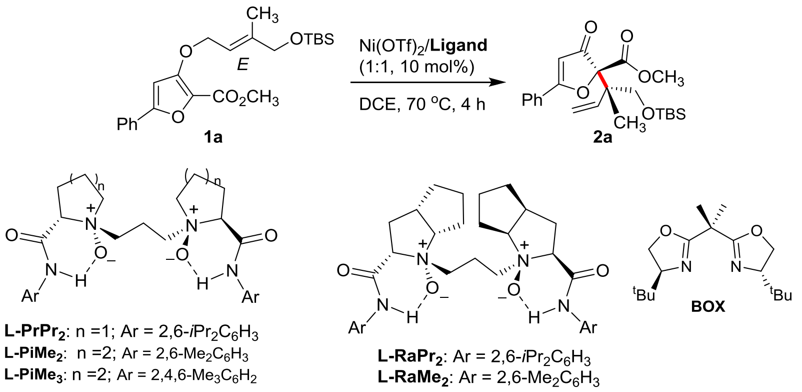

Entry[a]	Ligand	Yield [%][b]	dr[c]	Ee[%][d]
1	**L-RaPr$_2$**	60	3:2	64
2	**L-PrPr$_2$**	35	1:1	33
3	**L-RaMe$_2$**	90	2:1	94
4	**L-PiMe$_2$**	85	5:1	98
5	**L-PiMe$_3$**	85	5:1	98
6	**BOX**	trace	-	-

[a] Unless otherwise noted, the reactions were performed with **1a** (0.1 mmol), Ni(OTf)$_2$/**Ligand** (1:1, 10 mol%), in DCE (1.0 mL) at 70 °C for 4 hours. [b] Isolated yield. [c] The dr value was determined by ^{1}H NMR and HPLC analysis. [d] Determined by HPLC analysis on chiral stationary phases.

Supplementary Table 3. Screening of the solvents

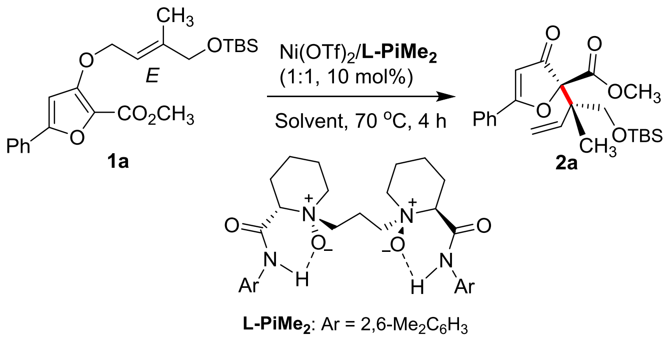

Entry[a]	Solvent	Yield [%][b]	dr[c]	Ee[%][d]
1	$CHCl_3$	80	5:1	98
2	$CHCl_2CHCl_2$	82	5:1	98
3	DCE	85	5:1	98
4	$CHCl_2CH_2Cl$	83	4:1	98

[a] Unless otherwise noted, the reactions were performed with **1a** (0.1 mmol), Ni(OTf)$_2$/**L-PiMe$_2$** (1:1, 10 mol%), in solvent (1.0 mL) at 70 °C for 4 hours. [b] Isolated yield. [c] The dr value was determined by ^{1}H NMR and HPLC analysis. [d] Determined by HPLC analysis on chiral stationary phases.

Supplementary Table 4. Screening of the silyl protecting group

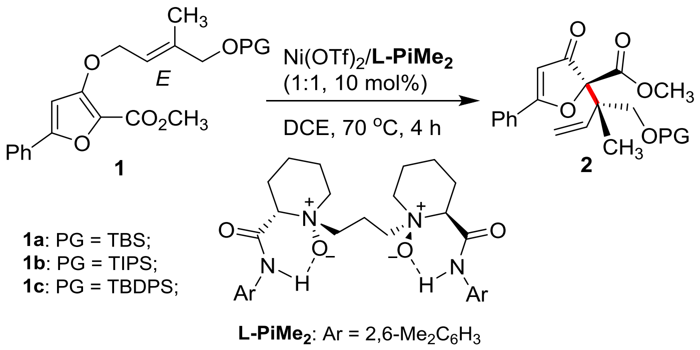

Entry[a]	PG	Yield [%][b]	dr[c]	Ee[%][d]
1	TBS	85	5:1	98
2	TIPS	90	8:1	98
3	TBDPS	90	5:1	98

[a] Unless otherwise noted, the reactions were performed with **1** (0.1 mmol), $Ni(OTf)_2$/**L-PiMe$_2$** (1:1, 10 mol%), in DCE (1.0 mL) at 70 °C for 4 hours. [b] Isolated yield. [c] The dr value was determined by ^{1}H NMR and HPLC analysis. [d] Determined by HPLC analysis on chiral stationary phases.

Supplementary Table 5. Screening of the metal counter ion

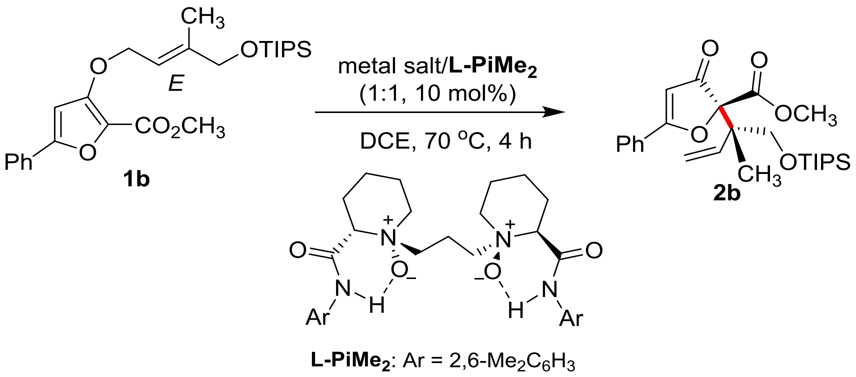

Entry[a]	Metal salt	Yield [%][b]	dr[c]	Ee[%][d]
1	$Ni(BF_4)_2{\cdot}6H_2O$	96	8:1	98
2	$Ni(OTf)_2$/NaBAr^F	75	4:1	92

[a] Unless otherwise noted, the reactions were performed with **1b** (0.1 mmol), metal/**L-PiMe$_2$** (1:1, 10 mol%), in DCE (1.0 mL) at 70 °C for 4 hours. [b] Isolated yield. [c] The dr value was determined by ^{1}H NMR and HPLC analysis. [d] Determined by HPLC analysis on chiral stationary phases.

Supplementary Table 6. Screening of the reaction temperature

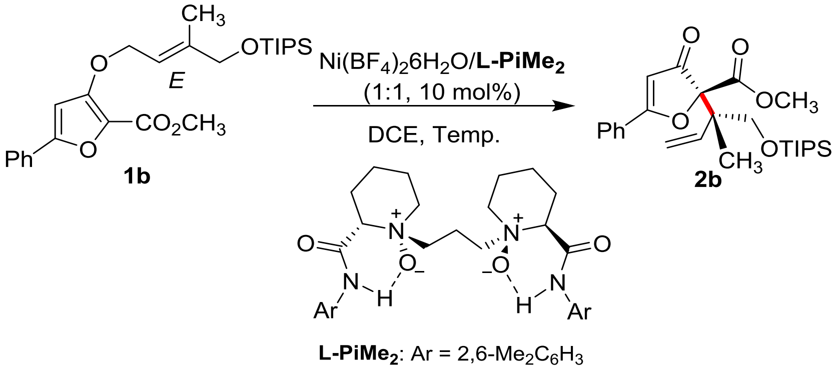

Entry[a]	Temp. (°C)	Yield [%][b]	dr[c]	Ee[%][d]
1	70	96	8:1	98
2	50	96	9:1	99
3[e]	35	96	10:1	99
4[e]	20	80	10:1	99

[a] Unless otherwise noted, the reactions were performed with **1b** (0.1 mmol), $Ni(BF_4)_2 \cdot 6H_2O$/**L-PiMe$_2$** (1:1, 10 mol%), in DCE (1.0 mL) at the indicated temperature for 4-24h. [b] Isolated yield. [c] The dr value was determined by ^{1}H NMR and HPLC analysis. [d] Determined by HPLC analysis on chiral stationary phases. [e] React at the corresponding temperature for 4 days.

Supplementary Table 7. Crystal date and structure refinement for **3g**

Empirical formula	$C_{16}H_{13}ClO_4$
Formula weight	304.71
Temperature/K	296.9(3)
Crystal system	orthorhombic
Space group	$P2_12_12_1$
a/Å	7.5541(7)
b/Å	11.5427(11)
c/Å	16.9547(12)
α/°	90
β/°	90
γ/°	90
Volume/Å^3	1478.4(2)
Z	4
ρ_{calc}g/cm^3	1.369
μ/mm^{-1}	2.409
F(000)	632.0
Crystal size/mm^3	0.75 × 0.6 × 0.4
Radiation	CuKα (λ = 1.54184)

2Θ range for data collection/°	10.436 to 145.17
Index ranges	-9 ≤ h ≤ 8, -14 ≤ k ≤ 14, -20 ≤ l ≤ 20
Reflections collected	8132
Independent reflections	2875 [R_{int} = 0.0315, R_{sigma} = 0.0235]
Data/restraints/parameters	2875/0/199
Goodness-of-fit on F^2	1.048
Final R indexes [I>=2σ (I)]	R_1 = 0.0551, wR_2 = 0.1509
Final R indexes [all data]	R_1 = 0.0592, wR_2 = 0.1605
Largest diff. peak/hole / e $Å^{-3}$	0.22/-0.28
Flack parameter	0.003(11)

Supplementary Table 8. Optimization of the deprotection/lactonization conditions

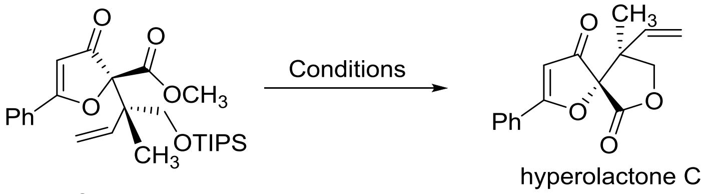

(*S*,*S*)-**2a**, 99% ee, 10:1 dr;

Entry[a]	Conditions	Yield [%][b]	dr[c]	Ee[%][d]
1	PPTS (4.0 equiv.)/DCE, 35 °C	No reaction	-	-
2	PPTS(4.0 equiv.)/DCE, 80 °C	No reaction		
3	TBAF(1 equiv.)/ THF	No reaction		
4	TBAF(10 equiv.)/ THF	Complex		
5	HF.pyridine (4 equiv.)/ CH_3CN, 35 °C	No reaction		
6	HF.pyridine (4 equiv.)/ CH_3CN, 70 °C	No reaction		
7	TMSBr(4 equiv.)/CH_3OH/DCM, 35 °C	91%	10:1	99

[a] Unless otherwise noted, the reactions were performed with (S,S)-**2a** (22.9 mg, 0.05 mmol) in Solvent at corresponding conditions, TLC analysis. [b] Isolated yield. [c] The dr value was determined by ^{1}H NMR and HPLC analysis. [d] Determined by HPLC analysis on chiral stationary phases.

Supplementary Method

General information

^{1}H NMR spectra were recorded on commercial instruments (400 MHz). Chemical shifts were reported in ppm from tetramethylsilane with the solvent resonance as the internal standard ($CDCl_3$, δ = 7.26). Spectra were reported as follows: chemical shift (δ ppm), multiplicity (s = singlet, d = doublet, t = triplet, q = quartet, m = multiplet), coupling constants (Hz), integration and assignment. ^{13}C NMR spectra were collected on commercial instruments (100 MHz) with complete proton decoupling. Chemical shifts are reported in ppm from the tetramethylsilane with the solvent resonance as internal standard ($CDCl_3$, δ = 77.0).

Enantiomeric excesses (*ee*) were determined by HPLC analysis using the corresponding commercial chiralpak column as stated in the experimental procedures at 25 ^{o}C. The chiralpak columnLux 5u Cellulose-2 was purchased from phenomenex company. Optical rotations were reported as follows: $[a]_D^{25}$ (*c*: g/100 mL, in solvent). HRMS was recorded on a commercial apparatus (ESI Source).

All catalytic reactions were run in dried glassware. THF, toluene and diethyl ether (Et_2O) were distilled from sodium. CH_2Cl_2, $ClCH_2CH_2Cl$ and HMPA were distilled over CaH_2. Cinnamyl bromide and (2, 2, 2-trifluoroethyl)-methoxycarbonylmethyl-phosphonate were purchased from Adamas-beta®.

All racemic products were obtained by using $Ni(OTf)_2$ (10 mmol%) in concert with racemic *N*,*N*'-dioxide ligand (**L-PiMe$_2$** or **L-PiMe$_3$**, 10 mmol%) at 70 ^{o}C.

General procedure for the synthesis of *E*-substrates (1a-1q)

The substituted furans (**3a-3p**) were synthesized according to the previous works[2,3].

The (*E*)-4-chloro-2-methylbut-2-en-1-ol **4** was synthesized on the basis of Rohdich's general procedure[4].

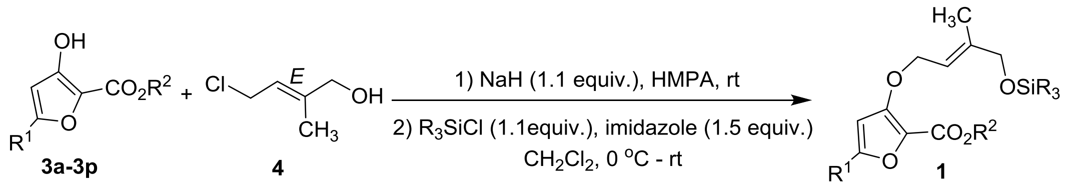

Step 1: Substituted furan (**3a-3p**, 1.0 mmol, 1.0 equiv.) was dissolved in dry HMPA (5 mL). At room temperature, NaH (26.4 mg, 1.1 mmol, 1.1 equiv.) was added. After 1 hour, (*E*)-4-chloro-2-methylbut-2-en-1-ol (**4**, 180 mg, 1.5 mmol, 1.5 equiv.) in HMPA was added in drops. The mixture was stirred at room temperature overnight. Then 10 mL of saturated aq. NH_4Cl solution was added to quench the reaction. The solution was extracted thrice with ethyl acetate, washed thrice with brine, and dried over Na_2SO_4. Evaporation of solvent and purification by flash chromatography (hexane/ethyl acetate, 2:1) afforded the mixture containing the *O*-alkylation product and the *C*-alkylation product, which were directly used in the next step.

Step 2: The mixture was dissolved in dichloromethane (10 mL) at 0 ℃, the imidazole (1.5 equiv.) and R_3SiCl (1.1 equiv.) were added in sequence. The mixture was stirred at room temperature for 5 hours. After the reaction completing, 10 mL of H_2O was added to quench the reaction. The solution was extracted thrice with dichloromethane, washed with brine, and dried over Na_2SO_4.

Evaporation of solvent and purification by flash chromatography (hexane/ethyl acetate, 20:1), the desired products (**1a-1q**) could be obtained.

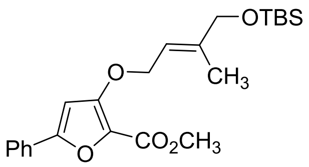

1a

Methyl-(*E*)-3-((4-((tert-butyldimethylsilyl)oxy)-3-methylbut-2-en-1-yl)oxy)-5-phenylfuran-2-carboxylate (1a): Synthesized by the general procedure. Yield reported over two steps.

Yield: 199 mg, 0.48 mmol, 48% yield (clear oil);

^{1}H NMR (400 MHz, $CDCl_3$) δ 7.69 (d, *J* = 7.2 Hz, 2H), 7.36 – 7.29 (m, 3H), 6.54 (s, 1H), 5.82 – 5.68 (m, 1H), 4.71 (d, *J* = 6.4 Hz, 2H), 4.01 (s, 2H), 3.84 (s, 3H), 1.68 (s, 3H), 0.84 (s, 9H), 0.00 (s, 6H);

^{13}C NMR (101 MHz, $CDCl_3$) δ 159.3, 155.5, 155.4, 140.5, 129.3, 128.8, 128.0, 124.8, 118.1, 98.1, 68.4, 67.4, 51.4, 25.9, 18.4, 13.9, 5.4;

HRMS (ESI) calcd for $[M+H]^+$: $C_{23}H_{33}O_5Si$, m/z: 417.2091, observed: 417.2095.

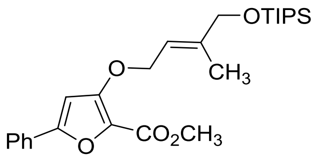

1b

Methyl-(*E*)-3-((3-methyl-4-((triisopropylsilyl)oxy)but-2-en-1-yl)oxy)-5-phenylfuran-2-carboxylate (**1b**); Synthesized by the general procedure. Yield reported over two steps.

Yield: 206 mg, 0.45 mmol, 45% yield (clear oil)

^{1}H NMR (400 MHz, $CDCl_3$) δ 7.66 (d, *J* = 7.2 Hz, 2H), 7.34 – 7.25 (m, 3H), 6.52 (s, 1H), 5.78 (dd, *J* = 6.8, 5.6 Hz, 1H), 4.71 (d, *J* = 6.4 Hz, 2H), 4.06 (s, 2H), 3.82 (s, 3H), 1.66 (s, 3H), 1.04 – 0.90 (m, 21H);

^{13}C NMR (101 MHz, $CDCl_3$) δ 159.4, 155.5, 155.3, 140.6, 129.4, 129.3, 128.8, 128.1, 124.8, 117.8, 98.1, 68.3, 67.4, 51.4, 18.0, 13.9, 12.0;

HRMS (ESI) calcd for $[M+H]^+$: $C_{26}H_{39}O_5Si$, m/z: 459.2567, observed: 459.2571;

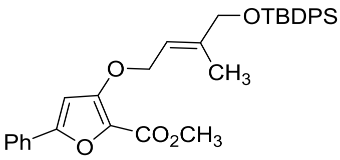

1c

Methyl-(*E*)-3-((4-((tert-butyldiphenylsilyl)oxy)-3-methylbut-2-en-1-yl)oxy)-5-phenylfuran-2-carboxylate (**1c**) ; Synthesized by the general procedure. Yield reported over two steps.

Yield: 216 mg, 0.40 mmol, 40% yield (clear oil);

^{1}H NMR (400 MHz, $CDCl_3$) δ 7.74 (d, *J* = 7.2 Hz, 2H), 7.69 – 7.59 (m, 4H), 7.42 – 7.32 (m, 9H), 6.60 (s, 1H), 5.92 (t, *J* = 6.0 Hz, 1H), 4.79 (d, *J* = 6.0 Hz, 2H), 4.09 (s, 2H), 3.91 (s, 3H), 1.72 (s, 3H), 1.04 (s, 9H).

^{13}C NMR (101 MHz, $CDCl_3$) δ 159.4, 155.5, 155.3, 140.2, 135.5, 133.4, 129.7, 129.3, 128.8, 128.1, 127.7, 124.9, 118.2, 98.1, 68.4, 67.8, 51.4, 26.8, 19.3, 14.0;

HRMS (ESI) calcd for $[M+Na]^+$: $C_{33}H_{36}O_5SiNa$, m/z: 563.2224, observed: 563.2226;

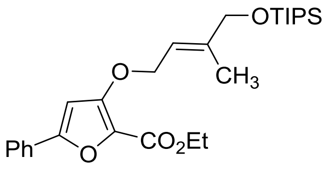

1d

Ethyl-(*E*)-3-((3-methyl-4-((triisopropylsilyl)oxy)but-2-en-1-yl)oxy)-5-phenylfuran-2-carboxylate (**1d**); Synthesized by the general procedure. Yield reported over two steps.

Yield: 198 mg, 0.42 mmol, 42% yield (clear oil)

^{1}H NMR (400 MHz, $CDCl_3$) δ 7.74 – 7.72 (m, 2H), 7.40 – 7.28 (m, 3H),6.60 (s, 1H), 5.86 (t, *J* = 6.4 Hz, 1H), 4.76 (d, *J* = 6.4 Hz, 2H), 4.36 (dd, *J* = 7.2, 14.4 Hz, 2H), 4.13 (s, 2H), 1.73 (s, 3H), 1.37 (t, *J* = 7.2 Hz, 3H), 1.14 – 1.03 (m, 21H);

^{13}C NMR (101 MHz, $CDCl_3$) δ 159.0, 155.2, 140.4, 129.4, 129.1, 128.7, 128.3, 124.7, 117.8, 98.2, 68.3, 67.4, 60.2, 18.0, 14.5, 13.8, 12.0;

HRMS (ESI) calcd for $[M+H]^+$: $C_{27}H_{41}O_5Si$, m/z: 473.2717, observed: 473.2727.

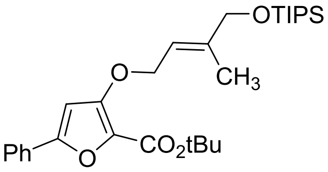

1e

Tert-butyl-(*E*)-3-((3-methyl-4-((triisopropylsilyl)oxy)but-2-en-1-yl)oxy)-5-phenylfuran-2-carboxylate (**1e**); Synthesized by the general procedure. Yield reported over two steps.

Yield: 200 mg, 0.4 mmol, 40% yield (clear oil)

^{1}H NMR (400 MHz, $CDCl_3$) δ 7.73 (d, *J* = 7.2 Hz, 2H), 7.41 – 7.29 (m, 3H), 6.58 (s, 1H), 5.86 (t, *J* = 6.4Hz, 1H), 4.75 (d, *J* = 6.4 Hz, 2H), 4.14 (s, 2H), 1.73 (s, 3H), 1.60 (s, 9H), 1.11 – 1.07 (m, 3H), 1.05 – 1.02 (m, 18H);

^{13}C NMR (101 MHz, $CDCl_3$) δ 158.6, 154.6, 154.5, 140.2, 129.6, 129.0, 128.7, 124.7, 117.9, 98.1, 81.1, 68.3, 67.4, 28.5, 18.0, 13.9, 12.0;

HRMS (ESI) calcd for $[M+H]^+$: $C_{29}H_{45}O_5Si$, m/z: 501.3030, observed: 501.3027.

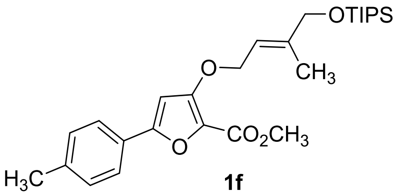

1f

Methyl-(*E*)-3-((3-methyl-4-((triisopropylsilyl)oxy)but-2-en-1-yl)oxy)-5-(p-tolyl)furan-2-carboxylate (**1f**); Synthesized by the general procedure. Yield reported over two steps.

Yield: 217 mg, 0.46 mmol, 46% yield (clear oil)

^{1}H NMR (400 MHz, $CDCl_3$) δ 7.63 (d, *J* = 6.8 Hz, 2H), 7.20 (d, *J* = 7.6 Hz, 2H), 6.54 (d, *J* = 1.6 Hz, 1H), 5.91 – 5.81 (m, 1H), 4.77 (d, *J* = 6.4 Hz, 2H), 4.14 (s, 2H), 3.89 (d, *J* = 1.2 Hz, 3H), 2.37 (s, 3H), 1.73 (s, 3H), 1.08 – 0.94 (m, 21H);

^{13}C NMR (101 MHz, $CDCl_3$) δ 159.4, 155.8, 155.5, 140.5, 139.4, 129.4, 127.7, 126.7, 124.8, 117.8, 97.5, 68.3, 67.5, 51.3, 21.4, 18.0, 13.9, 12.0;

HRMS (ESI) calcd for $[M+H]^+$: $C_{27}H_{41}O_5Si$, m/z: 473.2717, observed: 473.2726.

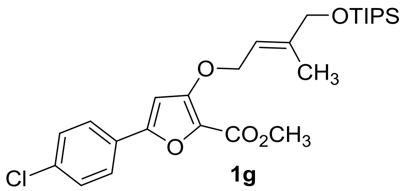

1g

Methyl-(*E*)-5-(4-chlorophenyl)-3-((3-methyl-4-((triisopropylsilyl)oxy)but-2-en-1-yl)oxy)furan-2-carboxylate (**1g**); Synthesized by the general procedure. Yield reported over two steps.

Yield: 212 mg, 0.43 mmol, 43% yield (clear oil)

^{1}H NMR (400 MHz, $CDCl_3$) δ 7.70 – 7.64 (m, 2H), 7.45 – 7.33 (m, 2H), 6.58 (s, 1H), 5.91 – 5.79 (m, 1H), 4.78 (d, *J* = 6.4 Hz, 2H), 4.14 (s, 2H), 3.89 (s, 3H), 1.74 (s, 3H), 1.10 – 1.00 (m, 21H);

^{13}C NMR (101 MHz, $CDCl_3$) δ 159.2, 155.2, 154.2, 140.7, 135.1, 129.0, 128.3, 127.8, 126.0, 117.6, 98.5, 68.4, 67.4, 51.4, 18.0, 13.9, 12.0;

HRMS (ESI) calcd for $[M+H]^+$: $C_{26}H_{38}ClO_5Si$, m/z: 493.2172, 494.2205, observed: 493.2172, 494.2199.

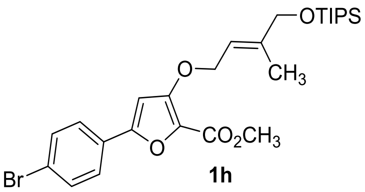

1h

Methyl-(*E*)-5-(4-bromophenyl)-3-((3-methyl-4-((triisopropylsilyl)oxy)but-2-en-1-yl)oxy)furan-2-carboxylate (1h); Synthesized by the general procedure. Yield reported over two steps.

Yield: 231 mg, 0.43 mmol, 43% yield (clear oil)

^{1}H NMR (400 MHz, $CDCl_3$) δ 7.60 (d, *J* = 8.4 Hz, 2H), 7.54 (d, *J* = 8.4 Hz, 2H), 6.59 (s, 1H), 5.85 (dd, *J* = 6.4, 5.2 Hz, 1H), 4.78 (d, *J* = 6.8 Hz, 2H), 4.14 (s, 2H), 3.89 (s, 3H), 1.73 (s, 3H), 1.10 – 1.00 (m, 21H);

^{13}C NMR (101 MHz, $CDCl_3$) δ 159.3, 155.1, 154.2, 140.7, 132.0, 128.3, 126.2, 123.4, 117.6, 98.6, 68.4, 67.4, 51.4, 18.0, 13.9, 12.0;

HRMS (ESI) calcd for $[M+H]^+$: $C_{26}H_{38}BrO_5Si$, m/z: 537.1666, 539.1646, observed: 537.1672, 539.1653.

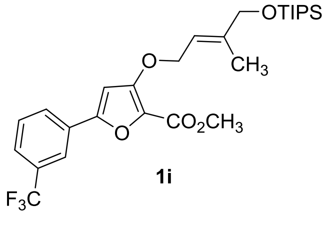

1i

Methyl-(*E*)-3-((3-methyl-4-((triisopropylsilyl)oxy)but-2-en-1-yl)oxy)-5-(3-(trifluoromethyl)phenyl)furan-2-carboxylate (**1l**); Synthesized by the general procedure. Yield reported over two steps.

Yield: 210 mg, 0.4 mmol, 40% yield (clear oil)

^{1}H NMR (400 MHz, $CDCl_3$) δ 7.95 (s, 1H), 7.91 (d, *J* = 7.6 Hz, 1H), 7.64 – 7.48 (m, 2H), 6.71 (s, 1H), 5.88 (s, 1H), 4.81 (d, *J* = 6.4 Hz, 2H), 4.15 (s, 2H), 3.91 (s, 3H), 1.74 (s, 3H), 1.15 – 0.99 (m, 21H);

^{13}C NMR (101 MHz, $CDCl_3$) δ 159.1, 155.0, 153.5, 140.8, 131.3 (q, J = 33 Hz), 130.1, 129.3, 128.7, 127.8, 125.5 (q, J = 4 Hz), 123.8 (q, J = 270 Hz), 121.4 (q, J = 4 Hz), 117.4, 99.3, 68.4, 67.3, 51.4, 17.9, 13.8, 11.9;

HRMS (ESI) calcd for $[M+H]^+$: $C_{27}H_{38}F_3O_5Si$, m/z: 527.2435, 528.2469, observed: 527.2435,

528.2462.

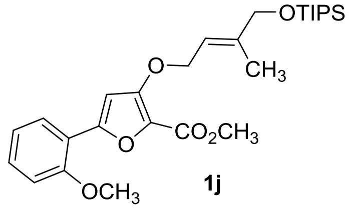

1j

Methyl-(*E*)-5-(2-methoxyphenyl)-3-((3-methyl-4-((triisopropylsilyl)oxy)but-2-en-1-yl)oxy)furan-2-carboxylate (**1j**); Synthesized by the general procedure. Yield reported over two steps.

Yield: 229 mg, 0.47 mmol, 47% yield (clear oil)

^{1}H NMR (400 MHz, $CDCl_3$) δ 7.32– 7.30 (m, 2H), 7.28 – 7.25 (m, 1H), 6.96 – 6.86 (m, 1H), 6.59–6.58 (m, 1H), 5.87 – 5.84 (m, 1H), 4.78 (d, *J* = 6.4 Hz, 2H), 4.14 (s, 2H), 3.90 (d, *J* = 1.4 Hz, 3H), 3.86 (d, *J* = 1.4 Hz, 3H), 1.74 (s, 3H), 1.10 – 0.99 (m, 21H);

^{13}C NMR (101 MHz, $CDCl_3$) δ 159.9, 159.4, 155.3, 140.6, 130.6, 129.8, 128.1, 117.7, 117.4, 115.3, 109.9, 98.4, 68.4, 67.4, 55.4, 51.4, 18.0, 13.9, 12.0;

HRMS (ESI) calcd for $[M+H]^+$: $C_{27}H_{41}O_6Si$, m/z: 489.2667, observed: 489.2662.

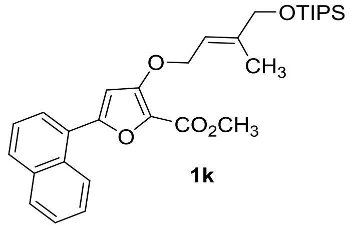

1k

Methyl-(*E*)-3-((3-methyl-4-((triisopropylsilyl)oxy)but-2-en-1-yl)oxy)-5-(naphthalen-1-yl)furan-2-carboxylate (**1k**); Synthesized by the general procedure. Yield reported over two steps.

Yield: 233mg, 0.46 mmol, 46% yield (clear oil)

^{1}H NMR (400 MHz, $CDCl_3$) δ 8.37 (d, *J* = 8.4 Hz, 1H), 7.84 (d, *J* = 8.4 Hz, 2H), 7.76 (d, *J* = 7.2 Hz, 1H), 7.58 – 7.41 (m, 3H), 6.65 (s, 1H), 5.89 (dd, *J* = 6.4, 5.2 Hz, 1H), 4.81 (d, *J* = 6.4 Hz, 2H), 4.13 (s, 2H), 3.90 (s, 3H), 1.73 (s, 3H), 1.10 – 0.98 (m, 21H);

^{13}C NMR (101 MHz, $CDCl_3$) δ 159.3, 155.5, 154.9, 140.6, 133.9, 130.2, 130.1, 128.7, 128.5, 127.2, 127.1, 126.2, 125.1, 125.0, 117.9, 102.5, 68.4, 67.4, 51.4, 18.0, 13.9, 12.0;

HRMS (ESI) calcd for $[M+H]^+$: $C_{30}H_{41}O_5Si$, m/z: 525.2667, observed: 525.2667.

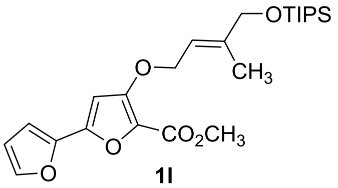

1l

Methyl-(*E*)-4-((3-methyl-4-((triisopropylsilyl)oxy)but-2-en-1-yl)oxy)-[2,2'-bifuran]-5-carboxylate (1f); Synthesized by the general procedure. Yield reported over two steps.

Yield: 170 mg, 0.38 mmol, 38% yield (clear oil)

^{1}H NMR (400 MHz, $CDCl_3$) δ 7.37 (s, 1H), 6.80 – 6.68 (m, 1H), 6.45 – 6.36 (m, 2H), 5.81 – 5.71 (m, 1H), 4.69 (d, *J* = 6.4 Hz, 2H), 4.05 (s, 2H), 3.80 (d, *J* = 1.6 Hz, 3H), 1.64 (s, 3H), 1.02 – 0.90 (m, 21H);

^{13}C NMR (101 MHz, $CDCl_3$) δ 159.2, 155.0, 147.8, 145.2, 143.3, 140.7, 127.6, 117.6, 111.9, 108.7, 97.9, 68.4, 67.4, 51.4, 17.9, 13.8, 12.0;

HRMS (ESI) calcd for $[M+H]^+$: $C_{24}H_{37}O_6Si$, m/z: 449.2354, observed: 449.2361.

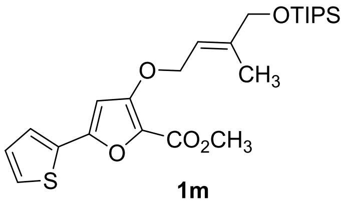

1m

Methyl-(*E*)-3-((3-methyl-4-((triisopropylsilyl)oxy)but-2-en-1-yl)oxy)-5-(thiophen-2-yl)furan-2-carboxylate (**1m**); Synthesized by the general procedure. Yield reported over two steps.

Yield: 181 mg, 0.39 mmol, 39% yield (clear oil)

^{1}H NMR (400 MHz, $CDCl_3$) δ 7.33 (dd, *J* = 3.6, 0.8 Hz, 1H), 7.24 (dd, *J* = 5.2, 1.2 Hz, 1H), 6.95 (dd, *J* = 5.2, 4.0 Hz, 1H), 6.35 (s, 1H), 5.78 – 5.72 (m, 1H), 4.66 (d, *J* = 6.8 Hz, 2H), 4.04 (s, 2H), 3.78 (s, 3H), 1.63 (s, 3H), 1.01 – 0.88 (m, 21H);

^{13}C NMR (101 MHz, $CDCl_3$) δ 159.1, 155.2, 151.0, 140.7, 132.2, 127.9, 127.5, 126.7, 125.4, 117.6, 98.0, 68.3, 67.4, 51.3, 17.9, 13.8, 12.0;

HRMS (ESI) calcd for $[M+H]^+$: $C_{24}H_{37}O_5SiS$, m/z: 465.2125, observed: 465.2123.

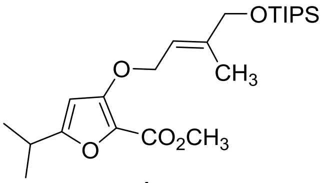

1n

Methyl-(E)-5-isopropyl-3-((3-methyl-4-((triisopropylsilyl)oxy)but-2-en-1-yl)oxy)furan-2-carboxylate (1n); Synthesized by the general procedure. Yield reported over two steps.

Yield: 144 mg, 0.35 mmol, 35% yield (clear oil)

^{1}H NMR (400 MHz, $CDCl_3$) δ 6.00 (s, 1H), 5.82 (t, *J* = 6.0 Hz, 1H), 4.71 (d, *J* = 6.4 Hz, 2H), 4.13 (s, 2H), 3.85 (s, 3H), 3.02 – 2.86 (m, 1H), 1.70 (s, 3H), 1.25 (d, *J* = 7.2 Hz, 6H), 1.10 - 1.01 (m, 21H);

^{13}C NMR (101 MHz, $CDCl_3$) δ 165.1, 159.4, 155.0, 140.0, 126.9, 117.8, 97.4, 68.1, 67.4, 51.2, 28.6, 20.7, 18.0, 13.8, 12.0;

HRMS (ESI) calcd for $[M+H]^+$: $C_{23}H_{41}O_5Si$, m/z: 425.2718, observed: 425.2717.

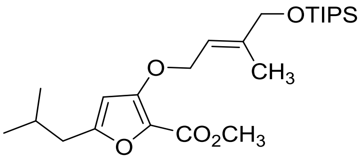

1o

Methyl-(E)-5-isobutyl-3-((3-methyl-4-((triisopropylsilyl)oxy)but-2-en-1-yl)oxy)furan-2-carboxylate (**1o**); Synthesized by the general procedure. Yield reported over two steps.

Yield: 149 mg, 0.34 mmol, 34% yield (clear oil)

^{1}H NMR (400 MHz, $CDCl_3$) δ 6.01 (s, 1H), 5.82 – 5.78 (m, 1H), 4.70 (d, *J* = 6.4 Hz, 2H), 4.13 (s, 2H), 3.85 (s, 3H), 2.47 (d, *J* = 7.2 Hz, 2H), 2.08 – 1.95 (m, 1H), 1.70 (s, 3H), 1.06 – 1.01 (m, 21H), 0.93 (d, *J* = 6.4 Hz, 6H);

^{13}C NMR (101 MHz, $CDCl_3$) δ 159.6, 155.2, 140.5, 127.5, 118.1, 100.5, 68.4, 67.7, 53.7, 38.2, 27.9, 22.55, 18.3, 14.1, 12.2;

HRMS (ESI) calcd for $[M+H]^+$: $C_{24}H_{43}O_5Si$, m/z: 439.2874, observed: 439.2869.

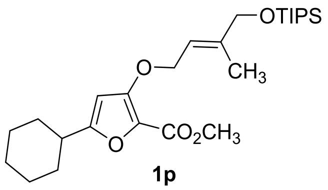

Methyl-(E)-5-cyclohexyl-3-((3-methyl-4-((triisopropylsilyl)oxy)but-2-en-1-yl)oxy)furan-2-carboxylate (**1p**); Synthesized by the general procedure. Yield reported over two steps.

Yield: 148.5 mg, 0.32 mmol, 32% yield (clear oil)

^{1}H NMR (400 MHz, $CDCl_3$) δ 5.90 (s, 1H), 5.73 (t, *J* = 6.0 Hz, 1H), 4.62 (d, *J* = 6.4 Hz, 2H), 4.05 (s, 2H), 3.77 (s, 3H), 2.53 (s, 1H), 1.95 (d, *J* = 7.6 Hz, 2H), 1.80 – 1.64 (m, 4H), 1.63 (s, 3H), 1.28 (t, *J* = 10.8 Hz, 4H), 1.09 – 0.98 (m, 21H);

^{13}C NMR (101 MHz, $CDCl_3$) δ 164.3, 159.4, 155.0, 140.2, 126.8, 117.9, 97.4, 68.1, 67.4, 51.2, 37.9, 31.0, 25.8, 25.7, 18.0, 13.8, 12.0;

HRMS (ESI) calcd for $[M+H]^+$: $C_{26}H_{45}O_5Si$, m/z: 465.3031, observed: 465.3041.

General procedure for the synthesis of (*E*)-substrates (1q- 1r)

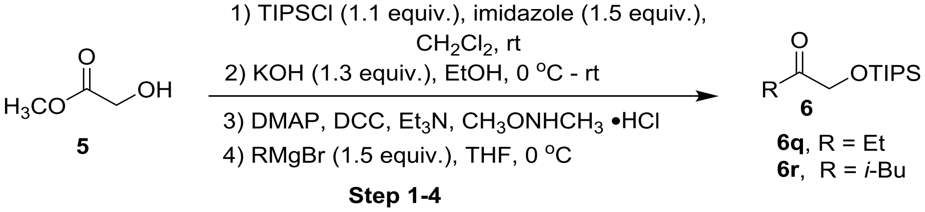

On the basis of the previous reports[5-7], the alkyl-substituted ketones could be synthesized in four steps. As follow:

Step 1: The ethyl glycolate **5** (9 g, 100 mmol) and imidazole (6.81 g, 150 mmol, 1.5 equiv.) were dissolved in CH_2Cl_2 (200 mL), and TIPSCl (22.2 g, 110 mmol, 1.1equiv.) was added slowly at 0 °C. Then the reaction mixture was stirred at the same temperature for 5 hours. The resulting solution was filtered through a short pad of Celite and the filtrate was concentrated in vacuum. The residue was purified by silica gel column chromatography (hexane/EtOAc, 50:1) to give the desired compound as colorless oil (22.1 g, 90 mmol, 90% yield).

Step 2: KOH (6.55 g, 117 mmol, 1.3 equiv.) was dissolved in EtOH (60 mL). At 0 °C, a solution of the above compound (22.1 mg, 90 mmol, 1.0 equiv.) in EtOH (400 mL) was slowly added. The mixture was stirred overnight at room temperature and concentrated under reduced pressure. The residue was dissolved in water (300 mL) and carefully neutralized with 1.0 M HCl solution to p*H* 3-4. The solution was extracted with EtOAc two times. The organic layer was washed with brine and dried over anhydrous Na_2SO_4, filtered and evaporated. The residue was purified by silica gel column chromatography (hexane/EtOAc, 3:1) to give the acid derivative as colorless oil (17.7 g, 76.5 mmol, 85% yiled).

Step 3: The acid derivative (17.7 g, 76.5 mmol, 1.0 equiv.) was dissolved in dry CH_2Cl_2 (450 mL), *N*,*O*-dimethylhydroxylamine hydrochloride (8.9 g, 92 mmol, 1.2 equiv.), DMAP (280 mg, 2.3 mmol), DCC (15.8 g, 76.5 mmol, 1 equiv.), and Et_3N (12.8 mL, 92 mmol, 1.2 equiv.) were sequentially added to the reaction mixture. The solution was stirred overnight at room temperature. The resulting solution was filtered through a short pad of Celite and the filtrate was concentrated in vacuum. Then 100 mL of saturated aq. NH_4Cl solution was added and the solution was

extracted thrice with CH_2Cl_2, washed with brine, and dried over Na_2SO_4. Evaporation of solvent and purification by flash chromatography (hexane/ethyl acetate, 5:1) afforded Weinreb amide as a colorless oil (16.7 g, 61 mmol, 80% yield).

Step 4: Weinreb amide (4.12 g, 15 mmol, 1.0 equiv.) was dissolved in dry THF. At 0 ^{o}C, the alkylmagnesium bromide (22.5 mmol, 1.5 equiv., 1.0 M in THF) was slowly added. After 4 hours, saturated aq. NH_4Cl solution was added, and the reaction mixture was slowly warmed to room temperature. The mixture was extracted with EtOAc. The combined organic layer was dried over Na_2SO_4, filtered, and evaporated. The residue was purified by flash chromatography (hexane/ethyl acetate, 20:1) to afford the compound **6** as a colorless oil (**6q**, R= Et, 3.29 g, 13.5 mmol, 90% yield; **6r**, R= *i*-Bu, 3.62 g, 13.3 mmol, 89% yield).

Then the substrates **6** were conducted in the next steps for the synthesis of the substrates **1q** and **1r**.

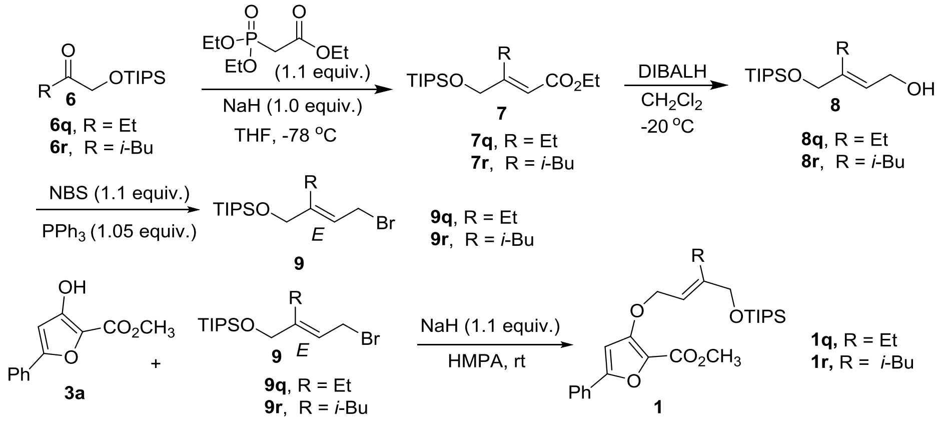

C**ompound 7:** The triethylphosphonoacetate (3.2 g, 14.3 mmol, 1.1 equiv.) was conducted in dry THF. At 0 ^{o}C, NaH (312 mg, 13 mmol, 1.0 equiv.) was added and the mixture was stirred at the same temperature for 30 min. The alkyl-substituted ketone **6** (13 mmol, 1.0 equiv.) in THF was added in the mixture and the mixture was stirred at room temperature for 12 h. Saturated aq. NH_4Cl solution was added. The resulting slurry was diluted with EtOAc and washed with water and brine. The organic layer was separated and dried over Na_2SO_4, filtered, and evaporated in vacuo to give the crude product as an *E*, *Z* mixture. The mixture was purified by flash chromatography on silica gel (0–1% EtOAc/hexane) and the E-product **7** could be obtained in good yield as a colorless oil (**7q**, R= Et, 1.82 g, 5.85 mmol, 45% yield; **7r**, R= *i*Bu, 1.77 g, 5.2 mmol, 40% yield).

Compound 8: The *E*-compound **7** (5 mmol, 1.0 equiv.) was dissolved in dry CH_2Cl_2. Then DIBAL-H (1.0 M hexane solution, 10.5 mmol, 2.1 equiv.) was dropped at –20 °C and the reaction continued for 2 h at the same temperature. Saturated aq. potassium tartrate solution was added and the mixture was vigorously stirred for 10 min. The solution was extracted thrice with CH_2Cl_2, washed with brine, and dried over Na_2SO_4. Evaporation of solvent and purification by flash chromatography (hexane/ethyl acetate, 10:1) afforded the compound **8** as a colorless oil (**8q**, R= Et, 1.14 g, 4.2 mmol, 94% yield; **8r**, R= *i*Bu, 1.27 g, 4.25 mmol, 95% yield).

Compound 9: The compound **8** (4.2 mmol, 1.0 equiv.) and triphenylphosphine (1.21 g, 4.62 mmol, 1.1 equiv.) were dissolved in CH_2Cl_2. *N*-bromosucciuimide (780 mg, 4.4 mmol, 1.05 equiv.) was added slowly at -20 ℃, stirred for 30 min at the same temperature. The reaction mixture was evaporated. The residue was purified by flash chromatography on silica gel (hexane/EtOAc, 50:1)

to provide the compound **9** as a colorless oil (**9q**, R= Et, 900 mg, 2.7 mmol, 65% yield; **9r**, R= *i*Bu, 900 mg,2.5 mmol, 60% yield).

(*E*)-substrate 1: The substituted furan (**3a**) (218 mg, 1.0 mmol, 1.0 equiv.) was dissolved in dry HMPA (5 mL). At room temperature, NaH (26.4 mg, 1.1 mmol, 1.1 equiv.) was added. After 1 hour, the compound **9** (1.5 mmol, 1.5 equiv.) in 4 mL of HMPA was added in drops. The mixture was stirred at room temperature overnight. Then 10 mL of saturated aq. NH_4Cl solution was added to quench the reaction. The solution was extracted three times with ethyl acetate, washed three times with brine, and dried over Na_2SO_4. Evaporation of solvent and purification by flash chromatography (hexane/ethyl acetate, 20:1) afforded the *E*-substrate **1** (**1q**, R= Et, 259 mg, 0.55 mmol, 55% yield; **1r**, R= *i*Bu, 280 mg, 0.56 mmol, 56% yield).

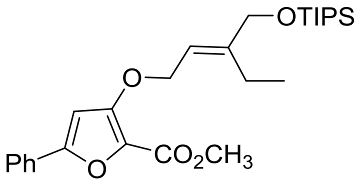

1q

Methyl-(*E*)-5-phenyl-3-((3-(((triisopropylsilyl)oxy)methyl)pent-2-en-1-yl)oxy)furan-2-carboxylate (**1q**); Synthesized by the general procedure, the reported yield is overall yield form the compound **6**.

Yield: 15% yield (clear oil)

^{1}H NMR (400 MHz, $CDCl_3$) δ 7.81 – 7.67 (m, 2H), 7.45 – 7.32 (m, 3H), 6.68 – 6.54 (m, 1H), 5.83 (t, *J* = 6.4 Hz, 1H), 4.79 (d, *J* = 6.0 Hz, 2H), 4.21 (s, 2H), 4.03 – 3.80 (m, 3H), 2.31 – 2.04 (m, 2H), 1.10 – 1.01 (m, 24H);

^{13}C NMR (101 MHz, $CDCl_3$) δ 159.4, 155.5, 155.3, 146.4, 129.4, 129.2, 128.8, 128.1, 124.8, 117.4, 98.2, 68.0, 65.6, 51.4, 21.6, 18.0, 13.5, 12.0;

HRMS (ESI) calcd for $[M+H]^+$: $C_{27}H_{41}O_5Si$, m/z: 473.2718, observed: 473.2723.

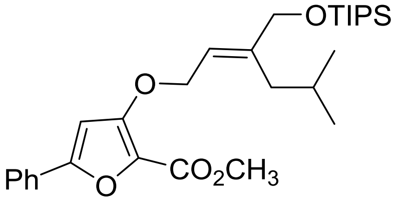

1r

Methyl-(*E*)-3-((5-methyl-3-(((triisopropylsilyl)oxy)methyl)hex-2-en-1-yl)oxy)-5-phenylfuran-2-carboxylate (**1r**); Synthesized by the general procedure, the reported yield is overall yield form the compound **6**.

Yield: 13% yield (clear oil)

^{1}H NMR (400 MHz, $CDCl_3$) δ 7.80 – 7.69 (m, 2H), 7.48 – 7.31 (m, 3H), 6.68 – 6.54 (m, 1H), 5.94 (t, *J* = 6.4 Hz, 1H), 4.78 (d, *J* = 6.4 Hz, 2H), 4.17 (s, 2H), 3.89 (d, *J* = 1.2 Hz, 3H), 2.02 (d, *J* = 7.6 Hz, 2H), 1.84 – 1.73 (m, 1H), 1.11 – 1.00 (m, 21H), 0.93 (d, *J* = 6.4 Hz, 6H);

^{13}C NMR (101 MHz, $CDCl_3$) δ 159.4, 155.5, 155.3, 143.5, 129.4, 129.2, 128.8, 128.1, 124.8, 119.0, 98.2, 68.3, 66.1, 51.4, 37.8, 27.5, 22.7, 18.0, 12.0;

HRMS (ESI) calcd for $[M+H]^+$: $C_{29}H_{45}O_5Si$, m/z: 501.3031, observed: 501.3029.

General procedure for the synthesis of (*E*)-substrate (1s)

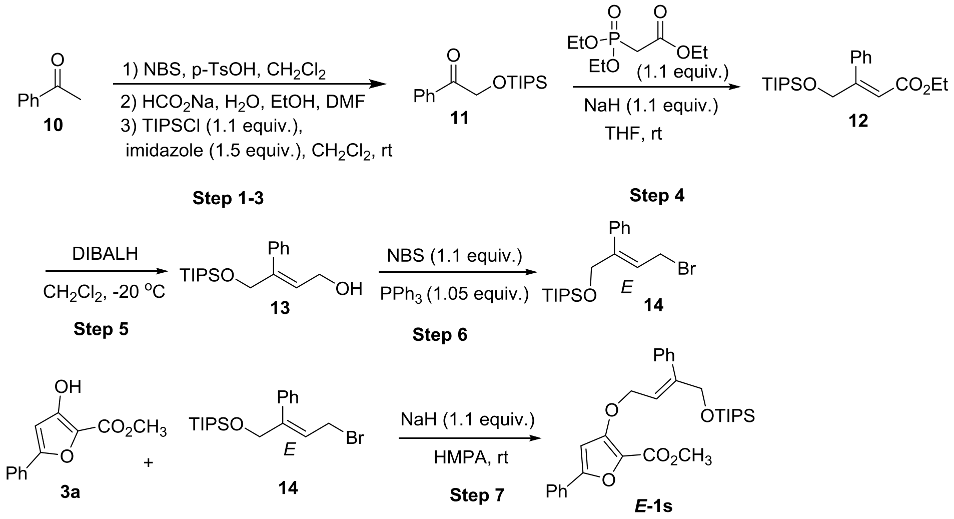

Step 1: The ketone **10** (6 g, 50 mmol, 1.0 equiv.) was dissolved in dry CH_2Cl_2. TsOH·H_2O (1.9 g, 10 mmol, 0.2 equiv.) and NBS (9.79 g, 55 mmol, 1.1 equiv.) was added in sequence and the reaction mixture was stirred at room temperature overnight. H_2O was added to quench the reaction. The solution was extracted three times with CH_2Cl_2, washed with brine, and dried over Na_2SO_4. Evaporation of solvent and purification by flash chromatography (hexane/ethyl acetate, 20:1) afford the bromo-product (7.96 g, 40 mmol, 80% yield).

Step 2: The bromo-product (7.96 g, 40 mmol, 1.0 equiv.), and HCO_2Na (8.16 g, 120 mmol, 3.0 equiv.) were dissolved in DMF (40 mL), H_2O (30 mL) and EtOH (110 mL). The mixture was heated to reflux. After 4-5 h, the reaction mixture was cooled to room temperature, then the solvent was removed under vacuum. The residue was diluted with water, and extracted twice with CH_2Cl_2. The combined organic layer was washed with brine. After drying over Na_2SO_4, solvent was evaporated under vacuum. The crude product was purified by flash chromatography (hexane/ethyl acetate, 5:1) to give the product (4.89 g, 36 mmol, 90% yield).

Step 3: The above compound (4.89g, 36 mmol, 1.0 equiv.) and imidazole (3.67 g, 54 mmol, 1.5 equiv.) were dissolved in CH_2Cl_2. TIPSCl (7.63 g, 39.6 mmol, 1.1 equiv.) was added slowly at 0 ^oC. Then the reaction mixture was stirred at the same temperature for 5 hours. The resulting solution was filtered off through a short pad of Celite and the filtrate was concentrated in vacuum. The residue was purified by silica gel column chromatography (hexane/EtOAc, 50:1) to give the compound **11** as colorless oil (9.46 g, 32.4 mmol, 90% yield).

Step 4: The triethylphosphonoacetate (4.93 g, 22 mmol, 1.1 equiv.) was dissolved in dry THF. At 0 ^oC, NaH (528 mg, 22 mmol, 1.1 equiv.) was added and the mixture was stirred at the same temperature for 30 min. The compound **11** (5.84 g, 20 mmol, 1.0 equiv.) in THF was added in the mixture and the mixture was stirred at room temperature for 12 h. Saturated aq. NH_4Cl solution was added. The resulting slurry was diluted with EtOAc and washed with water and brine. The organic layer was separated and dried over Na_2SO_4, filtered, and evaporated in vacuo to give the crude product as an *E*, *Z* mixture. The residue mixture was purified by flash chromatography on silica gel (0–1% EtOAc/hexane) to provide *E*-compound **12** as colorless oil (3.26 g, 9 mmol, 45% yield).

Step 5: The *E*-compound **12** (3.26 g, 9 mmol, 1.0 equiv.) was dissolved in dry CH_2Cl_2. DIBAL-H (1.0 M hexane solution, 18.9 mmol, 18.9 ml, 2.1 equiv.) was dropped at –20 ℃ and the

reaction was continued for 2 h at the same temperature. Saturated aq. potassium tartrate solution was added and the mixture was vigorously stirred for 10 min. The solution was extracted three times with CH_2Cl_2, washed with brine, and dried over Na_2SO_4. Evaporation of solvent and purification by flash chromatography (hexane/ethyl acetate, 10:1) afforded the compound **13** as colorless oil (2.43 g, 7.6 mmol, 95% yield).

Step 6: The compound **13** (5 mmol, 1.0 equiv.) and triphenylphosphine (1.44g, 5.5 mmol, 1.1 equiv.) were dissolved in dry CH_2Cl_2, then *N*-bromosucciuimide (943 mg, 5.3 mmol, 1.05 equiv.)was added slowly at -20 ℃, stirred for 30 min at room temperature. The reaction mixture was evaporated. The residue was purified by flash chromatography on silica gel (EtOAc/hexane, 50:1) to provide the compound **14** as colorless oil (1.34 g, 3.5 mmol, 65% yield).

Step 7: The substituted furan **3a** (218 mg, 1.0 mmol, 1.0 equiv.) was dissolved in dry HMPA (5 mL). At room temperature, NaH (26.4 mg, 1.1 mmol, 1.1 equiv.) was added. After 1 hour, the compound **14** (574 mg, 1.5 mmol, 1.5 equiv.) in 4 mL of HMPA was added in drops. The mixture was stirred at room temperature overnight. Then 10 mL of saturated aq. NH_4Cl solution was added to quench the reaction. The solution was extracted three times with ethyl acetate, washed three times with brine, and dried over Na_2SO_4. Evaporation of solvent and purification by flash chromatography (hexane/ethyl acetate, 20:1) afforded the product ***E*-1s** (260 mg, 0.5 mmol, 50% yield).

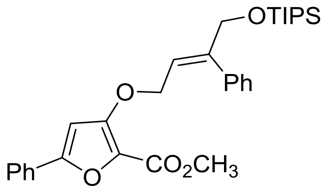

E-**1s**

Methyl-(*E*)-5-phenyl-3-((3-phenyl-4-((triisopropylsilyl)oxy)but-2-en-1-yl)oxy)furan-2-carboxylate (***E*-1s**);Synthesized by the general procedure, the reported yield is overall yield from the compound **10**.

Yield: 9% yield (clear oil)

^{1}H NMR (400 MHz, $CDCl_3$) δ 7.67 – 7.63 (m, 2H), 7.44 – 7.30 (m, 6H), 7.24 – 7.19 (m, 2H), 6.27 (s, 1H), 6.20 – 6.16 (m, 1H), 4.70 (d, *J* = 6.8 Hz, 2H), 4.45 (d, *J* = 1.2 Hz, 2H), 3.89 (s, 3H), 1.12 – 0.98 (m, 21H);

^{13}C NMR (101 MHz, $CDCl_3$) δ 159.3, 155.2, 155.1, 145.9, 137.3, 129.4, 129.2, 128.7, 128.5, 128.4, 128.1, 124.7, 119.8, 98.4, 68.7, 66.4, 51.3, 18.0, 12.0;

HRMS (ESI) calcd for $[M+H]^+$: $C_{31}H_{41}O_5Si$, m/z: 521.2717, observed: 521.2717.

General procedure for the synthesis of (*Z*)-substrates (1b' and 1n')

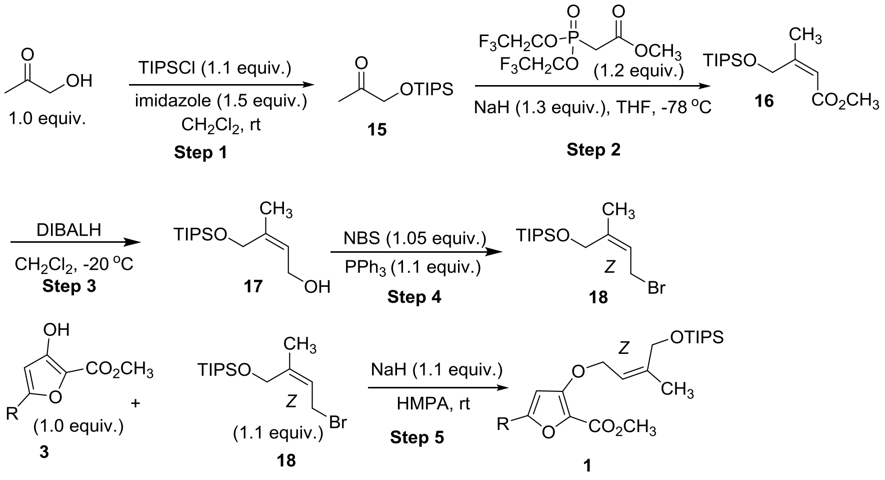

Step 1: The hydroxy acetone (1.48 g, 20 mmol, 1.0 equiv.) and imidazole (2.04 g, 30 mmol, 1.5 equiv.) were dissolved in CH_2Cl_2.TIPSCl (3.85 g, 20 mmol, 1.0 equiv.) was added slowly at 0 ^oC, then the reaction mixture was stirred for 5 hours at room temperature. The resulting solution was filtered through a short pad of Celite and the filtrate was concentrated in vacuum. The residue was purified by silica gel column chromatography (hexane/EtOAc, 50:1) to give the compound **15** as colorless oil (4.37 g, 19 mmol, 95% yield).

Step 2: The (2, 2, 2-trifluoroethyl) methoxycarbonylmethyl-phosphonate (7.25 g, 22.8 mmol, 1.2 equiv.) was dissolved in dry THF. At 0 ^oC, NaH (456 mg, 19 mmol, 1.0 equiv.) was added and the mixture was stirred at the same temperature for 30 min. Then, the reaction mixture was cooled to -78 ^oC, the compound **15** (4.37 g, 19 mmol, 1.0 equiv.) in THF was added in the mixture. Next, the mixture was stirred at -78 ^oC for 12 h. Saturated aq. NH_4Cl solution was added. The resulting slurry was diluted with EtOAc and washed with water and brine. The organic layer was separated and dried over Na_2SO_4, filtered, and evaporated in vacuo to give the crude product as an *E*, *Z* mixture. The residue mixture was purified by flash chromatography on silica gel (0–1% EtOAc/hexane) to provide *Z*-compound **16** as colorless oil (3.26 g, 11.4 mmol, 60% yield).

Step 3: The *Z*-compound **16** (3.26 g, 11.4 mmol, 1.0 equiv.) was dissolved in dry CH_2Cl_2, DIBAL-H was dropped (1.0M hexane solution, 23.9 mmol, 24 mL, 2.1 equiv.) at –20 °C and the reaction continued stirring for 2 h at the same temperature. Saturated aq. potassium tartrate solution was added and the mixture was vigorously stirred for 10 min. The solution was extracted thrice with CH_2Cl_2, washed with brine, and dried over Na_2SO_4. Evaporation of solvent and purification by flash chromatography (hexane/ethyl acetate, 10:1) afforded the compound **17** as colorless oil (2.79 g, 10.8 mmol, 95% yield).

Step 4: The compound **17** (2.79 g, 10.8 mmol, 1.0 equiv.) and triphenylphosphine (3.11 g, 11.88 mmol, 1.1 equiv.) were dissolved in CH_2Cl_2. *N*-bromosucciuimide (2.0 g, 11.34 mmol, 1.05 equiv.) was added slowly at -20 ℃, stirred for 30 min at the same temperature. The solution was extracted with CH_2Cl_2, washed with brine, and dried over Na_2SO_4.The solvent was evaporated. The residue was purified by flash chromatography on silica gel (EtOAc/hexane, 50:1) to provide the compound **18** as colorless oil (7 mmol, 65% yield).

Step 5: The substituted furan (**3**) (1.0 mmol, 1.0 equiv.) was dissolved in dry HMPA (5 mL). At room temperature, NaH (26.4 mg, 1.1 mmol, 1.1 equiv.) was added. After 1 hour, the compound **18** (1.5 mmol, 1.5 equiv.) in 4 mL of HMPA was added in drops. The mixture was stirred at room

temperature overnight. Then 10 mL of saturated aq. NH_4Cl solution was added to quench the reaction. The solution was extracted three times with ethyl acetate, washed three times with brine, and dried over Na_2SO_4. Evaporation of solvent and purification by flash chromatography (hexane/ethyl acetate, 20:1) afforded *Z*-substrate (**1b'** 256 mg, 0.56 mmol, 56% yield; **1n'** (241 mg, 0.57 mmol, 57% yield).

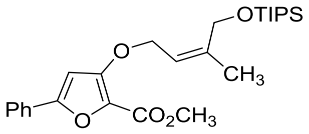

Z-**1b'**

Methyl-(*Z*)-3-((3-methyl-4-((triisopropylsilyl)oxy)but-2-en-1-yl)oxy)-5-phenylfuran-2-carboxylate (*Z*-1b'): Synthesized by the general procedure, the reported yield is overall yield form hydroxy acetone.

Yield: 20% yield (clear oil)

^{1}H NMR (400 MHz, $CDCl_3$) δ 7.75 (d, *J* = 7.8 Hz, 2H), 7.47 – 7.32 (m, 3H), 6.62 (s, 1H), 5.55 (s, 1H), 4.83 (d, *J* = 5.6 Hz, 2H), 4.33 (s, 2H), 3.90 (d, *J* = 1.2 Hz, 3H), 1.83 (s, 3H), 1.18 – 1.01 (m, 21H);

^{13}C NMR (101 MHz, $CDCl_3$) δ 159.3, 155.5, 155.4, 140.7, 129.4, 129.3, 128.8, 124.8, 120.7, 98.0, 68.2, 63.1, 51.4, 21.2, 18.0, 12.0;

HRMS (ESI) calcd for $[M+H]^+$: $C_{26}H_{39}O_5Si$, m/z: 459.2567, observed: 459.2566.

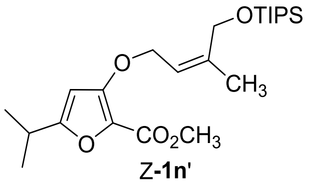

Z-**1n'**

Methyl-(*Z*)-5-isopropyl-3-((3-methyl-4-((triisopropylsilyl)oxy)but-2-en-1-yl)oxy)furan-2-carboxylate (*Z*-1n'): Synthesized by the general procedure, the reported yield is overall yield form hydroxy acetone.

Yield: 21% yield (clear oil)

^{1}H NMR (400 MHz, $CDCl_3$) δ 6.00 (s, 1H), 5.53 – 5.49 (m, 1H), 4.74 (d, *J* = 6.4 Hz, 2H), 4.29 (s, 2H), 3.86 (s, 3H), 2.96 – 2.89 (m, 1H), 1.81 (s, 3H), 1.26 (d, *J* = 6.98 Hz, 6H), 1.09 – 1.05 (m, 21H);

^{13}C NMR (101 MHz, $CDCl_3$) δ 165.2, 159.4, 155.0, 140.4, 126.8, 120.8, 97.3, 68.0, 63.1, 51.2, 28.6, 21.1, 20.7, 18.0, 11.9;

HRMS (ESI) calcd for $[M+H]^+$: $C_{23}H_{41}O_5Si$, m/z: 425.2718, observed: 425.2717.

General procedure for the synthesis of (*Z*)-substrates (1s' -1x')

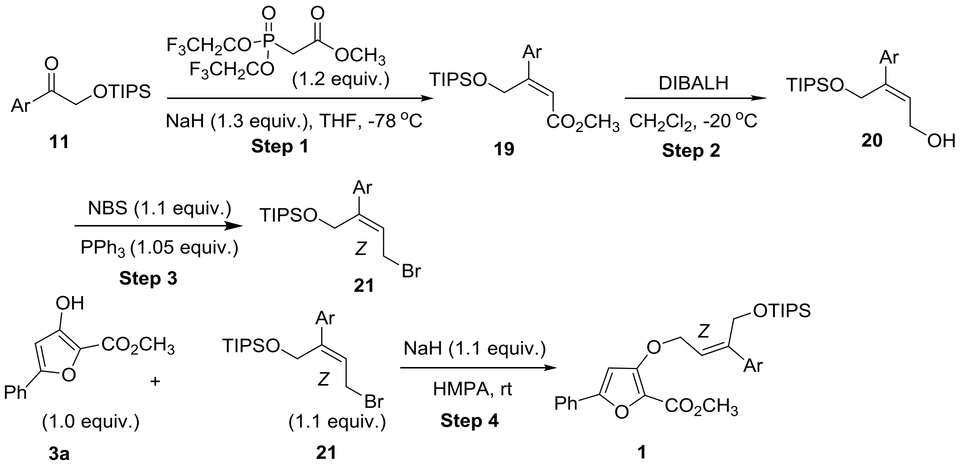

Step 1: The (2, 2, 2-trifluoroethyl)-methoxycarbonylmethyl-phosphonate (3.8 g, 12 mmol, 1.2 equiv.) was dissolved in dry THF. At 0 °C, NaH (312 mg, 13 mmol, 1.3 equiv.) was added and the mixture was stirred at the same temperature for 30 min. Then, the reaction mixture was cooled to -78 °C, the compound **11** (10 mmol, 1.0 equiv.) in THF was added in the mixture and stirred at -78 °C for 12 h, then stirred at room temperature for 5h. Saturated aq. NH_4Cl solution was added. The resulting slurry was diluted with EtOAc and washed with water and brine. The organic layer was separated and dried over Na_2SO_4, filtered, and evaporated in vacuo to give the crude product as an *E*, *Z* mixture. The residue mixture was purified by flash chromatography on silica gel (0–1% EtOAc/hexane) to provide *Z*-compound **19** as colorless oil.

Step 2: The *Z*-compound **19** (5 mmol, 1.0 equiv.) was dissolved in dry CH_2Cl_2 was dropped DIBAL-H (1.0 M hexane solution, 10.5 mmol, 10.5 mL,2.1 equiv.) at –20 °C and the reaction was continued for 2 h at the same temperature. Saturated aq. potassium tartrate solution was added and the mixture was vigorously stirred for 10 min. The solution was extracted thrice with CH_2Cl_2, washed with brine, and dried over Na_2SO_4. Evaporation of solvent and purification by flash chromatography (hexane/ethyl acetate, 10:1) afforded the compound **20** as colorless oil.

Step 3: The compound **20** (4 mmol, 1.0 equiv.) and triphenylphosphine (1.15 g, 4.4 mmol, 1.1 equiv.) were dissolved in CH_2Cl_2, and *N*-bromosucciuimide (747 mg, 4.2 mmol, 1.05 equiv.) was added slowly at -20 °C, stirred for 30 min at –20 °C. The solution was extracted thrice with CH_2Cl_2, washed with brine, and dried over Na_2SO_4.The reaction solvent was evaporated. The residue was purified by flash chromatography on silica gel (EtOAc/hexane, 50:1) to provide the compound **21** as a colorless oil.

Step 5: The substituted furan (**3a**) (218 mg, 1.0 mmol, 1.0 equiv.) was dissolved in dry HMPA (5 mL). At room temperature, NaH (26.4 mg, 1.1 mmol, 1.1 equiv.) was added. After 1 hour, the compound **21** (1.5 mmol, 1.0 equiv.) in 4 mL of HMPA was added in drops. The mixture was stirred at room temperature overnight. Then 10 mL of saturated aq. NH_4Cl solution was added to quench the reaction. The solution was extracted three times with ethyl acetate, washed three times with brine, and dried over Na_2SO_4. Evaporation of solvent and purification by flash chromatography (hexane/ethyl acetate, 20:1) afforded *Z*-product **1s'-1x'**.

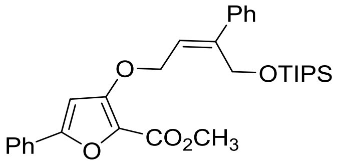

Z-**1s′**

Methyl-(*Z*)-5-phenyl-3-((3-phenyl-4-((triisopropylsilyl)oxy)but-2-en-1-yl)oxy)furan-2-carboxylate (*Z*-1s′): Synthesized by the general procedure, the reported yield is overall yield from the compound **11**.

Yield: 15% yield (clear oil)

^{1}H NMR (400 MHz, $CDCl_3$) δ 7.74 (d, *J* = 7.2 Hz, 2H), 7.44 – 7.23 (m, 8H), 6.68 (s, 1H), 6.02 (t, *J* = 5.6 Hz, 1H), 5.13 (d, *J* = 6.0 Hz, 2H), 4.73 (s, 2H), 3.91 (s, 3H), 1.16 – 0.99 (m, 21H);

^{13}C NMR (101 MHz, $CDCl_3$) δ 159.3, 155.6, 155.4, 142.5, 140.6, 129.4, 128.8, 128.3, 128.0, 127.6, 126.7, 125.5, 124.9, 98.0, 69.2, 62.8, 51.5, 18.1, 12.0;

HRMS (ESI) calcd for $[M+H]^+$: $C_{31}H_{41}O_5Si$, m/z: 521.2718, observed: 521.2718.

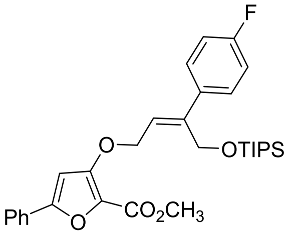

Z-**1t′**

Methyl-(*Z*)-3-((3-(4-fluorophenyl)-4-((triisopropylsilyl)oxy)but-2-en-1-yl)oxy)-5-phenylfuran-2-carboxylate (*Z*-1t′); Synthesized by the general procedure, the reported yield is overall yield from the compound **11**.

Yield: 14% yield (clear oil)

^{1}H NMR (400 MHz, $CDCl_3$) δ 7.77 – 7.72 (m, 2H), 7.42 – 7.29 (m, 5H), 7.10 – 6.94 (m, 2H), 6.68 (d, *J* = 2.0 Hz, 1H), 5.98 (t, *J* = 5.6 Hz, 1H), 5.09 (d, *J* = 5.6 Hz, 2H), 4.70 (s, 2H), 3.91 (d, *J* = 2.0 Hz, 3H), 1.15 – 1.01 (m, 21H);

^{13}C NMR (101 MHz, $CDCl_3$) δ 162.5 (d, 246 Hz), 159.3, 155.3, 154.9, 145.1, 133.1 (d, J = 4 Hz), 130.1 (d, J= 8 Hz), 129.3, 129.2, 128.8, 128.1, 124.7, 120.4, 115.4 (d, J = 21Hz), 115.0, 98.2, 68.5, 66.6, 51.3, 17.9, 11.9;

HRMS (ESI) calcd for $[M+H]^+$: $C_{31}H_{40}O_5SiF$, m/z: 539.2629, 540.2663, observed: 539.2625, 540.2651.

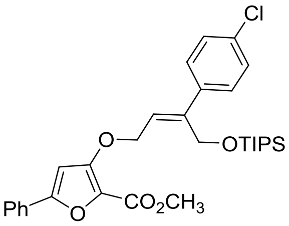

Z-**1u′**

Methyl-(*Z*)-3-((3-(4-chlorophenyl)-4-((triisopropylsilyl)oxy)but-2-en-1-yl)oxy)-5-phenylfuran-2-carboxylate (*Z*-1u′); Synthesized by the general procedure, the reported yield is overall yield from the compound **11**.

Yield: 16% yield (clear oil)

^{1}H NMR (400 MHz, $CDCl_3$) δ 7.74 (d, *J* = 7.2 Hz, 2H), 7.40 (t, *J* = 8.0 Hz, 2H), 7.37 – 7.31 (m, 3H), 7.31 – 7.25 (m, 2H), 6.67 (s, 1H), 6.02 (t, *J* = 5.6 Hz, 1H), 5.08 (d, *J* = 6.0 Hz, 2H), 4.69 (s, 2H), 3.91 (s, 3H), 1.16 – 1.03 (m, 21H);

^{13}C NMR (101 MHz, $CDCl_3$) δ 159.3, 155.6, 155.2, 141.6, 138.9, 133.4, 129.3, 128.81 (s, 5H), 128.4, 128.0, 125.7, 124.8, 97.9, 68.9, 62.2, 51.4, 18.0, 12.0;

HRMS (ESI) calcd for $[M+H]^+$: $C_{31}H_{40}O_5SiCl$, m/z: 555.2334, 556.2367, observed: 555.2338, 556.2367.

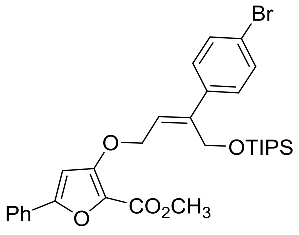

Z-**1v′**

Methyl-(*Z*)-3-((3-(4-bromophenyl)-4-((triisopropylsilyl)oxy)but-2-en-1-yl)oxy)-5-phenylfuran-2-carboxylate (*Z*-1v'); Synthesized by the general procedure, the reported yield is overall yield from compound **11**.

Yield: 16% yield (clear oil)

^{1}H NMR (400 MHz, $CDCl_3$) δ 7.74 (d, *J* = 8.0 Hz, 2H), 7.47 – 7.31 (m, 5H), 7.31 – 7.21 (m, 2H), 6.66 (d, *J* = 1.2 Hz, 1H), 6.02 (t, *J* = 5.2 Hz, 1H), 5.08 (d, *J* = 5.6 Hz, 2H), 4.69 (s, 2H), 3.91 (d, *J* = 1.2 Hz, 3H), 1.15 – 1.02 (m, 21H);

^{13}C NMR (101 MHz, $CDCl_3$) δ 159.3, 155.6, 155.2, 141.6, 139.4, 131.4, 129.4, 129.3, 128.8, 128.4, 128.0, 125.8, 124.8, 121.6, 97.9, 68.9, 62.1, 51.5, 51.4, 18.0, 12.0;

HRMS (ESI) calcd for $[M+H]^+$: $C_{31}H_{40}O_5SiBr$, m/z: 599.1828, 601.1808, observed: 599.1817, 601.1799.

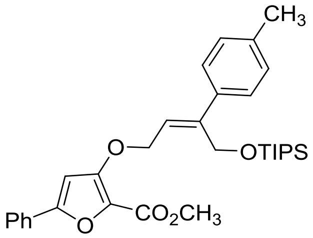

Z-**1w′**

Methyl-(*Z*)-5-phenyl-3-((3-(p-tolyl)-4-((triisopropylsilyl)oxy)but-2-en-1-yl)oxy)furan-2-carboxylate(*Z*:*E*=5:1); Synthesized by the general procedure, the reported yield is overall yield from the compound **11**.

Yield: 12% yield (clear oil)

^{1}H NMR (400 MHz, $CDCl_3$) δ 7.74 – 7.26 (m, 2H), 7.40 – 7.28 (m, 5H), 7.11 (d, *J* = 8.0 Hz, 2H), 6.69 (s, 1H), 6.01 (t, *J* = 6.0 Hz , 1H), 5.13 (d, *J* = 6.0 Hz, 2H), 4.71 (s, 2H), 3.90 (s, 3H), 2.31 (s, 3H), 1.10– 1.01 (m, 21H);

^{13}C NMR (101 MHz, $CDCl_3$) δ 159.3, 155.6, 155.4, 142.2, 137.7, 137.4, 129.4, 129.3, 129.2, 129.0, 128.8, 128.7, 128.3, 128.0, 126.5, 124.8, 98.0, 69.3, 62.8, 51.4, 21.1, 18.1, 12.0;

HRMS (ESI) calcd for $[M+H]^+$: $C_{32}H_{43}O_5Si$, m/z:535.2874, observed: 535.2874.

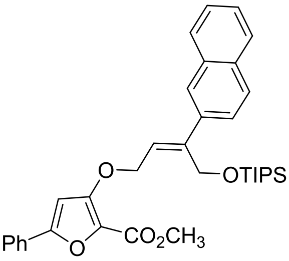

Z-**1x′**

Methyl-(*Z*)-3-((3-(naphthalen-2-yl)-4-((triisopropylsilyl)oxy)but-2-en-1-yl)oxy)-5-phenylfuran-2-carboxylate (*Z*-1x'); Synthesized by the general procedure, the reported yield is overall yield from the compound **11**.

Yield: 15% yield (clear oil)

^{1}H NMR (400 MHz, $CDCl_3$) δ 7.88 (s, 1H), 7.84 – 7.70 (m, 5H), 7.55 (t, *J* = 8.8 Hz, 1H), 7.50 – 7.30 (m, 5H), 6.70 (d, *J* = 2.0 Hz, 1H), 6.19 (t, *J* = 5.6 Hz, 1H), 5.17 (d, *J* = 5.6 Hz, 2H), 4.83 (s, 2H), 3.92 (d, *J* = 1.6 Hz, 3H), 1.22 – 1.06 (m, 21H);

^{13}C NMR (101 MHz, $CDCl_3$) δ 159.4, 155.6, 155.4, 142.2, 137.9, 133.3, 132.8, 129.4, 129.3, 128.8, 128.2, 128.0, 127.9, 127.6, 126.2, 126.1, 126.0, 125.5, 124.9, 98.0, 69.3, 62.6, 51.5, 26.9, 18.1, 12.0;

HRMS (ESI) calcd for $[M+H]^+$: $C_{35}H_{43}O_5Si$, m/z: 571.2880, observed: 571.2887.

General procedure for the synthesis of the substrate 1y

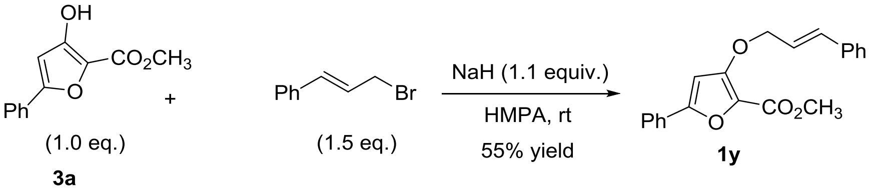

The substituted furan (**3a**) (218 mg, 1.0 mmol, 1.0 equiv.) was dissolved in dry HMPA (5 mL). At room temperature, NaH (26.4 mg, 1.1 mmol, 1.1 equiv.) was added. After 1 hour, cinnamyl bromide (296 mg, 1.5 mmol, 1.5 equiv.) in 4 mL of HMPA was added in drops. The mixture was stirred at room temperature overnight. Then 10 mL of saturated aq. NH_4Cl solution was added to quench the reaction. The solution was extracted three times with ethyl acetate, washed three times with brine, and dried over Na_2SO_4. Evaporation of solvent and purification by flash chromatography (hexane/ethyl acetate, 20:1) afforded the substrate **1y**.

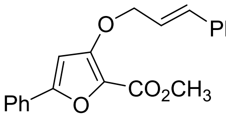

1y

Methyl 3-(cinnamyloxy)-5-phenylfuran-2-carboxylate (1y);

Yield: 184 mg, 0.55 mmol, 55% yield (white solid)

^{1}H NMR (400 MHz, $CDCl_3$) δ 7.74 (d, *J* = 6.4 Hz, 2H), 7.46 – 7.21 (m, 8H), 6.75 (d, *J* = 16.0 Hz, 1H), 6.64 (s, 1H), 6.42 – 6.38 (m, 1H), 4.84 (s, 2H), 3.91 (s, 3H);

^{13}C NMR (101 MHz, $CDCl_3$) δ 159.3, 155.6, 155.3, 136.1, 133.9, 129.4, 129.3, 128.8 , 128.7, 128.2, 128.0, 126.7, 124.9, 123.5, 98.0, 72.4, 51.5;

HRMS (ESI) calcd for $[M+H]^+$: $C_{21}H_{19}O_4$, m/z: 355.1277, observed: 355.1277.

General procedure for the synthesis of substrate *E*-1z and *Z*-1z'

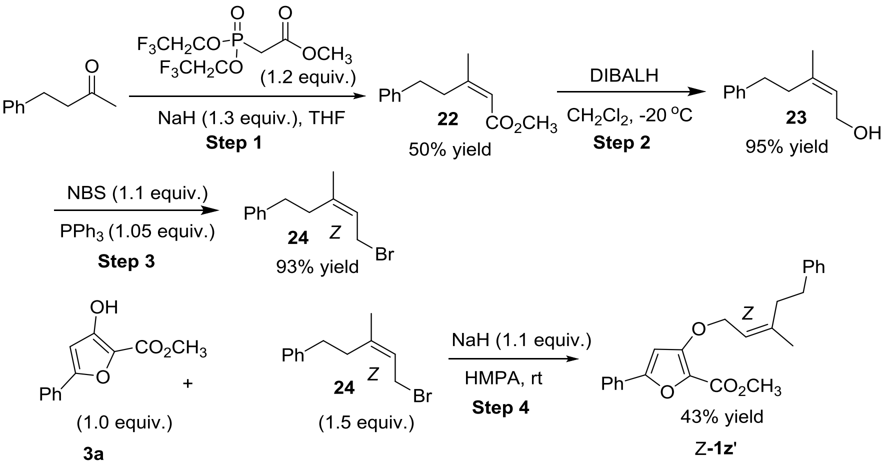

Step 1: The (2, 2, 2-trifluoroethyl)methoxycarbonylmethyl-phosphonate (5.72 g, 18 mmol, 1.2 equiv.) was dissolved in dry THF. At 0 ^oC, the NaH (456 mg, 19.5 mmol, 1.3 equiv.) was added and the mixture was stirred at the same temperature for 30 min. Then, the reaction mixture was cooled to -78 ^oC, the ketone (2.22 g, 15 mmol, 1.0 equiv.) in THF was added in the mixture at and the mixture was stirred at -78 ^oC for 12 h, then stirred at room temperature for 5h. Saturated aq. NH_4Cl solution was added. The resulting slurry was diluted with EtOAc and washed with water and brine. The organic layer was separated and dried over Na_2SO_4, filtered, and evaporated in vacuo to give the crude product as an *E*, *Z* mixture. The residue mixture was purified by flash chromatography on silica gel (0–1% EtOAc/hexane) to provide *Z*-compound **22** as colorless oil (1.53 g, 7.5 mmol, 50% yield).

Step 2: The *Z*-compound **22** (1.53 g, 7.5 mmol, 1.0 equiv.) was dissolved in dry CH_2Cl_2. DIBAL-H (1.0 M hexane solution, 15.8 mmol, 15.8 mL, 2.1 equiv.) was dropped at –20 °C and the reaction was continued for 2 h at the same temperature. Saturated aq. potassium tartrate solution was added and the mixture was vigorously stirred for 10 min. The solution was extracted three times with CH_2Cl_2, washed with brine, and dried over Na_2SO_4. Evaporation of solvent and purification by flash chromatography (hexane/ethyl acetate, 10:1) afforded the compound **23** as colorless oil (1.23 g, 7 mmol, 94% yield).

Step 3: The compound **23** (1.23 g, 7 mmol, 1.0 equiv.) and triphenylphosphine (2.0 g, 7.7 mmol, 1.1 equiv.) were dissolved in CH_2Cl_2. *N*-bromosucciuimide (1.42 g, 8 mmol, 1.05 equiv.) was added slowly at -20 ℃, stirred for 30 min at –20 °C. The solution was extracted three times with CH_2Cl_2, washed with brine, and dried over Na_2SO_4.The reaction solvent was evaporated. The residue was purified by flash chromatography on silica gel (EtOAc/hexane, 50:1) to provide the compound **24** as colorless oil (1.32 g, 5.5 mmol, 79% yield).

Step 5: The substituted furan (**3a**) (218 mg, 1.0 mmol, 1.0 equiv.) was dissolved in dry HMPA (5 mL). At room temperature, NaH (26.4 mg, 1.1 mmol, 1.1 equiv.) was added. After 1 hour, the compound **24** (358 mg, 1.5 mmol, 1.5 equiv.) in 4 mL of HMPA was added in drops. The mixture was stirred at room temperature overnight. Then 10 mL of saturated aq. NH_4Cl solution was added to quench the reaction. The solution was extracted three times with ethyl acetate, washed three times with brine, and dried over Na_2SO_4. Evaporation of solvent and purification by flash chromatography (hexane/ethyl acetate, 20:1) afforded the substrate **Z-1z'** (161 mg, 0.43 mmol, 43% yield).

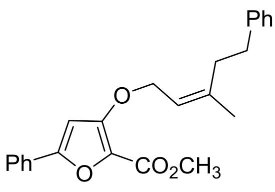

Z-**1z′**

Methyl (*Z*)-3-((3-methyl-5-phenylpent-2-en-1-yl)oxy)-5-phenylfuran-2-carboxylate (*Z*-1z');
Synthesized by the general procedure, the reported yield is overall yield from ketone.
Yield: 16% yield (clear oil);
^{1}H NMR (400 MHz, $CDCl_3$) δ 7.74 (d, *J* = 6.8 Hz, 2H), 7.43 – 7.33 (m, 3H), 7.31 – 7.27 (m, 2H), 7.22 – 7.17 (m, 3H), 6.44 (d, *J* = 1.6 Hz, 1H), 5.52 (s, 1H), 4.34 (d, *J* = 5.6 Hz, 2H), 3.87 (d, *J* = 1.6 Hz, 3H), 2.75 (t, *J* = 7.2 Hz, 2H), 2.43 (t, *J* = 7.2 Hz, 2H), 1.83 (s, 3H);
^{13}C NMR (101 MHz, $CDCl_3$) δ 159.3, 155.4, 141.6, 141.2, 129.4, 129.3, 128.8, 128.6, 128.4, 126.1, 124.9, 120.6, 97.9, 68.1, 51.4, 34.7, 34.3, 23.6;
HRMS (ESI) calcd for $[M+H]^+$: $C_{24}H_{25}O_4$, m/z: 377.1747, observed: 377.1748.

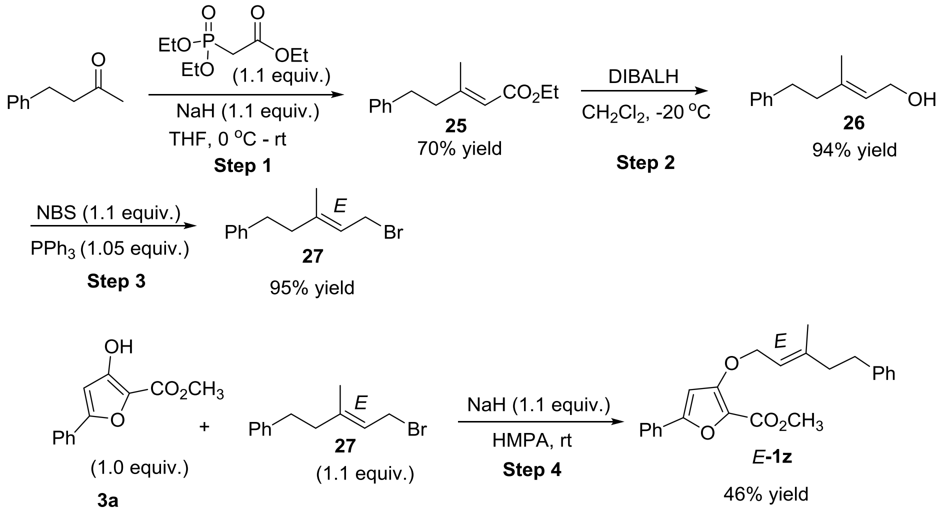

Step 1: The triethylphosphonoacetate (3.69 g, 16.5 mmol, 1.1 equiv.) was dissolved in dry THF. At 0 °C, the NaH (396 mg, 16.5 mmol, 1.1 equiv.) was added and the mixture was stirred at the same temperature for 30 min. The ketone (2.22 g, 15 mmol, 1.0 equiv.) in THF was added in the mixture and the mixture was stirred at the room temperature for 12 h. Saturated aq NH_4Cl solution was added. The resulting slurry was diluted with EtOAc and washed with water and brine. The organic layer was separated and dried over Na_2SO_4, filtered, and evaporated in vacuo to give the crude product as an *E*, *Z* mixture. The residue mixture was purified by flash chromatography on silica gel (0–1% EtOAc/hexane) to provide *E*-compound **25** as a colorless oil (2.29g, 10.5 mmol, 70% yield).
Step 2: The *E*-compound **25** (2.29 g, 10.5 mmol, 1.0 equiv.) was dissolved in dry CH_2Cl_2. DIBAL-H (1.0 M hexane solution, 22 mmol, 22 mL, 2.1 equiv.) was dropped at –20 °C and the reaction was continued for 2 h at the same temperature. Saturated aq. potassium tartrate solution was added and the mixture was vigorously stirred for 10 min. The solution was extracted three times with CH_2Cl_2, washed with brine, and dried over Na_2SO_4. Evaporation of solvent and purification by flash chromatography (hexane/ethyl acetate, 10:1) afforded the compound **26** as colorless oil (1.74 g, 9.9 mmol, 94% yield).

Step 3: The compound **26** (880 mg, 5 mmol, 1.0 equiv.) and triphenylphosphine (1.44g, 5.5 mmol, 1.1 equiv.) were dissolved in CH_2Cl_2. *N*-bromosucciuimide (925 mg, 5.2 mmol, 1.05 equiv.) was added slowly at -20 ℃, stirred for 30 min at room temperature. The solution was extracted three times with CH_2Cl_2, washed with brine, and dried over Na_2SO_4. The reaction solvent was evaporated. The residue was purified by flash chromatography on silica gel (EtOAc/hexane, 50:1) to provide the compound **27** as colorless oil (1.12 g, 4.7 mmol, 80% yield).

Step 4: The substituted furan **3a** (218 mg, 1.0 mmol, 1.0 equiv.) was dissolved in dry HMPA (5 mL). At room temperature, NaH (26.4 mg, 1.1 mmol, 1.1 equiv.) was added. After 1 hour, the compound **27** (356 mg, 1.5 mmol, 1.5 equiv.) in 4 mL of HMPA was added in drops. The mixture was stirred at room temperature overnight. Then 10 mL of saturated aq NH_4Cl solution was added to quench the reaction. The solution was extracted thrice with ethyl acetate, washed three times with brine, and dried over Na_2SO_4. Evaporation of solvent and purification by flash chromatography (hexane/ethyl acetate, 20:1) afforded product ***E*-1z** (173 mg, 0.46 mmol, 46% yield).

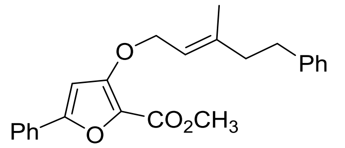

E-**1z**

Methyl (*E*)-3-((3-methyl-5-phenylpent-2-en-1-yl)oxy)-5-phenylfuran-2-carboxylate (*E*-1z); Synthesized by the general procedure, the reported yield is overall yield from ketone.

Yield: 24% yield (clear oil);

^{1}H NMR (400 MHz, $CDCl_3$) δ 7.74 (d, *J* = 6.8 Hz, 2H), 7.52 – 7.32 (m, 3H), 7.27 – 7.21 (m, 2H), 7.15 (d, *J* = 6.4 Hz, 3H), 6.54 (d, *J* = 1.6 Hz, 1H), 5.52 (s, 1H), 4.70 (d, *J* = 5.6 Hz, 2H), 3.90 (d, *J* = 2.0 Hz, 3H), 2.75 (t, *J* = 7.2 Hz, 2H), 2.37 (t, *J* = 7.2 Hz, 2H), 1.80 (s, 3H).

^{13}C NMR (101 MHz, $CDCl_3$) δ 159.3, 155.4, 141.7, 141.3, 129.4, 129.3, 128.8, 128.3, 128.0, 125.9, 124.9, 119.5, 98.0, 68.7, 51.4, 41.2, 34.1, 17.0;

HRMS (ESI) calcd for $[M+H]^+$: $C_{24}H_{25}O_4$, m/z: 377.1747, observed: 377.1743.

General procedure for the catalytic asymmetric Claisen rearrangement of substituted furans

In a test tube with a magnetic stirring bar, *N,N'*-dioxide ligand (**L-PiMe$_2$** or *ent*-**L-PiMe$_2$** or *ent*-**L-PiMe$_3$**) (10 mol%), $Ni(BF_4)_2$ $6H_2O$ (10 mol%) in CH_2Cl_2 (0.01 M) were stirred at 35 ^oC for 1h. Then removing CH_2Cl_2 and adding the substrate **1**, DCE (0.1 M). The mixture was stirred at 35 ^oC for 4 - 8 days. The reaction mixture was detected by TLC. After completion, flash column chromatography was carried out to provide the desired product. The product was analyzed by HPLC and NMR.

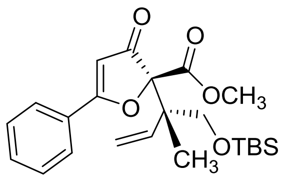

(*S*,*S*)-**2a**

Methyl-(*S*)-2-((*S*)-1-((tert-butyldimethylsilyl)oxy)-2-methylbut-3-en-2-yl)-3-oxo-5-phenyl-2,3-dihydrofuran-2-carboxylate (**2a**)

Colourless oil; 37.4 mg, 90% yield, 99%/98% ee, 8:1 dr; $[a]_D^{25} = -106$ (c 0.36, CH_2Cl_2); Determined by HPLC analysis [Daicel chiralpak IC, n-hexane/i-PrOH = 90/10, 0.8 mL/min, λ = 254 nm, t_1 = 15.30 min, t_2 =

16.17 min, t_3 = 18.85 min, t_4 = 19.62 min];

^{1}H NMR (400 MHz, $CDCl_3$) δ 7.76 – 7.63 (m, 2H), 7.43 – 7.27 (m, 3H), 6.54 (s, 1H), 5.81 – 5.69 (m, 1H), 4.71 (d, *J* = 6.4 Hz, 2H), 4.01 (s, 2H), 3.84 (s, 3H), 1.68 (s, 3H), 0.84 (s, 9H), 0.00 (s, 6H);

^{13}C NMR (101 MHz, $CDCl_3$) δ 159.3, 155.5, 155.3, 140.5, 129.3, 128.8, 124.8, 118.1, 98.1, 68.4, 67.4, 51.4, 25.9, 18.4, 13.9, -5.4;

HRMS (ESI) calcd for $[M+H]^+$: $C_{23}H_{33}O_5Si$, m/z: 417.2091, observed: 417.2102;

Chiral HPLC spectrum of racemic **2a**

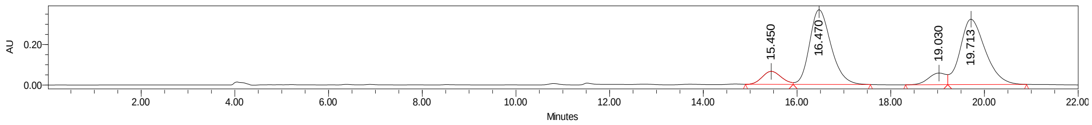

	Retention Time	Area	% Area
1	15.450	1816592	6.71
2	16.470	11718615	43.28
3	19.030	1489583	5.50
4	19.713	12049243	44.50

*Chiral HPLC spectrum of cat-***2a**

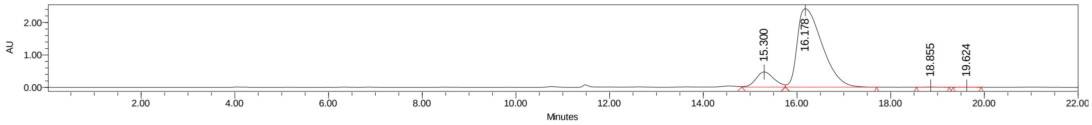

	Retention Time	Area	% Area
1	15.300	12512310	12.27
2	16.178	89268493	87.55
3	18.855	104746	0.10
4	19.624	82323	0.08

	Retention Time	Area	% Area
1	16.178	89268493	99.91
2	19.624	82323	0.09

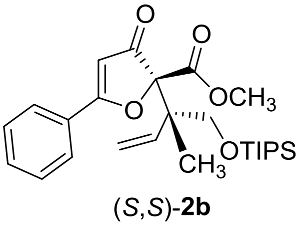

(*S*,*S*)-**2b**

Methyl-(*S*)-2-((*S*)-2-methyl-1-((triisopropylsilyl)oxy)but-3-en-2-yl)-3-oxo-5-phenyl-2,3-dihydrofuran-2-carboxylate;

0.1 mmol scale reaction;

colourless oil; 43.9 mg, 96% yield, 99% ee, 10:1 dr; $[a]_D^{25}$ = −75.8 (c 1.96, CH_2Cl_2); Determined by HPLC analysis [Daicel chiralpak IE, n-hexane/i-PrOH = 95/5, 1.0 mL/min, λ = 254 nm, t_1 = 23.60 min, t_2 = 24.90 min, t_3 = 27.02 min, t_4 = 29.92 min];

^{1}H NMR (400 MHz, $CDCl_3$) δ 7.86 (d, *J* = 7.2 Hz, 2H), 7.61-7.55 (m, 1H), 7.54 – 7.48 (m, 2H), 6.21 (dd, *J* = 17.6, 10.4 Hz, 1H), 5.97 (s, 1H), 5.21 (dd, *J* = 16.0, 12.0 Hz, 2H), 3.88 (d, *J* = 9.6 Hz, 1H), 3.89 –3.74 (m, 2H), 3.72 (s, 3H), 1.42 (s, 3H), 1.07 – 1.04 (m, 3H), 1.03 – 0.98 (m, 18H);

^{13}C NMR (101 MHz, $CDCl_3$) δ 196.3, 184.0, 165.2, 138.1, 132.8, 128.9, 128.5, 127.1, 116.2, 101.3, 92.6, 67.6, 52.9, 50.4, 18.0, 15.5, 12.0;

HRMS (ESI) calcd for $[M+H]^+$: $C_{26}H_{39}O_5Si$, m/z: 459.2561, observed: 459.2567.

Chiral HPLC spectrum of racemic **2b**

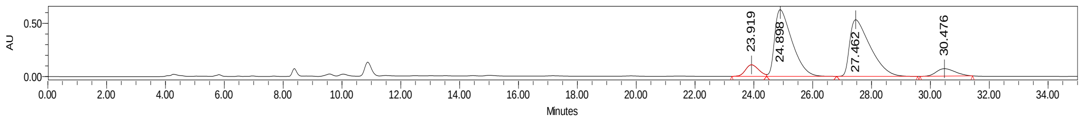

	Retention Time	Area	% Area
1	23.919	3367427	5.81
2	24.898	25755720	44.47
3	27.462	25684924	44.35
4	30.476	3110908	5.37

Chiral HPLC spectrum of cat-**2b** (*E*-**1b** as the starting material)

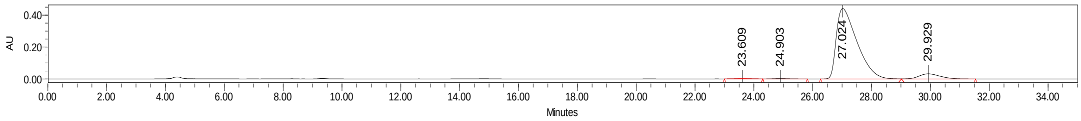

	Retention Time	Area	% Area
1	23.609	40854	0.18
2	24.903	46344	0.20
3	27.024	21431588	93.37
4	29.929	1433601	6.25

	Retention Time	Area	% Area
1	24.903	46344	0.22
2	27.024	21431588	99.78

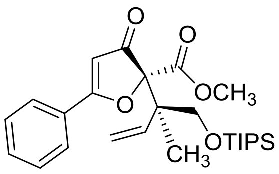

(*R*,*R*)-**2b**

Methyl-(*R*)-2-((*R*)-2-methyl-1-((triisopropylsilyl)oxy)but-3-en-2-yl)-3-oxo-5-phenyl-2,3-dihydrofuran-2-carboxylate;

0.1 mmol scale reaction;

colourless oil; 43.5 mg, 95% yield, 99% ee, 10:1 dr; Determined by HPLC analysis [Daicel chiralpak IE, n-hexane/i-PrOH = 95/5, 1.0 mL/min, λ = 254 nm, t_1 = 23.87 min, t_2 = 24.93 min, t_3 = 28.02 min, t_4 = 30.61 min];

HRMS (ESI) calcd for $[M+H]^+$: $C_{26}H_{39}O_5Si$, m/z: 459.2561, observed: 459.2566.

Ent-cat-**2b** (*E*-**1b** as the starting material)

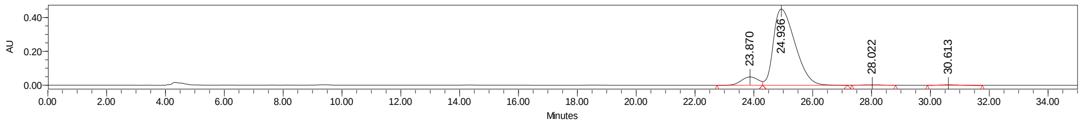

	Retention Time	Area	% Area
1	23.870	1964654	7.79
2	24.936	23052824	91.40
3	28.022	98262	0.39
4	30.613	104831	0.42

	Retention Time	Area	% Area
1	24.936	23052824	99.58
2	28.022	98262	0.42

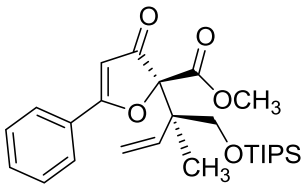

(*S*,*R*)-**2b'**

Methyl-(*S*)-2-((*R*)-2-methyl-1-((triisopropylsilyl)oxy)but-3-en-2-yl)-3-oxo-5-phenyl-2,3-dihydrofuran-2-carboxylate

0.1 mmol scale reaction;

colourless oil; 43.5 mg, 95% yield, 99% ee, 15:1 dr; $[a]_D^{25}$ = −170.6 (c 0.34, CH_2Cl_2); Determined by HPLC analysis [Daicel chiralpak IE, n-hexane/i-PrOH = 95/5, 1.0 mL/min, λ = 254 nm, t_1 = 23.82 min, t_2 = 25.33 min, t_3 = 27.81 min, t_4 = 29.87 min];

^{1}H NMR (400 MHz, $CDCl_3$) δ 7.89 – 7.83 (m, 2H), 7.62 – 7.56 (m, 1H), 7.58 – 7.55 (m, 2H), 6.00 (dd, *J* = 17.6, 11.2 Hz, 1H), 5.94 (s, 1H), 5.21 (dd, *J* = 17.4, 11.2 Hz, 2H), 3.98 (d, *J* = 9.6 Hz, 1H), 3.73 (s, 3H), 3.68 (d, *J* = 9.6 Hz, 1H), 1.50 (s, 3H), 1.06 (s, 21H);

^{13}C NMR (101 MHz, $CDCl_3$) δ 196.7, 184.4, 164.9, 137.4, 132.9, 128.9, 128.4, 127.2, 116.5, 101.2, 92.5, 68.0, 52.7, 49.7, 18.0, 15.5, 12.0;

HRMS (ESI) calcd for $[M+H]^+$: $C_{26}H_{39}O_5Si$, m/z: 459.2561, observed: 459.2560.

Chiral HPLC spectrum of cat-**2b** (*Z*-**1b'** as the starting material)

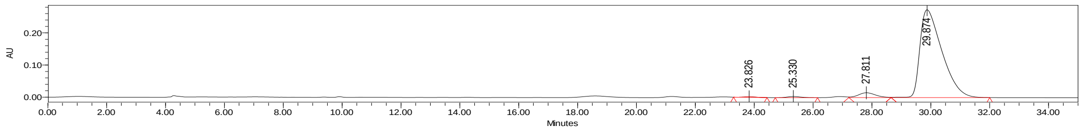

	Retention Time	Area	% Area
1	23.826	46295	0.31
2	25.330	108365	0.73
3	27.811	617452	4.14
4	29.874	14142251	94.82

	Retention Time	Area	% Area
1	23.826	44469	0.31
2	29.874	14142251	99.69

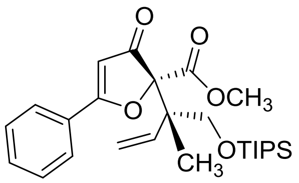

(*R*,*S*)-**2b'**

methyl-(R)-2-((S)-2-methyl-1-((triisopropylsilyl)oxy)but-3-en-2-yl)-3-oxo-5-phenyl-2,3-dihydrofuran-2-carboxylate

0.1 mmol scale reaction;

colourless oil; 43.5 mg, 95% yield, 99% ee, 15:1 dr; Determined by HPLC analysis [Daicel chiralpak IE, n-hexane/i-PrOH = 95/5, 1.0 mL/min, λ = 254 nm, t_1 = 23.51 min, t_2 = 25.02 min, t_3 = 26.75 min, t_4 = 30.20 min];

HRMS (ESI) calcd for $[M+H]^+$: $C_{26}H_{39}O_5Si$, m/z: 459.2560, observed: 459.2554.

Chiral HPLC spectrum of ent-cat-**2b** (*Z*-**1b'** as the starting material)

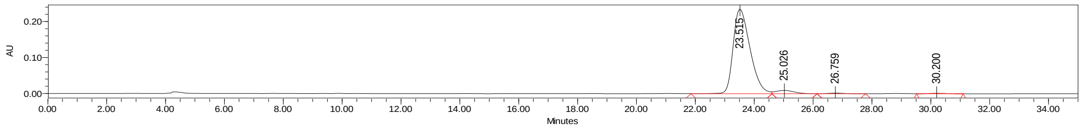

	Retention Time	Area	% Area

1	23.515	9359725	94.64
2	25.026	411624	4.16
3	26.759	74728	0.76
4	30.200	43461	0.44

	Retention Time	Area	% Area
1	23.515	9359725	99.54
2	30.200	43461	0.46

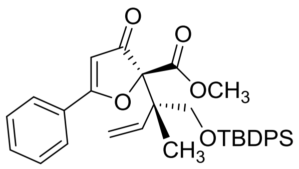

(*S*,*S*)-**2c**

Methyl-(S)-2-((S)-1-((*tert*-butyldiphenylsilyl)oxy)-2-methylbut-3-en-2-yl)-3-oxo-5-phenyl-2,3-dihydrofuran-2-carboxylate (2c)

0.1 mmol scale reaction;

colourless oil; 48.6 mg, 90% yield, 99%/93% ee, 5:1 dr; $[a]_D^{25} = -131$ (c 0.3, CH_2Cl_2); Determined by HPLC analysis [Daicel chiralpak IC, n-hexane/i-PrOH = 90/10, 1.0 mL/min, λ = 254 nm, t_1 = 13.26 min, t_2 = 14.90 min, t_3 = 17.41 min, t_4 = 19.74 min];

^{1}H NMR (400 MHz, $CDCl_3$) δ 7.84 (d, *J* = 7.2 Hz, 2H), 7.64 -7.59 (m, 3H), 7.51 (d, *J* = 8.0 Hz, 3H), 7.41 – 7.29 (m, 5H), 7.14 (t, *J* = 8.0 Hz, 2H), 6.15 (dd, *J* = 17.6, 11.2 Hz, 1H), 5.99 (s, 1H), 5.19 (dd, *J* = 17.6, 11.6 Hz, 2H), 3.81 (d, *J* = 10.0 Hz, 1H), 3.67 (s, 3H), 3.48 (d, *J* = 10.0 Hz, 1H), 1.47 (s, 3H), 1.01 (s, 9H);

^{13}C NMR (101 MHz, $CDCl_3$) δ 196.1, 183.8, 165.2, 138.0, 135.6, 133.1, 132.9, 132.8, 129.7, 129.5, 129.0, 128.5, 127.6, 127.5, 127.2, 116.4, 101.4 92.6, 67.8, 52.9, 50.4, 26.8, 19.3, 15.6;

HRMS (ESI) calcd for $[M+H]^+$: $C_{33}H_{37}O_5Si$, m/z: 541.2405, observed: 541.2410.

Chiral HPLC spectrum of racemic **2c**

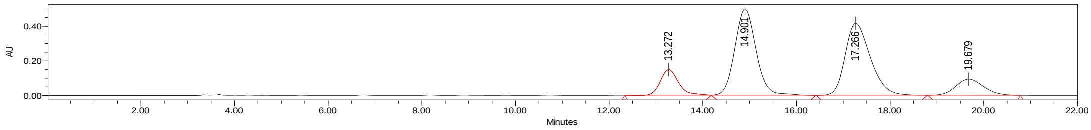

	Retention Time	Area	% Area
1	13.272	3997123	10.26
2	14.901	15663341	40.19
3	17.266	15487960	39.74
4	19.679	3826218	9.82

*Chiral HPLC spectrum of cat-***2c**

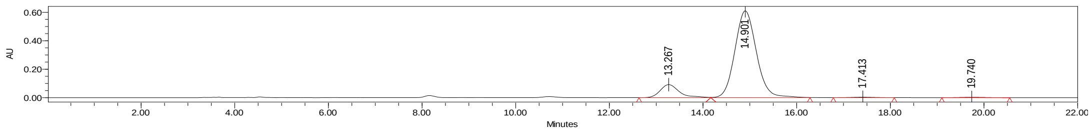

	Retention Time	Area	% Area
1	13.267	2565439	11.62
2	14.901	19330788	87.54
3	17.413	97640	0.44
4	19.740	88469	0.40

	Retention Time	Area	% Area
1	14.901	19330788	99.50
2	17.413	97640	0.50

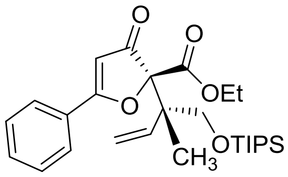

(*S*,*S*)-**2d**

Ethyl-(S)-2-((S)-2-methyl-1-((triisopropylsilyl)oxy)but-3-en-2-yl)-3-oxo-5-phenyl-2,3-dihydrofuran-2-carboxylate (**2d**)

0.1 mmol scale reaction;

colourless oil; 44.4 mg, 94% yield, 99%/96% ee, 5:1 dr; $[a]_D^{25} = -125.9$ (c 0.82, CH_2Cl_2); Determined by HPLC analysis [Daicel chiralpak IE, n-hexane/i-PrOH = 95/5, 1.0 mL/min, λ = 254 nm, t_1 = 14.42 min, t_2 = 16.25 min, t_3 = 18.29 min, t_4 = 19.53 min];

^{1}H NMR (400 MHz, $CDCl_3$) δ 7.88 – 7.84 (m, 2H), 7.55 – 7.54 (m, 1H), 7.54 – 7.47 (m, 2H), 6.17 (dd, *J* = 17.6, 11.2 Hz, 1H), 5.95 (s, 1H), 5.28 – 5.18 (m, 2H), 4.25 – 4.14 (m, 2H), 3.90 (d, *J* = 8.8 Hz, 1H), 3.72 (d, *J* = 8.8 Hz, 1H), 1.47 (s, 3H), 1.23 (t, *J* = 6.8 Hz, 3H), 1.04 – 0.94 (m, 21H);

^{13}C NMR (101 MHz, $CDCl_3$) δ 196.4, 183.9, 164.7, 138.3, 132.7, 128.9, 128.6, 127.11 (d, *J* = 4.8 Hz, 7H), 116.3, 101.3, 92.6, 67.5, 62.1, 50.4, 18.0, 15.5, 14.0, 12.0;

HRMS (ESI) calcd for $[M+H]^+$: $C_{27}H_{41}O_5Si$, m/z: 473.2718, observed: 473.2715.

Chiral HPLC spectrum of racemic **2d**

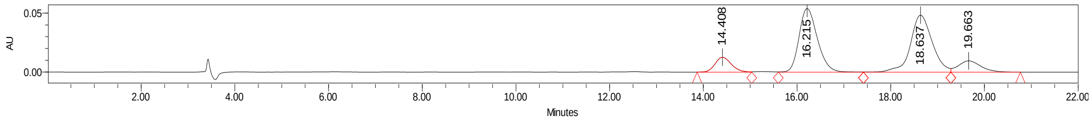

	Retention Time	Area	% Area
1	14.408	305501	8.37
2	16.215	1468058	40.24
3	18.637	1556003	42.65
4	19.663	318963	8.74

Chiral HPLC spectrum of cat-**2d**

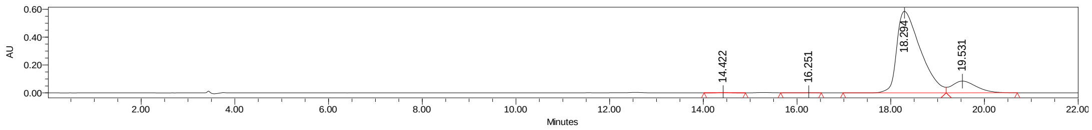

	Retention Time	Area	% Area
1	14.422	53305	0.22
2	16.251	20686	0.09
3	18.294	20570397	86.74
4	19.531	3071131	12.95

	Retention Time	Area	% Area
1	16.251	20686	0.10
2	18.294	20570397	99.90

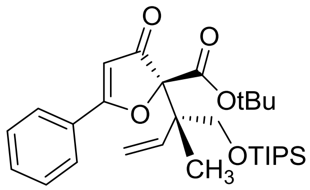

(*S*,*S*)-**2e**

Tert-butyl-(S)-2-((S)-2-methyl-1-((triisopropylsilyl)oxy)but-3-en-2-yl)-3-oxo-5-phenyl-2,3-dihydrofuran-2-carboxylate (**2e**)

0.1 mmol scale reaction;

colourless oil; 45 mg, 90% yield, 99%/98% ee, 1.5:1 dr; $[a]_D^{25}$ = −173.8 (c 0.18, CH_2Cl_2); Determined by HPLC analysis [Daicel chiralpak Lux 5u Cellulose-2, n-hexane/i-PrOH = 99/1, 1.0 mL/min, λ = 254 nm, t_1 = 9.43 min, t_2 = 10.65 min, t_3 = 12.89 min, t_4 = 14.76 min];

^{1}H NMR (400 MHz, $CDCl_3$) δ 7.85 (d, *J* = 7.6 Hz, 2H), 7.60 – 7.43 (m, 3H), 6.07 (dd, *J* = 17.6, 10.8 Hz, 1H), 5.92 (s, 1H), 5.20 (d, *J* = 12.4 Hz, 2H), 3.92 (d, *J* = 9.6 Hz, 1H), 3.66 (d, *J* = 9.6 Hz, 1H), 1.55 (s, 2H), 1.44 (s, 9H), 1.05 – 0.95 (dd, *J* = 7.2, 3.0 Hz, 21H);

^{13}C NMR (101 MHz, $CDCl_3$) δ 196.6, 183.6, 163.5, 138.6, 132.6, 128.9, 128.8, 127.1, 127.0, 116.3, 114.9, 101.3, 93.2, 92.9, 83.4, 68.0, 67.5, 50.3, 49.5, 27.9, 18.0, 15.6, 12.0;

HRMS (ESI) calcd for $[M+H]^+$: $C_{29}H_{45}O_5Si$, m/z: 501.3031, observed: 501.3018;

Chiral HPLC spectrum of racemic **2e**

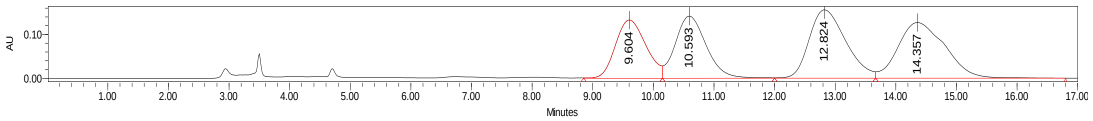

	Retention Time	Area	% Area
1	9.604	4638675	20.13
2	10.593	5029922	21.83
3	12.824	6547834	28.41
4	14.357	6828957	29.63

Chiral HPLC spectrum of cat-**2e**

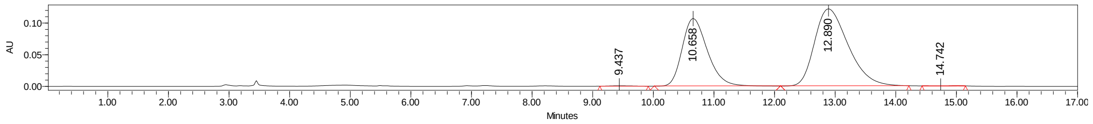

	Retention Time	Area	% Area
1	9.437	16037	0.21
2	10.658	3063853	39.44
3	12.890	4681479	60.27
4	14.742	6234	0.08

	Retention Time	Area	% Area
1	12.890	4681479	99.87
2	14.742	6234	0.13

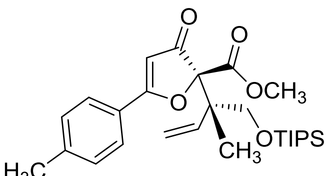

2f

Methyl-(*S*)-2-((*S*)-2-methyl-1-((triisopropylsilyl)oxy)but-3-en-2-yl)-3-oxo-5-(p-tolyl)-2,3-dihydrofuran-2-carboxylate;

0.19 mmol scale reaction;

colourless oil; 86 mg, 95% yield, 99%/96% ee, 9:1 dr; $[a]_D^{25}$ = −121.6 (c 0.91, CH_2Cl_2); Determined by HPLC analysis [Daicel chiralpak IE, n-hexane/i-PrOH = 95/5, 1.0 mL/min, λ = 254 nm, t_1 = 30.91 min, t_2 =

32.67 min, t_3 = 42.17 min, t_4 = 46.3 min];

^{1}H NMR (400 MHz, $CDCl_3$) δ 7.75 (d, *J* = 8.2 Hz, 2H), 7.30 (d, *J* = 8.0 Hz, 2H), 6.21 (dd, *J* = 18.0, 10.8 Hz, 1H), 5.91 (s, 1H), 5.21 (dd, *J* = 15.6, 12.0 Hz, 2H), 3.87 (d, *J* = 9.6 Hz, 1H), 3.74 (d, *J* = 9.6 Hz, 1H), 3.71 (s, 3H), 2.43 (s, 3H), 1.40 (s, 3H), 1.05-0.96 (m, 21H);

^{13}C NMR (101 MHz, $CDCl_3$) δ 195.2, 183.3, 164.3, 142.7, 137.2, 128.6, 126.1, 124.7, 115.1, 99.6, 91.5, 66.5, 51.8, 49.3, 20.7, 16.9, 14.5, 11.0;

HRMS (ESI) calcd for $[M+H]^+$:$C_{27}H_{41}O_5Si$, m/z: 473.2723, observed: 473.2726.

Chiral HPLC spectrum of racemic **2f:**

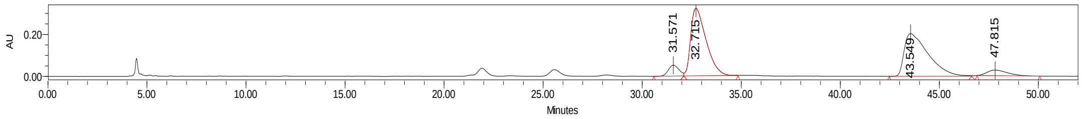

	Retention Time	Area	% Area
1	31.571	1959036	5.12
2	32.715	17353700	45.34
3	43.549	17054420	44.56
4	47.815	1906625	4.98

*Chiral HPLC spectrum of cat-***2f:**

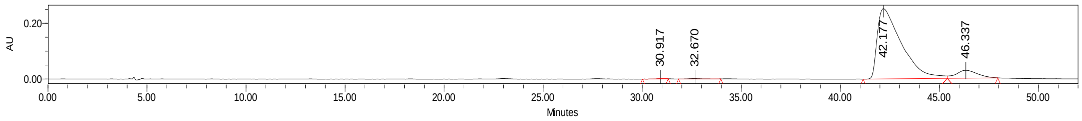

	Retention Time	Area	% Area
1	30.917	50950	0.21
2	32.670	53652	0.22
3	42.177	21565624	90.38
4	46.337	2190474	9.18

	Retention Time	Area	% Area
1	32.670	68856	0.32
2	42.177	21413962	99.68

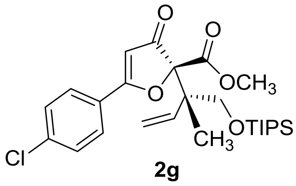

2g

Methyl-(*S*)-5-(4-chlorophenyl)-2-((*S*)-2-methyl-1-((triisopropylsilyl) oxy)but-3-en-2-yl)-3-oxo-2,3-dihydrofuran-2-carboxylate;

0.3 mmol scale reaction;

colourless oil; 142 mg, 95% yield, 99%/98% ee, 8:1 dr; $[a]_D^{25}$ = −137.8 (c 1.06, CH_2Cl_2); Determined by HPLC analysis [Daicel chiralpak ADH, n-hexane/i-PrOH = 97/3, 1.0 mL/min, λ = 254 nm, t_1 = 6.76 min, t_2 = 7.88 min, t_3 = 8.60 min, t_4 = 9.63 min];

^{1}H NMR (400 MHz, $CDCl_3$) δ 7.80 (d, *J* = 8.0 Hz, 2H), 7.49 (d, *J* = 8.0 Hz, 2H), 6.18 (dd, *J* = 18.0, 10.8 Hz, 1H), 5.96 (s, 1H) , 5.23 (dd, *J* = 16.4, 12.0 Hz, 2H), 3.88 (d, *J* = 9.6 Hz, 1H), 3.73 (s, 3H), 3.70 (d, *J* = 9.6 Hz, 1H), 1.44 (s, 3H), 1.01 (s, 21H);

^{13}C NMR (101 MHz, $CDCl_3$) δ 196.0, 182.6, 165.0, 139.0, 138.0, 129.3, 128.3, 127.0, 116.3, 101.5, 92.7, 67.6, 52.9, 50.4, 17.9, 15.5, 12.0;

HRMS (ESI) calcd for $[M+H]^+$:$C_{26}H_{38}O_5SiCl$,m/z: 493.2177, 495.2148, observed: 493.2177,

495.2178.

Chiral HPLC spectrum of racemic **2g:**

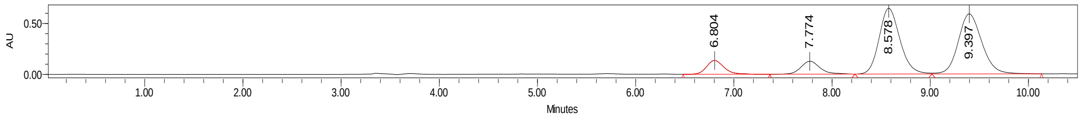

	Retention Time	Area	% Area
1	6.804	1671811	7.35
2	7.774	1726118	7.58
3	8.578	9523280	41.84
4	9.397	9838022	43.23

Chiral HPLC spectrum of cat-**2g:**

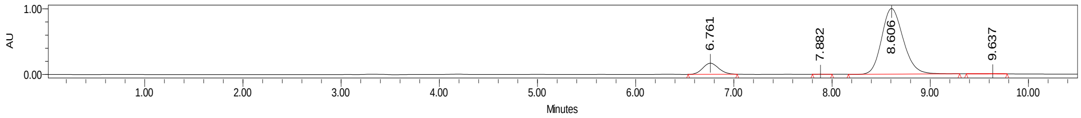

	Retention Time	Area	% Area
1	6.761	1909346	11.14
2	7.882	11875	0.07
3	8.606	15188559	88.66
4	9.637	22296	0.13

	Retention Time	Area	% Area
1	8.606	15188559	99.85
2	9.637	22296	0.15

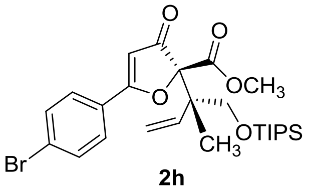

2h

Methyl-(*S*)-5-(4-bromophenyl)-2-((*S*)-2-methyl-1-((triisopropylsilyl) oxy)but-3-en-2-yl)-3-oxo-2,3-dihydrofuran-2-carboxylate;

0.27 mmol scale reaction;

colourless oil; 134 mg, 92% yield, 99%/96% ee, 9:1 dr; $[a]_D^{25} = -131.9$ (c 0.63, CH_2Cl_2); Determined by HPLC analysis [Daicel chiralpak ADH, n-hexane/i-PrOH = 97/3, 1.0 mL/min, λ = 254 nm, t_1 = 6.83 min, t_2 = 7.72 min, t_3 = 8.74 min, t_4 = 9.90 min];

^{1}H NMR (400 MHz, $CDCl_3$) δ 7.72 (d, *J* = 8.4 Hz, 2H), 7.64 (d, *J* = 8.4 Hz, 2H), 6.16 (dd, *J* = 17.6, 10.4 Hz, 1H), 5.95 (s, 1H), 5.22 (dd, *J* = 15.6, 11.6 Hz, 2H), 3.86 (d, *J* = 9.6 Hz, 1H), 3.71 (s, 3H), 3.68 (d, *J* = 9.6 Hz, 1H), 1.42 (s, 3H), 1.05-0.96 (m, 21H);

^{13}C NMR (101 MHz, $CDCl_3$) δ 196.0, 182.7, 165.0, 138.0, 132.3, 128.4, 127.6, 127.4, 116.4, 101.6, 92.8, 67.6, 52.9, 50.4, 18.0, 15.5, 12.0;

HRMS (ESI) calcd for $[M+H]^+$: $C_{26}H_{38}O_5SiBr$, m/z: 537.1672, 539.1651, observed: 537.1671, 539.1655.

Chiral HPLC spectrum of racemic **2h**:

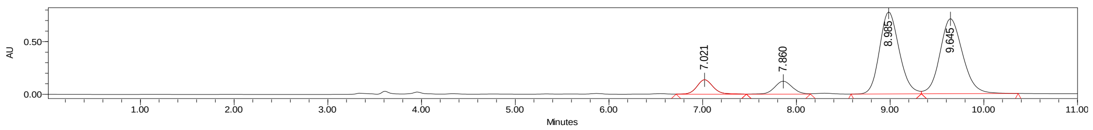

	Retention Time	Area	% Area
1	7.021	1645496	6.20
2	7.860	1608050	6.06
3	8.985	11329461	42.67
4	9.645	11967054	45.07

*Chiral HPLC spectrum of cat-***2h**:

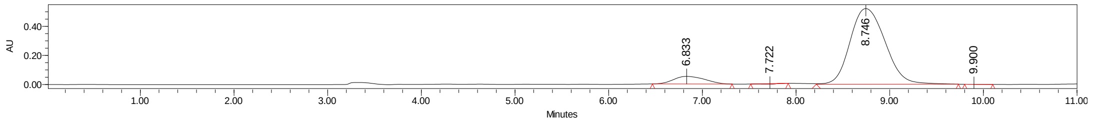

	Retention Time	Area	% Area
1	6.833	1215961	8.25
2	7.722	25423	0.17
3	8.746	13501679	91.56
4	9.900	3284	0.02

	Retention Time	Area	% Area
1	8.746	13501679	99.98
2	9.900	3284	0.02

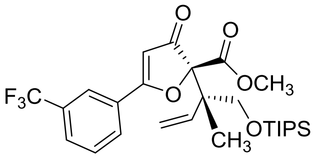

2i

Methyl-(*S*)-2-((*S*)-2-methyl-1-((triisopropylsilyl)oxy)but-3-en-2-yl)-3-oxo-5-(3-(trifluoromethyl)phenyl)-2,3-dihydrofuran-2-carboxylate; 0.41 mmol scale reaction; colourless oil; 200 mg, 92% yield, 99%/96% ee, 8:1 dr; $[a]_D^{25} = -120.9$ (c 0.86, CH_2Cl_2); Determined by HPLC analysis [Daicel chiralpak ADH, n-hexane/i-PrOH = 97/3, 1.0 mL/min, λ = 254 nm, t_1 = 4.99 min, t_2 = 5.66 min, t_3 = 6.81 min, t_4 = 7.75 min];

^{1}H NMR (400 MHz, $CDCl_3$) δ 8.09 (s, 1H), 8.02 (d, *J* = 7.6 Hz, 1H), 7.82 (d, *J* = 8.0 Hz, 1H), 7.66 (t, *J* = 7.6 Hz, 1H), 6.21 (dd, *J* = 17.6, 10.8 Hz, 1H), 6.03 (s, 1H), 5.23 (dd, *J* = 17.2, 11.2 Hz, 2H),, 3.87 (d, *J* = 9.6 Hz, 1H), 3.72 (s, 3H), 3.69 (d, *J* = 9.6 Hz, 1H), 1.41 (s, 3H), 1.05-0.96 (m, 21H);

^{13}C NMR (101 MHz, $CDCl_3$) δ 195.97, 182.00, 164.88, 137.90, 131.61 (d, *J* = 33 Hz), 130.29, 129.63, 129.41, 129.10 (q, *J* = 4 Hz), 123.59 (q, *J* = 4 Hz), 123.5 (q, J = 271Hz), 116.30, 102.31, 92.93, 67.83, 52.92, 50.43, 17.87, 15.56, 11.96;

HRMS (ESI) calcd for $[M+H]^+$: $C_{27}H_{38}O_5SiF_3$, m/z: 527.2441, observed: 527.2440.

Chiral HPLC spectrum of racemic **2i**:

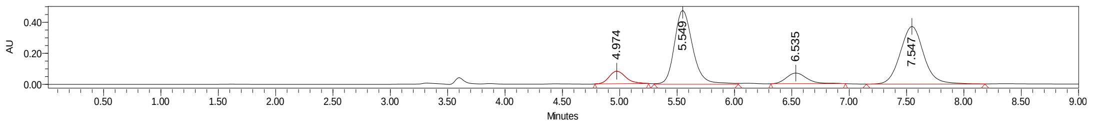

	Retention Time	Area	% Area
1	4.974	945055	7.55
2	5.549	5289133	42.28
3	6.535	993976	7.95
4	7.547	5281761	42.22

*Chiral HPLC spectrum of cat-***2i**:

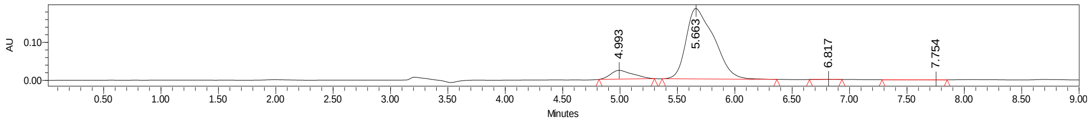

	Retention Time	Area	% Area
1	4.993	310984	8.97
2	5.663	3145809	90.77
3	6.817	5352	0.15
4	7.754	3678	0.11

	Retention Time	Area	% Area
1	5.663	3151896	99.50
2	7.754	15866	0.50

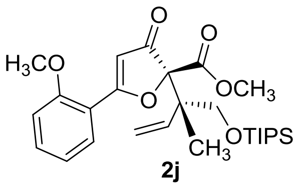

2j

Methyl-(*S*)-5-(4-methoxyphenyl)-2-((*S*)-2-methyl-1-((triisopropylsilyl)oxy)but-3-en-2-yl)-3-oxo-2,3-dihydrofuran-2-carboxylate;

0.1 mmol scale reaction;

colourless oil; 48 mg, 99% yield, 99% ee, 12:1 dr; [a]$_D^{25}$ = −51.3 (c 0.46, CH_2Cl_2); Determined by HPLC analysis [Daicel chiralpak ADH, n-hexane/i-PrOH = 97/3, 1.0 mL/min, λ = 254 nm, t_1 = 6.94 min, t_2 = 8.05 min, t_3 = 9.85 min, t_4 = 11.28 min];

^{1}H NMR (400 MHz, $CDCl_3$) δ 7.47 – 7.35 (m, 3H), 7.14 – 7.08 (m, 1H), 6.22 (dd, *J* = 11.6, 17.2 Hz, 1H), 5.94 (s, 1H), 5.22 (dd, *J* = 10.4, 17.2 Hz, 1H), 3.87 (s, 3H), 3.85 (d, *J* = 9.6 Hz, 1H),3.75 (d, *J* = 9.6 Hz, 1H), 3.72(s, 3H), 1.40 (s, 3H), 1.07 – 0.99 (m, 21H);

^{13}C NMR (101 MHz, $CDCl_3$) δ 196.3, 183.9, 165.2, 159.9, 138.1, 130.0, 129.7, 119.8, 118.5, 116.2, 112.1, 101.6, 92.6, 67.6, 55.5, 52.9, 50.3, 18.0, 15.5, 12.0;

HRMS (ESI) calcd for $[M+H]^+$: $C_{27}H_{41}O_5Si$, m/z: 489.2672, observed: 489.2672.

Chiral HPLC spectrum of racemic **2j**:

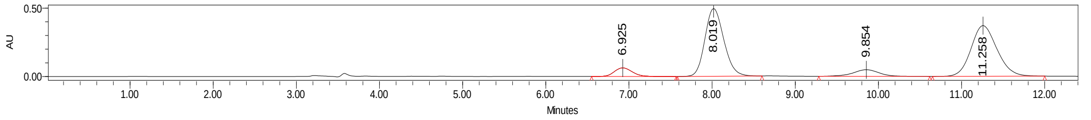

	Retention Time	Area	% Area
1	6.925	950595	5.32
2	8.019	7814845	43.76
3	9.854	1052350	5.89
4	11.258	8041009	45.03

*Chiral HPLC spectrum of cat-***2j**:

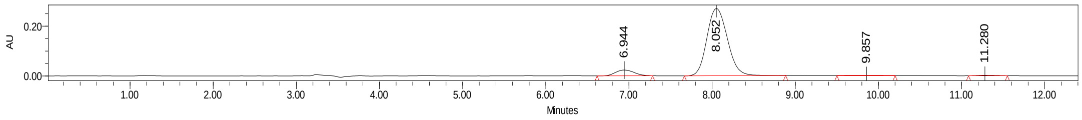

	Retention Time	Area	% Area
1	6.944	359845	7.17
2	8.052	4623556	92.09
3	9.857	11538	0.23
4	11.280	25950	0.52

	Retention Time	Area	% Area
1	8.052	4623556	99.44
2	11.280	25950	0.56

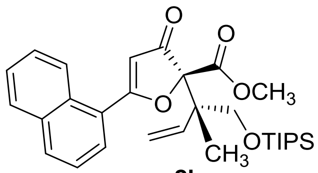

2k

Methyl-(*S*)-2-((*S*)-2-methyl-1-((triisopropylsilyl)oxy)but-3-en-2-yl)-5-(naphthalen-1-yl)-3-oxo-2,3-dihydrofuran-2-carboxylate;

0.1 mmol scale reaction;

colourless oil; 48.9 mg, 96% yield, 99%/98% ee, 9:1 dr; $[a]_D^{25}$ = −227.3 (c 0.84, CH_2Cl_2); Determined by HPLC analysis [Daicel chiralpak Lux 5u Cellulose-2, n-hexane/i-PrOH = 95/5, 1.0 mL/min, λ = 254 nm, t_1 = 15.86 min, t_2 = 17.01 min, t_3 = 18.44 min, t_4 = 21.02 min];

^{1}H NMR (400 MHz, $CDCl_3$) δ8.62 (d, *J* = 8.4 Hz, 1H), 8.04 (d, *J* = 8.0 Hz, 1H), 7.98 – 7.90 (m, 2H), 7.65 – 7.53 (m, 3H), 6.30 (dd, *J* = 10.8, 17.2 Hz,1H), 6.02 (s, 1H), 5.22 (dd, *J* = 10.4, 17.2 Hz, 1H), 3.87 (d, *J* = 9.6 Hz, 2H), 3.81 (s, 3H), 1.41 (s, 3H), 1.08 – 0.99 (m, 21H);

^{13}C NMR (101 MHz, $CDCl_3$) δ 196.3, 186.0, 165.2, 138.0, 133.8, 133.0, 130.3, 128.8, 128.3, 127.8, 126.6, 125.3, 124.9, 116.3, 105.9, 92.7, 67.5, 53.0, 50.2, 18.0, 15.6, 12.0;

HRMS (ESI) calcd for $[M+H]^+$: $C_{30}H_{41}O_5Si$, m/z: 509.2723, observed: 509.2722.

Chiral HPLC spectrum of racemic **2k**:

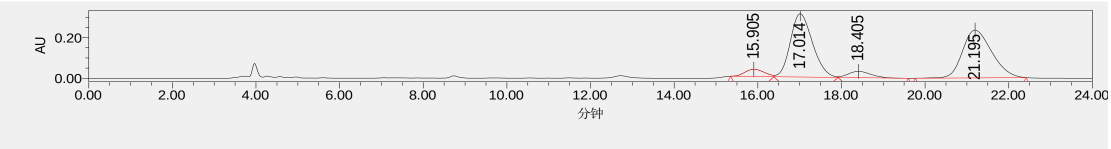

	Retention Time	Area	% Area
1	15.905	1093204	4.35
2	17.014	11407164	45.35
3	18.405	1170886	4.66
4	21.195	11480865	45.65

*Chiral HPLC spectrum of cat-***2k**:

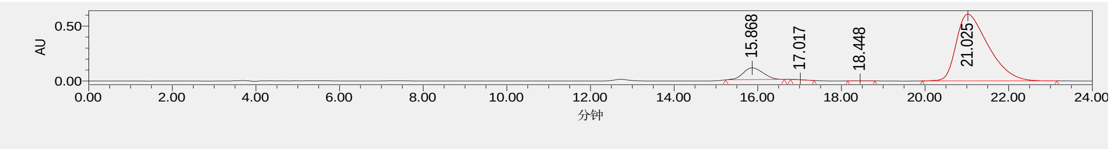

	Retention Time	Area	% Area
1	15.868	3524667	9.83
2	17.017	22455	0.06
3	18.448	38467	0.11
4	21.025	32288537	90.01

	Retention Time	Area	% Area
1	17.017	22455	0.07
2	21.025	32288537	99.93

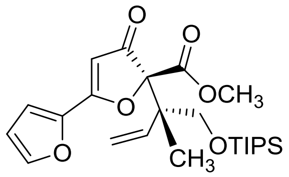

2l

Methyl-(*S*)-5-((*S*)-2-methyl-1-((triisopropylsilyl)oxy)but-3-en-2-yl)-4-oxo-4,5-dihydro-[2,2'-bifuran]-5-carboxylate;

0.34 mmol scale reaction;

colourless oil; 149 mg, 97% yield, 99%/98% ee, 8:1 dr; $[a]_D^{25} = -130.1$ (c 0.89, CH_2Cl_2); Determined by HPLC analysis [Daicel chiralpak ODH, n-hexane/i-PrOH = 99/1, 0.8 mL/min, λ = 254 nm, t_1 = 13.06 min, t_2 = 14.76 min, t_3 = 15.47 min, t_4 = 16.53 min];

^{1}H NMR (400 MHz, $CDCl_3$) δ 7.64 (s, 1H), 7.14 (d, *J* = 3.6 Hz, 1H), 6.58 (dd, *J* = 3.2, 1.2 Hz, 1H), 6.14 (dd, *J* = 18.0, 10.8 Hz, 1H), 5.80 (s, 1H), 5.16 (dd, *J* = 16.8, 11.6 Hz, 2H), 3.82 (d, *J* = 9.6 Hz, 1H), 3.68 (s, 3H), 3.65 (d, *J* = 9.6 Hz, 1H), 1.36 (s, 3H), 1.02-0.93 (m, 21H);

^{13}C NMR (101 MHz, $CDCl_3$) δ 195.3, 174.2, 165.1, 147.1, 144.6, 138.1, 116.1, 115.1, 112.6, 100.1, 92.2, 67.7, 52.8, 50.3, 17.9, 15.5, 11.9;

HRMS (ESI) calcd for $[M+H]^+$: $C_{24}H_{37}O_6Si$, m/z: 449.2359, observed: 449.2362.

Chiral HPLC spectrum of racemic **2l**:

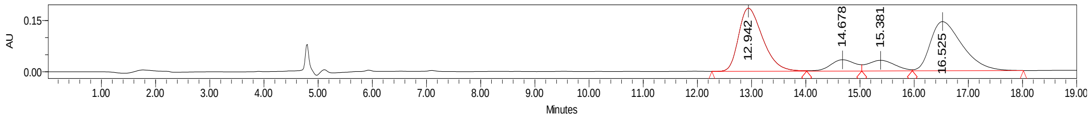

	Retention Time	Area	% Area
1	12.942	5710051	41.68
2	14.678	1048876	7.66
3	15.381	1088782	7.95
4	16.525	5852062	42.72

*Chiral HPLC spectrum of cat-***2l**:

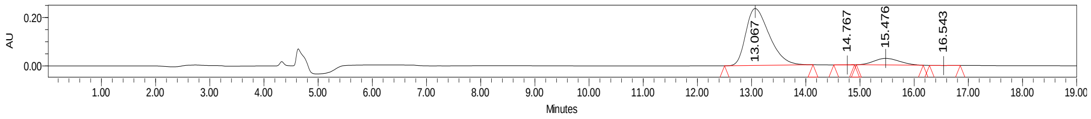

	Retention Time	Area	% Area
1	13.067	7084846	88.70
2	14.767	4704	0.06
3	15.476	890046	11.14

4	16.543	7580	0.09

	Retention Time	Area	% Area
1	13.067	7084846	99.89
2	16.543	7580	0.11

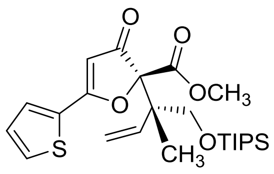

2m

Methyl-(*S*)-2-((*S*)-2-methyl-1-((triisopropylsilyl)oxy)but-3-en-2-yl)-3-oxo-5-(thiophen-2-yl)-2,3-dihydrofuran-2-carboxylate;

0.41 mmol scale reaction;

colourless oil; 180 mg, 95% yield, 99%/90% ee, 9:1 dr; $[a]_D^{25}$ = −100.1 (c 0.60, CH_2Cl_2); Determined by HPLC analysis [Daicel chiralpak Lux 5u Cellulose-2, n-hexane/i-PrOH = 95/5, 0.8 mL/min, λ = 254 nm, t_1 = 20.12 min, t_2 = 21.36 min, t_3 = 28.55 min, t_4 = 53.03 min];

^{1}H NMR (400 MHz, $CDCl_3$) δ 7.70 (d, *J* = 3.2 Hz, 1H), 7.66 (d, *J* = 4.8 Hz, 1H), 7.21 – 7.17 (m, 1H), 6.19 (dd, *J* = 18.0, 10.8 Hz, 1H), 5.79 (s, 1H), 5.20 (dd, *J* = 14.8, 11.6 Hz, 2H), 3.85 (d, *J* = 9.6 Hz, 1H), 3.72 (d, *J* = 9.6 Hz, 1H),, 3.70 (s, 3H), 1.38 (s, 3H), 1.05-0.96 (m, 21H);

^{13}C NMR (101 MHz, $CDCl_3$) δ 195.3, 138.1, 132.1, 131.4, 130.7, 128.6, 116.1, 100.0, 92.6, 67.7, 52.8, 50.3, 17.9, 15.6, 12.0;

HRMS (ESI) calcd for $[M+H]^+$: $C_{24}H_{37}O_5SiS$, m/z: 465.2131, observed: 465.2129.

Chiral HPLC spectrum of racemic **2m**:

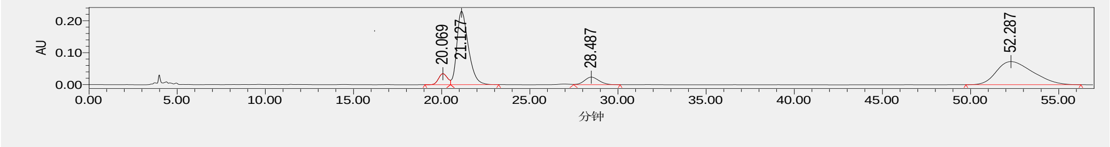

	Retention Time	Area	% Area
1	20.069	1216878	5.21
2	21.127	10399976	44.55
3	28.487	1353807	5.80
4	52.287	10374611	44.44

*Chiral HPLC spectrum of cat-***2m**:

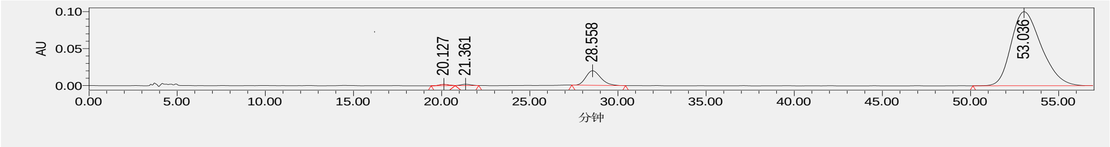

	Retention Time	Area	% Area
1	20.127	67570	0.51
2	21.361	72069	0.54
3	28.558	1142231	8.63
4	53.036	11950112	90.31

	Retention Time	Area	% Area
1	21.361	72069	0.60
2	53.036	11950112	99.40

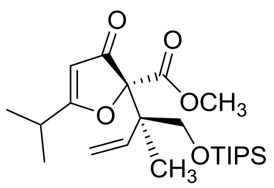

(*R*,*R*)-**2n**

Methyl-(*R*)-5-isopropyl-2-((*R*)-2-methyl-1-((triisopropylsilyl)oxy)but-3-en-2-yl)-3-oxo-2,3-dihydrofuran-2-carboxylate;

0.1 mmol scale reaction;

colourless oil; 40.7 mg, 96% yield, 99%/92% ee, 9:1 dr; $[a]_D^{25}$ = 43.6 (c 0.80, CH_2Cl_2); Determined by HPLC analysis [Daicel chiralpak IE, n-hexane/i-PrOH = 99/1, 1.0 mL/min, λ = 254 nm, t_1 = 39.70 min, t_2 = 46.73 min, t_3 = 52.61 min];

^{1}H NMR (400 MHz, $CDCl_3$) δ 6.12 (dd, *J* = 17.6, 11.2 Hz, 1H),5.35 (s, 1H), 5.21 (dd, *J* = 17.6, 12.0 Hz, 2H), 3.81 – 3.74 (m, 2H), 3.71 (s, 3H), 2.83 – 2.76 (m, 1H), 1.31 (s, 3H), 1.27 (d, *J* = 6.8 Hz, 6H), 1.06 – 1.03(m, 21H);

^{13}C NMR (101 MHz, $CDCl_3$) δ 197.3, 197.0, 165.1, 138.0, 116.1, 101.7, 92.2, 67.0, 52.8, 49.7, 30.2, 19.4, 19.3, 18.0, 15.4, 12.0;

HRMS (ESI) calcd for $[M+H]^+$: $C_{23}H_{41}O_5Si$, m/z: 425.2723, observed: 425.2720.

Chiral HPLC spectrum of racemic **2n** (two peaks overlap)**:**

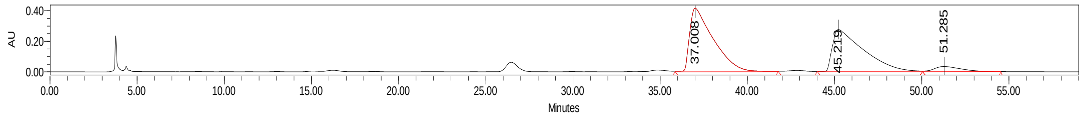

	Retention Time	Area	% Area
1	37.008	39462862	50.94
2	45.219	34423826	44.43
3	51.285	3589409	4.63

*Chiral HPLC spectrum of ent-cat-***2n** (*E*-**1n** as the starting material, for analysis we did the *ent*-catalytic experiments):

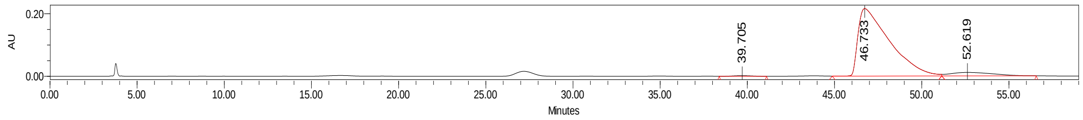

	Retention Time	Area	% Area
1	39.705	148078	0.51
2	46.733	27186248	93.08
3	52.619	1871815	6.41

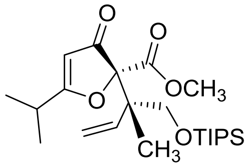

(*R*,*S*)-**2n'**

Methyl-(*R*)-5-isopropyl-2-((S)-2-methyl-1-((triisopropylsilyl)oxy)but-3-en-2-yl)-3-oxo-2,3-dihydrofuran-2-carboxylate

0.1 mmol scale reaction;

colourless oil; 36.0 mg, 85% yield, 98% ee, 16:1 dr; $[a]_D^{25}$ = 94.5 (c 0.42, CH_2Cl_2); Determined by HPLC analysis [Daicel chiralpak IE, n-hexane/i-PrOH = 99/1, 1.0 mL/min, λ = 254 nm, t_1 = 38.15 min, t_2 = 44.76 min, t_3 = 46.11 min];

^{1}H NMR (400 MHz, $CDCl_3$) 5.90 (dd, *J* = 16.8, 11.6 Hz, 1H), 5.32 (s, 1H), 5.13 (dd, *J* = 17.2, 11.6 Hz, 1H), 3.88 (d, *J* = 9.2 Hz, 1H), 3.71 (s, 3H), 3.63 (d, *J* = 9.2 Hz, 1H), 2.89 – 2.71 (m, 1H), 1.43 (s, 3H), 1.27 (d, *J* = 7.2 Hz, 6H), 1.06 (s, 21H).

^{13}**C NMR** (101 MHz, $CDCl_3$) δ 197.49, 197.48, 164.97, 137.48, 116.36, 101.67, 92.01, 67.58, 52.65, 49.13, 30.16, 19.45, 19.28, 17.96, 17.93, 15.37, 11.99;

HRMS (ESI) calcd for $[M+H]^+$: $C_{23}H_{41}O_5Si$, m/z: 425.2723, observed: 425.2718.

*Chiral HPLC spectrum of ent-cat--***2n** (*Z*-**1n'** as the starting material, for analysis we did the *ent*-catalytic experiments):

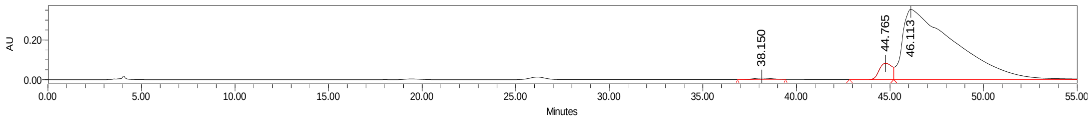

	Retention Time	Area	% Area
1	38.150	485827	0.65
2	44.765	3835850	5.17
3	46.113	69928943	94.18

	Retention Time	Area	% Area
1	38.150	485827	0.69
2	46.113	69928943	99.31

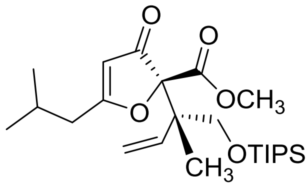

2o

Methyl-(*S*)-5-isobutyl-2-((*S*)-2-methyl-1-((triisopropylsilyl)oxy)but-3-en-2-yl)-3-oxo-2,3-dihydrofuran-2-carboxylate;

0.1 mmol scale reaction;

colourless oil; 41.5 mg, 95% yield, 99%/97% ee, 8:1 dr; $[a]_D^{25}$ = −11.6 (c 0.44, CH_2Cl_2); Determined by HPLC analysis [Daicel chiralpak IE, n-hexane/i-PrOH = 99/1, 1.0 mL/min, λ = 254 nm, t_1 = 27.37 min, t_2 = 29.24 min, t_3 = 36.73 min, t_4 = 41.78 min];

^{1}**H NMR** (400 MHz, $CDCl_3$) δ 6.11 (dd, *J* = 17.2, 11.8 Hz, 1H), , 5.36 (s, 1H), 5.18 (dd, *J* = 20.0, 11.6 Hz, 2H), 3.88 – 3.72 (m, 2H), 3.71 –3.62 (m, 3H), 2.42 (d, *J* = 7.2 Hz, 2H), 2.18 – 2.02 (m, 1H), 1.32 (s, 3H), 1.16 – 0.71 (m, 27H);

^{13}**C NMR** (101 MHz, $CDCl_3$) δ 197.0, 192.0, 165.1, 138.0, 116.1, 104.7, 67.0, 52.7, 49.7, 39.7, 26.6, 22.6, 22.4, 18.0, 15.4, 12.0;

HRMS (ESI) calcd for $[M+H]^+$: $C_{24}H_{43}O_5Si$, m/z: 439.2880, observed: 439.2885.

Chiral HPLC spectrum of racemic **2o**:

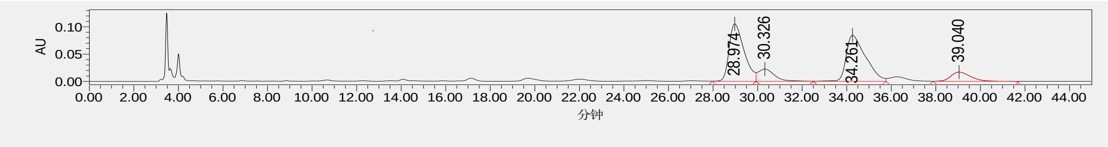

	Retention Time	Area	% Area
1	28.974	4934627	40.43
2	30.326	1148279	9.41
3	34.261	5087732	41.68
4	39.040	1035279	8.48

*Chiral HPLC spectrum of cat-***2o**:

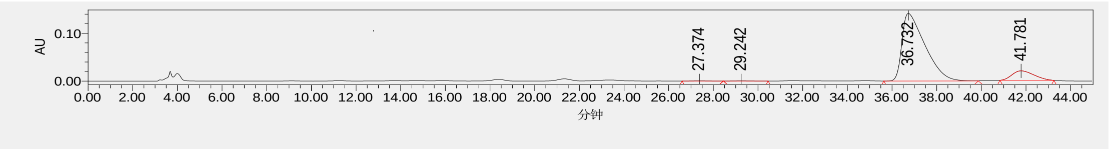

	Retention Time	Area	% Area
1	27.374	19765	0.17
2	29.242	22802	0.20
3	36.732	10099281	87.82
4	41.781	1358017	11.81

	Retention Time	Area	% Area
1	27.374	19765	0.20
2	36.732	10099281	99.80

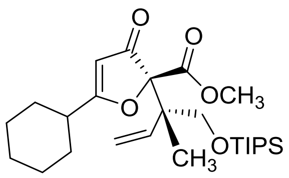

2p

Methyl-(*S*)-5-cyclohexyl-2-((*S*)-2-methyl-1-((triisopropylsilyl)oxy)but-3-en-2-yl)-3-oxo-2,3-dihydrofuran-2-carboxylate;

0.1 mmol scale reaction;

colourless oil; 43.6 mg, 94% yield, 98%/90% ee, 9:1 dr; $[a]_D^{25}$ = −23.8 (c 0.75, CH_2Cl_2); Determined by HPLC analysis [Daicel chiralpak IE, n-hexane/i-PrOH = 99/1, 1.0 mL/min, λ = 254 nm, t_1 = 46.95 min, t_2 = 54.46 min, t_3 = 64.77 min];

^{1}H NMR (400 MHz, $CDCl_3$) δ 6.10 (dd, *J* = 17.6, 10.8 Hz, 1H), 5.31 – 5.08 (m, 3H), 3.83 – 3.73 (m, 2H), 3.71 (s, 3H), 2.52 – 2.48 (m, 1H), 2.00 (s, 2H), 1.83 (d, *J* = 11.6 Hz, 2H), 1.71 (d, *J* = 12.4 Hz, 1H), 1.47 – 1.25 (m, 8H), 1.04 (s, 21H);

^{13}C NMR (101 MHz, $CDCl_3$) δ 197.1, 196.3, 165.2, 138.0, 116.1, 101.9, 92.0, 67.0, 52.8, 49.7, 39.4, 29.8, 29.5, 25.7, 25.5, 18.0, 15.4, 12.0;

HRMS (ESI) calcd for $[M+H]^+$: $C_{26}H_{45}O_5Si$, m/z: 465.3036, observed: 465.3034.

Chiral HPLC spectrum of racemic **2p** (two peaks overlap):

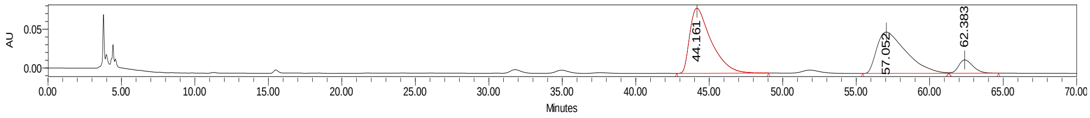

	Retention Time	Area	% Area
1	44.161	8479473	49.81
2	57.052	7374754	43.32
3	62.383	1170194	6.87

Chiral HPLC spectrum of cat-**2p**:

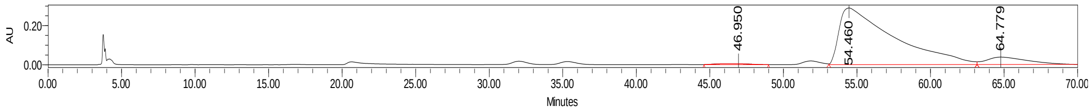

	Retention Time	Area	% Area
1	46.950	742714	0.90

2	54.460	74862354	90.27
3	64.779	7329094	8.84

	Retention Time	Area	% Area
1	46.950	742714	0.98
2	54.460	74862354	99.02

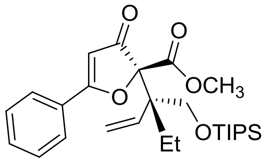

2q

Methyl-(S)-3-oxo-5-phenyl-2-((S)-3-(((triisopropylsilyl)oxy)methyl)pent-1-en-3-yl)-2,3-dihydrofuran-2-carboxylate;

0.1 mmol scale reaction;

colourless oil; 40 mg, 85% yield, 99% ee, 13:1 dr; $[a]_D^{25}$ = −263.5 (c 0.15, CH_2Cl_2); Determined by HPLC analysis [Daicel chiralpak IE, n-hexane/i-PrOH = 95/5, 1.0 mL/min, λ = 254 nm, t_1 = 18.60 min, t_2 = 21.96 min, t_3 = 23.82 min, t_4 = 28.66 min];

^{1}H NMR (400 MHz, $CDCl_3$) δ 7.91 – 7.80 (m, 2H), 7.64 – 7.53 (m, 1H), 7.53 – 7.45 (m, 2H), 6.06 (dd, *J* = 17.6, 11.2 Hz, 1H), 5.95 (s, 1H), 5.27 (d, *J* = 11.2 Hz, 1H), 5.15 (d, *J* = 17.6, 1H), 4.12 (d, *J* = 10.0 Hz, 1H), 3.97 (d, *J* = 10.0 Hz, 1H), 3.70 (s, 3H), 2.07 – 1.95 (m, 1H), 1.92 – 1.81 (m, 1H), 1.11 – 1.02 (m, 21H), 0.83 (t, *J* = 7.6 Hz, 3H);

^{13}C NMR (101 MHz, $CDCl_3$) δ 196.9, 184.4, 165.3, 135.8, 132.8, 128.9, 128.6, 127.2, 116.2, 101.4, 93.0, 63.2, 53.0, 52.7, 21.9, 18.0, 12.2, 8.2;

HRMS (ESI) calcd for $[M+H]^+$: $C_{27}H_{41}O_5Si$, m/z: 473.2723, observed: 473.2723.

Chiral HPLC spectrum of racemic **2q**:

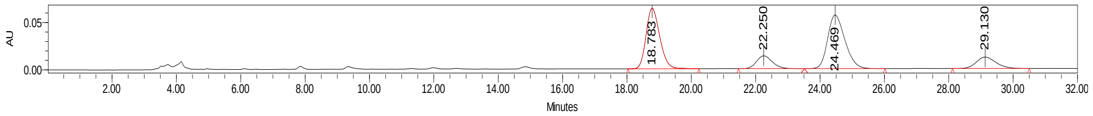

	Retention Time	Area	% Area
1	18.783	1888263	38.02
2	22.250	463104	9.32
3	24.469	2101779	42.32
4	29.130	513154	10.33

*Chiral HPLC spectrum of cat-***2q**:

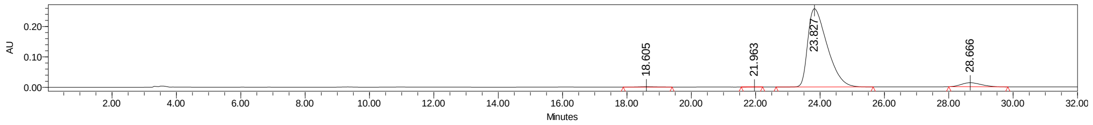

	Retention Time	Area	% Area
1	18.605	37354	0.33
2	21.963	8416	0.07
3	23.827	10770467	94.49
4	28.666	582612	5.11

	Retention Time	Area	% Area

1	18.605	37354	0.35
2	23.827	10770467	99.65

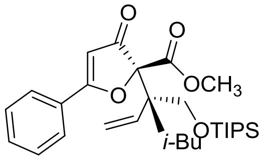

2r

Methyl-(*S*)-3-oxo-5-phenyl-2-((*S*)-3-(((triisopropylsilyl)oxy)methyl)hept-1-en-3-yl)-2,3-dihydrofuran-2-carboxylate;

0.1 mmol scale reaction;

colourless oil; 42 mg, 84% yield, 98%/99% ee, 5:1 dr; $[a]_D^{25}$ = −140.0 (c 0.20, CH_2Cl_2); Determined by HPLC analysis [Daicel chiralpak IE, n-hexane/i-PrOH = 95/5, 1.0 mL/min, λ = 254 nm, t_1 = 14.91 min, t_2 = 16.57 min, t_3 = 20.88 min, t_4 = 22.94 min];

^{1}H NMR (400 MHz, $CDCl_3$) δ 7.83 – 7.77 (m, 2H), 7.55 – 7.49 (m, 1H), 7.48 – 7.41 (m, 2H), 6.23 (dd, *J* = 18.0, 11.2 Hz, 1H), 5.89 (s, 1H), 5.21 (d, *J* = 11.2 Hz,1H),5.13 (d, *J* = 18.0 Hz,1H), 4.06 (dd, *J* = 26.0, 10.0 Hz, 2H), 3.61 (s, 3H), 1.65 – 1.58 (m, 1H), 1.55 – 1.52 (m, 2H), 1.03 (dd, *J* = 6.4, 2.5 Hz, 21H), 0.80 – 0.76 (m, 6H);

^{13}C NMR (101 MHz, $CDCl_3$) δ 196.1, 183.7, 164.0, 135.4, 131.8, 127.9, 127.5, 126.2, 114.3, 100.4, 92.1, 63.5, 52.4, 51.6, 37.9, 28.7, 22.7, 17.1, 11.2;

HRMS (ESI) calcd for $[M+H]^+$: $C_{29}H_{45}O_5Si$, m/z: 501.3036, observed: 501.3032.

Chiral HPLC spectrum of racemic **2r**:

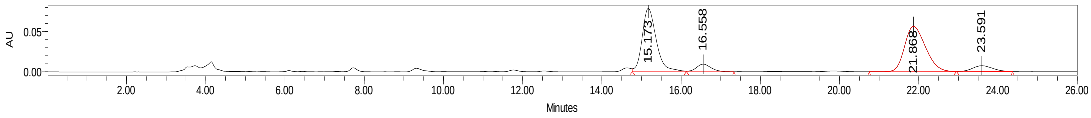

	Retention Time	Area	% Area
1	15.173	1955054	43.20
2	16.558	241884	5.34
3	21.868	2067695	45.69
4	23.591	261341	5.77

*Chiral HPLC spectrum of cat-***2r**:

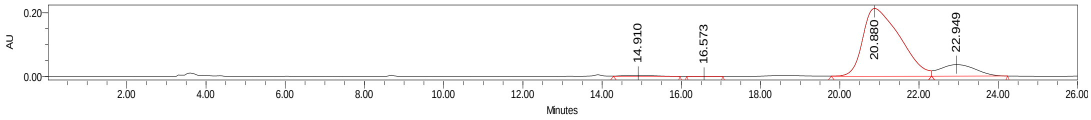

	Retention Time	Area	% Area
1	14.910	155684	0.97
2	16.573	1948	0.01
3	20.880	13689746	85.43
4	22.949	2176215	13.58

	Retention Time	Area	% Area
1	14.911	127671	0.92
2	20.880	13689746	99.08

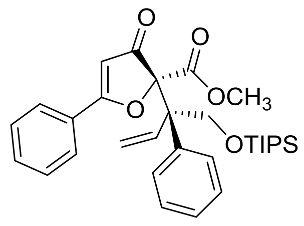

2s'

Methyl-(*R*)-3-oxo-5-phenyl-2-((*R*)-2-phenyl-1-((triisopropylsilyl)oxy)but-3-en-2-yl)-2,3-dihydrofuran-2-carboxylate;

0.086 mmol scale reaction;

colourless oil; 40 mg, 88% yield, 83% ee, 19:1 dr; $[a]_D^{25}$ = 103.4 (c 0.35, CH_2Cl_2); Determined by HPLC analysis [Daicel chiralpak IC, n-hexane/i-PrOH = 95/5, 1.0 mL/min, λ = 254 nm, t_1 = 10.85 min, t_2 = 14.31 min];

^{1}H NMR (400 MHz, $CDCl_3$) δ 7.72 (d, *J* = 7.6 Hz, 2H), 7.54 (t, *J* = 7.2 Hz, 1H), 7.45 (t, *J* = 8.0 Hz, 4H), 7.20-7.10 (m, 3H), 6.87 (dd, *J* = 18.0, 11.2 Hz, 1H), 5.78 (s, 1H), 5.45 (d, *J* = 11.6 Hz, 1H), 5.39 (d, *J* = 18.0 Hz, 1H), 4.69 (d, *J* = 10.0 Hz, 1H), 4.54 (d, *J* = 10.0 Hz, 1H), 3.65 (s, 3H), 1.0-0.96 (m, 21H);

^{13}C NMR (101 MHz, $CDCl_3$) δ 196.1, 184.1, 165.4, 137.9, 137.7, 132.8, 129.3, 128.8, 128.2, 127.4, 127.1, 127.0, 116.9, 101.3, 92.8, 64.5, 58.2, 53.0, 18.0, 12.1;

HRMS (ESI) calcd for $[M+H]^+$: $C_{31}H_{41}O_5Si$, m/z: 521.2723, observed: 521.2723.

Chiral HPLC spectrum of racemic **2s'**:

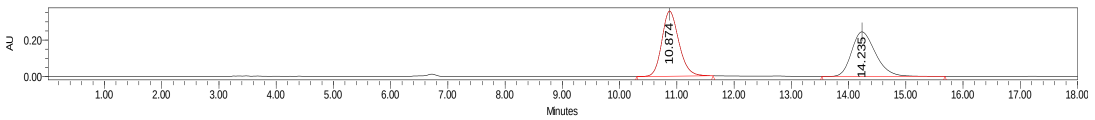

	Retention Time	Area	% Area
1	10.874	7344776	50.99
2	14.235	7058208	49.01

*Chiral HPLC spectrum of cat-***2s'**:

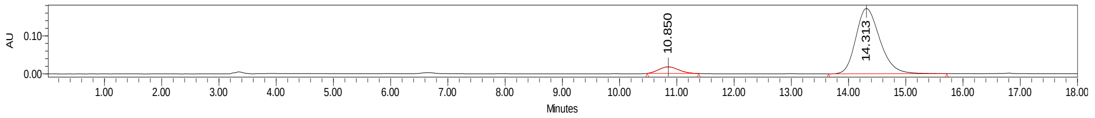

	Retention Time	Area	% Area
1	10.850	465328	8.46
2	14.313	5036542	91.54

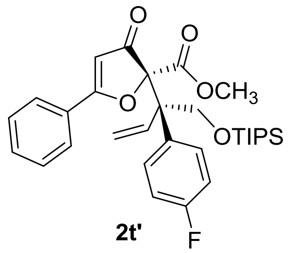

2t'

Methyl-(*R*)-2-((*R*)-2-(4-fluorophenyl)-1-((triisopropylsilyl)oxy)but-3-en-2-yl)-3-oxo-5-phenyl-2,3-dihydrofuran-2-carboxylate

0.175 mmol scale reaction;

colourless oil; 88 mg, 94% yield, 91% ee, 15:1 dr; $[a]_D^{25}$ = 118.4 (c 0.38, CH_2Cl_2); Determined by HPLC analysis [Daicel chiralpak IE, n-hexane/i-PrOH = 95/5, 1.0 mL/min, λ = 254 nm, t_1 = 10.28 min, t_2 = 11.87 min, t_3 = 13.31 min, t_4 = 17.88 min];

^{1}H NMR (400 MHz, $CDCl_3$) δ 7.71 (d, *J* = 7.2 Hz, 2H), 7.55 (t, *J* = 7.2 Hz, 1H), 7.49 – 7.39 (m, 4H), 6.84 (t, *J* = 8.8 Hz, 2H), 6.77 (dd, *J* = 18.0, 11.2 Hz, 1H), 5.76 (s, 1H), 5.50 (d, *J* = 11.6 Hz, 1H), 5.41 (d, *J* = 18.0 Hz, 1H), 4.78 (d, *J* = 10.0 Hz, 1H), 4.52 (d, *J* = 10.0 Hz, 1H), 3.68 (s, 3H), 1.02 – 0.92 (m, 21H);

^{13}C NMR (101 MHz, $CDCl_3$) δ 196.12, 184.35, 165.25, 161.7 (d, J = 244 Hz), 137.75, 133.30 (d, *J* = 3.0 Hz), 133.00, 131.22 (d, *J* = 8 Hz), 128.90, 128.03, 127.07, 117.03, 114.0 (d, J= 21 Hz), 101.2, 92.59, 63.92, 57.61, 53.10, 17.98, 12.08;

HRMS (ESI) calcd for $[M+Na]^+$: $C_{31}H_{39}O_5SiFNa$, m/z: 561.2448, observed: 561.2145.

Chiral HPLC spectrum of racemic **2t'**:

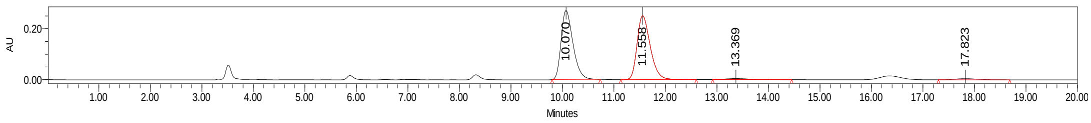

	Retention Time	Area	% Area
1	10.070	4338167	47.99
2	11.558	4434222	49.05
3	13.369	130100	1.44
4	17.823	137668	1.52

*Chiral HPLC spectrum of cat-***2t'**:

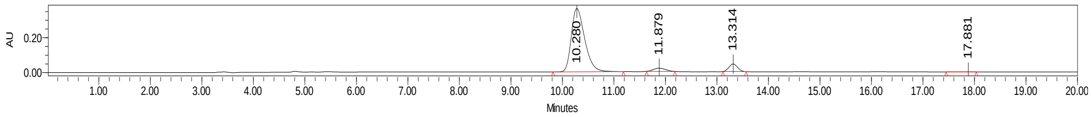

	Retention Time	Area	% Area
1	10.280	6570702	89.04
2	11.879	303755	4.12
3	13.314	504245	6.83
4	17.881	649	0.01

	Retention Time	Area	% Area
1	10.280	6570702	95.58
2	11.879	303755	4.42

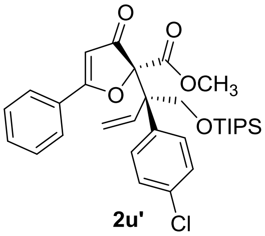

2u'

Methyl-(*R*)-2-((*R*)-2-(4-chlorophenyl)-1-((triisopropylsilyl)oxy)but-3-en-2-yl)-3-oxo-5-phenyl-2,3-dihydrofuran-2-carboxylate

0.11 mmol scale reaction;

colourless oil; 57 mg, 94% yield, 93% ee, 15:1 dr; $[a]_D^{25}$ = 96.9 (c 0.43, CH_2Cl_2); Determined by HPLC analysis [Daicel chiralpak IE, n-hexane/i-PrOH = 95/5, 1.0 mL/min, λ = 254 nm, t_1 = 8.83 min, t_2 = 9.79 min, t_3 = 11.72 min, t_4 = 12.60 min];

^{1}H NMR (400 MHz, $CDCl_3$) δ 7.73 – 7.68 (m, 2H), 7.56 (t, *J* = 7.6 Hz, 1H), 7.47 (t, *J* = 7.6 Hz, 2H), 7.40 (d, *J* = 8.8 Hz, 2H), 7.14 (d, *J* = 8.4 Hz, 2H), 6.77 (dd, *J* = 18.0, 11.2 Hz, 1H), 5.78 (s, 1H), 5.51 (d, *J* = 11.2 Hz, 1H), 5.38 (d, *J* = 18.0 Hz, 1H), 4.74 (d, *J* = 10.0 Hz, 1H), 4.49 (d, *J* = 10.0 Hz, 1H), 3.68 (s, 3H), 1.00 – 0.91 (m, 21H);

^{13}C NMR (101 MHz, $CDCl_3$) δ 196.0, 184.4, 165.2, 137.4, 136.3, 133.0, 132.9, 131.0, 128.9, 128.0, 127.4, 127.1, 117.3, 101.2, 92.4, 63.9, 57.7, 53.1, 18.0, 12.1;

HRMS (ESI) calcd for $[M+H]^+$: $C_{31}H_{40}O_5SiCl$, m/z: 555.2334, observed: 555.2329.

Chiral HPLC spectrum of racemic **2u'**:

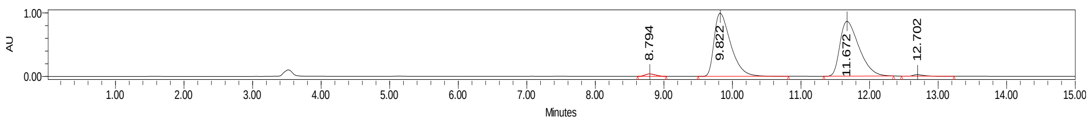

	Retention Time	Area	% Area
1	8.794	393809	1.16
2	9.822	16732109	49.11
3	11.672	16716039	49.06
4	12.702	230871	0.68

*Chiral HPLC spectrum of cat-***2u'**:

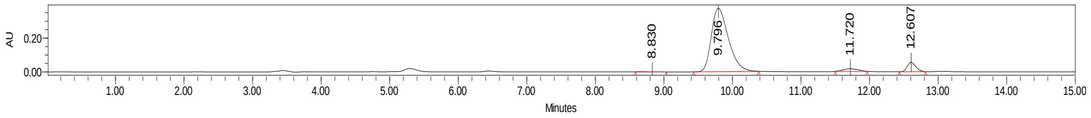

	Retention Time	Area	% Area
1	8.830	274	0.00
2	9.796	6489183	90.23
3	11.720	226096	3.14
4	12.607	476436	6.62

	Retention Time	Area	% Area
1	9.796	6489183	96.63
2	11.720	226096	3.37

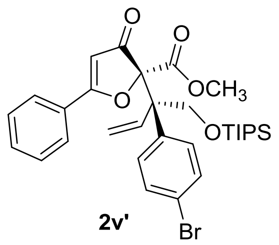

2v'

Methyl-(*R*)-2-((*R*)-2-(4-bromophenyl)-1-((triisopropylsilyl)oxy)but-3-en-2-yl)-3-oxo-5-phenyl-2,3-dihydrofuran-2-carboxylate

0.082 mmol scale reaction;

colourless oil; 45 mg, 92% yield, 91% ee, 15:1 dr; $[a]_D^{25}$ = 98.4 (c 0.44, CH_2Cl_2); Determined by HPLC analysis [Daicel chiralpak IE, n-hexane/i-PrOH = 95/5, 1.0 mL/min, λ = 254 nm, t_1 = 9.45 min, t_2 = 9.96 min, t_3 = 11.98 min, t_4 = 12.74 min];

^{1}H NMR (400 MHz, $CDCl_3$) δ 7.71 (d, *J* = 8.4 Hz, 2H), 7.56 (t, *J* = 6.8 Hz, 1H), 7.47 (t, *J* = 7.6 Hz, 2H), 7.34-7.26 (m, 5H), 6.77 (dd, *J* = 18.0, 11.2 Hz, 1H), 5.78 (s, 1H), 5.50 (d, *J* = 11.2 Hz, 1H), 5.37 (d, *J* = 18.0 Hz, 1H), 4.71 (d, *J* = 10.0 Hz, 1H), 4.47 (d, *J* = 10.0 Hz, 1H), 3.68 (s, 3H), 1.00-0.91 (m, 21H);

^{13}C NMR (101 MHz, $CDCl_3$) δ 195.9, 184.4, 165.2, 137.3, 136.9, 133.1, 131.3, 130.4, 128.9, 128.0, 127.1, 121.2, 117.3, 101.2, 92.4, 64.0, 57.8, 53.2 18.0, 12.1;

HRMS (ESI) calcd for $[M+H]^+$: $C_{31}H_{40}O_5SiBr$, m/z: 599.1828, 601.1808, observed: 599.1832, 601.1818.

Chiral HPLC spectrum of racemic **2v**:

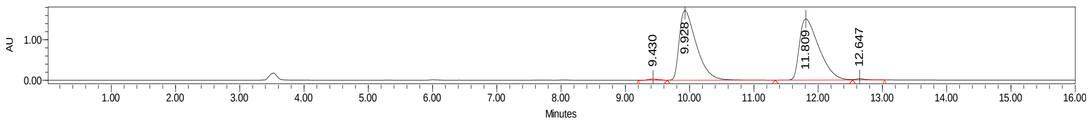

	Retention Time	Area	% Area
1	9.430	328160	0.51
2	9.928	31750300	49.39
3	11.809	31899049	49.62
4	12.647	302893	0.47

*Chiral HPLC spectrum of cat-***2v'**:

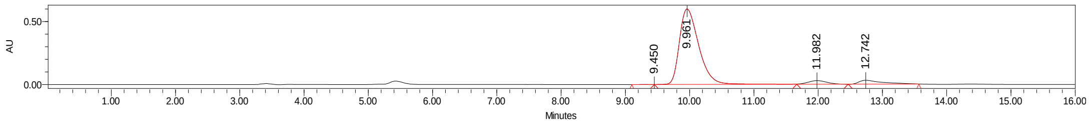

	Retention Time	Area	% Area
1	9.450	1570	0.01
2	9.961	12485592	90.39
3	11.982	565323	4.09
4	12.742	760583	5.51

	Retention Time	Area	% Area
1	9.961	12485592	95.67
2	11.982	565323	4.33

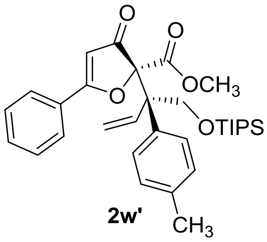

2w'

Methyl-(*R*)-3-oxo-5-phenyl-2-((*R*)-2-(p-tolyl)-1-((triisopropylsilyl)oxy)but-3-en-2-yl)-2,3-dihydrofuran-2-carboxylate

0.1 mmol scale reaction;

colourless oil; 42.7 mg, 80% yield, 62% ee, 15:1 dr; $[a]_D^{25}$ = 55.5 (c 0.42, CH_2Cl_2); Determined by HPLC analysis [Daicel chiralpak IA, n-hexane/i-PrOH = 97/3, 1.0 mL/min, λ = 254 nm, t_1 = 7.76 min, t_2 = 8.36 min, t_3 = 9.32 min, t_4 = 9.99 min];

^{1}H NMR (400 MHz, $CDCl_3$) δ 7.77 – 7.73 (m, 2H), 7.57 – 7.52 (m, 1H), 7.50 – 7.44 (m, 2H), 7.37 – 7.33 (m, 2H), 7.01 (d, *J* = 8.4 Hz, 2H), 6.94 (dd, *J* = 17.6, 11.2 Hz, 1H), 5.81 (s, 1H), 5.49 (d, *J* = 11.2 Hz, 1H), 5.34 (d, *J* = 18.0 Hz, 1H), 4.50 (s, 2H), 3.64 (s, 3H), 2.25 (s, 3H), 0.96 – 0.90 (m, 21H).

^{13}C NMR (101 MHz, $CDCl_3$) δ 196.0, 183.9, 165.5, 137.6, 136.5, 135.1, 132.7, 129.1, 128.8, 128.4, 128.2, 127.1, 116.7, 101.3, 92.8, 65.3, 58.0, 53.0, 21.0, 18.0, 12.1;

HRMS (ESI) calcd for $[M+H]^+$: $C_{32}H_{43}O_5Si$, m/z: 535.2880, observed: 535.2881.

Chiral HPLC spectrum of racemic **2w'**:

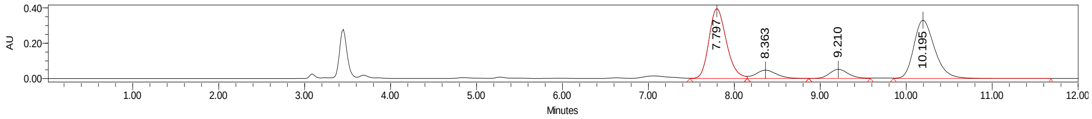

	Retention Time	Area	% Area
1	7.797	5582742	43.81
2	8.363	804876	6.32
3	9.210	806979	6.33
4	10.195	5548133	43.54

*Chiral HPLC spectrum of cat-***2w'**:

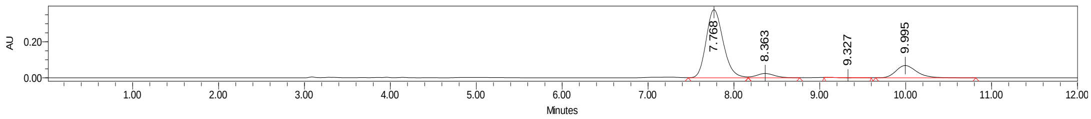

	Retention Time	Area	% Area
1	7.768	5222662	77.31
2	8.363	363715	5.38
3	9.327	13037	0.19
4	9.995	1156450	17.12

	Retention Time	Area	% Area
1	7.768	5222662	81.87
2	9.995	1156450	18.13

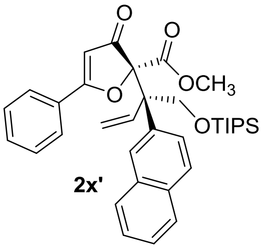

2x'

Methyl-(*R*)-2-((*R*)-2-(naphthalen-2-yl)-1-((triisopropylsilyl)oxy)but-3-en-2-yl)-3-oxo-5-phenyl-2,3-dihydrofuran-2-carboxylate

0.167 mmol scale reaction;

colourless oil; 76 mg, 80% yield, 83%/98% ee, 8:1 dr; $[a]_D^{25}$ = 75.2 (c 0.38, CH_2Cl_2); Determined by HPLC analysis [Daicel chiralpak IE, n-hexane/i-PrOH = 90/10, 1.0 mL/min, λ = 254 nm, t_1 = 9.94 min, t_2 = 11.32 min, t_3 = 13.42 min, t_4 = 16.98 min];

^{1}H NMR (400 MHz, $CDCl_3$) δ 7.92 (s, 1H), 7.74 – 7.66 (m, 4H), 7.64 – 7.62 (m, 2H), 7.54 – 7.47 (m, 1H), 7.45 – 7.36 (m, 4H), 6.96 (dd, *J* = 18.0, 11.6 Hz, 1H), 5.74 (s, 1H), 5.56 (d, *J* = 11.6 Hz, 1H), 5.41 (d, *J* = 17.6 Hz, 1H), 4.80 (d, *J* = 10.0 Hz, 1H), 4.59 (d, *J* = 10.0 Hz, 1H), 3.65 (s, 3H), 1.01 – 0.92 (m, 21H);

^{13}C NMR (101 MHz, $CDCl_3$) δ 196.0, 184.2, 165.4, 137.7, 135.4, 132.8, 132.3, 129.1, 128.8, 128.2, 127.2, 127.1, 126.6, 125.8, 125.5, 117.2, 101.3, 92.7, 64.5, 58.4, 53.1, 18.0, 12.1;

HRMS (ESI) calcd for $[M+H]^+$: $C_{35}H_{43}O_5Si$, m/z: 571.2880, observed: 571.2885.

Chiral HPLC spectrum of racemic **2x'**:

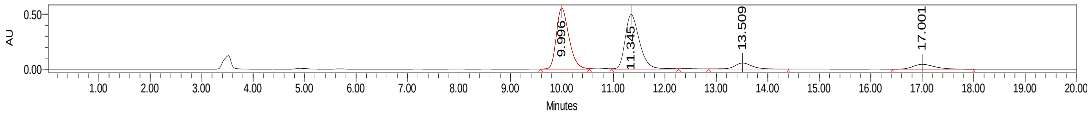

	Retention Time	Area	% Area
1	9.996	9498852	43.90
2	11.345	9604468	44.39
3	13.509	1287077	5.95
4	17.001	1246177	5.76

*Chiral HPLC spectrum of cat-***2x'**:

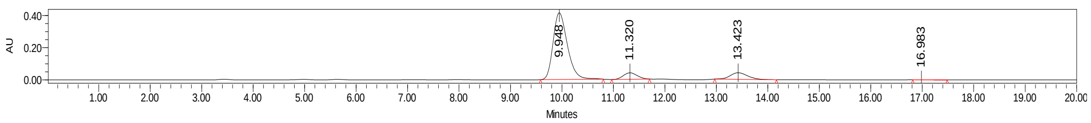

	Retention Time	Area	% Area
1	9.948	8002611	82.33
2	11.320	750181	7.72
3	13.423	958213	9.86
4	16.983	9070	0.09

	Retention Time	Area	% Area
1	9.948	8002611	91.43
2	11.320	750181	8.57

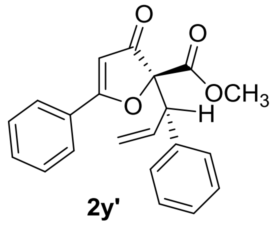

2y'

Methyl-(S)-3-oxo-5-phenyl-2-((S)-1-phenylallyl)-2,3-dihydrofuran-2-carboxylate

0.1 mmol scale reaction;

White Solid; 32.3 mg, 97% yield, 95% ee, 12:1 dr;$[a]_D^{25}$ = -166.4 (c 0.86, CH_2Cl_2);Determined by HPLC analysis [Daicel chiralpak IA, n-hexane/i-PrOH = 93/7, 1.0 mL/min, λ = 254 nm, t_1 = 10.75 min, t_2 = 11.64 min, t_3 = 12.65 min, t_4 = 14.01 min];

^{1}H NMR (400 MHz, $CDCl_3$)7.79 (d, J = 7.4 Hz, 2H), 7.57 (t, J = 7.2 Hz, 1H), 7.48 (t, J = 7.6 Hz, 2H), 7.27 – 7.16 (m, 2H), 7.16 – 7.01 (m, 3H), 6.37 – 6.28 (m, 1H), 5.62 (s, 1H), 5.41 (d, J = 17.2 Hz, 1H), 5.30 (d, J = 10.4 Hz, 1H), 4.54 (d, J = 8.4 Hz, 1H), 3.82 (s, 3H);

^{13}C NMR (101 MHz, $CDCl_3$) δ 196.2, 186.0, 165.1, 135.5, 134.7, 133.2, 129.2, 129.0, 128.3, 128.0, 127.6, 127.3, 118.7, 100.4, 94.5, 53.8, 53.4;

HRMS (ESI) calcd for $[M+H]^+$: $C_{21}H_{19}O_4$, m/z: 335.1278, observed: 335.1278.

Chiral HPLC spectrum of racemic **2y'**:

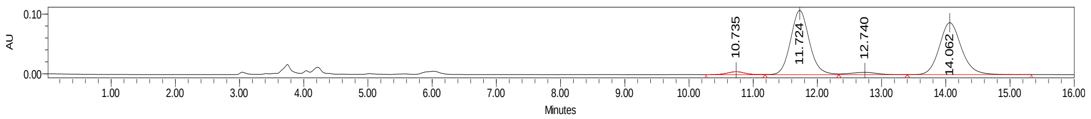

	Retention Time	Area	% Area
1	10.735	96320	2.15
2	11.724	2184929	48.84
3	12.740	110046	2.46
4	14.062	2082214	46.55

*Chiral HPLC spectrum of cat-***2y'**:

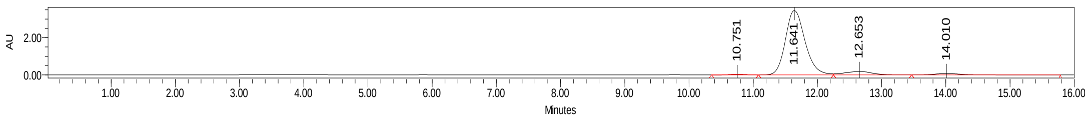

	Retention Time	Area	% Area
1	10.751	354964	0.44
2	11.641	73359666	90.42
3	12.653	5423832	6.69
4	14.010	1990834	2.45

	Retention Time	Area	% Area
1	11.641	73359666	97.59
2	14.010	1814793	2.41

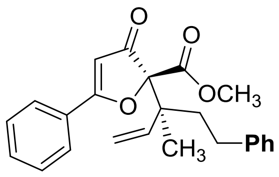

(*S*,*S*)-2z

Methyl-(*S*)-2-((*S*)-3-methyl-5-phenylpent-1-en-3-yl)-3-oxo-5-phenyl-2,3-dihydrofuran-2-carboxylate

0.1 mmol scale reaction;

Clear Oil; 35.3 mg, 94% yield, 98%/94% ee, 5:1 dr;[a]$_D^{25}$ = -105.9 (c 0.77, CH_2Cl_2); Determined by HPLC analysis [Daicel chiralpak IA, n-hexane/i-PrOH = 95/5, 1.0 mL/min, λ = 254 nm, t_1 = 11.39 min, t_2 = 12.02 min, t_3 = 14.11 min, t_4 = 17.29 min];

^{1}H NMR (400 MHz, $CDCl_3$) δ 7.85 (d, *J* = 7.6 Hz, 2H), 7.58 (t, *J* = 7.2 Hz, 1H), 7.50 (t, *J* = 7.2 Hz, 2H), 7.25 – 7.22 (m, 2H), 7.14 (t, *J* = 8.8 Hz, 3H), 6.12 (dd, *J* = 17.2, 10.8 Hz, 1H), 5.98 (s, 1H), 5.28(d, *J* = 10.8 Hz, 1H), 5.18 (d, *J* = 10.8 Hz, 1H), 3.74 (s, 3H), 2.59 – 2.40 (m, 2H), 2.13 – 1.87 (m, 2H), 1.44 (s, 3H);

^{13}C NMR (101 MHz, $CDCl_3$) δ 196.4, 184.9, 165.0, 142.3, 139.1, 133.1, 129.0, 128.5, 128.4, 128.2, 127.3, 125.8, 116.7, 101.5, 94.3, 52.9, 48.2, 36.0, 30.4, 16.1;

HRMS (ESI) calcd for $[M+Na]^+$: $C_{24}H_{24}NaO_4$, m/z:399.1566, observed: 399.1569.

Chiral HPLC spectrum of racemic **2z**:

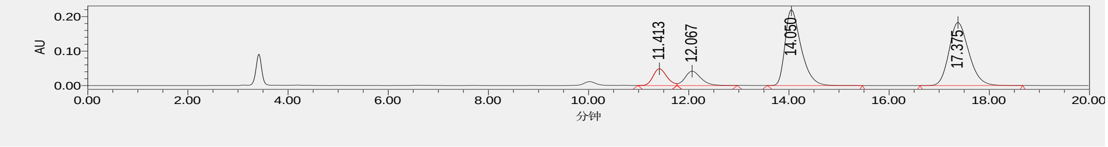

	Retention Time	Area	% Area
1	11.413	926666	7.87
2	12.067	930932	7.91
3	14.050	4886496	41.52
4	17.375	5024051	42.69

*Chiral HPLC spectrum of cat-***2z** (*E*-**1z** as the starting material):

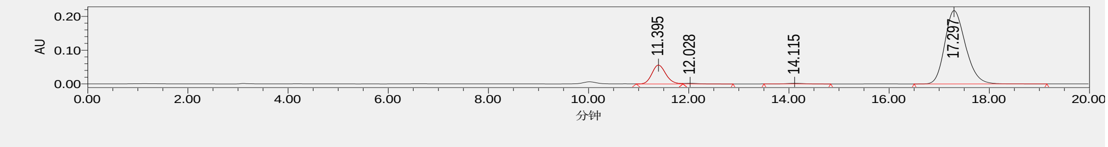

	Retention Time	Area	% Area
1	11.395	1076564	14.56
2	12.028	38790	0.52
3	14.115	46882	0.63
4	17.297	6233078	84.28

	Retention Time	Area	% Area
1	14.115	42523	0.68
2	17.297	6233078	99.32

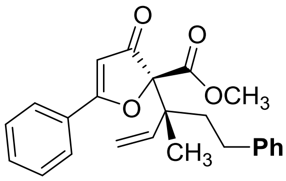

(*S*,*R*)-**2z'**

Methyl-(*S*)-2-((*R*)-3-methyl-5-phenylpent-1-en-3-yl)-3-oxo-5-phenyl-2,3-dihydrofuran-2-carboxylate

0.1 mmol scale reaction;

Clear Oil; 36 mg, 96% yield, 99%/97% ee, 9:1 dr; $[a]_D^{25}$ = -177.1 (c 78, CH_2Cl_2); Determined by HPLC analysis [Daicel chiralpak IA, n-hexane/i-PrOH = 95/5, 1.0 mL/min, λ = 254 nm, t_1 = 10.45 min, t_2 = 11.35 min, t_3 = 14.08 min, t_4 = 17.26 min];

^{1}H NMR (400 MHz, $CDCl_3$) δ 7.85 (d, *J* = 7.2 Hz, 2H), 7.58 (t, *J* = 7.6 Hz, 1H), 7.50 (t, *J* = 7.6 Hz, 2H), 7.25 – 7.21 (m, 2H), 7.20 – 7.05 (m, 3H), 6.08 (dd, *J* = 17.2, 10.8 Hz, 1H), 5.96 (s, 1H), 5.25 (d, *J* = 10.8 Hz, 1H),5.18 (d, *J* = 10.8 Hz, 1H), 3.74 (s, 3H), 2.60 – 2.42 (m, 2H), 2.09 – 1.89 (m, 2H), 1.49 (s, 3H);

^{13}C NMR (101 MHz, $CDCl_3$) δ 196.7, 184.7, 165.0, 142.3, 138.4, 133.1, 129.0, 128.5, 128.4, 128.2, 127.3, 125.9, 116.8, 101.5, 94.4, 52.9, 47.6, 37.0, 30.2, 16.1;

HRMS (ESI) calcd for $[M+Na]^+$: $C_{24}H_{24}NaO_4$, m/z: 399.1566, observed: 399.1574.

Chiral HPLC spectrum of racemic **2z**:

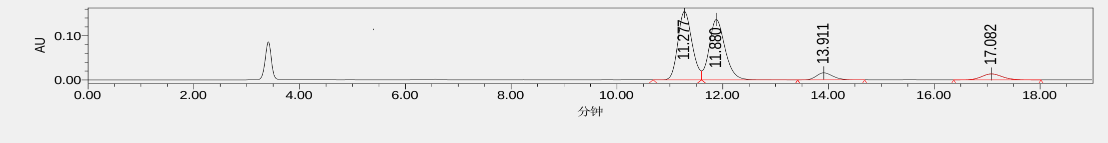

	Retention Time	Area	% Area
1	11.277	2866087	44.66
2	11.880	2845926	44.34
3	13.911	342326	5.33
4	17.082	363713	5.67

Chiral HPLC spectrum of cat-**2z'** (*Z*-**1z'** as the starting material):

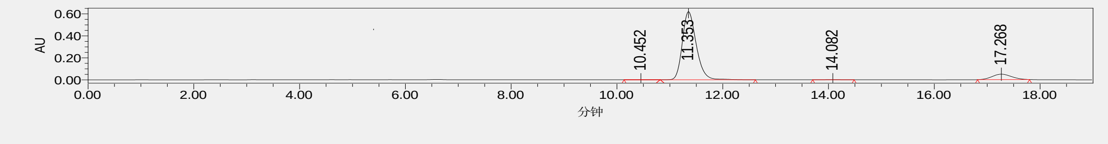

	Retention Time	Area	% Area
1	10.452	14113	0.11
2	11.353	11313502	89.70
3	14.082	24239	0.19
4	17.268	1261262	10.00

	Retention Time	Area	% Area
1	10.452	14113	0.12
2	11.353	11313502	99.88

General procedure for the gram-scale reaction of 1b and 1k

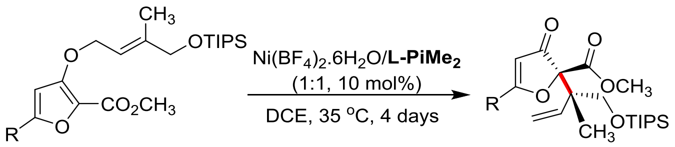

1b, R = Ph, 1 mmol, 458 mg
1k, R = 1-Nap, 1 mmol, 508 mg

2b, 439 mg, 96% yield,
2k, 484 mg, 95% yield,

In a test tube with a magnetic stirring bar, *N*,*N'*-dioxide ligand **L-PiMe$_2$** (54 mg, 0.1 mmol, 10 mol %), Ni(BF$_4$)$_2$ 6H$_2$O (34 mg, 0.1 mmol, 10 mol %) in CH_2Cl_2 (10.0 mL) were stirred at 35 ^{o}C for 1h. After that the solvent become reseda homogeneous solution. Then removing CH_2Cl_2 and adding substrates **1b** (458 mg, 1.0 mmol) or **1k** (508 mg, 1.0 mmol), DCE (10.0 mL). The mixture was stirred at 35 ^{o}C for 4 days. The reaction mixture was detected by TLC. After completion, flash column chromatography was carried out to provide the desired product **2b** (439 mg, 96% yield, 98% ee, 10:1 dr) or **2k** (484 mg, 95% yield, 98% ee, 9:1 dr).

General procedure for the deprotection/cyclization reaction and analytical, spectral characterization data of the products

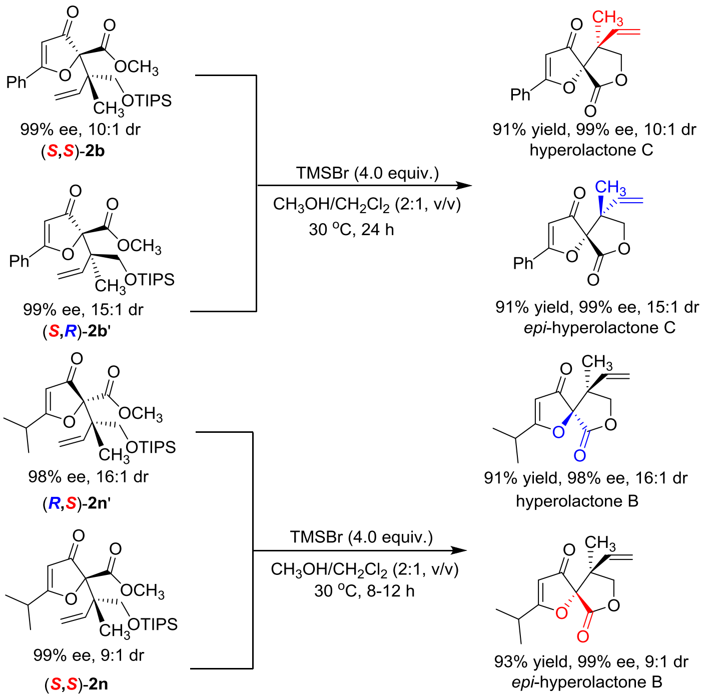

To a solution of the compound **2** (0.1 mmol) in CH_3OH/CH_2Cl_2 (1.5 mL, 2:1, v/v), TMSBr (0.4 mmol, 4.0 equiv.) was added, the reaction mixture was stirred at 30 ^{o}C for 8-24 h. Then the reaction was quenched by the addition of a saturated aq. sodium bicarbonate solution (1 mL) and diluted with water (1 mL).The product was extracted with ethyl acetate (3×10 mL). The combined organic extracts were washed with brine (20 mL) and concentrated in vacuo. Flash chromatography (hexane/ethyl acetate, 10:1) afforded the desired product.

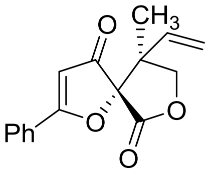

hyperolactone C

(5*S*,9*S*)-9-methyl-2-phenyl-9-vinyl-1,7-dioxaspiro[4.4]non-2-ene-4,6-dione ;

White solid; 24.5 mg, 91% yield, 99% ee, 10:1 dr; $[a]_D^{25}$ = −280.7 (c 0.33, CH_2Cl_2); $[a]_D^{25}$ = −264.5 (c 0.11, $CHCl_3$), ref. { Crockett, S. L., Schühly, W., Belaj, F., Khan, I. A. Hyperolactone C. *Acta Crystallogr. Sect. E* **60**, o2174-o2176 (2004), $[a]_D$ = − 270.7 (*c* 0.11, $CHCl_3$)}; Determined by HPLC analysis [Daicel chiralpak Lux 5u Cellulose-2, n-hexane/i-PrOH = 90/10, 1.0 mL/min, λ = 254 nm, t_1 = 22.06 min, t_2 = 29.48 min, t_3 = 31.55 min, t_4 = 38.28 min];

^{1}H NMR (400 MHz, $CDCl_3$) δ 7.85 (d, *J* = 7.6 Hz, 2H), 7.61 (t, *J* = 7.2 Hz, 1H), 7.52 (t, *J* = 7.6 Hz, 2H), 6.05 – 5.93 (m, 2H), 5.28 – 5.23 (m, 2H), 4.97 (d, *J* = 8.4 Hz, 1H), 4.11 (d, *J* = 8.8 Hz, 1H), 1.53 (s, 3H);

^{13}C NMR (101 MHz, $CDCl_3$) δ 196.6, 187.3, 168.1, 134.2, 133.6, 129.1, 127.7, 127.5, 119.1, 100.3, 93.1, 74.1, 48.9, 19.6;

HRMS (ESI) calcd for $[M+H]^+$: $C_{16}H_{15}O_4$, m/z: 271.0964, observed:271.0965.

Chiral HPLC spectrum of racemic hyperolactone C

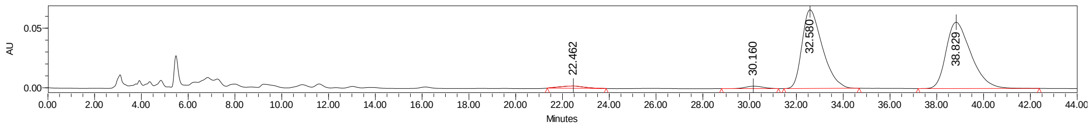

	Retention Time	Area	% Area
1	22.462	139321	1.67
2	30.160	107123	1.28
3	32.580	3989896	47.83
4	38.829	4105386	49.22

Chiral HPLC spectrum of hyperolactone C (major):

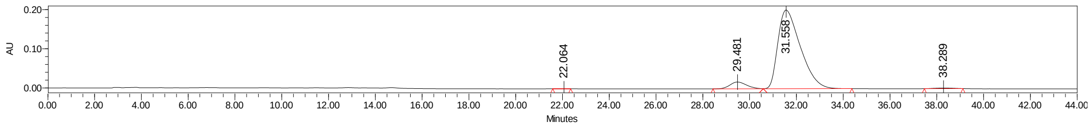

	Retention Time	Area	% Area
1	22.064	780	0.01
2	29.481	866937	6.03
3	31.558	13433316	93.38
4	38.289	84762	0.59

	Retention Time	Area	% Area
1	31.558	13433316	99.37
2	38.289	84762	0.63

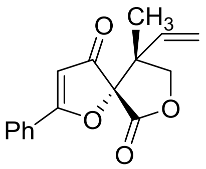

epi-hyperolactone C

(5*S*,9*R*)-9-methyl-2-phenyl-9-vinyl-1,7-dioxaspiro[4.4]non-2-ene-4,6-dione;

White solid; 24.5 mg, 91% yield, 99% ee, 15:1 dr; $[a]_D^{25}$ = −264.8 (c 0.13, CH_2Cl_2); Determined by HPLC analysis [Daicel chiralpak Lux 5u Cellulose-2, n-hexane/i-PrOH = 90/10, 1.0 mL/min, λ = 254 nm, t_1 = 22.33 min, t_2 = 29.27 min, t_3 = 31.95 min, t_4 = 38.15 min];

^{1}H NMR (400 MHz, $CDCl_3$) δ 7.85 (d, *J* = 8.4 Hz, 2H), 7.61 (t, *J* = 6.8 Hz,

1H), 7.53 – 7.49 (m, 2H), 6.14 – 5.99 (m, 2H), 5.46 – 5.26 (m, 2H), 4.78 (d, J = 8.4 Hz, 1H), 4.43 – 4.34 (m, 1H), 1.30 (d, J = 1.2 Hz, 3H);

^{13}C NMR (101 MHz, $CDCl_3$) δ 196.6, 187.5, 168.2, 136.8, 133.7, 129.1, 127.7, 127.5, 116.3, 100.4, 92.3, 73.3, 48.9, 15.5;

HRMS (ESI) calcd for $[M+H]^+$: $C_{16}H_{15}O_4$, m/z: 271.0964, observed: 271.0967.

Chiral HPLC spectrum of epi-hyperolactone C (major):

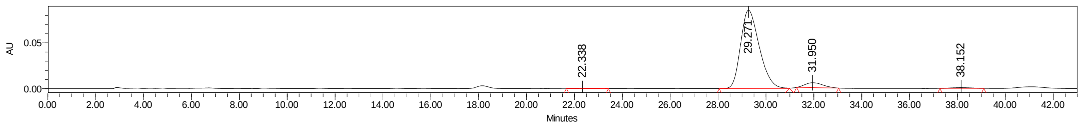

	Retention Time	Area	% Area
1	22.338	5241	0.11
2	29.271	4629592	93.56
3	31.950	281392	5.69
4	38.152	32224	0.65

	Retention Time	Area	% Area
1	22.338	5241	0.11
2	29.271	4629592	99.89

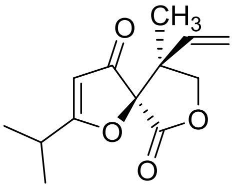

hyperolactone B

(5*R*,9*S*)-2-isopropyl-9-methyl-9-vinyl-1,7-dioxaspiro[4.4]non-2-ene-4,6-dione;

White solid; 21.4 mg, 91% yield, 98% ee, 16:1 dr; $[a]_D^{25}$ = +309 (c 0.5, CH_2Cl_2); $[a]_D^{25}$ = +388.8 (c 0.018, EtOH), ref. { Aramaki, Y., Chiba, K., Tada, M. Spirolactones, Hyperolactone A-D from Hypericum Chinense. *Phytochemistry* **38**, 1419-1421 (1995), $[a]_D$ = +411.1 (*c* 0.018, EtOH)}; Determined by HPLC analysis [Daicel chiralpak Lux 5u Cellulose-2, n-hexane/i-PrOH = 90/10, 1.0 mL/min, λ = 254 nm, t_1 = 16.45 min, t_2 = 19.47 min, t_3 = 32.47 min, t_4 = 34.51 min];

^{1}H NMR (400 MHz, $CDCl_3$) δ 6.01 – 5.87 (m, 1H), 5.44 (d, J = 0.4 Hz, 1H), 5.33 – 5.24 (m, 2H), 4.71 (d, J = 8.4 Hz, 1H), 4.32 (d, J = 8.4 Hz, 1H), 2.92 – 2.79 (m, 1H), 1.29 (dd, J = 6.8, 2.4 Hz, 6H), 1.23 (s, 3H);

^{13}C NMR (101 MHz, $CDCl_3$) δ 201.3, 197.5, 168.1, 136.7, 116.0, 101.2, 91.8, 73.2, 48.4, 30.4, 19.7, 19.4, 15.4;

HRMS (ESI) calcd for $[M+H]^+$: $C_{13}H_{16}O_4$, m/z:237.1121, observed:237.1122.

Chiral HPLC spectrum of racemic hyperolactone B

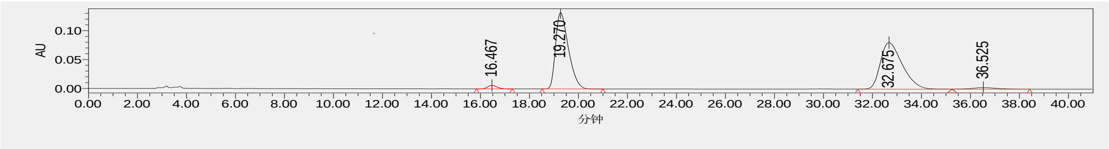

	Retention Time	Area	% Area
1	16.467	163066	1.61
2	19.270	4823824	47.57
3	32.675	4981888	49.13
4	36.525	170963	1.69

Chiral HPLC spectrum of hyperolactone B (major)

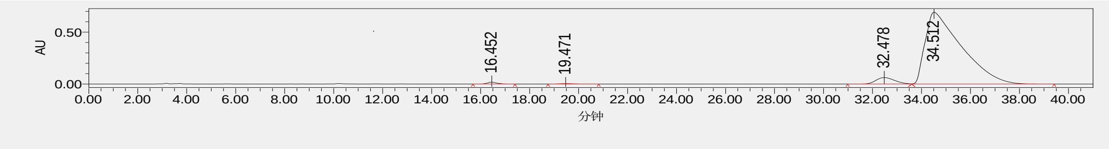

	Retention Time	Area	% Area
1	16.452	474297	0.63
2	19.471	93521	0.12
3	32.478	3316174	4.41
4	34.512	71240802	94.83

	Retention Time	Area	% Area
1	16.452	461832	0.64
2	34.512	71240802	99.36

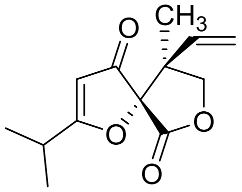

epi-hyperolactone B

(5*S*,9*S*)-2-isopropyl-9-methyl-9-vinyl-1,7-dioxaspiro[4.4]non-2-ene-4,6-dione;

White solid; 22 mg, 93% yield, 99%/92% ee, 9:1 dr; $[a]_D^{25}$ = −297.3 (c 0.30, CH_2Cl_2);Determined by HPLC analysis [Daicel chiralpak Lux 5u Cellulose-2, n-hexane/i-PrOH = 90/10, 1.0 mL/min, λ = 254 nm, t_1 = 16.40 min, t_2 = 18.92 min, t_3 = 32.92 min, t_4 = 36.60 min];

^{1}H NMR (400 MHz, $CDCl_3$) δ 5.92 (dd, *J* = 17.2, 10.8 Hz, 1H), 5.41 – 5.33 (m, 1H), 5.32 – 5.20 (m, 2H), 4.89 (d, *J* = 8.4 Hz, 1H), 4.05 (d, *J* = 8.5 Hz, 1H), 2.94 – 2.76 (m, 1H), 1.41 (s, 3H), 1.28 (dd, *J* = 6.8, 2.8 Hz, 6H)；

^{13}C NMR (101 MHz, $CDCl_3$) δ 201.1, 197.4, 168.1, 134.3, 118.9, 101.1, 92.5, 74.0, 48.4, 30.4, 19.7, 19.3 (2C);

HRMS (ESI) calcd for $[M+H]^+$: $C_{13}H_{16}O_4$, m/z: 237.1121, observed: 237.1122.

Chiral HPLC spectrum of epi-hyperolactone B (major):

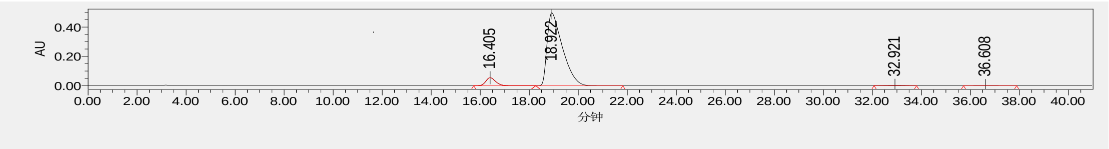

	Retention Time	Area	% Area
1	16.405	1491965	6.42
2	18.922	21563290	92.84
3	32.921	116487	0.50
4	36.608	54273	0.23

	Retention Time	Area	% Area
1	18.922	21563290	99.46
2	32.921	116487	0.54

Supplementary References

1. Aramaki, Y., Chiba, K., Tada, M. Spiro-lactones, Hyperolactone A-D from Hypericum Chinense. Spiro-lactones, hyperolactone A-D from *Hypericum chinense. Phytochemistry* **38**, 1419 (1995).
2. Wasserman, H. H., Lee, G. M. The chemistry of vicinal tricarbonyls. A new synthesis of substituted furans. *Tetrahedron Letters* **35**, 9783 (1994).
3. Du, C., Li, L. Q., Li, Y., Xie, Z. X. Construction of Two Vicinal Quaternary Carbons by Asymmetric Allylic Alkylation: Total Synthesis of Hyperolactone C and (−)-Biyouyanagin A. *Angew. Chem. Int. Ed.* **48**, 7853 (2009).
4. Hecht, S., Amslinger, S., Jauch, J., Kis, K., Trentinaglia, V., Adam, P., Eisenreich, W., Bacher, A., Rohdich, F. Studies on the non-mevalonate isoprenoid biosynthetic pathway. Simple methods for preparation of isotope-labeled (*E*)-1-hydroxy-2-methylbut-2-enyl 4-diphosphate. *Tetrahedron Letters* **43**, 8929 (2002).
5. Evans, D. A., Glorius, F., Burch, J. D. Complex Aldol Reactions for the Construction of Dense Polyol Stereoarrays: Synthesis of the C33−C36 Region of Aflastatin A. *Org. Lett.*, **7**, 3331 (2005)
6. Li, H., Yoo, J. C., Hong, J. H. *Nucleosides, Nucleotides and Nucleic Acids*, Synthesis and anti-HCV Evaluation of 4′-(α)-ethyl and 2′-(β)-methyl-carbodine Analogues. **28**, 809 (2009)
7. Broadley, K. J., Buffat, M. G. P., Burnell, E., Davies, R. H., Moreau, X., Snee, S., and Thomas, E. J. Stereoselective synthesis of oxazolidinonyl-fused piperidines of interest as selective muscarinic (M1) receptor agonists: a novel M1 allosteric modulator. *Org. Biomol. Chem.***14**, 2057 (2016).
